# Beyond the Chromosome: The Prevalence of Unique Extra-Chromosomal Bacteriophages with Integrated Virulence Genes in Pathogenic *Staphylococcus aureus*

**DOI:** 10.1371/journal.pone.0100502

**Published:** 2014-06-25

**Authors:** Bryan Utter, Douglas R. Deutsch, Raymond Schuch, Benjamin Y. Winer, Kathleen Verratti, Kim Bishop-Lilly, Shanmuga Sozhamannan, Vincent A. Fischetti

**Affiliations:** 1 Laboratory of Bacterial Pathogenesis and Immunology, The Rockefeller University, New York, New York, United States of America; 2 Henry M. Jackson Foundation, Bethesda, Maryland, United States of America; 3 Naval Medical Research Center-Frederick, Fort Detrick, Maryland, United States of America; Institut Pasteur Paris, France

## Abstract

In *Staphylococcus aureus,* the disease impact of chromosomally integrated prophages on virulence is well described. However, the existence of extra-chromosomal prophages, both plasmidial and episomal, remains obscure. Despite the recent explosion in bacterial and bacteriophage genomic sequencing, studies have failed to specifically focus on extra-chromosomal elements. We selectively enriched and sequenced extra-chromosomal DNA from *S. aureus* isolates using Roche-454 technology and uncovered evidence for the widespread distribution of multiple extra-chromosomal prophages (ExPΦs) throughout both antibiotic-sensitive and -resistant strains. We completely sequenced one such element comprised of a 43.8 kbp, circular ExPΦ (designated ФBU01) from a vancomycin-intermediate *S. aureus* (VISA) strain. Assembly and annotation of ФBU01 revealed a number of putative virulence determinants encoded within a bacteriophage immune evasion cluster (IEC). Our identification of several potential ExPΦs and mobile genetic elements (MGEs) also revealed numerous putative virulence factors and antibiotic resistance genes. We describe here a previously unidentified level of genetic diversity of stealth extra-chromosomal elements in *S. aureus*, including phages with a larger presence outside the chromosome that likely play a prominent role in pathogenesis and strain diversity driven by horizontal gene transfer (HGT).

## Introduction

Chromosomally integrated prophages are known to play key roles in the pathogenicity and virulence of *S. aureus*. Integrated prophages encode *S. aureus*-specific toxins such as Panton-Valentine leukotoxin (PVL) and staphylococcal enterotoxin A [1], [2], as well as an immune evasion cluster (IEC) locus involved in modulating the immune system to facilitate staphylococcal adaptation to humans. The IEC locus encodes chemotaxis inhibitory proteins (CHIPS), staphylokinase (SAK), staphylococcal enterotoxin type-A/P (SEA/SEP), and staphylococcal complement inhibitor (SCIN) [3]. In addition to chromosomally integrated forms, prophages have been reported to exist as lysogenic linear and circular ‘plasmidial’ elements extra-chromosomally in *Escherichia, Bacillus, Halomonas, Chlamydia*, and *Borrelia* species [4]–[9]. However, the widespread presence of plasmidial prophages has not been described in *S. aureus*.

A report by Casjens in 2003 showed the existence of only two plasmidial prophages in over 80 published bacterial DNA sequences among diverse species [10]. One strain of *Borrelia burgdorferi,* B31, contained 12 different plasmidial prophages, (almost 20 percent of its genome), while *Chlamydia pneumoniae* contained only one [6], [7]. The presence of plasmidial prophages in *B. burgdorferi* and *C. pneumoniae* is attributed to evolutionary pressure on their smaller genomes to remove all non-essential DNA [10], [11]. Plasmidial prophages may have been uncovered in *B. burgdorferi* because of its higher copy number prophages combined with its small chromosome, allowing for the identification and sequencing of these additional, smaller, genetic elements. Thus, plasmidial prophages might not be as rare as this study indicated, but rather difficult to identify. Additional evidence also suggests that lysogeny in the environment favors the plasmidial form. *B. anthracis* strains isolated worldwide have nearly identical chromosomal prophage sequences, yet up to 20% of isolates encode a diverse array of additional inducible phages [9], [12]–[17]. The potential importance of these extra-chromosomal prophages is described in a recent study of *B. anthracis* phages (likely plasmidial) with significant roles in the adaptive behavior of the pathogen [18].

In this study we term transient phages as ‘episomal’, and those existing solely in the cytoplasm as ‘plasmidial’. Two previous reports have examined extra-chromosomal prophages in *S. aureus* [19], [20]. One study reported that lysogenic *S. aureus* phages Φs80b and Φs84b could integrate into the chromosome of *S. aureus* strain s64c but remained plasmidial or solely extra-chromosomal in *S. aureus* strain 8325–4. Thus, the *S. aureus* host cell determined the extra-chromosomal state of these prophages: episomal in *S. aureus* s64c, and plasmidial in 8325–4. In strain s64c, this extra-chromosomal state was reported as ‘transient’, where the integrated and extra-chromosomal states could switch during serial passages [19]. Following this study, an alternative host transcription factor, **σ** ^H^, was found to directly affect integration and excision of *S. aureus* phages; *sigH* deletion from the *S. aureus* genome caused an increase in the episomal state of the phage [20]. Thus, it is likely that certain prophages spend less time chromosomally integrated but rather exist largely in the extra-chromosomal compartment. Such prophages differ significantly from particles resulting from spontaneous induction of integrated prophage, which are produced at such low frequencies (<10^−5^) in the bacterial population that they cannot be detected using current sequencing techniques.

MGEs in both the integrated and circularized states can have major effects on the bacterial cell. For example, a phage-like MGE in *Streptococcus pyogenes* chromosomal island in an M1 serotype (SpyCIM1), can integrate at a specific site in the chromosome during stationary phase of growth, blocking transcription of a mismatch repair gene, *mutL*, thereby increasing the mutation rate. During exponential growth however, SpyCIM1 excises and replicates in the extra-chromosomal compartment of the cell, enabling *mutL* transcription [21]. The integration/excision of phage-like elements has been identified in other bacterial species as well, including the P4-like cryptic prophage of *E. coli* [22], a 42 kb prophage-like element in *Bacillus subtilis* [23], a 14.6 kb element in *Clostridium difficile* [24], and a phage-chromosomal island in the genome of *Enterococcus faecalis* V583 [25]. While the role of the alternating excision versus integration of *S. aureus* ExPΦ is unclear, these studies suggest it will likely have an important role in cellular processes.

Next-generation sequencing (NGS) in *S. aureus* has been biased toward sequencing the bacterial chromosome; not surprisingly, smaller, low-copy, extra-chromosomal elements (like ExPΦ) would have been overlooked unless specifically isolated [10]. Moreover, in some cases the presence of episomal prophages in the extra-chromosomal compartment of *S. aureus* may be a transient event and therefore potentially difficult to detect due to extremely low-copy numbers. In this study, we developed a method to effectively enrich extra-chromosomal DNA that includes low-copy number episomal and/or plasmidial ExPΦs and other extra-chromosomal elements from *S. aureus*. In addition, a NGS approach was used to achieve extremely high coverage and delineate genetic diversity of MGEs. Our results revealed naturally present and potentially widespread ExPΦs in virulent and antibiotic-resistant strains of *S. aureus*. ExPΦs were shown to be either episomal or plasmidial. The complete genomic sequence of one extra-chromosomal *S. aureus* phage ФBU01 from a VISA strain, was assembled and annotated, and found to contain *scn*, *chp*, *sak*, and *sep* IEC virulence genes associated with β-hemolysin containing phages [3]. By specifically targeting the extra-chromosomal compartment of the bacterial cell, we have uncovered an array of previously unidentified genetic diversity overlooked by traditional sequencing approaches, offering new insights into *S. aureus* pathogenesis.

## Materials and Methods

### Bacterial Strains and Growth Conditions for Extra-chromosomal DNA isolation

Bacterial strains used in this study are described in Table S1 in File S1. *Staphylococcus aureus* strains were cultured in Bacto Brain Heart Infusion (#L9043; BHI, Becton Dickinson, Sparks, MD) and incubated at 37°C. *S. aureus* from freezer stocks were revived on BHI agar plates and incubated for 16 to 24 hrs. One to two-day-old single colonies were inoculated into BHI broth and grown overnight (12–15 hrs). Cultures were then diluted 1∶100 in 150 mL BHI and grown to an OD_600_ of 0.6 to 0.9. Cultures were then centrifuged at 4,000 RPM for 20 minutes at 4°C. Cell pellets were used immediately or stored overnight at −20°C.

### Extra-chromosomal DNA Isolation

*S. aureus* cell pellets were resuspended in 12 mL of the QIAGEN (Valencia, CA USA) Resuspension Buffer (50 mM Tris·Cl, pH 8.0; 10 mM EDTA; 100 µg/mL RNaseA) supplemented with 200 µg lysostaphin from *Staphylococcus simulans* (#L9043, Sigma-Aldrich St. Louis, MO), 800 ng of PlySs2 lysin [26], and 12 mg lysozyme (#BP353, Fisher Scientific (Pittsburgh, PA USA). The resuspended cells were incubated at 37°C until the suspension cleared or a maximum of 30 min. Extra-chromosomal DNA was then isolated using the QIAGEN HiSpeed Plasmid Midi Kit (#12643) following modified versions of the user-developed protocol (QP10.doc Aug-01) and HiSpeed Plasmid Purification Handbook (11/2005). To prevent shearing of DNA larger than 50 kb, the QIAGEN QIAfilter cartridge was not used, rather, the supernatant was centrifuged at 20,000×g for 30 minutes at 4°C and filtered using sterile cheese cloth prior to addition to the HiSpeed Midi column. DNA was eluted from the QIAGEN QIAprecipitator with 0.5 mL sterile H_2_O at 65°C.

### Extra-chromosomal DNA manipulation

DNA was separated by electrophoresis at 50 V for 60 min on a 0.7 % Agarose, 1X, TAE gel. DNA was stained with ethidium bromide, and visualized under ultraviolet transillumination on an Alpha Imager HP (Cell Biosciences, Santa Clara, CA USA). Total DNA was estimated by measuring final concentrations of extra-chromosomal DNA preparations using Thermo Scientific (Wilmington, DE, USA) NanoDrop 1000, software version 3.1.0. If needed, samples were concentrated using Amicon Ultra 0.5 mL 30,000 MWCO centrifugal units (#UFC503024, Millipore, Billerica, MA USA); centrifuged at 2000×*g* for 30 min or as needed at 4°C. The centrifugation force was kept below 5000×*g* to prevent shearing of large plasmids (Millipore Technical Publications Protocol WWW-UF). When needed, samples under a total volume of 100 µl were concentrated further by Speedvac (Thermo Scientific Savant) set at low drying rate. Samples were stored at −20°C and shipped overnight on ice to the sequencing facility. Samples treated with Plasmid-Safe linear DNase were incubated for 2–4 hrs at 37°C with 1 µl 25 mM ATP, 2.5 µl 10× Reaction Buffer and 1 µl (10 U) Plasmid-Safe Linear DNase (25 µl reaction volume). Heating at 70°C for 30 min then inactivated the linear DNase.

### Roche-454 sequencing of Extra-chromosomal Samples

‘Whole-genome sequencing’ (WGS) on extra-chromosomal DNA samples was performed using the Roche- (Branford, CT USA) 454 Sequencing Genome Sequencer FLX as described in Margulies *et al.* 2005 [27]. Total DNA needed for sequencing ranged from 600 to 900 ng. Sample DNA libraries were generated using the Roche GS Rapid Library Prep Kit- 12 (05608228001). All DNA was fragmented using a M220 Focused-ultrasonicator (Covaris, Woburn, MA USA), parameters set as followed: Intensity: 3; Duty Cycle: 5%; Cycles per burst: 200; Treatment time: 80 sec. DNA fragmentation samples were fragment end repaired using the following reaction mixture: 2.5 µl RL 10× PNK Buffer; 2.5 µl RL ATP; 1 µl RL dNTP; 1 µl RL T4 Polymerase; 1 µl RL PNK; and 1 µl RL Taq polymerase. The reaction mixture was incubated at 25°C for 20 min and 72°C for 20 min. Twelve Multiplex Identifier (MID) Adaptors (numbers 1–12) of Roche GS Rapid Library MID Adaptors Kit (#05619211001) were ligated to repaired DNA ends. Next, Agencourt AMPure XP beads (#A63880, Beckman Coulter, Indianapolis, IN USA) cleaned up small DNA fragments from extra-chromosomal DNA samples. Libraries were quantitated using the TBS-380 Fluorometer (r-BioPharm, Darmstadt, Germany). The DNA quality was further assessed using the Agilent Bioanalyzer DNA high sensitivity assay (#5067-4626) based on manufacturer's instructions. Roche GS Titanium Sequencing Kit XLR70 (#05233526001) was used according to the manufacturer's protocols and instructions (Roche GS FLX Sequencing Method Manual December 2007). Emulsion PCR Genome sequencing was performed in a high-density picolitre reactors with Roche GS Titanium PicoTiterPlate Kit 70×75 (# 05233682001), Roche GS Titanium LV emPCR Kit (Lib-L) 2 emulsions (05618428001), and Roche GS Titanium emPCR Breaking Kits MV/LV 12pc (#05233658001). Signal processing was performed off-rig on a Linux cluster of 10 nodes connected via gigabit ethernet. Each node contained eight 64-bit processing cores running at 2.3 GHz with 8 GB of RAM. *De novo* assembly of sequences was performed using Roche gsAssembler Newbler at default settings. Final assemblies were submitted to Genbank with the following accession numbers: ΦBU01, KF831354; pBU108a, KF831355; pBU108b, KF831356; and pBU108c, KF831357.

### Nucleotide homology identification of Roche-454 sequencing results of *S. aureus* samples

Contigs generated by Roche gsAssembler Newbler resulting from the Roche-454 sequencing runs were identified as plasmid, bacteriophage, chromosome, or miscellaneous MGE by BLASTN analysis comparing to GenBank database [28] (http://www.nibi.nih.gov/BLAST). Bacteriophage gene identification was performed using BLASTP analysis. Homologies found in the GenBank database were based on greater than 50 percent identity [29]. Bacteriophage ФNM3 (NCBI Reference Sequence: NC_008617.1) was used as a reference for preliminary sequence assembly of φBU01 using MacVector with the PHRAP algorithm at default settings [30], [31].

### Southern blot analysis

Aliquots of 200–400 ng extra-chromosomal DNA were separated (50 volts for 60 min) on a 0.7 % agarose, 1X TAE gel. Invitrogen (Eugene, OR USA) SYBR Safe DNA gel stain (# S33102) was added to the gel at a 1∶10,000 dilution. DNA was treated and transferred from gel to Positively Charged Nylon Transfer Membrane (#RPN303B, GE Healthcare, UK) using standard Southern blot procedures [32]. Hybridization probes were prepared from PCR products generated using primers 5′-CCTGTTGCTTGGGTAACTGTATC-3′ and 5′-AATGGCAGAAAGTGGCTGG-3′ for identified bacteriophage in the NRS19 strain and primers 5′TGCCATTGTGATGAGGAGGG-3′ and 5′-GCAACGCAGATTGTTTGAGTG-3′ for the identified bacteriophage of the NRS26 strain. These PCR products were purified with QIAquick PCR Purification Kit # 28106 and labeled for use as a Southern blot probe using Biotin DecaLabel DNA Labeling Kit (#K0652, Thermo Scientific (Waltman, MA USA). DNA transferred to nylon membrane was pre-hybridized and hybridized based on standard Southern blot procedures [32]. Southern blot probe detection was performed using Fermentas (Glen Burnie, MD USA) Biotin Chromogenic Detection Kit (#K0661) based on manufacturer's instructions.

### Pulsed-field Gel Electrophoresis

Pulsed field gel electrophoresis was performed with a modified procedure from Chung *et al.* 2000 [33]. *S. aureus* cultures were grown overnight, then diluted 1∶100 in BHI, and 10 mL samples were taken at 6, 8, and 24hrs. Samples of φBU01 were also taken at an OD_600_ of 0.12, 0.65, 1.3, 2.0 and 2.5 (24 hrs). Samples were concentrated to an adjusted OD_600_ of 5.0–10.0. Cultures were added to Lonza (Allendale, NJ) SeaPlaque GTG low melting agarose (# 50111) and allowed to solidify. Samples were treated in 0.75 % agarose disks with 100 µg/mL RNase A, 50 µg/mL lysostaphin, 1 mg/mL lysozyme, and 25 µg/mL PlySs2 for 5 hrs. Agarose disks were then treated with 1 mg/mL proteinase K for 17 hours and overnight with *Sma*I. DNA was separated by pulse-field electrophoresis for 23 hours at 6.0 V/cm. Gel was stained with ethidium bromide for 30 min and destained in dH_2_0 for 30 min.

### Sequence assembly of NRS19 ExPΦ

Primers were designed based on preliminary mapping of the contigs to the reference genome (Table S2 in File S1). PCR amplicons used in Sanger sequencing were generated using NRS19 extra-chromosomal DNA as a template. Each primer was designed to sequence specific regions on the PCR product and unknown sequence regions were determined using a primer walking method [34]. Sanger DNA sequencing used in the primer walking method was performed by Genewiz (South Plainfield, NJ USA) [35]. *De novo* assembly of the sequence contigs was performed using MacVector assembly (without template) with the PHRAP assembly algorithm, default settings [30], [31].

### Induction of phage from NRS109, NRS24, VISA NRS19 and NRS26 and DNase treatment

Phage induction with mitomycin C was performed following a modified protocol described by Cao *et al.* [36]. 10 mL pre-warmed BHI was inoculated with a 1∶20 dilution of an overnight *S. aureus* culture in BHI and grown at 37°C, 200 rpm to late exponential phase. Mitomycin C was added to cultures at a concentration of 1 µg/mL, incubated 30 minutes, then resuspended in fresh, pre-warmed BHI with aeration to induce phage. Samples were then incubated 4 hours at 37°C, 200 rpm, centrifuged at 4,000 rpm at 4°C for 20 minutes and the supernatant filtered using 0.22 µm filters. 10 µl of filtered supernatant (from induced and uninduced cultures) was spotted on BHI soft-agar overlays (0.6% agar, 4 mM Ca^2+^) of *S. aureus* reporter strains NRS77, RN4220, and Newman (Table S1 in File S1), dried, and grown overnight at 37°C. Plates were inspected at 16 and 24 hrs for plaque formation. Supernatants were then precipitated in 10% polyethylene glycol (PEG)-8000 and 0.5 M NaCl following standard protocols and resuspended in SM buffer (100 mM NaCl, 8 mM MgSO_4_ • 7 H_2_O, 50 mM Tris-Cl (pH 7.5), 0.002% (w/v) gelatin). Prior to verification of bacteriophage DNA by PCR, bacteriophage samples were treated with 2 µl DNase (#2222, Applied Biosciences Ambion) at 37°C for 30 min. DNase in the bacteriophage samples was inactivated by incubation at 75°C for 10 min.

### Transmission electron microscopy of PEG-precipitated samples

PEG-precipitated phage samples as described above were visualized at The Rockefeller University Electron Microscopy Resource Center. Briefly, 10 µL of sample was spotted on glow discharged 200-mesh copper grids with carbon film (Electron Microscopy Sciences, Cat# CF200-Cu) and incubated for 2.5 min. Grids were then washed 2x in ddH_2_O, and stained with 2% 0.2 µm filtered uranyl acetate. Excess solution was wicked away and grids were allowed to dry at least 30 min before visualization. Grids were examined on the JEOL 100CX transmission electron microscope using AMT V600 software (Advanced Microscopy Techniques) and images captured at 33,000x and 50,000x magnification.

### PCR amplification of DNA

PCR amplicons for Southern analysis, primer walking for sequence gap closure, and for verification of the presence of free phage particles in culture supernatants were all generated using NRS19 extra-chromosomal DNA preparation as template. Two DNA polymerases were used: (1) Taq PCR Master Mix (#201443, QIAGEN) and (2) Phusion DNA Polymerase (F-530S, Thermo Scientific). The reaction condition for the Taq polymerase were as follows: (1) 95°C for two min; (2) 95°C for 30 sec; (3) appropriate annealing temperature for 30 sec; (4) 72°C for one min per kb; (5) repeat steps two through five 29 times; (6) 72°C for 10 min; and (7) 4°C indefinitely. The reaction conditions used for the Phusion polymerase were as follows: 1) 98°C for 30 sec; (2) 98°C for 10 sec; (3) appropriate annealing temperature for 30 sec; (4) 72°C for 30 sec per kb; (5) repeat steps two through five 29 times; (6) 72°C for 10 min; and (7) 4°C indefinitely. Following PCR, DNA was separated by electrophoresis at 100 V for 20 min on a 1.5 % agarose gel, run in 0.5 X TAE buffer. DNA was stained with EtBr, and visualized under ultraviolet transillumination on a Cell Biosciences (Santa Clara, CA USA) Alpha Image HP.

## Results

### Identification of ExPΦs in *Staphylococcus aureus*

**Figure 1:**
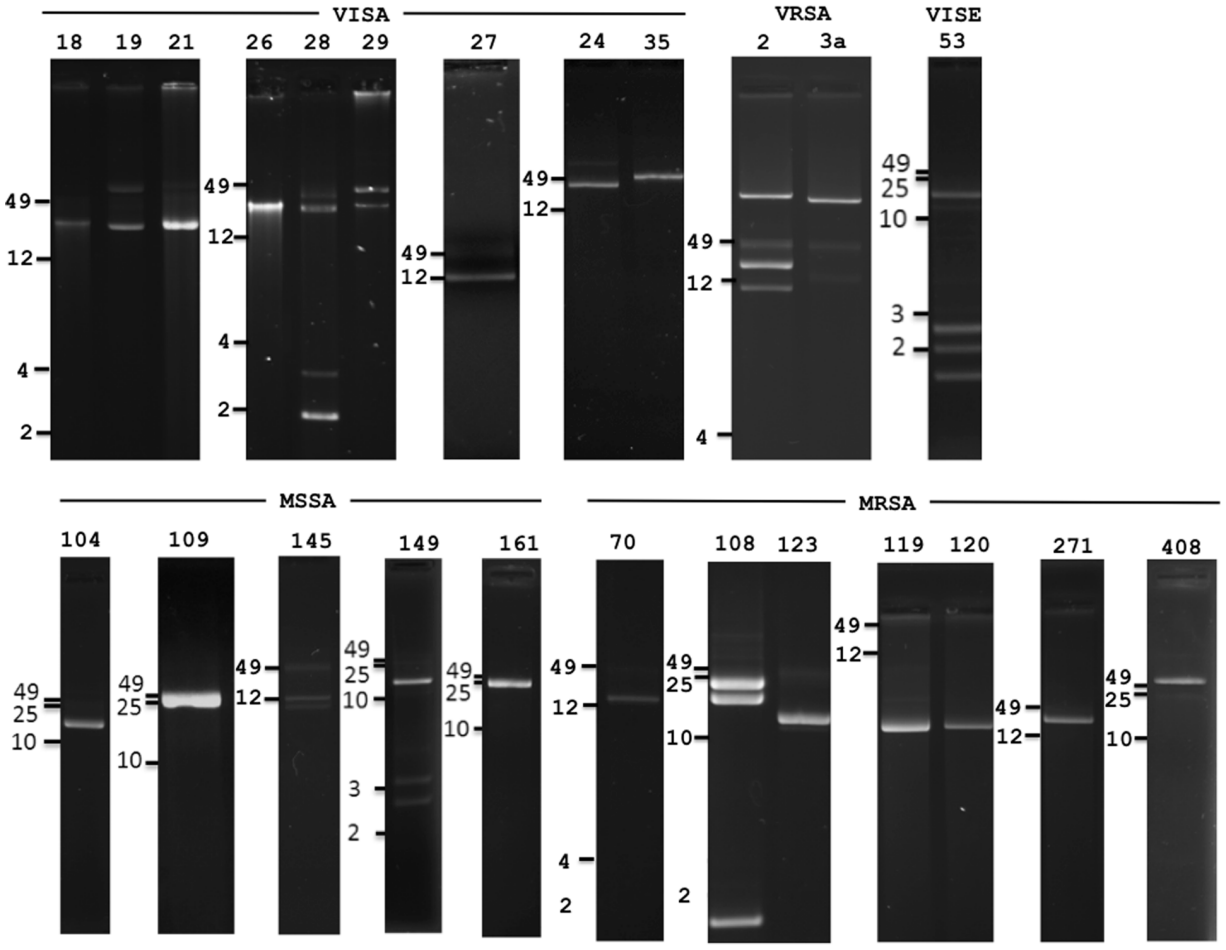
*S. aureus* extra-chromosomal DNA isolated from VISA, VRSA, MRSA, VISE and MSSA strains. Extra-chromosomal DNA from *S. aureus* strains was separated by electrophoresis on a 0.7% agarose gel and stained with ethidium bromide. Numbers on top designate NRS or VRS strain designations from the NARSA repository (http://www.narsa.net/content/staphLinks.jsp): NRS numbers 18, 19, 21, 24, 26, 27, 28, 29, and 35 are VISA strains; VRS 2 and 3a are VRSA strains; NRS 70, 108, 119, 120, 123, 271 and 408 are MRSA strains; 53 is a VISE; and 104, 109, 145, 149, and 161 are MSSA strains. Separate 0.7% agarose gels profiling the extra-chromosomal isolations were combined; numbers next to the black dashes on the left next indicate the position of molecular weight standards in kb.

**Figure 2:**
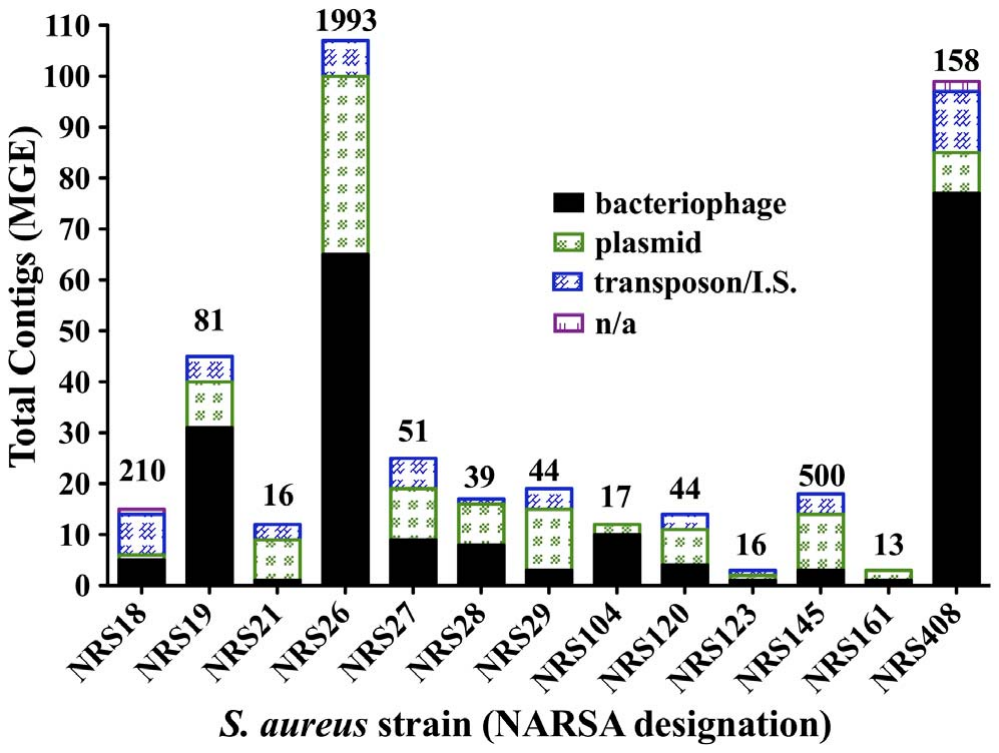
Identification and classification of contig sequences that contain phage genes and other genetic elements from *S. aureus* extra-chromosomal DNA. BLAST nucleotide sequence analysis identified the sequence identity of contigs generated from *S. aureus* extra-chromosomal DNA. Contigs were labeled based on homology to published nucleotide sequences: bacteriophage (black), plasmid (green), transposon/insertion sequences (IS) (blue), or not in GenBank database (n/a; purple). Contigs were labeled transposon/(IS) if a transposon or insertion element was unassociated or associated with plasmid or chromosome. Numbers above rectangular bars represent the total contigs within an individual sequencing sample. Y-axis lists total contigs identified as mobile genetic elements (MGE) based on BLAST homology identifications.

Extra-chromosomal DNA from 24 *Staphylococcus* NARSA strains (Table S1 in File S1) was isolated, and without further treatment, sequenced using Roche-454 technology with the resulting sequence assemblies screened for phage genes/genomes. Most extra-chromosomal DNA samples contained one or more bands ranging in size from 2 to >50-kilobasepairs (kb) when visualized on agarose gels (Figure 1). Many of these extra-chromosomal bands were within the range (15 to 44 kb) expected for *S. aureus* phage genomes [37]. Roche-454, Newbler assembler generated contigs (overlapping DNA segments that together represent a consensus region of DNA) of various sizes from 101 to 34,936 base pairs. Strikingly, BLASTN homology searches of these contigs revealed that ExPΦs were prevalent; seven of nine vancomycin-intermediate *S. aureus* (VISA), three of seven methicillin-resistant *S. aureus* (MRSA), and three of five methicillin-sensitive *S. aureus* (MSSA) extra-chromosomal DNA samples possessed phage genes (Figure 2 and Figure S1 in File S1). Phage DNA was not identified in the two vancomycin-resistant *S. aureus* (VRSA) strains (VRS2 and VRS3a) and the one vancomycin-intermediate *S. epidermidis* (VISE) strain (NRS53). Thus, Roche-454 based sequencing of *S. aureus* extra-chromosomal DNA revealed evidence of the possible widespread existence of ExPΦs for the first time in natural isolates of these human pathogens.

We analyzed the extra-chromosomal DNA from these *S. aureus* strains in more detail to categorize the MGEs found in our sequencing data. From BLASTN sequence analysis, all *S. aureus* extra-chromosomal contigs could be categorized as either plasmid, phage, or in some cases chromosomal DNA (Figure 2 and Figure S1 in File S1). If a contig was homologous to a transposon or insertion sequence, a notation was made. Phage genes that were identified within the contigs (File S2) are listed in Table S3 in File S1. Bioinformatic analysis of the sequences also yielded complete and nearly complete *de novo* plasmid assemblies. For example, sequencing the extra-chromosomal DNA of MRSA strain NRS108 yielded three complete plasmid sequences (accession number KF831355, KF831356, and KF831357; Figure S2 in File S1 and Table S4 in File S1).

### Sequencing extra-chromosomal DNA

Each extra-chromosomal DNA fraction was prepared as described in *Materials and Methods* and subjected to multiplexed shotgun sequencing using the Roche-454 Titanium protocol, followed by *de novo* assembly. The number of reads collected per sample varied from under 6,000 to over 71,000 (Table S5 in File S1). Similarly, the number of contigs resulting from *de novo* assembly varied, from 2 to 1993 per sample. The number of contigs was generally proportional to the Q39 score (the percentage of bases with a quality score of 39 and below), in that the fewer the number of contigs, the lower the score. Assemblies with large numbers of contigs, such as that of NRS26 (1993 contigs) and NRS145 (500 contigs), generally had higher Q39 scores (5.90% and 5.99%, respectively), possibly indicating that either the depth of coverage was relatively low, or the presence of more than one genomic species. This could occur with either low-level chromosomal contamination or the presence of multiple phages.

### Complete DNA sequence of an ExPΦ phage found in NRS19 (VISA)

**Figure 3:**
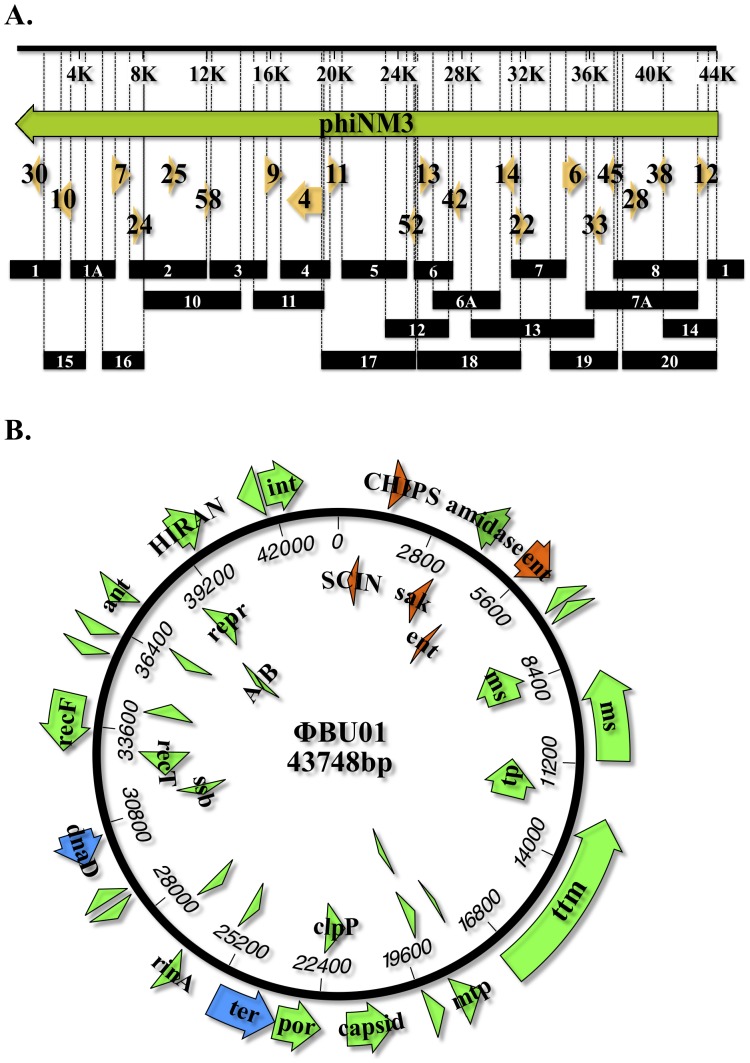
Strategy for genome sequencing of φBU01 from VISA strain NRS19 and completion of a genetic map resulting in a single circular contig. (A) Initial 454 sequencing of an unknown ExPФ in VISA strain NRS19 was assembled relative to *S. aureus* bacteriophage ФNM3 (NC_008617.1) template sequence. Alignment was generated using an algorithm with default settings in MacVector Version 12.6 [30], [31]. The preliminary alignments of 454 sequencing contigs (orange arrows, Table S3 in File S1, File S2) were used as a reference to generate PCR amplifications (black bars) to be used for primer walking and to determine unknown sequences. Numbers inside orange arrows designate specific contig numbers. Primers used to generate the ExPФ sequences (numbers within black bars designate PCR product) through primer walking (Sanger) are listed in Table S2 in File S1. (B) Physical map of VISA ΦBU01. Genes in orange indicate toxins encoded on the prophage, blue indicates genes associated with DNA replication and processing, and green indicates structural, miscellaneous, and hypothetical (blank arrows) genes. A list of the genes and descriptions are in Table S6 in File S1.

**Figure 4:**
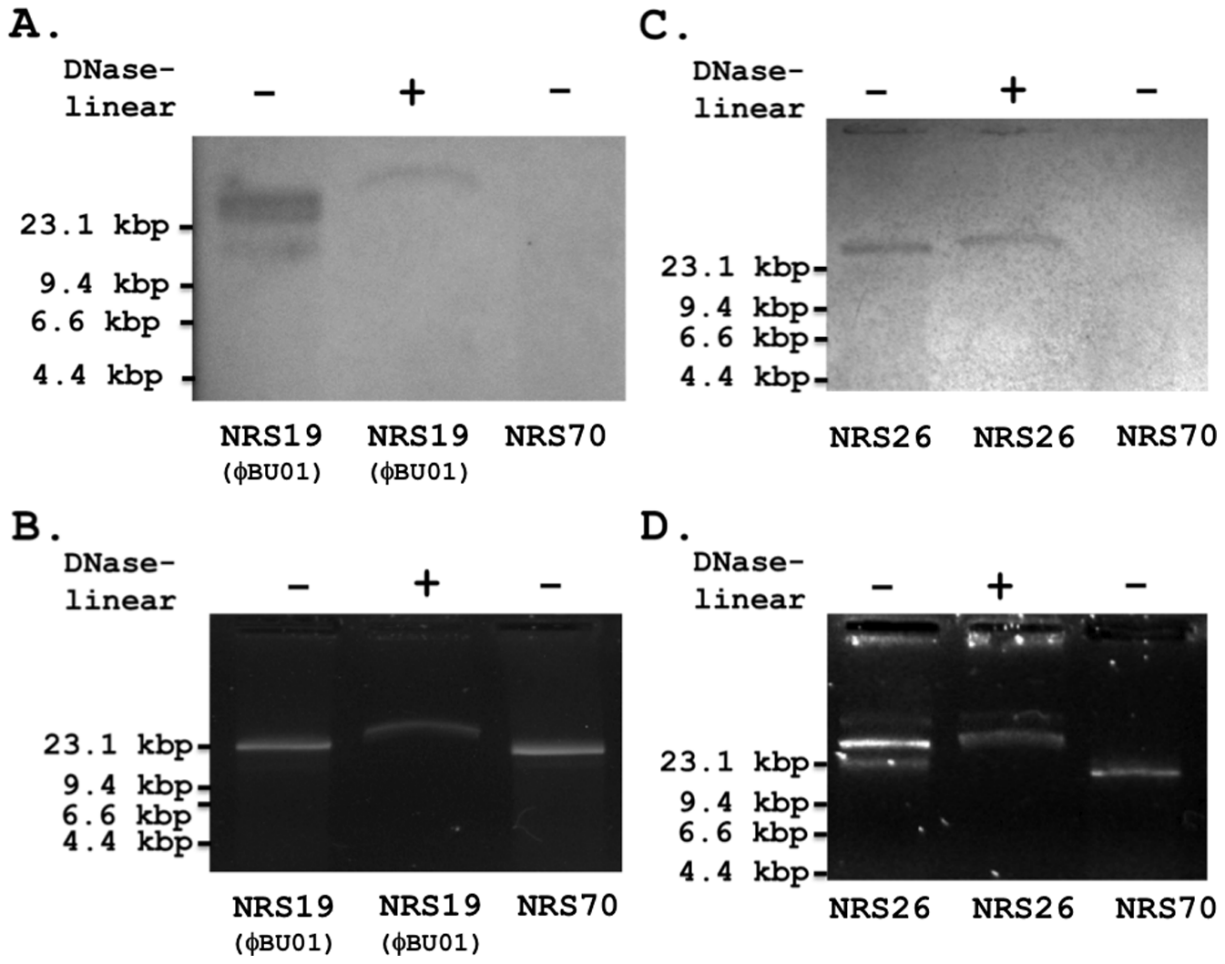
VISA extra-chromosomal DNA contains circular ExPФ's. Extra-chromosomal DNA of VISA strains NRS19 (ΦBU01, A and B) and NRS26 (C and D) was treated with or without Plasmid-Safe linear DNase treatment (DNase-linear, +/−), separated on a 0.7% agarose gel (B and D), and visualized with SYBR Safe DNA stain. DNA bands separated on the gel were transferred to a nylon membrane and analyzed by Southern blot using probes based on bacteriophage genes (A and C). Probes were generated by PCR amplification of genes located in bacteriophage phage DNA using primers sets bdu211/bdu212 for NRS19 (ΦBU01) and bdu215/bdu216 for NRS26 (Table S2 in File S1). MRSA strain NRS70 (70, A and C) was used as a negative control in the Southern blot.

The complete DNA sequence of the ExPΦ found in strain NRS19, ФBU01 (accession number KF831354), was determined by primer walking on PCR-amplified extra-chromosomal phage DNA (Figure 3 and Table S6 in File S1). Using a MacVector 12.6 PHRAP [30], [31] alignment algorithm (default settings), the contigs of the initial Roche-454 DNA sequencing were aligned to a reference genome sequence (NC_008617.1) of the *S. aureus* ФNM3 phage genome (Figure 3A). The predicted orientation of the contigs allowed for the design of primers for gap closure. These primers (Table S2 in File S1) were used to amplify the unknown regions of the prophage DNA and the PCR amplicons were sequenced, allowing for gap closure of the Roche-454 contigs. PCR amplification of all regions throughout the predicted ФBU01 genome, including the 5′and 3′ ends, provided additional evidence suggesting that the ExPΦ DNA was a closed circular unit (Figure 3A; PCR#1). ФBU01 was ultimately determined to be a 43.8 kb circular prophage with DNA sequence similarities to the *Siphoviridae* family of phages. The assembled circular sequence of ФBU01 and Southern blot identification of a Plasmid-Safe (a DNase specific for linear DNA) treated sample of NRS19 (Figure 4A and B) provided definitive evidence that the prophage exists as a closed circular extra-chromosomal element.

### Verification of ExPΦ DNA

The identified ExPΦs were either circular (e.g., the genome of coliphage P1) or linear (e.g., the genome of φAP50 of *Bacillus anthracis*) [5], [8]–[10], since prior to sequencing, the extra-chromosomal samples were not treated with Plasmid-Safe. While this step prevents the exclusion of linear phages (and other linear genetic elements), it may allow for increased chromosomal DNA contamination. The number of contigs varied among the different extra-chromosomal samples of *S. aureus*. (Figure 2, Figure S1 in File S1, and Table S5 in File S1), likely indicating some chromosomal contamination in samples with higher numbers of contigs. Southern blot analysis was used to determine if phage DNA identified by extra-chromosomal sequencing was in fact not integrated in the chromosome. VISA strain NRS19 was chosen for Southern blot analysis due to a high percentage of contigs that were homologous to phage DNA (Figure 2). VISA strain NRS26 was chosen to verify the robustness the technique in a “contaminated” sample, as it contained 65 contigs identified as phage DNA, and the highest number of chromosomal contigs of all sequenced strains.

Southern blot analysis revealed phage DNA in the extra-chromosomal fractions of both NRS19 (of which one was ΦBU01) and NRS26 (Figure 4A and C). These extra-chromosomal samples were pretreated with Plasmid-Safe, to digest all linear DNA prior to Southern blot analysis (Figure 4A and C). Interestingly, the NRS19 non-DNase treated sample contained at least three DNA bands that tested positive for phage DNA (Figure 4A), with only one band reactive after Plasmid-Safe treatment. However it is unclear at this time if one of these is the linear form of ExPΦ ФBU01 or a different phage altogether. These bands did not directly correspond to the prominent extra-chromosomal band in Figure 4B, suggesting these ExPΦs are present in very low-copy numbers in the bacterial population. Sequencing statistics reveals the majority of the DNA in the NRS19 sample is plasmid DNA. The NRS19 extra-chromosomal sample appears to contain at least one plasmid similar to *S. aureus* pGO1, which is predicted to be one of the extra-chromosomal bands visualized on the agarose gel.

The efficacy of the linear DNase is shown in a control study that removed sheared, contaminating chromosomal DNA from an extra-chromosomal DNA sample (Figure S3B in File S1). The extra-chromosomal bands containing phage DNA were observed in both linear DNase treated and untreated samples, verifying that the viral DNA exists extra-chromosomally in a closed-circular (DNase-resistant) conformation. NRS70 extra-chromosomal DNA was not reactive to the phage-specific probe and likely a plasmid (Figure 4A and C), and was therefore considered a negative control for ExPΦs. However, linear DNase treatment did not affect its DNA, indicating that it was in fact circular (Figure S3A in File S1).

**Figure 5:**
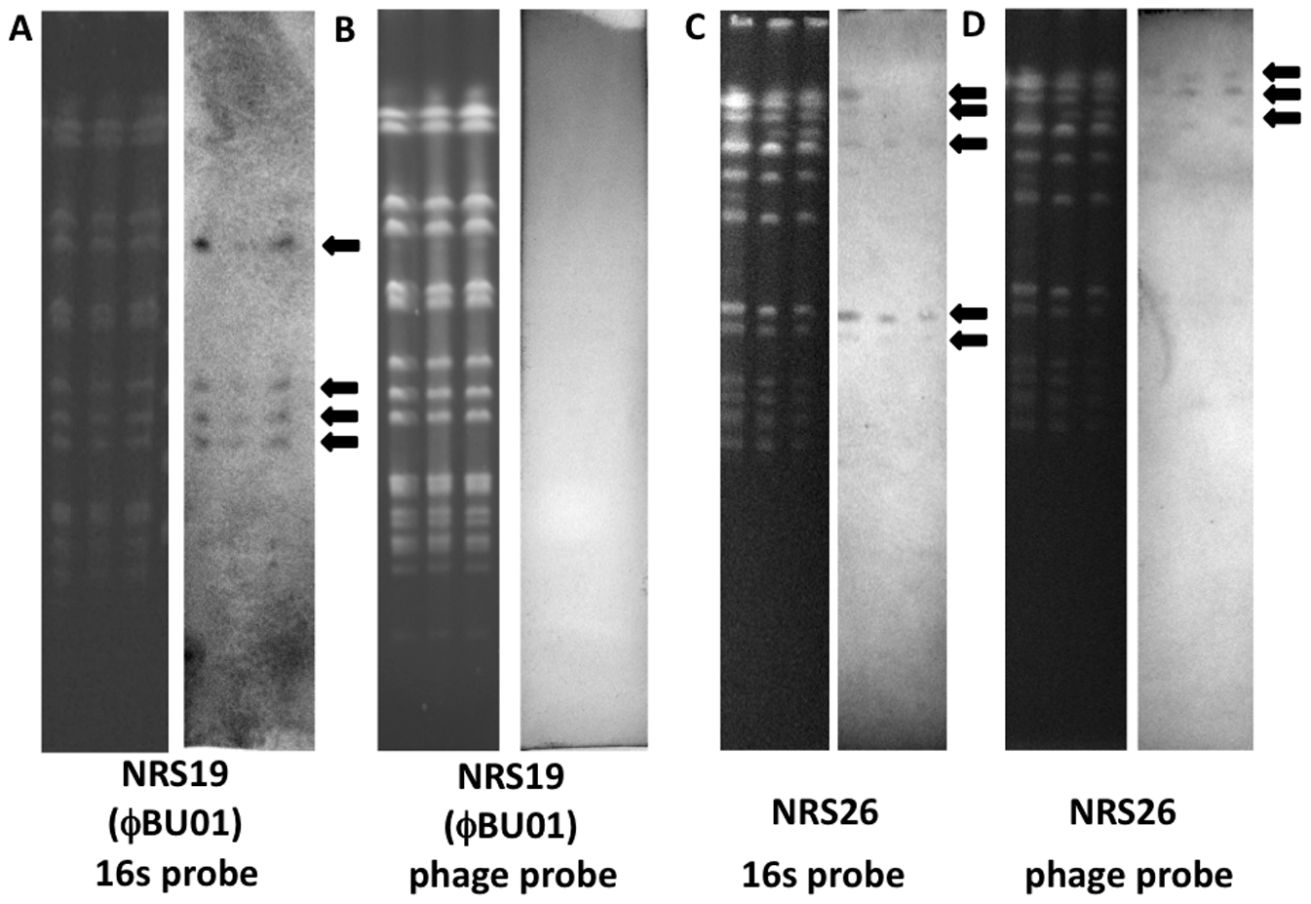
VISA ExPΦ's localize in and outside of the chromosome. *S. aureus* culture samples were taken at 6, 8 and 24 hours following 1∶100 dilution of overnight cultures. Samples were concentrated to an adjusted OD_600_ of 5.0 to 10. Chromosomal DNA was prepared inside 0.75% agarose beads and cut with *Sma*I restriction digestion enzyme. DNA bands separated by PFGE (left in each set) were transferred to a nylon membrane (right in each set) and analyzed by Southern blot. (A and C) Biotin incorporated labeled probe specific to 16S DNA of *S aureus* DNA encoding 16S ribosomal RNA. (B) Biotin labeled probe specific to an ExPΦ found in NRS19 (ΦBU01) was used for a Southern blot probe on NRS19 cultures. (D) Biotin labeled probe specific to an ExPΦ found in NRS26 was used for a Southern blot probe on NRS26 cultures. Black arrows indicate location of bands on the Southern blots.

While Southern blot analysis (Figure 4) of extra-chromosomal DNA verified that ExPΦ DNA was present, it remained unclear if this DNA existed entirely extra-chromosomally or if it could also integrate at times within the chromosome of *S. aureus*. To help address this, we analyzed the chromosomal DNA of strains NRS19 (ΦBU01), and NRS26 by pulsed-field gel electrophoresis (PFGE) (Figure 5). Primers bdu211 and bdu212 were designed from ExPΦ ΦBU01 found in NRS19 and primers bdu215 and bdu216 from an ExPΦ found in NRS26. Primers specific to 16S ribosomal RNA were used as a control to ascertain if chromosomal DNA was detected in NRS19 and NRS26 Southern blot transfers (Figure 5A and C). After 12 trials of which 6 were at different stages of bacterial growth, we were unable to detect ExPΦ DNA of ΦBU01 in the chromosome of NRS19 (Figure 5B and Figure S6 in File S1). However, ExPΦ DNA was detected in the chromosome of NRS26 (Figure 5D), suggesting that this phage may exist in both chromosomal and extra-chromosomal compartments in different cells within the population.

### *S. aureus* ExPΦs are inducible

To test whether ExPΦs are inducible, *S. aureus* strains NRS19 and NRS26 were treated with mitomycin C [36] and shown to produce plaques on select *S. aureus* reporter strains; controls without mitomycin C treatment were negative. Polyethylene glycol-precipitated phage preparations were first treated with DNase to remove any host chromosomal DNA not contained within the phage particles. Phage particles were then disrupted, and the released DNA was used as template in PCR. DNA extracted from mitomycin C-induced culture supernatants of NRS19 (which includes ΦBU01) and NRS26 was subjected to PCR analysis. Primers bdu211, bdu212 and bdu215, bdu216 described above were used to determine if ExPΦ DNA could be detected in the induced phage fraction. The primers specific for the ExPΦs of NRS19 and NRS26 detected the presence of phages from both strains (Figure S4 in File S1), verifying that ExPΦs of NRS19 and NRS26 could be packaged into phage particles. It remains unclear, however, whether the ExPΦs direct their own packaging, or if a helper phage is required.

### Enriched ExPΦs represent bona fide extra-chromosomal DNA elements

The ExPΦs that were enriched, sequenced, and identified in our study appear to represent stable extra-chromosomal DNA elements and are not cytoplasmically-located DNA elements from spontaneous excision/induction of stably integrated prophage. Clear evidence for this can be found in the sequencing run statistics of NRS19. Of 42,971 total sequencing reads from the extra-chromosomal sequencing of NRS19, 327 reads mapped onto ФBU01, representing 0.761% of the total sequencing reads. We calculated that if the presence of ФBU01 in the cytoplasmic compartment was solely due to spontaneous excision (assuming a spontaneous induction frequency of 10^−4^), then ФBU01 DNA should represent approximately 0.0078% of the extra-chromosomal DNA sample. Thus, ФBU01 is represented in our extra-chromosomal sample at a nearly 100-fold higher percentage (Table S7 in File S1), verifying that its presence in the extra-chromosomal compartment is attributed to events other than spontaneous excision.

In addition, some of the extra-chromosomal *S. aureus* samples sequenced did not contain any phage contigs; NRS24 (VISA) and NRS109 (MSSA) represent two such samples. Since these strains did not appear to contain ExPΦs, we were curious if they contained integrated prophage. Upon treatment with mitomycin C, we could induce phage from NRS24, which plaqued on *S. aureus* strain RN4220 (Figure S4A in File S1). While we did not detect plaques from the supernatant of induced NRS109, we did indeed confirm phage induction by transmission electron microscopy (TEM) (Figure S5A in File S1). The presence of phage was also confirmed by TEM in the supernatants of NRS24, NRS19 and NRS26 after treatment with mitomycin C (Figure S5B-D in File S1). Clearly, all four *S. aureus* strains can produce phage, yet in the absence of mitomycin C, extra-chromosomal sequencing only detected phage contigs in NRS19 and NRS26. If spontaneous excision events were solely responsible for the identification of phage elements in extra-chromosomal DNA samples, then we would expect to identify phage DNA in NRS24 and NRS109 (owing to spontaneous excision/induction of each strains' respective prophages). The lack of any extra-chromosomal phage contigs in the prophage-positive strains NRS24 and NRS109 indicates that the extra-chromosomal phage DNA of strains NRS19 and NRS26 are not the result of spontaneous excision but rather more stable, potentially plasmidial forms.

### Presence of virulence determinants in ExPΦ genomes

Sequence analysis of putative ExPΦ contigs from different *S. aureus* strains identified several putative, phage-encoded virulence factors with the potential to be horizontally transferred to other bacteria (Figure 3B, Table S3 in File S1, and Table S6 in File S1). The ExPΦs from strains NRS19, NRS26, and NRS408 all encoded the virulence-associated protein E gene first described in *Dichelobacter nodosus* and identified in pathogenicity islands of *S. aureus*. However, its mechanism of action has not been determined [38]. Additionally, staphylococcal enterotoxin type-I was found in NRS26 ExPΦ, while SCIN and SEP was encoded in NRS19 ExPΦ. SCIN is a staphylococcal complement inhibitor that binds to and inhibits C3 convertase activity on the bacterial surface [39], while SEP encodes a superantigen that can induce the T-cell proliferative response and cytokine production [40]. The complete sequence of ФBU01 from VISA strain NRS19 also revealed the presence of CHIPS, an exoprotein that inhibits neutrophil and monocyte chemotaxis [41], and SAK, which interacts with both α-defensins and plasminogen host proteins. SAK interaction with α-defensins inhibits their bactericidal activity, while binding of SAK to plasminogen produces plasmin, facilitating the invasion of S. aureus into surrounding tissues [42]. β-hemolysin-converting phages have been reported to contain 8-kb regions encoding SCIN, CHIPS, SAK and SEP genes, as part of an immune evasion cluster (IEC) [3]. Thus, ExPΦ ФBU01, encoding IEC-like genes, is one of many unidentified ExPΦ harboring virulence determinants that may play a major role in *S. aureus* virulence and in the horizontal spread of such genes among staphylococci.

## Discussion

Next generation sequencing has become a powerful tool to help decipher *S. aureus* genomes and extra-chromosomal elements [43], and can now screen entire bacterial chromosomes in a single sequencing reaction [44]. As such, the technology has allowed for genetic monitoring of staphylococcal outbreaks through genomic sequencing of MRSA and VRSA isolates [43], [45]. In addition, *S. aureus* MGEs have recently come into focus with 93 complete and 57 partial staphylococcal plasmids recently sequenced [46]. While these and other sequencing studies on *S. aureus* have helped accumulate an extensive amount of genomic information, they have yet to focus on the presence of plasmid-like phages and other ExPΦs in the cytoplasmic compartment of the cell [46]–[48]. Our work focused on NGS of extra-chromosomal DNA and identified a number of ExPΦs in natural isolates of *S. aureus* for the first time.

Southern blot analysis performed in this study (Figures 4 and 5) suggests that the ExPΦ of NRS19 (ΦBU01) exists primarily in the extra-chromosomal compartment, while that of NRS26 alternates between chromosomally-integrated and excised forms. Despite not detecting ФBU01 ExPΦ DNA in the chromosome of NRS19, we cannot definitively state that ФBU01 is a plasmid-like (solely extra-chromosomal) prophage element. Its genome includes an integrase, suggesting that it may integrate into the NRS19 chromosome at a specific growth phase or condition not tested in our Southern blots. Additionally, it is possible that ФBU01 may act as a plasmidial phage in NRS19, but is capable of integrating into the chromosome of other strains, similar to that of phages Φs80b and Φs84b with *S. aureus* strains s64c and 8325–4 [19].

However, an integrase does not always determine the fate of plasmidial and integrated bacteriophages. *Borrelia burgdorferi* strain B31 contains a plasmidial-prophage cp32-1 that encodes an integrase (AAF07426.1), and *Bacillus cereus* plamidial-prophage pE33L54 contains multiple integrases [13]. Additionally, linear plasmidal prophage, Vp58.5 (similar to plasmid prophages N15, PY54, and ΦKO2) of *Vibrio parahaemolyticus* is 92% identical to integrated VHML phage. The replication of linear plasmidial-phages requires a replication protein (RepA), an origin of replication (ori), a protelomerase (Tel), and a telomere resolution site. Both Vp58.5 and VHML phage contain these elements. Therefore the presence of single genes such as integrases, is not a reliable indicator of whether a bacteriophage can integrate or is solely excised [49]. Whether ΦBU01 is an episome or plasmid-like element of NRS19 is currently under investigation, but nonetheless, the prophage clearly exists as an ExPΦ of *S. aureus*.

Extra-chromosomal DNA from nine VISA, seven MRSA, two VRSA, one VISE and five virulent MSSA strains were isolated and screened for ExPΦs using Roche-454 sequencing (Table S1 in File S1). Sufficient extra-chromosomal DNA was obtained for sequencing by effectively lysing mid-exponential cells from 150 mL of culture using the PlySs2 lysin [26] combined with lysostaphin and lysozyme. The use of three cell wall hydrolases facilitated controlled gentle lysis of the staphylococcal cells without the necessity of mechanical force, thus reducing chromosomal shearing. As a result, chromosomal contamination in the extra-chromosomal fraction was minimized, allowing for increased depth of coverage of lower-copy genetic elements. Additionally, during the preparation of DNA samples, rotational force during centrifugation was kept less than 5000×*g* to reduce potential shearing of large DNA elements. Furthermore, Roche-454 sequencing was selected as it generated DNA sequencing read lengths longer than other NGS platforms [50], allowing *de novo* assembly of contig sequences of low-copy DNA elements.

Our study both enriched for non-genomic cytoplasmic DNA and screened specifically for potential ExPΦs. While we identified ExPΦs in the majority of MRSA, VISA, and MSSA strains, it is unknown whether our inability to identify these phage elements in some strains is due to their absence or very low-copy number. Directing our attention toward extra-chromosomal elements in *S. aureus* also allowed for complete, *de novo* assembly of multiple plasmids found within the same *S. aureus* strain (Figure S2 in File S1, Table S4 in File S1). Thus, our sample preparation and sequencing approach revealed both novel plasmidial and other low-copy elements in bacterial cytoplasm that may be missed during full genomic sequencing.

ФBU01 shares sequence homology to several phages, including *S. aureus* bacteriophage ФNM3 (GenBank: DQ530361.1) containing a region of 98% percent identity with a query coverage of 83%. ФNM3 is a defective β-haemolysin (*hlb*) converting phage essential to the virulence of *S. aureus* Newman strain [41], [51]. The homologous genes of ФBU01 and ФNM3 include the immune evasion cluster genes (*sea, sak, chp*, and *scn*), the CHIPS gene responsible for reduction in neutrophil recruitment, phage structural genes, and an integrase gene (tyrosine recombinase family XerC). ФNM3 is unique in that it does not encode an excisionase, and primarily remains integrated within the chromosome [51]. While ФBU01 is primarily extra-chromosomal, it also does not appear to contain a known excisionase (Figure 3B and Table S6 in File S1). ФBU01 homology differs from ФNM3 primarily in nucleotides 37,344 to 40,547. This region contains the repressor, anti-repressor, and HIRAN DNA-binding protein of ФBU01. A second striking difference can be seen in the replication protein of the two phages. The first 150 amino acids of the replication proteins differ between ФNM3 and ФBU01, while the second 150 amino acids are highly conserved. The differences in repressor and replication proteins may explain why ФNM3 is primarily an integrated phage, whereas ФBU01 exists as an extra-chromosomal phage. Future studies will focus on host and phage encoded factors that cause prophages to remain integrated or excised.

Extra-chromosomal isolations separated by electrophoresis contained multiple bands (Figures 1 and 4) in DNA agarose gels and Southern blots. While this study focused primarily on one ExPΦ from VISA NRS19 (ФBU01) and one from VISA NRS26, the presence of multiple DNA elements in these strains strongly suggests that they harbor additional ExPΦs. In addition, the multiple DNA bands seen in agarose gels suggest an array of other genetic elements, the nature of which are currently under investigation. Potential *S. aureus* MGEs that may be identified in this analysis include phages, plasmids, transposons, *S. aureus* pathogenicity islands (SaPIs), and staphylococcal cassette chromosomes (SCCs), all of which may be transferred horizontally between staphylococci [52]. Thus, sequencing extra-chromosomal DNA may provide a new approach to study the movement of virulence determinants and antibiotic resistance genes via MGEs.

The rates of excision of episomal phages vary depending on both phage and bacterial host. For example, phage φ12 in host NCTC8325 has an excision rate of 10^−4^, while φ11 in the same host has an excision rate closer to 10^−2^. In the same study, ΦSA2mw had excision rates over 10^−2^ in NCTC8325, yet under 10^−3^ in host strain MW2 [20]. Excision rates of 10^−4^ are within the label of “spontaneous excision”, with a range of 10^−4^ to 10^−5^. However, rates of 10^−2^ represent a two-log increase and potentially occur in 1% of the population. Based on our calculations, we predict that at a minimum, the episomal phages identified by our technique would excise at a rate of 10^−2^ or greater (Table S7 in File S1). Episomal phages with increased excision rates can have more effects on cell phenotype, host genome interactions, and increased HGT. The techniques outlined in this paper would therefore be ideal for screening and identifying important low-copy MGEs, as well as elements with higher excision rates.

Integrated phages can be vertically transferred from mother to daughter cells, passing on the phage's genetic information, associated virulence factors, and transcriptional regulators that can have important effects on the bacterial cell. The extra-chromosomal form provides multiple DNA copies of the phage, yet its relevance in vertical and HGT is currently overlooked. While the rate at which ExPΦs are transferred is unclear, it is possible that they could mediate their own transfer or be transferred by helper phages or other elements. Transduction by phage plays a pivotal role in *S. aureus* HGT both during colonization and infection of humans [53]–[55]. Results of this study show that ExPΦs of NRS19 and NRS26 were inducible and thus infective (Figure S4 in File S1). HGT in *S. aureus* is largely dependent upon phage transduction [54]; SaPIs are capable of utilizing phage machinery to transfer genetic information from donor to recipient cells [56], and transduction can also transfer plasmids and chromosomal markers [57]. *S. aureus* phages 80α and 29 are capable of transducing SCC*mec* typeIV and SCC*mec* type I MGEs (20 to 25 kb) into *S. aureus* recipient strains [58]. Our study also detected SCC*mec* elements in the extra-chromosomal sequencing results. Therefore, it is plausible that MGEs detected in this study, including ExPΦs, could be transferred by phage transduction.

Further studies on the replication of ExPΦs in *S. aureus* are needed before categorizing these genetic elements as true plasmidial prophages. The mechanism for the replication of ΦBU01 is presently unclear, however, a putative DnaD replication protein is encoded on ΦBU01. DnaD replication proteins have been found in *S. aureus* phages [59], although little is known about their role in phage DNA replication. DnaD however, is essential for replication in *B. subtilis* [60], and yeast two-hybrid studies showed DnaD recruited to the membrane fraction of the cell [61]. While very little is known about the putative open reading frames of *S. aureus* phage DNA replication modules [57], the role of DnaD in DNA initiation and membrane recruitment might be essential for ExPΦs like ΦBU01 to replicate and/or partition for appropriate dissemination to daughter cells. Further studies are needed to determine if DnaD and other unknown genes of the ΦBU01 genome are essential for phage DNA replication and partitioning.

In conclusion, it is clear that advances in NGS have allowed for cost effective whole genomic sequencing of a number of organisms, leading to the sequencing of more 10,000 bacterial genomes and several thousand phage genomes to date [62]. However, despite this wealth of data, current genomic sequencing approaches for bacteria may miss the vast genetic diversity seen in the extra-chromosomal compartment. Our study addresses this major gap. The unique approach of enriching for extra-chromosomal DNA coupled with the high throughput capability of Roche-454 sequencing to sequence these DNA elements has revealed new information from a cellular compartment often overlooked in traditional chromosome-centric sequencing. We show for the first time the potential widespread existence of ExPΦs in antibiotic resistant and virulent strains of *S. aureus*. These elements may play a major role in bacterial virulence and human disease, as they encode multiple *S. aureus* virulence determinants. Perhaps their presence as multiple ExPΦ is beneficial by allowing for increases in gene dosage as a result of accumulative higher expression levels. Importantly, ExPΦs could potentially facilitate the dissemination of these virulence factors through transduction or in tandem with another MGE. Understanding the biology of these elements and their contribution to pathogenesis will pave the way for better epidemiology, diagnostics, and therapeutics for *S. aureus*. Furthermore, the approach described here can also easily be extended to reveal the cytoplasmic DNA elements of other bacterial pathogens.

## Supporting Information

File S1 **Supplementary Figures and Tables.**
(DOCX)

File S2 **Preliminary alignments of 454 sequencing contigs identified as bacteriophage DNA.**
(DOCX)
