# Supplementary material for: Beyond the Chromosome: The Prevalence of Unique Extra-Chromosomal Bacteriophages with Integrated Virulence Genes in Pathogenic Staphylococcus aureus
Source: PLoS One. 2014 Jun 25;9(6):e100502. doi: 10.1371/journal.pone.0100502 (PMC4070920; doi:10.1371/journal.pone.0100502)
Supplement: File S1 — Supplementary Figures and Tables. (DOCX) [file pone.0100502.s001.docx]

**File S1: Supplementary Figures and Tables**

**Figure S1: Identification and classification of all contig sequences generated from *S. aureus* extra-chromosomal DNA.**

BLAST nucleotide sequence analysis identified DNA sequence of the contigs generated from *S. aureus* extra-chromosomal DNA. Contigs were grouped based on homology to published nucleotide sequences: bacteriophage (black), plasmid (green), transposon/IS (blue), or not in GenBank database (n/a; purple). Contigs were labeled transposon/insertion sequence if transposon or insertion element was associated with plasmid, chromosome, or unassociated. The total number of DNA sequencing contigs from an individual sequencing sample is represented by the number above each bar. Y-axis lists total contigs identified as mobile genetic elements (MGE) based on homology identifications.****

**Figure S2: Physical and genetic maps of three *S. aureus* plasmids found in NRS108.**

NRS108 extra-chromosomal DNA was sequenced using Roche-454 sequencing and assembled using the Roche gsAssembler Newbler under default settings, resulting in plasmids pBU108a, pBU108b, and pBU108c. Putative open reading frames and orientations are shown. Yellow indicates genes associated with antibiotic resistance, orange indicating virulence-associated genes, blue indicating DNA replication and processing, and green indicating miscellaneous and hypothetical (blank arrows) genes. Final assemblies were submitted to Genbank with the following accession numbers: pBU108a, KF831355; pBU108b, KF831356; and pBU108c, KF831357. Genes are listed with descriptions on Table S5 in File S1.

**A.** **B.**

**Figure S3: Linear DNase treatment of *S. aureus* NRS70 and *Bacillus cereus* 1399 extra-chromosomal DNA.**

Extra-chromosomal DNA of NRS70 (A) and 1399 (B) was treated with or without Plasmid-Safe^®^ linear DNase (Linear DNase, +/-). 1399 (B) was also treated with or without RNaseA (RNaseA, +/-). Treated and untreated extra-chromosomal samples were separated on a 0.7% agarose gel. DNA separated on the agarose gel was visualized with SYBR^®^ Safe DNA stain.


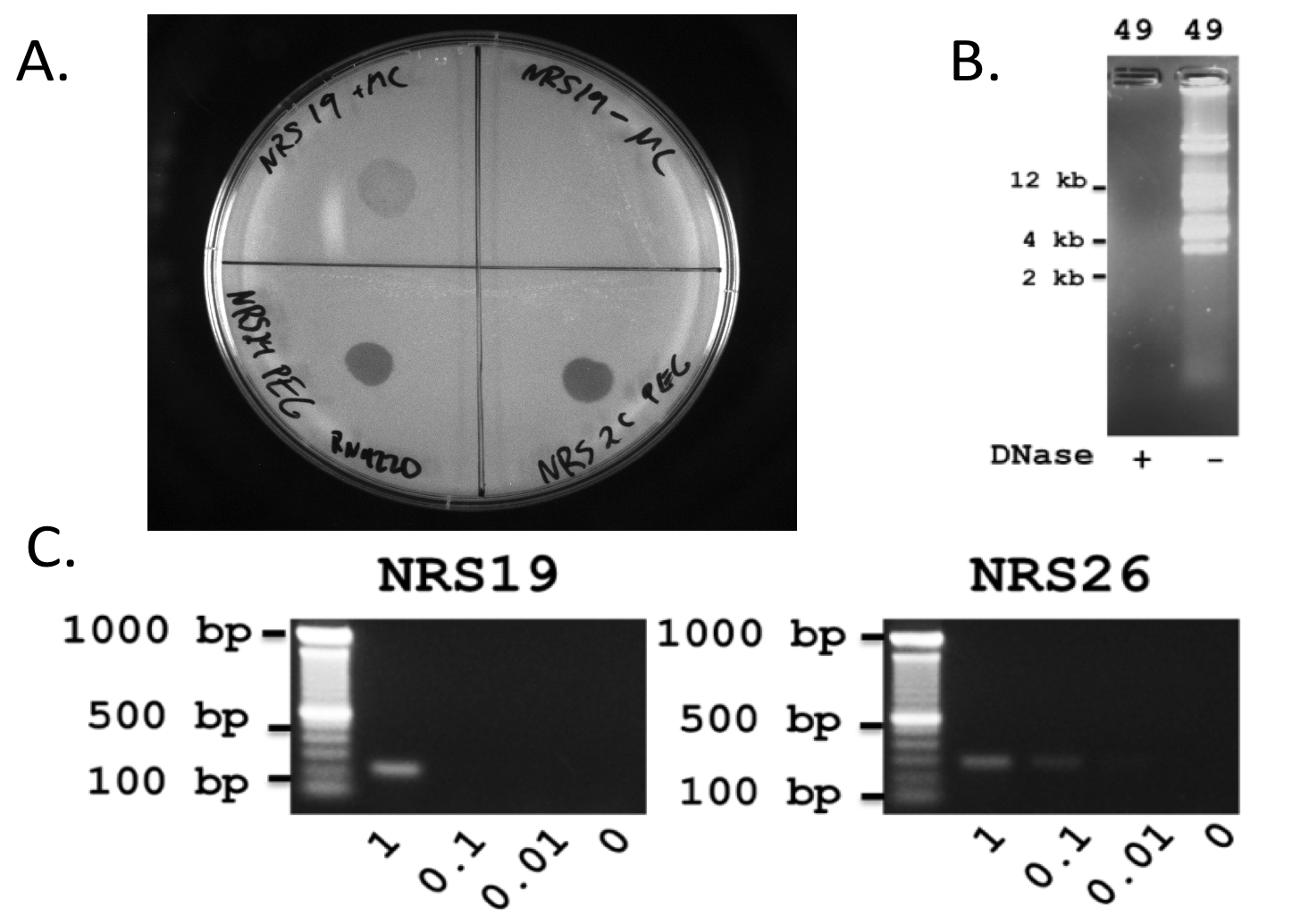

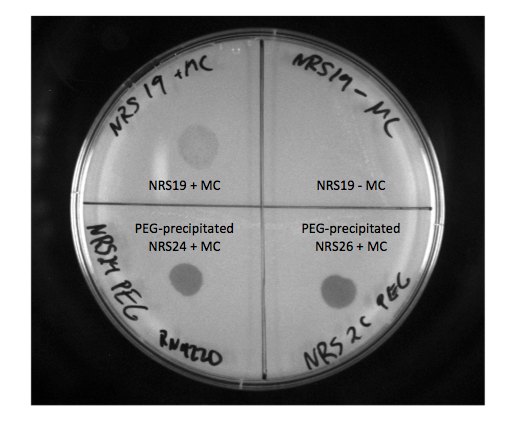


NRS19 (ϕBU01)

NRS26

**Figure S4: NRS19, NRS24, and NRS26 produce phage upon mitomycin C induction and ExPΦs of NRS19 and NRS26 are detected in inducible bacteriophages.**

Late exponential cultures of NRS19 (includes ΦBU01), NRS24, and NRS26 were treated with 1 µg/mL mitomycin C to induce phage or were left untreated. (A) Upper half of plate: After filtration, induced (white arrow) and uninduced (black arrow) NRS19 culture supernatants were tested for the ability to form a zone of clearing on a *S. aureus* RN4220 reporter strain. Induced NRS19 culture supernatant showed a zone of clearing on the soft-agar overlay, while uninduced NRS19 culture supernatant did not show any clearing. Bottom half of plate: PEG-precipitated induced culture supernatants of NRS24 (left) and NRS26 (right) were also spotted on RN4220, showing zones of clearing. (B) DNase treatment on *Bacillus cereus* strain 49 verified DNA was degraded following treatment. (C) Induced phage was precipitated with PEG and DNase treated. Potential, precipitated phages of NRS19 and NRS26 were tested with PCR using phage specific primers (bdu211/bdu212 and bdu215/bdu216, respectively).


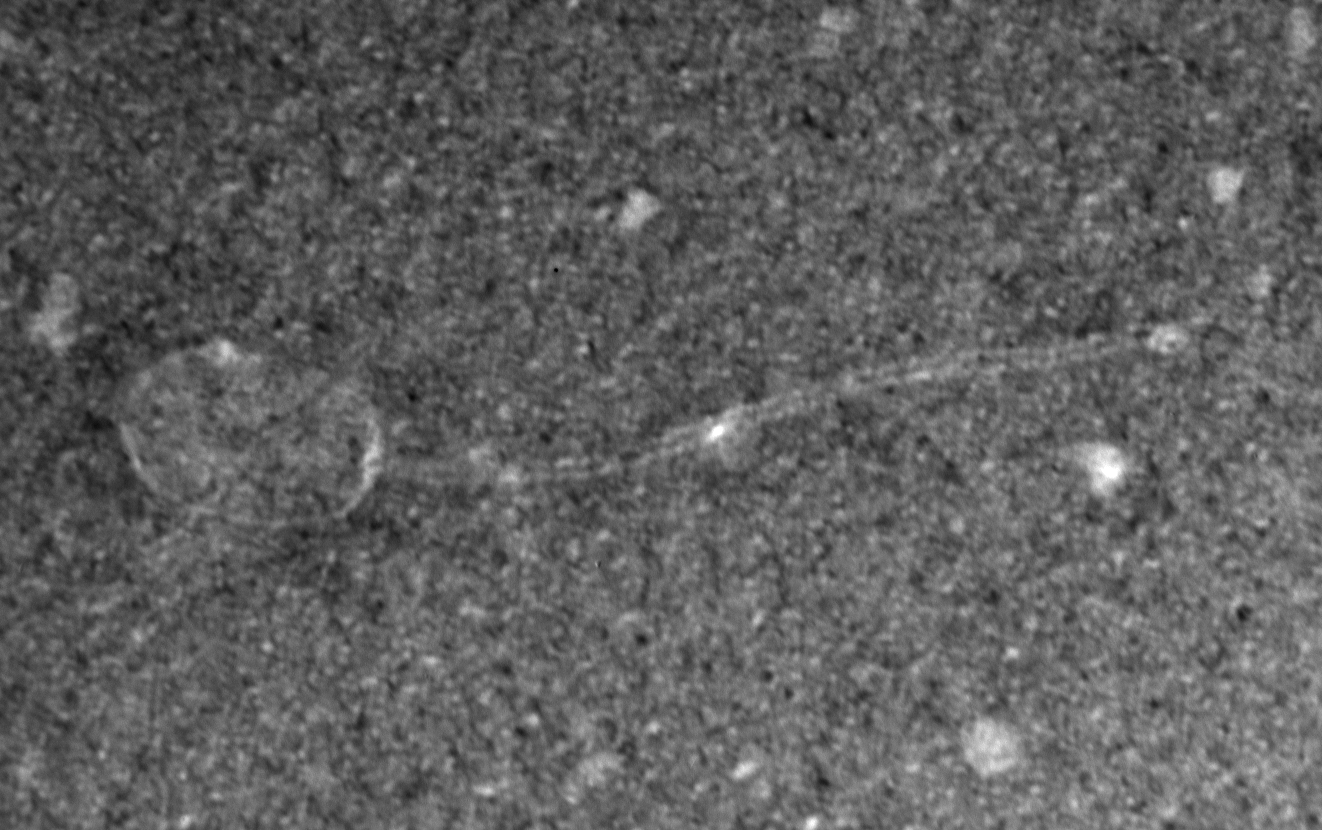

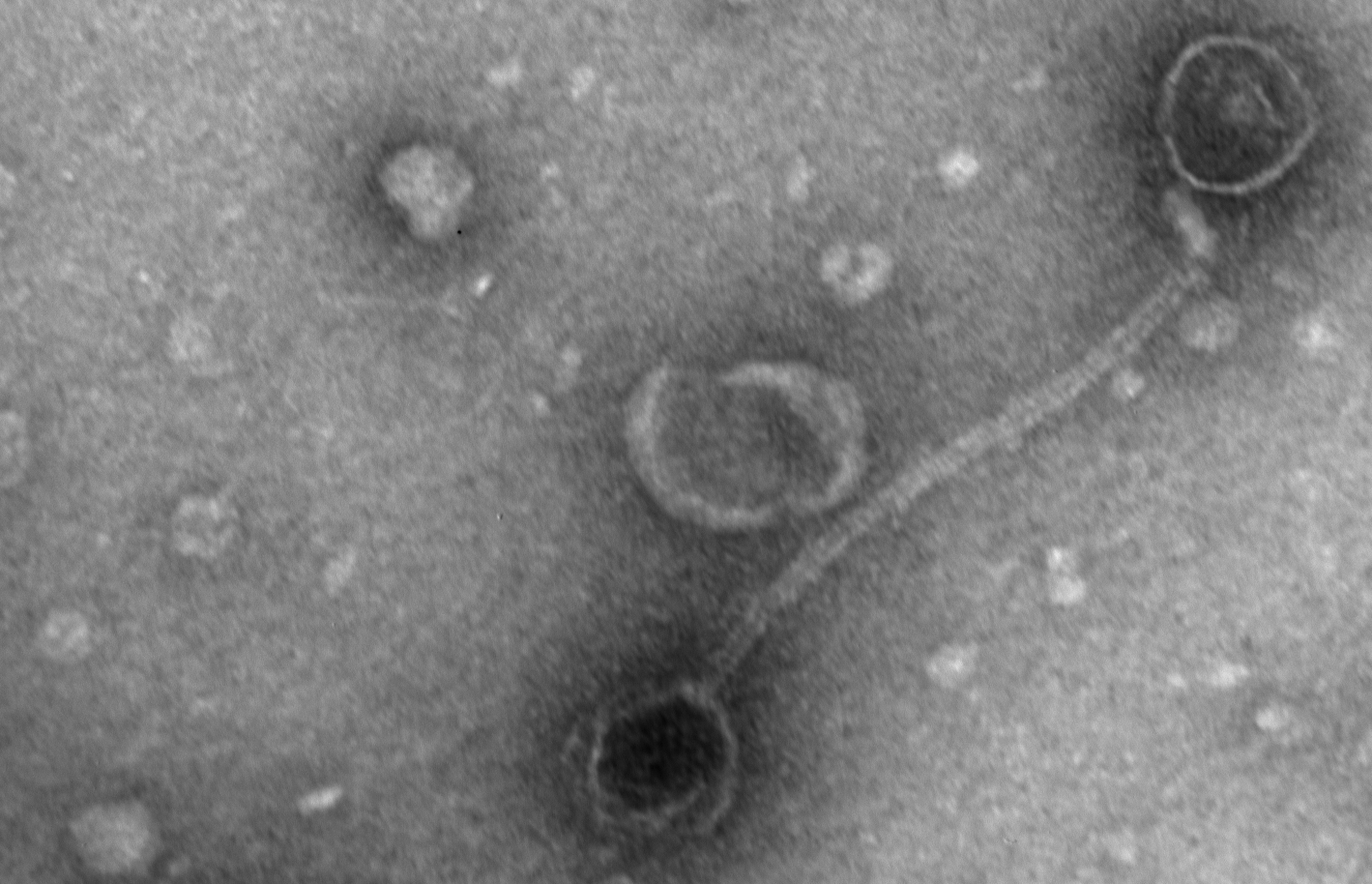

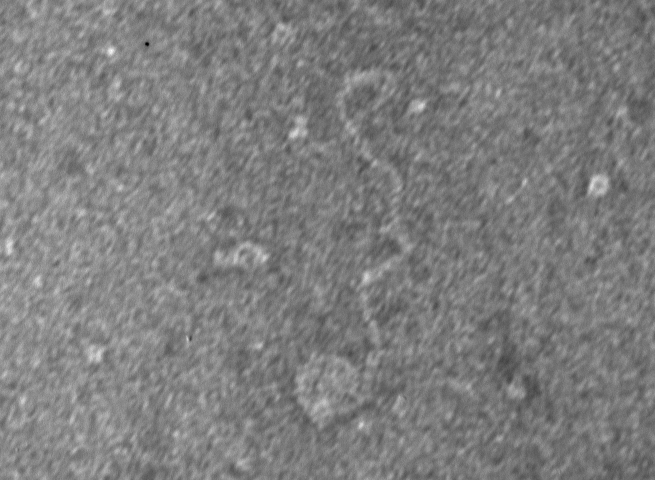

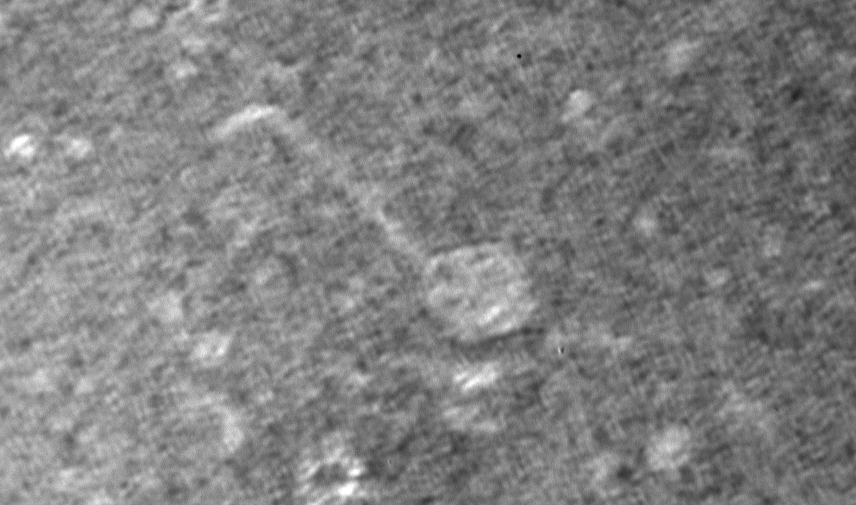


**A**

**B**

**C**

**D**

F**igure S5: Transmission electron microscopy (TEM) of induced phage from NRS109, NRS24, NRS19, and NRS26.**

Mitomycin C-induced cultures were PEG precipitated and visualized with TEM. A) Phage from NRS109 , B) Phage from NRS24, C) Phage from NRS19 (includes ΦBU01) and D) Phage from NRS26. Panels A, B, and C are at 50,000x magnification, D at 33,000x.

**Figure S6: VISA ExPΦ φBU01 localizes outside of the chromosome throughout the growth cycle.**

*S. aureus* culture samples of NRS19 (containing φBU01) were taken at an OD_600_ of 0.12, 0.65, 1.3, 2.0 and 2.5 (24 hrs). Samples were concentrated to an adjusted OD_600_ of 5.0 to 10. Chromosomal DNA was prepared inside 0.75 % agarose beads and cut with *Sma*I restriction digestion enzyme. DNA bands separated by PFGE (A and C) were transferred to a nylon membrane (right side of each set) and analyzed by Southern blot. A biotin labeled probe specific to an ExPΦ found in NRS19 was used for Southern a blot probe on NRS19 cultures (B). A biotin incorporated labeled probe specific to 16S DNA of *S aureus* DNA encoding 16S ribosomal RNA (D). Black arrows indicate location of bands on Southern blot. L designates size ladder.

**Table S1: List of strains and plasmids used in this study.**

| **NARSA Strain** | **Strain** | **Strain Type** | **Description** | **Geographic Origin** | **Year** | **Reference** |
| --- | --- | --- | --- | --- | --- | --- |
| ***Staphylococcus aureus*** | | |  |  |  |  |
| VRS2 | HIP11983 | VRSA | *mecA(+)* and *vanA(+)* | Pennsylvania (US) | 2002 | [[1](#_ENREF_1)] |
| VRS3a | HIP13170 | VRSA | *mecA(+)* and *vanA(+)* | New York (US) | 2004 | [[2](#_ENREF_2)] |
| NRS18 | HIP06854 | VISA | *mecA(+)* | New Jersey (US) | 1998 | [[3](#_ENREF_3)] |
| NRS19 | HIP07256 | VISA | *mecA(+)* | Illinois (US) | 1999 | [[4](#_ENREF_4)] |
| NRS21 | HIP07920 | VISA | *mecA(+)* | Rhode Island (US) | 1998 | [[4](#_ENREF_4)] |
| NRS24 | HIP09143 | VISA | *mecA(+)* | Ohio, US | 2000 | [[4](#_ENREF_4)] |
| NRS26 | HIP09313 | VISA | *mecA(+)* | Texas (US) | 2000 | [[4](#_ENREF_4)] |
| NRS27 | HIP09433 | VISA | *mecA(+)* | Michigan (US) | 2000 | [[4](#_ENREF_4)] |
| NRS28 | HIP09662 | VISA | *mecA(+)* | West Virginia (US) | 2000 | [[4](#_ENREF_4)] |
| NRS29 | HIP09735 | VISA | *mecA(+)* | North Carolina (US) | 2000 | [[4](#_ENREF_4)] |
| NRS35 | LIM-1 | VISA | *mecA(+)* | France | 1995 | [[5](#_ENREF_5)] |
| NRS53 | N/A | VISE | *mecA(+)* | Pennsylvania (US) | 2001 | NARSA |
| NRS70 | N315 | MRSA | *mecA(+)* | Japan | 1982 | [[6](#_ENREF_6)] |
| NRS104 | Cowan I | MSSA | high producer of protein A | US | 1935 | NARSA |
| NRS108 | A960649 | MRSA | *mecA(+)* | France | 1999 | [[7](#_ENREF_7)] |
| NRS109 | FRI361 | MSSA | *Sec* | n/a | 1989 | [[8](#_ENREF_8)] |
| NRS119 | SA LinR #12 | MRSA | *mecA(+)* | Massachusetts (US) | 2001 | [[9](#_ENREF_9)] |
| NRS120 | SA LinR #13 | MRSA | *mecA(+)* | Massachusetts (US) | 2001 | [[9](#_ENREF_9)] |
| NRS123 | MW2 | MRSA | *mecA(+)* | North Dakota (US) | 1998 | [[10](#_ENREF_10),[11](#_ENREF_11)] |
| NRS144 | RN4220 | MSSA | research strain | n/a | 1983 | [[12](#_ENREF_12)] |
| NRS145 | RN4282 | MSSA | TSST-1 arg group I | Minnesota (US) | n/a | [[12](#_ENREF_12),[13](#_ENREF_13)] |
| NRS149 | 502A | MSSA | arg group II | n/a | 1971 | [[14](#_ENREF_14)] |
| NRS161 | 2000 0509 | MSSA | *PVL(+)* | France | n/a | NARSA |
| NRS271 | n/a | MRSA | *mecA(+)* | United Kingdom | 2002 | [[15](#_ENREF_15)] |
| NRS408 | HIP11713 | MRSA | *mecA(+)* | Michigan (US) | n/a | [[16](#_ENREF_16)] |
| n/a | Newman | MSSA | research strain | n/a | 1952 | [[17](#_ENREF_17)] |
|  |  |  |  |  |  |  |
| ***Bacillus species*** | |  |  |  |  |  |
| n/a | 1414 | n/a | n/a | n/a | n/a | laboratory strain |
| n/a | 1399 | n/a | n/a | n/a | n/a | laboratory strain |
|  |  |  |  |  |  |  |
| **NRS Plasmid source** | **Plasmid Strain** | **Plasmid name** | **Description** | **Geographic Origin** | **Year** | **Reference** |
| NRS19 | HIP07256 | ΦBU01 | ExPΦ | Illinois (US) | 1999 | this study |
| NRS108 | A960649 | pBU108a | T4SS*, ssaA* | France | 1999 | this study |
| NRS108 | A960649 | pBU108b | *blaZ, entP,entJ, entR, cadD* | France | 1999 | this study |
| NRS108 | A960649 | pBU108c | *ermC* | France | 1999 | this study |

**Table S2: List of primers used for sequencing and Southern blot in this study.**

| **Sequencing/Southern Description** | **Template PCR** | **Primer** | **Forward Sequence 5’→3’** | **PCR product generated** |
| --- | --- | --- | --- | --- |
|  |  |  |  |  |
| NRS19fwd | n/a | bdu211 | CCTGTTGCTTGGGTAACTGTATC |  |
| NRS19rev | n/a | bdu212 | AATGGCAGAAAGTGGCTGG |  |
| NRS26fwd | n/a | bdu215 | TGCCATTGTGATGAGGAGGG |  |
| NRS26rev | n/a | bdu216 | GCAACGCAGATTGTTTGAGTG |  |
| Fwd@contig12 | 1 | bdu221 | GCAATTAGCTATGATGGTTACAT | 1 |
| Rev@contig30 | 1 | bdu222 | GGTAAATAACAATACACACCCTTC |  |
| Rev@contig22 | 7 | bdu224 | CGTTGACGTTACGCAAACTGACG | 7 |
| Fwd@contig6 | 7A | bdu225 | CTGTCTGGATTGATTTCATGTATCTG | 7A |
| Rev@contig10 | 1 | bdu228 | CGTTGTAGGAAGACCACTATTTT | 1 |
| Fwd@contig10 | 1A | bdu229 | TATACTACGGGGGAGTAGTATG | 1A |
| Rev@contig7 | 1A | bdu230 | ACAAAATACAGTACCTTTGG | 1A |
| Fwd@contig7 | 2 | bdu231 | CGACCATACAAATAACATG | 2 |
| Fwd@contig25 | 2 | bdu233 | TGTTGCTATTATTTCCAACGC |  |
| Rev@contig58 | 2 | bdu234 | TCATACAACCTGAAAACGTC | 2 |
| Fwd@contig58 | 3 | bdu235 | CTATCCACTCTTTCTCATCC | 3 |
| Rev@contig9 | 3 | bdu236 | GGCACTGTGCAGTTAATTTGG | 3 |
| Fwd@contig9 | 4 | bdu237 | CAGGACCTGCTTTTGCACC | 4 |
| Fwd@contig42 | 4 | bdu239 | CGCCTTTTTCACCGTCTCCA |  |
| Rev@contig11 | 4 | bdu240 | CGATATCGACGACCCAATACC | 4 |
| Fwd@contig11 | 5 | bdu241 | CTGGACCATTATTTTCAGTATAC | 5 |
| Rev@contig52 | 5 | bdu242 | GGGCTTGAAAAAGTCATAGC | 5 |
| Fwd@contig52 | 6 | bdu243 | CTTCAGGCAAATTCATTCGC | 6 |
| Fwd@contig13 | 6 | bdu245 | GTCTTCCCACATGTAATCAA | 6A |
| Rev@contig42 | 6 | bdu246 | GCGAATGAATAACGATATTAAAAAG | 12 |
| Rev@contig14 | 6A | bdu248 | GAGGGATTACATGGAGATAG | 6A |
| Fwd@contig14 | 7 | bdu249 | GCATATCCCCAAGTAACTT | 7 |
| Rev@contig22 | 7 | bdu250 | GTACAACAAACACGTGGACAATC |  |
| Fwd@contig45 | 8 | bdu251 | CTGTTACGTTTAAAATATGTGGC | 8 |
| Rev@contig12 | 8 | bdu252 | TTTAACTTGTCTAGTCCAGC | 7A,8 |
| Fwd@contig24 | 2 | bdu253 | GGATTACTCTTCCTCAGCACATTGTTG |  |
| Fwd@contig58Fwd | 3 | bdu254 | CTATTTACAACTGAAACTTGGTCTGA | 11 |
| Rev@contig9Rev | 3 | bdu255 | GGTTTCAGAAATGAAATGATTGAATTCCC |  |
| Fwd@contig11Fwd | 5 | bdu256 | GCAGTGTATAAATCCATATCATCTAACGC |  |
| Rev@contig52Rev | 5 | bdu257 | CGGTTAAAGATTATACTTATCAACGT |  |
| Fwd@contig34 | 6A | bdu260 | CACTTCCCCAAAACCTCCTTGACTCG |  |
| Fwd@contig10Rev | 1 | bdu301 | GGAGAATTAACATCATTATG |  |
| Fwd@contig10Fwd | 1A | bdu303 | GAGCATGGCGCTTCCTCC |  |
| Fwd@contig7Rev | 1A | bdu304 | GCTATCTTTTTATTTGCTC |  |
| Fwd@contig7Rev | 1A | bdu305 | GCCACCATCTATCCAACTTGC |  |
| Rev@contigontg24 | 2 | bdu306 | CTAGCAAATGAAACAGCTG |  |
| Fwd@contig24Fwd | 2 | bdu307 | CATCAGAACCAAACATAATAT | 10 |
| Rev@contig25Rev | 2 | bdu308 | CAATTCCTATGGTGGTGTCGTTGCAC |  |
| Rev@contig25Fwd | 2 | bdu309 | CAGAAGCTACAGAATTACTG |  |
| Fwd@contig25Fwd | 2 | bdu310 | CTTTTGATGTATTATGTTC |  |
| Fwd@contig58Rev | 10 | bdu311 | GAATTGTCATTAACATTACG |  |
| Rev@contig58Fwd | 10 | bdu312 | GTATTGTTGAACAAAAAGGTAAAG |  |
| Rev@contig58Fwd2 | 3 | bdu313 | CAACAAAAGTAAAGTTTAG |  |
| Fwd@contig58Fwd2 | 3 | bdu314 | CATCTTCATTGCTTCGTTTTTACG |  |
| Rev@contig9Rev2 | 3 | bdu315a | CGACAGACCCTATTCCTTGGTTACG | 10 |
| Fev@contig9Rev | 11 | bdu315 | GCCGACGGTATTGCTTTTG |  |
| Rev@contig9Fwd | 11 | bdu316 | GTTGCAGAATTCATTTAAAGATG | 11 |
| Fwd@contig9Fwd | 4 | bdu317 | GCTTCTATATGTTTTAAATTC |  |
| Rev@contig11Fwd | 4 | bdu318 | GTGAAATCATCGACTTATTGA |  |
| Rev@contig11Fwd2 | 5 | bdu319 | CGTTCTTAAAAGGTACTGGTAAAG |  |
| Fwd@contig11Fwd2 | 5 | bdu320 | CTAGAAACTCTTTCAGC | 12 |
| Rev@contig52Rev2 | 12 | bdu321 | GGTATGGATTCGACTTGTCC |  |
| Fwd@contig52Rev | 12 | bdu322 | CAAGTGCTTTAGACATGTCTTTG |  |
| Fwd@contig42Rev | 6 | bdu324 | CTCAAGCGTATCCTCATCACTTTC |  |
| Rev@contig42 | 6A | bdu325 | GTATCAGTATTAATAGTGATCGTGC |  |
| Fwd@contig42 | 6A | bdu326 | CTACATCATCATTAGCCGTCATC |  |
| Rev@contig34Rev | 6A | bdu337 | GGCACTAGCACTGATAATTGA |  |
| Fwd@contig34 | 6A | bdu338 | AGCAATTATTTTTCTTAAGAG | 13 |
| Fwd@contig22 | 7 | bdu341 | CATAGTTCCGCGTTTGTAACCTACC |  |
| Rev@contig6 | 7 | bdu342 | GAAACAATACAAACATCTATCAATCAC |  |
| Rev@contig6Fwd | 7A | bdu343 | TGAAAATAACACAGATGAACAGGAC | 13 |
| Fwd@contig6Fwd | 7A | bdu344 | GATAATAAGTCTTTTATTTCTTCCTGCG |  |
| Rev@contig45 | 7A | bdu344 | ACCAATCTTTTTATTAGTTTCAATTTC |  |
| Rev@Contig28 | 7A | bdu346 | GTTCAACATACAAGAAAAAGAAAATG |  |
| Fwd@contig28 | 8 | bdu347 | CATTTTCTTTTTCTTGTATGTTGAAC |  |
| Rev@contig38 | 8 | bdu348 | CTTTTAAAGCTATATCATGTG |  |
| Fwd@contig38 | 8 | bdu349 | GGCGTCTTTATTATAAATGGAGATGC | 14 |
| Fwd@contig30Rev | 1 | bdu350 | GATCGGAATTTTTGTTGCAATATACGG |  |
| Fwd@contig12 | 1 | bdu351 | GCAAGAAATATAAAGATTGAGC |  |
| Rev@contig12Fwd | 1 | bdu352 | TCTAACAAATGAGAATACACGGATGTAG | 14 |
| Rev@contig12Rev1 | 8 | bdu359 | GAATCTACTATGGAATCATTATTTGGCAATGAC |  |
| Fwd@contig22Fwd | 7 | bdu360 | GCATGATCACCATGTTCGTGTGTGATAAGACACC | 13 |
| Fwd@contig38Fwd1 | 14 | bdu361 | CGCGATAAAAGTTATGATTTCAAATGAATACTCTG | |
| Rev@contig6Rev | 7A | bdu362 | GAGATGCAATGAAGTCAGCAATGTTACACTG |  |
| Fwd@contig38Fwd2 | 14 | bdu363 | CTTATGAGTTACCTAAAGATTTATC |  |
| Rev@contig12Rev | 7A | bdu364 | TTCATTCCAAATAATGATTCCATAG |  |
| Fwd@contig10rev | 15 | bdu366 | GACCAAATTCATCAAAACAC | 15 |
| Fwd@contig10 | 15 | bdu367 | GGAATCAGTACACACCATC |  |
| Rev@contig10-1 | 15 | bdu368 | CATTTTACCTAGTAACTCAAC |  |
| Fwd@contig10-2 | 15 | bdu369 | GTTGAGTTACTAGGTAAAATG |  |
| Rev@contig10-2 | 15 | bdu370 | CATAGAAATATTACAGTCGC |  |
| Fwd@contig10-3 | 15 | bdu371 | GCGACTGTAATATTTCTATG |  |
| Rev@conig10-3 | 15 | bdu372 | GTATGTTATAGCTAGCC |  |
| Fwd@contig10fwd | 15 | bdu373 | GTCGAATGAACTTGATGC |  |
| Rev@contig10fwd-2 | 15 | bdu374 | GGCATGAGAGATTGATTGTG |  |
| Fwd@contig7rev2 | 15 | bdu375 | CACCTATACCTTTAGTAG |  |
| Rev@contig7rev | 15 | bdu376 | GCATTGATAGGTCTAGTAATC | 15 |
| Fwd@contig7-1 | 16 | bdu377 | CCATTAAATGCATCTGTG | 16 |
| Rev@contig7-1 | 16 | bdu378 | CAGGTCATCAATGGTATAATG |  |
| Fwd@contig7-2 | 16 | bdu379 | CATTATACCATTGATGACCTG |  |
| Rev@contig7-2 | 16 | bdu380 | GACAGGAAGCATACTGC |  |
| Fwd@contig7-3 | 16 | bdu381 | GCAGTATGCTTCCTGTC |  |
| Rev@contig7-3 | 16 | bdu382 | CGCAAGTAGATGTTGCGC |  |
| Rev@fwdcontig24 | 16 | bdu383 | GGAGTCAAAGAATGAAGG |  |
| Fwd@contig4-1 | 16 | bdu395 | GGAGTAGTATTATAAAATGG |  |
| Fwd@contig24fwd2 | 16 | bdu384 | GGCCCCACATTCAAAGC | 16 |
| Rev@contig4-1 | 4 | bdu396 | CCATTTTATAATACTACTCC |  |
| Fwd@contig4-2 | 4 | bdu397 | CAAAACAACTATGTAGC |  |
| Rev@contig4-2 | 4 | bdu398 | CGTTAATTTAGCGAAACC |  |
| Fwd@contig4-3 | 4 | bdu399 | CAAAATAAGTATTTTGAAACG |  |
| Rev@contig4-3 | 4 | bdu400 | GGAGTAGTATTATAAAATGG |  |
| Rev@contig4-4 | 4 | bdu402 | CAAAACAACTATGTAGC |  |
| Fwd@contig4fwd | 17 | bdu403 | CGTTAATTTAGCGAAACC | 17 |
| Rev@contig11-1 | 17 | bdu404 | CAAAATAAGTATTTTGAAACG |  |
| Eev@contig11-2 | 17 | bdu406 | TTCGTGATCCGCAAGGTG |  |
| Fwd@contig11-3 | 17 | bdu407 | CACCTTGCGGATCACGAA |  |
| Rev@contig11-3 | 17 | bdu408 | GCATATCTAGCGCGTATAAG |  |
| Fwd@contig11fwd2 | 17 | bdu409 | GACCTTTAATTTCACCATAG |  |
| Rev@contig11fwd2 | 17 | bdu410 | CGCATTTGCGAATGTTTCCG |  |
| Fwd@contig11fwd3 | 17 | bdu411 | GAAGGATTGTGAATCATCA |  |
| Rev@contig52rev | 17 | bdu412 | GCGCGAGCTGTCGCTCAAAGTC | 17 |
| fwd@contig34fwd | 18 | bdu415 | GCATCCCTTTTAACTTGTTTC |  |
| Fwd@contig8-1 | 18 | bdu416 | GCGCTTTCTTATATACG |  |
| Fwd@contig8-2 | 18 | bdu417 | GCTTTTCTCGCCATTTTATCC |  |
| Fwd@contig14-1 | 18 | bdu418 | CATAATCGTCTAAATACCAACG |  |
| Rev@contig14-1 | 18 | bdu419 | GATAACGGATTCGGTTTC | 18 |
| Fwd@contig22-1 | 7 | bdu420 | GAGCGATCAATACTTATCCAACCAG |  |
| Fwd@contig22-2 | 7 | bdu421 | CATATACACACCTCACTTTC |  |
| Rev@contig22-1 | 7 | bdu422 | CATTTAATTCATTTAAGTAGTC |  |
| Fwd@contig22Fwd1 | 7 | bdu423 | CTTGCATTTGTTCATAG |  |
| Rev@contig21-1 | 7 | bdu424 | CAGCACGGATGACGCTAGTC |  |
| Fwd@contig6-1 | 19 | bdu425 | CGATTGATATTGACACTTCAATTTCTG | 19 |
| Rev@contig6-1 | 19 | bdu426 | CAAAGATATCAAAGATATTCCG |  |
| Fwd@contig6-2 | 19 | bdu427 | CGGAATATCTTTGATATCTTTG |  |
| Rev@contig6-2 | 19 | bdu428 | GACAAAGTATATTCGTCG |  |
| Fwd@contig6fwd1 | 19 | bdu429 | GTGCTATCCAATAAGTAAGC |  |
| Rev@contig45-1 | 19 | bdu430 | CCAAATACAACATTATCTTTG | 19 |
| Fwd@cnitg28-1 | 20 | bdu431 | CGCAACATGTTTAGCTAC | 20 |
| Rev@contig38-1 | 20 | bdu432 | CTATCCTTAAAACTGTTC |  |
| Fwd@contig38-1 | 20 | bdu433 | GAAGCGAAACAAGACAAAGTC |  |
| Rev@contig38-1 | 20 | bdu434 | CTATCCTTAAAACTGTTC |  |
| Fwd@contig12-1 | 20 | bdu435 | CTATCCTTAAAACTGTTC |  |
| Rev@contig12-1 | 20 | bdu436 | GCTGACCGAATAGCACCGTTTG | 20 |
| Rev@contig30-1 | 1 | bdu437 | GAGTTTGATTATTTTCTTGATA |  |
| Fwd@Contig10-1 | 1 | bdu438 | CATTATGAAAAAGAAATTAG |  |
| 16Ssouthfwd#1 |  | bdu439 | GCAGACTACAATCCGAACTGAG |  |
| 16Ssouthrev#1 |  | bdu440 | GCTCGTGTCGTGAGATGTTG |  |

**Table S3: Putative gene products identified in bacteriophage contigs following 454 sequencing (numbers adjacent to gene name identify contig number). Contig sequences are located in (File S2).**

**NRS18 (VISA)**

20 Mannosyl-glycoprotein

endo-β-N-acetylglucosamine

54 hypothetical; RinB

58 hypothetical

87 hypothetical

196 hypothetical

**NRS19 (VISA)**

4 phage tail tape measure

6 hypothetical; RecF N-terminal domain protein

7 Enterotoxin type A

8 hypothetical protein

9 tail tape measure protein

10 amidase, CHIPS

11 hypothetical

12 integrase

13 terminase; hypothetical

14 DnaD

16 holin/amidase

19 phage tape measure protein

21 recombinase

22 ssDNA binding protein

24 hypothetical

25 minor structural protein

28 AntA/AntB

30 hypothetical; SCIN

33 hypothetical

34 dUTP triphosphatase/

35 hypothetical

38 peptidase; transcriptional repressor

42 hypothetical

43 hypothetical

45 hypothetical

52 phage terminase

55 HIRAN

58 phage tail domain protein

61 virulence-assoc. protein E

68 hypothetical

74 hypothetical

**NRS21 (VISA)**

11 Phage head morphogenesis protein; hypothetical

**NRS26 (VISA)**

12 hypothetical

13 DnaA domain protein

16 Virulence-associated protein E

31 holin/autolysin

32 major tail protein

38 terminase

60 hypothetical; tail tape measure

66 hydrolase; AP2 domain protein

77 invertase

99 N-acetylglucosaminidase

100 cell wall hydrolase; mannosyl-glycoprotein endo-beta-N-acetylglucosaminidase

138 lipase

163 capsid protein, Clp protease

165 hypothetical

175 phage tail; terminase

180 phage head morphogenesis

189 hypothetical

225 DnaC; hypothetical

229 PD-(DE)XK nuclease

286 phage major tail protein

337 phage protein

374 terminase

487 hypothetical

526 amidase

595 endopeptidase tail

630 enterotoxin type I

645 hypothetical

701 hypothetical

716 transposase

796 hypothetical

805 sigmaB-controlled protein

876 hypothetical

948 hypothetical

998 hypothetical

1062 hypothetical

1113 phage helicase

1140 hydrolase

1156 portal protein

1194 ear protein

1196 hypothetical

1214 hypothetical

1230 XRE family transcriptional regulator

1322 hypothetical

1329 prophage endopeptidase tail

1434 DNA binding/pathogenicity island family protein

1444 DNA polymerase

1473 tail tape measure protein

1511 hypothetical

1531 tape measure protein

1565 integrase

1594 hypothetical

1678 hypothetical

1729 XRE family transcriptional regulator

1798 dUTP diphosphatase family

1801 hypothetical

1837 tail fiber protein

1888 tail tape measure

1910 hypothetical

1915 hypothetical

1923 hypothetical

1925 hypothetical

1928 integrase

1935 dUTP pyrophosphatase

1967 hypothetical

**NRS27 (VISA)**

11 hypothetical

12 hypothetical

16 hypothetical

17 hypothetical

20 hypothetical

21 RecT-family protein

22 tape measure protein

31 tail tape measure protein

49 hypothetical protein

**NRS28 (VISA)**

4 phage antirepressor

13 hypothetical; tail assembly protein

17 hypothetical

26 hypothetical protein/major head protein

27 PD-(D/E)XK nuclease superfamily protein

29 phage portal protein

37 head morphogenesis protein

39 major teichoic acid biosynthesis protein C

**NRS29 (VISA)**

9 hypothetical phage protein

11 ligase A protein

19 prophage endopeptidase tail

**NRS104 (MSSA)**

2 tape measure protein

3 major teichoic acid biosynthesis protein

4 hypothetical

5 hypothetical

6 prophage endopeptidase tail

7 tail assembly protein

8 phage tail protein

9 hypothetical

12 tail assembly protein

14 hypothetical

**NRS120 (MRSA)**

18 hypothetical protein

25 Pathogenicity island protein

29 tail length tape measure

42 PVL-like family protein

**MRSA123 (MRSA)**

14 hypothetical; phage tail

**MSSA145 (MSSA)**

83 n/a-hypothetical protein

92 bacteriophage holin

201 phage minor structural protein

**MSSA161 (MSSA)**

11 radical SAM protein/Fe-S oxidoreductase

**MRSA408 (MRSA)**

8 hypothetical; tape measure

9 hypothetical; RecF

12 tape measure

13 tape measure

15 dUTP diphosphatase

16 hypothetical

18 prophage endopetidiase tail

19 hypothetical

20 amidase

21 Recombinase

22 ssDNA binding protein

23 integrase

25 RusA/ DnaD

27 hypothetical

28 hypothetical

29 portal protein

31 tail tape measure protein

32 tail tape measure protein

33 phage major tail protein

34 Phage tail domain protein

35 integrase

36 hypothetical; tail protein

37 terminase; portal protein

38 phospholipase C precursor

39 SPP1 family phage head morphogenesis protein

44 phage tail fiber

45 phospholipase C precursor

46 phage tape measure protein

47 hypothetical protein

48 PemK-like family protein/CI-like repressor

51 phage major tail protein, TP901-1 family

53 hypothetical protein

54 mannosyl-glycoprotein endo-beta-N-acetylglucosaminidase

55 hypothetical phage protein

56 Putative major teichoic acid biosynthesis protein C

57 amidase

58 hypothetical protein phage protein

59 phospholipase C precursor (pseudogene)

62 hypothetical

63 hypothetical

65 holin, SPP1 family

66 hypothetical

69 phospholipase C precursor

70 phage terminase

72 capsid protein

73 hypothetical

74 hypothetical

75 hypothetical

76 hypothetical

77 hypothetical

84 hypothetical

89 phospholipase C precursor (pseudogene)

90 hypothetical protein

91 phage repressor

93 phage N-acetylglucosaminidase

94 hypothetical protein phage protein

99 phospholipase C precursor; hypothetical

100 major tail protein/hypothetical protein

102 RinA

104 virulence-associated E protein

106 hypothetical

109 hypothetical

112 hypothetical

113 Terminase large subunit

116 virulence-assoc. protein E

118 phospholipase C precursor

119 hypothetical

120 phage portal protein,

121 L54a, Cro-related protein

123 hypothetical

126 DNA polymerase

127 phage major tail protein,

128 phage tape measure protein

129 phospholipase C precursor

133 phospholipase C precursor

135 lytic protein

138 RinB

157 hypothetical

**Table S4: List of putative genes identified in plasmids identified in NRS108.**

| **pBU108a** | |  |  |  |
| --- | --- | --- | --- | --- |
| **Start** | **Stop** |  | **Abbreviation** | **Putative Gene Product/Description** |
| 1664 | 1221 | C | ssb | Single-strand DNA binding protein |
| 2821 | 1679 | C |  | Hypothetical protein |
| 3167 | 2871 | C |  | Hypothetical protein |
| 3365 | 3171 | C |  | Hypothetical protein |
| 3917 | 3627 |  |  | Hypothetical protein |
| 4335 | 4655 | C |  | Hypothetical protein |
| 5045 | 4749 | C | ftsK | Cell division protein FtsK |
| 4882 | 5031 |  |  | Hypothetical protein |
| 6082 | 5099 | C | repA | Replication initiation protein |
| 4882 | 5031 | C |  | Hypothetical |
| 6742 | 7608 |  | parA | CobQ/CobB/MinD/ParA nucleotide binding domain protein |
| 7601 | 7822 |  |  | Hypothetical protein |
| 8002 | 8184 |  |  | Hypothetical protein |
| 8267 | 8506 |  |  | Hypothetical protein |
| 8831 | 9043 |  |  | Hypothetical protein |
| 9151 | 9438 |  | mutS | DNA mismatch repair protein MutS |
| 9513 | 9695 |  |  | Hypothetical protein |
| 9734 | 10683 |  |  | Hypothetical protein |
| 10810 | 10959 |  |  | Hypothetical protein |
| 10992 | 11441 |  |  | Hypothetical protein |
| 11459 | 11689 |  |  | Hypothetical protein |
| 12206 | 11790 | C | yolD | YolD-like protein |
| 13426 | 13980 |  | res | Resolvase |
| 14242 | 14607 |  |  | Hypothetical protein |
| 15092 | 15211 |  |  | Hypothetical protein |
| 16083 | 15478 | C | end | Restriction endonuclease |
| 17102 | 16086 | C | ltrC | LtrC-like protein |
| 18506 | 17157 | C | virA | Viral A-type inclusion protein |
| 20665 | 18548 | C | topo3 | DNA topoisomerase III |
| 21363 | 20689 | C |  | Hypothetical protein |
| 22708 | 21383 | C | ltrC | LtrC-like protein |
| 23268 | 22705 | C |  | Hypothetical protein |
| 23657 | 23271 | C |  | Hypothetical protein |
| 24815 | 23667 | C |  | Hypothetical protein |
| 25090 | 24836 | C |  | Hypothetical protein |
| 26191 | 25346 | C |  | Hypothetical protein |
| 26815 | 26204 | C |  | Hypothetical protein |
| 27942 | 26836 | C | ssaA | Secretory antigen SsaA-like protein; transposon-related protein |
| 29942 | 27945 | C | trsE | TrsE protein |
| 30750 | 29962 | C |  | Hypothetical protein |
| 31069 | 30704 | C |  | Hypothetical protein |
| 32921 | 31080 | C |  | Hypothetical protein |
| 33374 | 32922 | C |  | Hypothetical protein |
| 1206 | 33371 | C | tss4 | Type IV conjugative secretion system |
|  |  |  |  |  |
| **pBU108b** | |  |  |  |
| **Start** | **Stop** |  | **Abbreviation** | **Putative Gene Product/Description** |
|  |  |  |  |  |
| 15 | 821 |  | entJ | Enterotoxin type J |
| 1689 | 911 | C | entR | Enterotoxin type R |
| 3207 | 2236 | C | oxi | YhdH/YhfP-family quinone oxidoreductase alcohol dehydrogenase |
| 3331 | 3750 |  | marR | MarR regulatory protein |
| 4714 | 4103 | C | sin | Recombinase |
| 5419 | 4841 | C | binR | Invertase |
| 6063 | 5683 | C | blaI | Beta-lactamase repressor |
| 7852 | 6053 | C | blaR-1 | Beta-lactamase regulatory protein |
| 7917 | 8761 |  | blaZ | Beta-lactamase regulatory protein |
| 10886 | 9115 | C | abiK | Bacteriophage abortive infection mechanism |
| 11199 | 11032 | C |  | Hypothetical protein |
| 12203 | 11595 | C |  | Hypothetical protein |
| 12221 | 11696 | C |  | Hypothetical protein |
| 12395 | 12231 | C |  | Hypothetical protein |
| 12283 | 12771 |  | ftsK | FtsK cell division protein |
| 13689 | 13853 |  |  | Hypothetical protein |
| 14300 | 14160 | C | rep | Replication protein |
| 14829 | 14482 | C | cadX | Cadmium efflux system accessory protein |
| 15477 | 14848 | C | cadD | Cadmium resistance transporter |
| 15835 | 15641 | C |  | Hypothetical protein |
| 15852 | 16075 | C |  | Hypothetical protein |
| 16483 | 16358 | C |  | Hypothetical protein |
| 16756 | 16610 | C |  | Hypothetical protein |
| 16949 | 16761 | C | rep | Replication protein |
| 18023 | 17079 | C | repA | Replication initiation protein |
| 18422 | 19237 |  | rep | Replication protein |
| 19341 | 19742 |  | rep | Replication protein |
| 19855 | 20049 |  | rep | Replication protein |
| 20760 | 21437 |  |  | Hypothetical protein |
| 21800 | 21531 | C |  | Hypothetical protein |
| 21921 | 22064 |  |  | Hypothetical protein |
| 22352 | 22462 |  |  | Hypothetical protein |
| 22495 | 23148 |  |  | Hypothetical protein |
| 23374 | 23499 |  |  | Hypothetical protein |
| 25180 | 24144 | C |  | Hypothetical protein |
| 26550 | 25775 |  | entP | Enterotoxin-type P |
| 27120 | 27287 |  |  | Hypothetical protein |
|  |  |  |  |  |
| **pBU108c** | |  |  |  |
| **Start** | **Stop** |  | **Abbreviation** | **Putative Gene Product/Description** |
|  |  |  |  |  |
| 2305 | 567 | C | ermC | Erythromycin resistance |
| 1858 | 1382 | C | repL | Replication protein |

**Table S5: Sequencing Statistics of Extra-chromosomal Fractions**

| **Sample** | **#reads** | **# bases** | **# contigs (large)** | **assembled size** | **Q-39 score** |
| --- | --- | --- | --- | --- | --- |
| VRS2 | 17,046 | 4,891,590 | 26 (19) | 121,332 | 0.45% |
| VRS3 | 19,404 | 5,536,472 | 20 (13) | 97,073 | 0.31% |
| NRS18 | 8,925 | 2,525,116 | 210 (105) | 111,925 | 7.14% |
| NRS19 | 42,971 | 12,455,574 | 81 (44) | 69,492 | 4.16% |
| NRS21 | 9,278 | 2,628,699 | 16 (12) | 63,153 | 0.86% |
| NRS24 | 15,336 | 3,067,313 | 19 (9) | 67,057 | 0.84% |
| NRS26 | 50,236 | 14,300,894 | 1993 (1635) | 1,986,310 | 5.90% |
| NRS27 | 34,488 | 6,996,429 | 49 (12) | 77,398 | 0.90% |
| NRS28 | 20,777 | 5,851,630 | 44 (24) | 44,835 | 2.38% |
| NRS29 | 28,212 | 8,214,967 | 39 (18) | 52,458 | 1.36% |
| NRS35 | 22,604 | 4,579,683 | 3 (3) | 31,672 | 0.23% |
| NRS53 | 53,213 | 11,045,363 | 71 (19) | 73,766 | 1.48% |
| NRS70 | 15,814 | 4,474,267 | 47 (19) | 49,698 | 2.14% |
| NRS104 | 58,307 | 11,847,974 | 17 (5) | 34,839 | 0.88% |
| NRS108 | 68,358 | 13,898,507 | 53 (28) | 93538 | 1.99% |
| NRS109 | 63,402 | 13,080,628 | 26 (4) | 40,012 | 0.71% |
| NRS119 | 20,863 | 5,979,904 | 19 (12) | 34,115 | 0.63% |
| NRS120 | 36,233 | 10,570,711 | 44 (20) | 45,579 | 1.08% |
| NRS123 | 47,638 | 9,706,886 | 16 (10) | 33,970 | 2.47% |
| NRS145 | 30,910 | 6,460,971 | 500 (132) | 234,837 | 5.99% |
| NRS149 | 5,826 | 1,150,171 | 2 (2) | 27,481 | 0.41% |
| NRS161 | 33,482 | 6,668,583 | 13 (4) | 38,131 | 0.82% |
| NRS271 | 19,019 | 5,375,293 | 6 (2) | 36,443 | 0.69% |
| NRS408 | 71,651 | 15,130,756 | 158 (60) | 118,555 | 4.50% |

**Table S6: List of putative genes identified in ϕBU01.**

| **Start** | **Stop** | **Strand** | **Abbreviation** | **Putative Gene Product/Description** |
| --- | --- | --- | --- | --- |
| 373 | 723 | C | SCIN | Staphylococcal complement inhibitor |
| 1394 | 1855 |  | CHIPS | Chemotaxis inhibitory protein |
| 2931 | 3461 | C | sak | Staphylokinase |
| 3613 | 4386 | C |  | Amidase |
| 4380 | 4685 | C | ent | Enterotoxin type A/P |
| 5225 | 6085 | C | ent | Enterotoxin type A/P |
| 6419 | 6793 | C |  | Hypothetical |
| 6849 | 7139 | C |  | Hypothetical |
| 7325 | 8833 | C | ms | Minor structural |
| 8781 | 11111 | C | ms | Minor Structural |
| 11127 | 12611 | C | tp | Tail protein |
| 12608 | 17152 | C | tmp | Tape measure protein |
| 17797 | 18021 | C |  | Hypothetical |
| 18063 | 18707 | C | mtp | Major tail protein |
| 18708 | 19118 | C |  | Hypothetical |
| 18834 | 19160 |  |  | Hypothetical |
| 19112 | 19516 | C |  | Hypothetical |
| 19513 | 19875 | C | hta | Head-tail adaptor |
| 20437 | 21657 | C |  | Capsid |
| 21605 | 22354 | C | clpP | Protease |
| 22326 | 23489 | C |  | Portal |
| 23505 | 25166 | C |  | Terminase |
| 25163 | 25552 | C |  | Hypothetical |
| 25638 | 25940 | C | HNH | HNH endonuclease |
| 26169 | 26585 | C | rinA | Transcriptional activator |
| 26959 | 27354 | C |  | Hypothetical |
| 28865 | 29242 | C |  | Hypothetical |
| 27827 | 28387 | C | dUTPase | dUTPase |
| 28356 | 28676 | C |  | Hypothetical |
| 29246 | 29668 | C | rusA | DNA replication, recombination, and repair |
| 29884 | 30783 | C | dnaD | Replication protein |
| 30807 | 31277 | C | ssb | Single-stranded DNA binding protein |
| 31976 | 32896 | C | recT | Recombination |
| 32898 | 34376 | C | recF | RecF/RecN/SN Recombination |
| 34291 | 34821 | C |  | Hypothetical |
| 35390 | 35692 | C |  | Hypothetical |
| 35945 | 36304 | C |  | Hypothetical |
| 36314 | 36703 |  |  | Hypothetical |
| 36903 | 37361 | C | ant | Antirepressor |
| 37668 | 38114 | C | A/B | AntA/B Anti-repressor |
| 37972 | 38262 | C |  | Hypothetical |
| 38127 | 38465 | C | cro | Repressor |
| 38521 | 39249 |  | repr | Repressor |
| 39246 | 40115 |  | HIRAN | Nucleic acid binding protein |
| 41127 | 41708 | C |  | Hypothetical |
| 41792 | 42853 |  | int | Integrase |

**Table S7: Comparison of predicted and actual ϕBU01 sequencing results**

The following calculates the % by mass of ϕBU01 expected from a typical extra-chromosomal preparation, if the presence of the closed, circular prophage was due solely to spontaneous excision/induction events. Then, the comparison is made between the predicted % ϕBU01 in a sample and the actual % ϕBU01 calculated by sequencing reads. The average number of NRS19 cells/prep was calculated by CFU counts, and the average DNA yield was ~ 1μg.

REFERENCES

1. (2002) Vancomycin-resistant *Staphylococcus aureus*--Pennsylvania, 2002. MMWR Morb Mortal Wkly Rep 51: 902.

2. (2004) Vancomycin-resistant *Staphylococcus aureus*--New York, 2004. MMWR Morb Mortal Wkly Rep 53: 322-323.

3. Critchley IA, Young CL, Stone KC, Ochsner UA, Guiles J, et al. (2005) Antibacterial activity of REP8839, a new antibiotic for topical use. J Antimicrob Chemother 49: 4247-4252.

4. Watanabe Y, Cui LZ, Katayama Y, Kozue K, Hiramatsu K (2011) Impact of rpoB Mutations on Reduced Vancomycin Susceptibility in *Staphylococcus aureus*. J Clin Microbiol 49: 2680-2684.

5. Ploy MC, Grelaud C, Martin C, de Lumley L, Denis F (1998) First clinical isolate of vancomycin-intermediate *Staphylococcus aureus* in a French hospital. Lancet 351: 1212-1212.

6. Kuroda M, Ohta T, Uchiyama I, Baba T, Yuzawa H, et al. (2001) Whole genome sequencing of meticillin-resistant *Staphylococcus aureus*. Lancet 357: 1225-1240.

7. Lelievre H, Lina G, Jones ME, Olive C, Forey F, et al. (1999) Emergence and spread in French hospitals of methicillin-resistant *Staphylococcus aureus* with increasing susceptibility to gentamicin and other antibiotics. J Clin Microbiol 37: 3452-3457.

8. Bohach GA, Schlievert PM (1989) Conservation of the biologically active portions of staphylococcal enterotoxins C1 and C2. Infect Immun 57: 2249-2252.

9. Tsiodras S, Gold HS, Sakoulas G, Eliopoulos GM, Wennersten C, et al. (2001) Linezolid resistance in a clinical isolate of *Staphylococcus aureus*. Lancet 358: 207-208.

10. McDougal LK, Steward CD, Killgore GE, Chaitram JM, McAllister SK, et al. (2003) Pulsed-field gel electrophoresis typing of oxacillin-resistant *Staphylococcus aureus* isolates from the United States: establishing a national database. J Clin Microbiol 41: 5113-5120.

11. Baba T, Takeuchi F, Kuroda M, Yuzawa H, Aoki K, et al. (2002) Genome and virulence determinants of high virulence community-acquired MRSA. Lancet 359: 1819-1827.

12. Kreiswirth BN, Lofdahl S, Betley MJ, O'Reilly M, Schlievert PM, et al. (1983) The toxic shock syndrome exotoxin structural gene is not detectably transmitted by a prophage. Nature 305: 709-712.

13. Lindsay JA, Ruzin A, Ross HF, Kurepina N, Novick RP (1998) The gene for toxic shock toxin is carried by a family of mobile pathogenicity islands in *Staphylococcus aureus*. Mol Microbiol 29: 527-543.

14. Shinefield HR, Ribble JC, Boris M (1971) Bacterial interference between strains of *Staphylococcus aureus*, 1960 to 1970. Am J Dis Child 121: 148-152.

15. Wilson P, Andrews JA, Charlesworth R, Walesby R, Singer M, et al. (2003) Linezolid resistance in clinical isolates of *Staphylococcus aureus*. J Antimicrob Chemother *5*1: 186-188.

16. Weigel LM, Clewell DB, Gill SR, Clark NC, McDougal LK, et al. (2003) Genetic analysis of a high-level vancomycin-resistant isolate of *Staphylococcus aureus*. Science 302: 1569-1571.

17. Duthie ES, Lorenz LL (1952) Staphylococcal coagulase; mode of action and antigenicity. J Gen Microbiol 6: 95-107.
