# Supplementary material for: Beyond the Chromosome: The Prevalence of Unique Extra-Chromosomal Bacteriophages with Integrated Virulence Genes in Pathogenic Staphylococcus aureus
Source: PLoS One. 2014 Jun 25;9(6):e100502. doi: 10.1371/journal.pone.0100502 (PMC4070920; doi:10.1371/journal.pone.0100502)
Supplement: File S2 — Preliminary alignments of 454 sequencing contigs identified as bacteriophage DNA. (DOCX) [file pone.0100502.s002.docx]

**File S2: Preliminary alignments of 454 sequencing contigs identified as bacteriophage DNA**

>NRS18contig00020 [Organism=Staphylococcus aureus] GGGGCTCATTTTtGAATATAGAGCCACCTTCCATAAACAAATGCAATCTAGgTGTTAAGCCATgtGCTTTTAGATCGATACGACCTTGTTtGTCATTGATACCTATTCTTATAGATGCTGTATTTtCATCTTCAGTGTAAAaTCGACAGCCAaTGTCACCTAAGTCAACACCATCATTTTtATTCTCGTTTCAACATCTTTtATTTGTACATTTATACACCTCTTTATTTATATTTATCTCTTATAAAGTAGATACCTTTtAAGCCGATTTGTTTATATAGCTTAGCGATTGTACTAGCTTGATGTTGGCACCACTCTATAGCAGTAGCGTATTGGTGCGTAGCTGGATTCTTAGGATTCCATCTGATTCTGTACAGTGTATtCTGtCcTTTGTTGATGTAAtCCTTtcTTACGAAGCTAGCACCGCCCATGATTGCTTTTGCTGgAGATGTCCAACCTTTATtcTtAGCAAATttCATTGCATAATCAGGgTCGTTGTCGAATGCACCAATACCGAAGTAATTATATGCACCGTATcTACCACTAGCGAAGTTACTTGTtCCGTATCCACTTTCTAaGAAAGCGTGCGCGATCAAATAGATTtCGTTAATGTtGTTTTCTTACAGGCTTCTGCAAATGCTTTGCCTTGTCCGTCTAGCGTTCCTTTCCCTTTgaGTATCTTATTAAGCGcaCTAACTGAAACGCCTTGATACTTGCCTAAATTAAGCATTTgaTAGCATtGtGTGTTACTTTCCCATATTCGcTtAACATTCATTGCTGagCTCGTT

>NRS18contig00054 [Organism=Staphylococcus aureus]

cgattgaattgctatcatattccggtattacatcagctaacacattacacttcattttatgtgTGCCTATCATATTAACTATTGACTCTTTGCTATACATCTACTCTGaCACCTCCGCCCTCATCAAATCAGACTGATCGCTCAACTTTGCGAAGTCACTCGGCGCctCTgCATCATCATTAGCCGTCATCATAATATATACTTGCTCAGTTACATACTTGCCTAGCTCATACATCGCTAGTAAGAATAaTAATCTTAGTATTTGTTTAGTCATTTCCCACACTCCCTTATATTTtCAAACAACTGCCCTAATTTAATAACTGCATTTCTTTTAACTTGTGCCTcGTACTTCTCTTTCGCTTCTTCTttACTCTCCGCATCAaCAACTGTAAACCTTTGATTGCTCTTAGCTTTAGTTATGTGTGTATGCTTACGTCCTGTTGAATCTTTAAAtGTTGTGACTAGGTATTGTGTCaTTCCTCaTAGCTCCCTTGAACTTGTTtGAGCTTACTCATAAAAAaCaTTACTAAAAATGCTATTAAGATATGCGTCTTTTGATGTTTATCAGCAAATGTaGATGTcACAAAGaTAGtAGCAATCAaCAaCaTTTCATATAGGTTtGTGTgTATagtctttttac

>NRS18contig00058 [Organism=Staphylococcus aureus]

TTCTGCGAAAaCAGTCGgAATtAACTCATAATTTtCTTCggttttttCGTTTAAAGGTACAGGGTTAAATGATTTGGTtGAACCGTCTAAACCCTTATCGAAAAGCAGCCATTGTAATGCGGTTGCTGTTGTAGTCTTGCCAGTCGCATTATTGCCGTATATTTTtGCATCTTTACCGTCAAAGTtAAaTTTTCTtCTTTGATTCCAGCAAAGTTCGATATAGTTAACTtATTTATTTtCATATCTTTCCTCATGCTCCTTTTTtAAtCTTCCGATGACCTCTTAGCACCTCGATAaTtAAaTTTTttaTtCGTTCATGGCTGTCTGGATTGATTTCATGTATCTGCACAAGCTTATtGTTtGTTTtGTAACTGTCGTGATAGTGCAAGAAATTAaTCGATAaGTATCcGTGATGATTACGTtCAaTTtCCAaTAATGCtCGTtGGTTtGACAAaGTATATTCGTCGAATAACGTCTTAAAAaTaTTCAATaTATTTCTTTCTGTATCTCTcATGCTTACACCTACCATCTcATGACTAAGTTAaTtaGTCtGTCcTGTTCGTCTGTGTTCTCTTcAaTCCATtCATctATtGCTTGGTTGAAtAAGtCTGATGCcATATCTAAGTC

>NRS18contig00087 [Organism=Staphylococcus aureus]

CaTATTTACGTTTTAAATTATTCAGCAAATTCATACGAGATTCATACTCGTTTAACACTTGTTCGTCGAATTCTGTATTAGCCATTTCATCATATAACTCATGTTTTGCATCTTCTAAAATGTAATAAAATTGATCAATATCTTCTTTtAATTTGTCATATTTATTtGGAACTATATCGTTtATTGTTAACAAATGGTTGCTTAGTTCATACAAaCGATCAGTGAtAGCATTTTCATCCGTTAAtGtCATATgTGCGTTATTAAGCGCTAAGCTTAATTTTtCAGAGTTTtGAATGCGTTTAAtATCTATTTCAAGTTGCTCTAT

>NRS18contig000196 [Organism=Staphylococcus aureus]

CtCCAaTTATATTAAGGTAACCAACACCACCCCAATATTCATTAACTGCATTGTTGTAAGCTTTTGCTGCTTCaTCTTCATTTACAAaGTGACCTAAGTTTtGGTTTTTtATCAACAGCTATACATGCATACCAATTATTATTTTTTtATCCCATGAAACGCCTTTATATTTAGATGAATTGTTACACTTCGCTTTGCTCCATCTTGTTTTATTACCTTCAGTTGTTAGATTTTTCTTGTGAAATCATTGTTTTtATTTTT

>NRS19contig00004 [organism=Staphylococcus aureus]

GATTCGCAAAATAGTATTCAATGAAGAGTATGATGAAGACGGTGTTTATAAaGAAaCTCAaGGTAAaCAAAACAACTATGTAGCAATTTGGTTCAGACAAGAGCGTCGAGACGGCACATTTAGAACGGTTTtATTACCTAAAGTCATGTTTACAAATCCTAAAATCGATGGAGAAACGGCTGAGAAAGATtGGGATTTCTCAAGTGAaGAGGTTGAAGGTGAGGCACTTTtCCcTTtAGTTGATAATAAAAaGTCAGTACGTAAGTATATCTTTgATTCAGCTAACATGACAAATCATGATGGAGACGGTGAAAaaGGCGAaGAGGCTTTCTTAAAGAAAATTTTAGGCGAAGAATATACTGGAAACGTGACAGAGGGTAACGAAGAAACTTtGTAACAAAACcGGCTTCATCGGAAACTGCGGTAAAGTCGGTTAATATACCAGATAGCATTAAAACACTTAAAGTTGGCGACACATACGATTTAAATGTTGTAGTAGAGCCATCTAATCAAAGTAAGTTATTGAAATACACAACAGATCAAACGAATATTGTATCAATCAATAGTGATGGTCAAGTTACTGCGGAAGCACAAGGCATTGCTACGGTTAAAGCAACAGTTGGtAATATGAGTGaCAcTATAACAaTAAaTgTAGAaGCATAAgAGGGGgCAACCCcTCTATTTtATTTGAAAATAAGgAGtAGTATTATAAAaTGGaCAAAATTAAAACGTAaCATTATTCAaTTAGTAGAAGACCcGAAaGCAAATGAAATTAAATtACAAACGTACTTAACACCACACTTCATTtCATTTGAAATTGTATACGAaGCAATGGATTTAATCGATGATATTGAGgACGAAAATaGCACGATGAAaCCAaGAGAAATCGCTGACaGATTGATGGAtATGGTTGTAAAAaTTtaCGATAAccccAaTTCACAGTTAAaGACCTAAAaGAaCGtATGCAtGCACCTGATGGAATGAaTGCACTTCGTGAACAAGTGATTTtCATTACTCAAGGTCAGCAAACTGAGGAAACTAGAAATTTTATCCAGAACATGAAATAAaGCCTGAAGATTTAACATATAAAGCAATGTTGAAAAATATGGATACTCTCATGATGGACTTAATTGAAAATGGTAAAGACGCTAACGAaGTTTTAAAAATGCCATTTCATTATGTACTTTCCATATATCAAAATAAAAACAATGACATTTCTGAAGAAAAAGCAGAGGCTTTAATTGATGCGTTTTAACCTTAACCGTTTGGTTAGGGTTATTTTTTtGAACTTTTTTAGAAAGGAGGTAAAAAaTGGgAGAAAGAATAAAAGGTTTATCTATAGGTTTGGATTTAGATGCAGCAAATTTAAATAGATCATTTGCAGAAATCAAACGAAACTTTAAAACTTTAAATTCTGACTTAAAATTAACCGGTAACAACTTCAAATATaCCGAAAAATCAACTCATAGTTACAAACAAAGGATTAAAGAACTtGATGGAACTATCACAGGTTATAAGAAAAACGTTGATGATTTAGCCAAGCAATATGGCAAGGTATCTCAAGAACAGGGCGAAAACAGCGCGGAAGCTCAAAAATTACGACAAGAaTaTAACAAACAAGCAAATGAGCTGAATTTTTtAGAAAAaGAaCTAGAAAAAaCAaCAACTGAGTTTGAAGAGTtCAAAAAaGCTCAAGTTGAAGCTCAAAGAATGGCAGAAAGTGGCTGGGgAAAAaCCaGTAAAGTTTTtGAAAGTATGGGACCTAAATTAaCAAAAATGGGTGATGGTTTAAAATCCATTGGTAAAGGTTTGATGATTGGTGTAACTGCACCTGTTTtAGGTATTGCAGCAGCATCAGGAAAaGCTTTTGCAGAAGTTGATAAAGGTTTAGATACAGTtACCCAAGCAACAGGAGCAACCGGCGGAGAGCTTAAGAAGTTGCAGAATTCATTTAAAGATGTTTATGGCAACTTTCCAGCAGACGCTGAGACTGTAGGCGGTGTTTtAGGGGAAGTTAACACAAGGTTAGGTTtCACTGGCAAaGAACTTGAGAGTGCCACAGAGTCATtCTTGAAATTTAGTCACATAACAGGTTCTGACGGCGTACAAGCCGTtCAATTAATTACGCGTGCAATGGGtGATGCAGGTATTGAAGCTGATGaGTATCAAAGTGTACTTGATAT

>NRS19contig00006 [organism=Staphylococcus aureus]

ATTACTGCTTtGTATTCGTCAGTTTGCGTAACGTCAACGTGAGTTATTTtCAACTtaTTAATTTtGTTTtGTATTCTTGCTGAACGCTCTTCTGCTTCGTTGATTTtAaTTTGTAAaTTATTGTTGTCATCCTCTAaTTTCTCGATAATTGGCTTtATTTtCTTGCCcTCTGAAATAAtGTGATTGATAGATGTTTGTATTGTTTCTAATTCTTTCGATTtGTTTGCATTGAaTTTCTGCAaTGCTTTTtCTCTTACCTCACTCACTTGTTCAGCTGGTAACTGTTGACCACAACAACTACATACATTGTCATCAAGATATTCAAATTTTTGATTTTTAGCTTTTTCTAAATCACTTTTTAATCCTTTATGATTTTCTAATAATTGATTACGTCGATTTTCTTCATGTGTAATTTGTtGTTtGTTTTGCTTTAATCTTGTTTTAAGATTCGCAACCGTTCCATTTTCAACGTGTAGCTCATTTGTTAAAGCATGTATTTTGTTCTCATTACTGGCGCTATTATTAGCTTCTATGCGCTTCAATTCTGATTGTTTATCAGCTAATTGGTTACGCAAATTAATTTCTTCTGCACCGTTTTGAATATCtATACGCTCATTTTCAAGTTGCTCAATTTCTtGTTTtATGATTGtGtGTCTATCATTATCGAATTCCGGTACATCCTGCTTATTTTGTtGCGTTTGGTtAATACGTATCGGAATATCTTTGATATCTTTGTTAATCTGTTTTAtCTTGTCTGTAaGAaTCTTTTTCTTtGTTTCAATTTCGTGATCTCCAAGAATATTATTTAGTtCTTTAAAAaTCATCATTTGTTTTAATGACATCCTCATCATTGATTGGTTTAGCGATTtCAAACAACAAACTtCTtCGTTTCTTCCAATCtaGTAAGTtAAaTGCTtGaGGgTTCgTAATtAACTtGAATaCATCTTcaTCAaTCAGTTCaTCAATaCGAGCTTTATAaTCcTTTACTTTtaTtgATTCATCATTGATaTaTTGTTTCTtCGTtCGACTTCGTGAGTATtCCTtGCGATTCGTTTtttGATTTATtGTGTATTtAGGATGTGACTCTTTttAAAAGtCGTAaTTTTCCGTCGATTtCAAATTCTGCGAAAaCaGTCGGAATTAaCTCATAATTTCTtCGTTTTtCGTTTAAAGGTACAGGGTTAAATGATTTGGTTGAaCcGTctAAaCCcTtaTCGAAAAGCAGCCATTGTAATGCGGTTGCTGTtGTAGTCTTgCcaGtcGCATTATTGCCGTAtATTTTTGCATCTTtaCcGtcAAAGTtAAAgcTTttCTtCTTTGATTCCAGCAAAGTTtcGATATAGTTAACTtATTtATTTTCATATCTTTCCTCATGCtCCTTTTTTAATCTTCCGATGACCTCTTAGCACCTCGATAaTtAAATTTTTtATTCGTTCATGGCTGTCTGGATTGATTTCATGTATCTGCACAAGC

>NRS19contig00007 [organism=Staphylococcus aureus]

cTATATaTCCTCAaCTGTGTATcTGgATATTGTCCTtGAgCACCAAaTAAATCATAaCcAACCgAaTCACcAGAAGAaGGgTGAaactCAaTtAatCctCTTtGtATTTTtCcATTAAaTGCATCTGTGTTATATAAATTATAtGTTTCATGTAAATAATGTCTTGACTGAAGGTCTAGCTCCTGAACAGTTACTTCTTTTTtATTAGTTTTAACCGTTCCCAAAGGTACTGTATTTTGTTTACCATCTATCCATAAGTTAATCGGTACTTTCTTTTCTTCCTCCAATTGATTATTATCATGTAACGTTACACCGCCATACATaCAAGCTGTTTTGAATGGTGTACCCCCAGTACATTGATAaCCATAATAAACACCATATAAATCCACTTTTTtCCCTTTGTATATATTGGCGGTGTCTTTTGAACCAAGGTCTACTAATAAATCATTATACCATTGATGACCTGTAAAAAAaTCATTAAACAATATAGTGTTCTTTAAAAaTTGATCATTACTCTCTTTATTTTCAATTATAGCACTTCCATTATGATAATAGGTTTGTCTAaGATTGCTTAAaGCAGTTCCCTGCAaTTCAGACTTTTTTtGCAAAtCTTTtCCATTTATTTCTTCGCTTTTCTCGCTACAATTTACAAATGGACTTGTtATCAACGTTAGGGCAATAAATGAAAGTAGTATAAaTGTTgTTTTTTtATTttAcTcACCTCTAAaGCATAATTCCAtATAAaCTTGTTATTATCCGAAATATTTGTATAATAGATTATTTTTGTTGAAGTAATTATTAGTtATATACAATAATTATGgAATGATCATTATTTACTtATACATACATATATtACTATAtcTTTTTTATACTTGTCTATAATATAATAAAAAGATTTCATTTTTACTCTATTATACATTTCAGATATCTTACTTACTTGGTATTGACAACACTTCCCAACTTCACTTGCAGTATGCTTCCTGTCTTTTGaGCTGTGTAATaCCATGTACGTACcTCTTTtCAATCAAAATAAAaaGCCAgtGCCgATGcAcTgaCCAAAAacATtATTTACaTTTAcGACcATaCAAatAACATgaTAACCAtCTTGCCcAacTCaTTAtGtCcACCTCCCTTAAGGtAATAACGCaGtAaTtGAtG

>NRS19contig00008 [organism=Staphylococcus aureus]

GTGATTTGgACATtAAATTTCTtCATTTTCCTGcTCCTCCTCATATTTATAGACCACTTGACCCGTCATAATCCCTACTGCCTCATCAAGACCAATATCTTCTTTGAGTGCATCTTGCATAGCATTAGGTAAACCCTTAAGTATTTCATCAAaCGCTTGCGCTTTCTTATATACGTCCTCAACcTCTTTTAGTAATCCCTCTGTGTCATTACCGTTATAGgCACTAGCACtgATAATTGATTGTTCAATTTGTTCGCGGTTATTCATCATTTCCATCTCCTCTAAAATAAAGTTAGTTGCTTCTGTTTCTCGTATTCCAAACCATGTTGCTTTATATATATTTCTAGCTCTTcGgCTGTATCAAAcGTCTtcTTtACgCCTTGCCAACCTGGtACGATATGCCcGTGAAAGTAaTAATtGCCgTTTgCTACATGGATATGTGCCACTCGTTCGTTATCTTGATACAGATATCTCTTAGATCCAAaGAATTGATTtAGGTATTCTTTgCGTGCGTTATCTGTCATGATCTACTTCTTAACTTTCACGAATATGTCGTTTTCCATCAGGTAGCACGCATAACGTCCTCTTGGATGTTTCTGAGGCACATTAAACAAGTGTGGCTTCTTTCTTCTtAGCTCAGCCTCTCTCTTtCGCTTTCTTTCCAATTTGCGTtCgaGTCTAGCTtGTTCCAGTCTTTCtaTtGTTTTCTTTCTCTGTACTCGCTTAAACGCGTACCTTCTGGTGCGTCCaTTGCTTCaTGTAGTTCCCAACcGTCTTTTACTCTCTTAGAAaCCATTCCAGCGgTTATACCGTGACTTTCTATTAATTCCATTTCAAATTTaCTGAACCTATAAGGTTTATCATTTATTGTTACAATCCTTGCTTTTCTCGCCaTTTTATCCACCTCTTATATTTCTTCTATTCGTATGATTATTTTGGGCTCAATTCCAtAACGCTTTGAGCTAGTTaTTTCTGTAATTTGGTtATCGTCTTtCCATACaTGGCCaTTACAAGCATCTAATACCGTTTtAATtAAGTTGTCGATAtCCGGcTTAGTCACTTTATaCTGCCCaaCcATTTcGcTTTTCTTTTtCTtCGACCATGaTTTAAGCAaTGGAAAAtACTAGGtGt

>NRS19contig00009 [organism=Staphylococcus aureus]

ACACCTAGTATATATTTGAATTTATCTTAAATtCCAAATTAACTGCACAGTGCCTTTTAGTATCATCACAATACCGTCCCAAACACCTCGCCAATTACCAGTGAATAAACTTGAAAAGAACTTAATAAAGCCAAGTATGATATTTAAAGCACCTTGTATTACTCCTTTTATATTCTCCCAAGTACTGACAATCAAGGCTTTAACCGCCGGCCAAATAAATTGCATCACTTGCCAAATCGCAAACATGATTGGTTTAATTACAAAATTTAAGATAAATtCAAATATAGCTTTGATAAAATTGCATATATTTTGAAGCGCTTGAaCAaTAGAAATtCcGTTTtCATTAAaGAATCCATTAaTTTGACTCCAAaTATCTTTAGCGAAaTCAACGATTGCTGAAACCGCTTGTTTAAaGACGTTTTTAACGGAATCAATGAAaGGTTGGATAAATTGAATGAAATTAcTAAACGTTTgTTTAACACTGTtAATTGCACCATTAaCAAAaTTTCTGAATGTTtCAGATTtCTTATAAGCTATtGTAAATGCGACTGCTAAACCAGCCAGTACACCTAACACGATACCAATTGGACCAGTTAATGCTGTGAAGACTGTtCCTAAAATAGGCACTTTAGTTGATAAAAAaCTAATCAATCCGCCAGCCTTTACAATACTAGCTAATAATGGAGCTAATACAGTTACTGCGTTGCCAATTGTGCTTATGAATGCACCTAATCCAAAAaCTACAGGACCAATTGCAGCAGCAATACCACCGAATATAACAATCGACCTTTTAGATCCATCACTTAAACTTGAAAACCAATCAACTGCTACAGATAGCTTTTTGATTAATtCTTCCATGACTGGAGCAAACGCACTTTCAATAGAAGCCCATACATCAGCACCTACTAATTTAAGTTtATtCATTGCTACTTtAAaTCTTTCGgAGCCACTTtCAGAATCTTTAAaTGTCTGaTtGACcGTTCCTTGCGAaTcTTCGATaGTTTTtAaGAaCTCTTGGTAaCTAAaGCGACcgCcTTTAATAGCATCTGCTAAATCAGGACcTGCTTTTGCACCAAATGCTtCAaTCGCtAAaCTtGTTGCGCTaGCTATATCCgtGT

>NRS19contig00010 [organism=Staphylococcus aureus]

aTCCTtGTTcaCCCAAGCATGTCACtGGGtGTTTTTtccTTtGATAGAGAGCATAGTTTTCATACTAcTCCCCcGTAGTATATATGACTTTAGCATTCCCGTATAAtAGTTtACGGGGTGCTTTTtATGTTATAATTAATTGTATATAGTAGGAGTGAACTATATAGCCTGTTAAGTGGCCTAGTAaCCTAACACTTATCCTGGCAATTGATACCCTTTTtGCCCTTCACTCGATAAATATATCTCAACAACATAGAAATATTACAGTCGCTACACCGCATCTTAAATGGTGTGGTTATTTTtATTGGAAGTGTGTATCAGGTATCAGTAATGTTAAAaCACCAGCTAAAAaTGAAAaGAATTCACCAGTGCCAGCAGGTTATACACTCGATAAAAaCAATGACCGTATAAAAaGaGACTGGTTATTACACagTTGCCAATGTTAAAGGTAaTAaCGTGAGGGATGGCTATTCAACTAATTCAAGAATTACAGGTGTATTACCCAATAACGCAACGATCAAATaTGACGgCGCATATTGCaTTAATGGcTATAGATGGATTaCTtATaTTGCtAaTaGTGGACAaCGTcGTTAtaTAGCgACAGGAGAGGtAGACAAGGCAGGTAATAGAATAAGCGGTTTTGGTAAGTTTAGTGCAGTTTGATAATTAGATATATAAAGGTTtGGCAAGTTATGAAATGTCTGCCAAACCTTTATATAAAAAAGAAATATCTACCTTTTAATTTATGTAACTACTATTAGTATGCATATTCATTAGTTTTACCAGGACCATTAATTACATAAGATGATTTAGACTCtCCTTTTTtAAAGAaGTATGTTTTAtACATTTtACCTAGTAACTCAaCATTTTTtCTATCTTCAGCAAGTGGTGTATTCaGATATaCTGTATAGTAACCTTTATTTtCAGTTAAAaTAaCCATTTTTtCAAATTGAGCAGTATTTTTTGtGCCTTtctCTAAATAATTTCTCAAACGTTCATCtAATTTTCCTAgCGTTGTAGGAAGACCACtaTTTTtAAATGATtCTTTATAAGCTTTTtCTTtCTCTAACATTTTCTTATTtGATTCtATTtCTT

>NRS19contig00011 [organism=Staphylococcus aureus]

GGACAATATAACTATATGCACACTCATCTCCGTCAGTATAAGTTGTAGGTAttgggtCGTCGATATCGTCAATAACAataaaggtacatcagtatcttttacATTAgggTAtttATTGAACTTAATATTATTGATATTTACGTGCTCTCTAATAATTCTGTCTTGACTAATCACTTCATGAACTTTGTACAAAATATCAATCACAATTTTTtCAACTCCCTTTTtAACGTTTCAAAATACTTATTTtGACCTTGTCTTATTGCTCTATTAATCCCACCCATAGCTTTAGGTTTtACAAATTTTCCTGACTTTTTCTCAACATGACCATTTTCAATTAAatGTACTATtCTAAATCGTTcAAAaGGCCcGCGCcACCTAATTGTAACAGTACGTTTtCCcTTtATCCATTCAGGTTCAGTACgaCCAATCTCACTAATCAGCGcTCCTGAGTCTTCTGAaGgTTTGAGTtGTTTTTTATTtCTTCAACAaTTACCTTAGCACCAGCTATTAACGCCTTATCTTGAACTTTTACCATCtCTTTTATGCCAAAATGTTTTTCTAATTCTCTTTCTAATGCTTTATCACCTGTCACTTTCACACTCATGAACTaTATCCTCCACGAATCATAATAAAGTCTTTATTATCCAAATCTGGTGATACTTGCTTTATATTCAAACGATTTTtGAAATATCTTGATTCAATTTCAAGATAATGTTCTtCACTGGGTAAATAATCACCTtGCGgATCACGAAtATACAATTTAATGTCATTTTGCGTtCCGTTTGAGATaGCTTGTTCTAATTCACGTAACCAGACACCaTCAATACTCGCCcAACAGCTATATAATAATTTTCTtCTTTTtCTCCAGCTTCTGGACCATTATTTTCAGTATACTTATAAAAATGAACACGCGTATTTAAACGTTTAGTTGTAATTCTAggtttctTaaaCACtttCTTCATCTTCTGATACCTCCAT

>NRS19contig00012 [organism=Staphylococcus aureus]

AAATAAATATATGTGAATAATCCTGCAACACCCGAACTACAAAACAACAaTATTATTTCCAAATTCTCACCTACTTTTTATTTTATTATatCAcATTTAGTACCTAGTACTAAaTattGGGTaGCCCgCCTACCCTTATTATTTTTTGCCAATTTTGAGGAGGGAACGCATGAAAACACGTTGTTACGATGGTAAAAAATGGCAATATGAATTTAaGCATGAAGGAAAAaGaTACCGTAAGAAAGGTTTtAGAACAaaGCGTGAaGCTAATTCtGCTGGACTAGACAaGTTAAaTGAGTTAAGAaGTGGTTTTAATATAGATAACTATATAACTCTTGAAGAATACTTCGAAAaTTGGATTAAAaCATATAAACAACCTGTTGTTAAAGAAAaTACCTACCGTCATTATAGAAATGCATTACAACATATACAAAAaCATAAAaTAGGTAAAaTGGAGTTATCAAAGaTAAATAGACAAGTTTATCAGAAATTCATAAACGACTATTCAAAAGAACACGCAAAaGAAaCTATAAGAAAAaCAAACGGTGCTATTCGGTCAGCTTTAGATGACGCATTATATGATGGACTTATTTTTAAAAaCCCCGCTTATAAAGTTAATTATAAAGCCGGAAAaCCTACGAAGTCAGAACAAGAAAAaTTCATCTCGGTAACTGAATATGAAATACTAAAAGATCACGTCAGAAaGAAGAGAACTCGTTCATCATTAGCGCTATTCATAATGATTTGTACGGGTTGTCGTGTCAGTGGTGCAaGAAaTATAAaGATTGAGCATATCAACCAAGTGAAAAaCACTATATTTATTGACGAGCGAAAAACCGaTACTTCCCCtAGATaTAtCAGTATCGCTAAATCTGATATGAAACACATTATGgAcGTCATAAGtACATTtGCAaTtaGCTATGaTGGTTAcaTTTTCAAAGAAGCCGGaTCTATAaTTAaCCTtcAGGCTATCAAT

>NRS19contig00013 [organism=Staphylococcus aureus]

TGTTATATCGAatAACcGATTtAGTtgcAcGgTTTATtATTtCTGTTTtACtAACTTCATAaGGaGCTTTTGGCGTTTtACCCGTCTTATTtCGTTTGTTATCCATTAAAACGGTTCtGATTTCATCAAACGATGTTTTTGCTTGATCTtCACTATTAGCAACAATGGAGATGTGATATTCTTtAACTCCGTGTAAGGgCGTAGAAAGAAAATCACTAATAGCACTTATTAGACCGTTTTTCCCGCCTCCACGTCCCATGAAAATAGCAAATTCTGTAAAGAAAGCTTCATCTGTATTTTtATCTATAAGAAATATATTAGCTATGATAAACCTTTGAAATGGTAATGTTGGAAAATACCATTTTtCAATAAATTTGATACAATCCTCGATTTtCtGTTCATCAAAATaTACATCATCTCGTGAATATATATGTGTTTGTAGATAATTAAAGAGATCAATTCTTTCTTtATTTAAAATTATCTTtCCTTGTTTCCACAAATTTATATATTCATCAACGTATTTATTACTAATCATAGGTAATCATCAGATGGCGTTTCTGTGTCTTCTTTCTCTTCGGGCAaTAAATCCGATAATtGTTTGATtATTTTTTGATATGCAGCATCTCTAGCATTAAATAGTTTGGCTACTGGtCTTTCCCTTTCATATGGTGGCGCCTTTTCAGATTGAGTAAATAAATCATAaTCACCTTTTtCTTTTATGTcTTCCCACATGTAATCAAGCATTACACGTAGCCTTGCTGCTTGAATAATtAAaCCATCAACTACTTTTAATTTATTGCTAGGTATGTCTTTATATAATACTTGTAGCCTTTCTTTTtCTTTAAGCACTAAATTTTCATCGACTATAATCTCCATTTCATCACCTGCCTTAAAATGGTTATAAGAGGGGGGgTTATACATGGATTTTtAAAATTATCGCGAAGTTTAC

>NRS19contig00014 [organism=Staphylococcus aureus]

aacccaacatacaaaacaacatcaaaccaaacacaaacaataatgataataaagataataatgaaaagaatgTGAaTAaTGAGAAGAAGAAGACAaCCGCCTTCGACTTCTTCcAAGATAACGGATTCGGTTTCATAACTTCTTACAATTTAGACGATTTAAATTATTATCTTGATTCATTTGAAAATGATTCAGATGAAATAGTTACCGCATCACTTAAAATCGCTAAAGACAGAAACAAAGTTACTTGGGGATATGCTAAAAGCATTTtGAATACATGGCTTAATGCAAACTtGAAATCTATtGAACAaGtACGTGCATTtGAAAAGCAACAaCTtgAAAGCAAAAaCAAAaTtATAAACCTTtCGTtAAACAATCAAAaGAAAAAaCACCcAAaTGGCTCACAGACAGCACGAGAGAAACGAAAACGCcGGAAGTagATGAAAaCcTTGAGAAAGACAGAGAAGCTTTTATtAAGCGTCTAAATAGCAAATGGGAGTGATTGAAAATGGATGCATTTGATAAATACTATCTATTTGATCATGACGGCAACAAAATGTTTTCAGTTACACCACATTTTAAAGATGGTCGGCATTTAGTTGTTGGAATAAAAGAAACAAAATTTAATGGTCGTCGTTGGTATTTAGACGATTATGAATTAAATACACTTATTGATAATGAACAAATGGAGTTAGGACACcAAACAAgCTTATTTGAATATATATGaGggATTACAtGgAGATAGAAATTAAaTTTAATGAAgTGTTtAaTGCGCcGATGGGgTCGCcTCGtCCACGCTTTCGtAaTACaGGTAgaTTTGTTCAAaCTtACAtGCCAaCGTCTTACACAAAGCATAAaGCGTATATACAAGGgCAAAtGCCTAAGTtAAAtCTAGAGCgCGcACTAAAaatCgAATTAG

>NRS19contig00016 [organism=Staphylococcus aureus]

CGACGTCGCCAGGCAaTGCTtGAAACGACACAGTaTTTTCGTAAACGGTtGCctCGTtAGTGAAaTCATtCCAtGTTGGAaTGTCTGCAGCGCCCACaCcTTTCAaCCTGTGaTtAAATAaGTAAAGCCAATaTtGGTTAGCAGtATCGAAGCATTGACATCCAAATGCATTGTCTGGATTCCACGCCTTACCCTCtAAACTTTTAAGATAaCTAaTAGCTTGACTGTATGTCCTAaCCgACGGCATtGTTATCATCTCCGTTCACTTTAGGTGCGCcACCAGTTGaCTGAaTGCCAGcTTTtACTtCATAAATTTTTtGTTGCCCTTTCTTAGATGCGTGAGTAAAGTTGTTATTCTTCCACCACGTCCAAATTGAAACAaTCCCAGTAACGACTGTgCTTATAAACACTTCGTCAACTGGGATTGGAGAAATATGTTTGATTGCTAAAAACTGATTGATCCATGCGACTATTAATAAAATTGTTCTTACGATTGTACCGATATCCATTTGTTTACTCcTTTTATCCAAAATAAAAAaCGaCTAAAAAaTTAGTCGTTTAAAATtATTCAaTGGTCAATGTCGGAGATCCTGAATAAACATCACTTATAGTGACATACAaCAtCCCTGAaGgaTtACTAAAGTtGATATTTttACTtGCAAcTCCGCTATTGACTCCTGATATTCCtAATtCACTTGACCcTAAaTTagTTTGCGAAATCcTCATTATACCGcTACGTAcATTTTCtATTGTCACcTGATAaCTTTtATTaGGTtCAaCTCCATTTATtGTCCATTTtGCTgTtGAaTcTtctATGCTAtCcGGATATTTaTTTTTA

>NRS19contig00019 [organism=Staphylococcus aureus]

ACTTTTGCTTgTGATACCGCTAAACCTTTACCTAAGCTACTACCTCCACCGTGTCCACTTACGAATGAAGTTACAGAGTCCCAAGCTGATGAAaTCGCATCGCCTACCGCGCTGACTACTTTGTGCGCAGCATTGGCTACACCCTCAGCTACTTTGCCGATTAATTCCGCTCCGGCATTTAAGAAATCACTGAAGAAACTTTTAAtCTTACCAAGTGCATCACTCATACCGTCACCTACATTTgAGGCAACtCTTTtAAACCCATCAGCTACTTTACTCGCGAAaCTTGTAaCTGTATTCCAAATGTTAGAAaCCCATTCAGAACCTTTTGTGATAATAAAGTTTAGTGCTTGCCCCATTTTTtCAGCTACACTCGAAGCCACTCGACTGAACCAACTTGTAACAGTGTTCCAAATACTGCTAACAAAATTAGTGATTGTACTCCATATCTGTGACCAACTTGTACCAAACATAGAAAGTGTTCGATTCATTACGCCAGTTAAAAaGCCGATAATTGACTCCCAAaCTGATTGCATGTATTGCCAAATCGTATCAAGCACATTGGTAACCGTaGTTTTAaTAGTCTCCCAAGCACCTGAGAAGTCGCCAGTAAGcAACTGAaTTAAAGCAGTGAAcAAACctACTATGATTTGGACTGCTACGGATATCACTGTTCCTATGgCTTGgAACGCAATtgTAaTtAAaGTCcACAAACCTtGTATGATATTCATAACGTTtgTAATGAT

>NRS19contig00020 [organism=Staphylococcus aureus]

ATATGGCACAAGCTATATTTACAGGAATTATTAACTCAAGCAACTTAGATGCCAATGATATATATTTAACAAATAAATCTAATGAACAAGCTTTAAAaGCATTCGCTGAAAAaCTAGGTGTTAACTATAGTtATGATGATGCGACATTATTAAAAGATGCAGATTATGTTTTTttAGGTACCAAACCGCATGACTTTGATGCTCTAGCAACaCGCATCAAACCACATATCACAAAAGaCAATTGCTTCATTTCAATTaTGGCAGGTATTCCGATTGATTatATTAAACaCAATtAGAAtGCCAAAATCCAGTTGCtAGAaTTATGCCAAAcACAAATgCgCAaGTtGGaCAcTCAGTtACTGgCATtAGTTTTtcAAACAACTTTGACCCTAAATCTAAAGATGAAATTAACGATTTAGTtAAAGCATTtGGTTCTGTAATTGAAGTATCAGaGGATCATTtACATCAAGTAACAGCTATCACCGGAAGCGGCCCAGCATTTTtATATCATGTATTCGAGCAATACGTTAAAGCTGGTACGAAaCTTGGtCTAGAAAAaGAaCAAGTTGAAGAATCTATACGCAACCTTATTATAGGTACAAGTAAGATGATTGAACGTTCAGATTTGAGCATGGCTcAaTtaGAAAaataTtACctctAAAGgTGgTaCGACACAAGCtGgCcTTgATACATTGTCACAaTAtGATTTAGTATCTATTTTCGAAGATtGTCTAAATGCT

>NRS19contig00021 [organism=Staphylococcus aureus]

TCACAACAAATTcAATTGaTAGTAAATGAACAaGaTAAAAAATtAaGAatGGAGACTATaTAAAaTGACGAaTGAATTaCTaTtAAAAAaCaTAAAAtGGGCGACAACGTTCTATCTAGAGTTAAGACATTAGAAGCACAAGgAGATTTACAGTTtCCTGCAAaCTaTTCGCCTgAGAaTGCAATgAAgTCAGCAATGTtACAaCTGcAaGAaTTAAaaGgATCTAAAAaaGATGGTTATAAACCAGCGCTGGAATTTGCAACTTCAACCAGCATAGCAAACGCCTTAATGGACATGGTTGTACAAGGTTTAAATCCTGCTAAGAATCAAGGCTATTTCATTATGTATGGCGATAAGGTTCAATTCCAAAGAaGTTACCACGGAACAATGGCAGTAACTAAACGTGTAGCAGGCGCAGAAGAAATTAATGCAGAAGTCATATTTGAAGGTGACGAAGTTAAGTATAAAACTAAAAaCGGAAAAaTTGTTGAACTTGAACATACACAGTCTTttGGTAACAGAAACACACAAAACaTTATCGGTGCATATGCAACAGTTGTATTTAAAGATGAAAGTAGAAATTACACTGAAaTCATGACATTTGAAGAGATtGAaGAaGCgTGGAaGCAatCaCAAATGGTTTATAACGGTGtATTTAAAgAaGAcGGtACAcACAgAagattccctcaagaaatggctaaaaagactgtaataaaccgtgcatgtaaaaaga

>NRS19contig00022 [organism=Staphylococcus aureus]

GTCAAGGAAGGTTTTCACTTTAgATCGtGACCAGTTAAAAaGGTCAGACAAGGTCAAAATCGATGTTAATCTTTGTCCTCTTTCTACGGTTACAATTtGGTTTCCAATAGGCACTTTTGCCTTTGAATGATTCGCTTCCATGAGTAAATATATCCATGCTTCAAACTTTGAAAATGTTCTCTTTtCTTTAAATAGCCAATGATTTTGAATTGAGCGATCAATACTTATCCAACCAGTCATATACACACCTCACTTTCAAACCGGTTAAaTTAGAATGGTAAATCATTGTCATCTATTTCAATCGGACCATTtGCATTCGCAAACGGATTATCTTTTACTGGTTTGTTATTTGAATATTGCGATTGTCCACGTGTTTGTTGTACTTGTTGTTGATATAAATCTTGTTGAGTGTCATTTGAGTTTTTCGGTtCTAAAAaTTGAATaCTATCAGCAaTAACTTCCGTAACGTaTACACGTTGACCTTCCTtATTTTCATAGTTCCGCGTTTGtAACCTACCATCTACGCCCGCCAACGaTCCTTTAGATAGGtATTTATtAACGTTCTCTGCTTGTTTTTAAATACGATGAtATTAATAAaGTCTGCCTCGcGCTCtCCTtGTgCATTCGTAAATGTGCGGTtAACTGCTAATGTGAaTGATGCTACaTTTACaCCACTTTGAGTGgTTCTTAaTTCTGGGTCTCTAGTTAAACGACCAACATaCTAGGTGT

>NRS19contig00024 [organism=Staphylococcus aureus]

TAtGAGATTCtCTTAGTtCaTTATGATgTTGCTTAtGTTCCTCTCtAAGTTCAAGCACATGATCAGCtGTTtCATTTGCTAGtATTTCAACaTCATCAACACGTTCAaCTAATtCAgAAAGCTcTTTTttATTttCtGAATatCATCCAAAACTaCACCtAcTTTCtAAGAAaGCTAtGAGCGTAaTGCTCaTAACTTAGTATAGTTACATtGTTTCGCTATCAACTGATTTAtCAGATgaCAAGTCAGTTCtATCCaCAACTtCTTTCacAACTTTcACACcGTTTTGaTTGCCTGTtAATTGATATAAaaGaTTtAAAGTTTCAGCAATCTTTTtAGCGTTTtCCTCAGATTTAAAaTCTTGTGCATAaCTTGCTGAATCGGACGTTGTAAAACTTCCTGTGAAATCTTGATATACTACACGTTCTGTACCTtCTTTGTCGATTTGTACTAAATAAACCTTTCTGTATTGTTGATAATTTCTTTTGCCATAATTAAATGACCTCCTTAAATTTTTGTATAAAAATAGTGCTAAGGATTACTCTTCCTCAGCACATTGTTGATTTTCTTTATTTTCTTGTATATACGCTTTtAACATCGCGTTTtCTTGTGTTAACCTCATAATTTCCTGTGATAAATAATGAATTGTATATTCAGGATTAGCTTGTAATCCTTGTTTGTTATCCTTC

>NRS19contig00025 [organism=Staphylococcus aureus]

GaCCTtGTTGTCTGTATAaGCATTAGCTTtCTTTTCAGCGTTTCTAGCCTTTAGTTCTGCGTTTtGTTTTGCCTCTTCAaGTTTAGCTTGAGCATCTTgTATAGCGCGTTGCTCTtCTTCCGAAaTTTtACcATCAGCaTACGCTTGCGATTCCTTCtCTTTAAGATCATCTTGAGCATCAATGTATGATTTTAAAGCTTCTTGCGCTTCTTGATTTGCTtGTTCAaTaCTTGCTTtAATCTCAGGATTATTGGaCAAATCACTTAACTGGTCATCAGTATATTGTTTTtGTTCTTCCAATCCGTTTCGATATTCGTTTAACGTAACTTTATCTTTGATTTCaCCTTTTAAAGTCGTTCTCTCAGCTTCAGCAGTATCTAAACGTTCAACAATACCGTCTTTGTCTGTTTTATAGTCCGATGTTTTtACATAGTCACGTAATTGTTCTTTTGTGGATTCTCTAGCTGCTTCAATAGCTGATTTAACAACATTAGGTTCTCCGACTAACTGCAAATCTTCATTCACCGTTAAaCCAAATTTTGTTGCTATTATTTCCAACGCTTCTTTATATTTTtCATCAGTGTATTGTGACTGTAATAATTTAAATCTATCTGAAATGGCCATTTtGACATCTTCTACATCTGTAtAAACaTCTTGTAaTTtCTTTCTAtACTC

>NRS19contig00028 [organism=Staphylococcus aureus]

GTTGCATAATCAtttCtgggCTATTCCAAGCTTTTTCtAACTTGGATGAAaTACTCTCTAAAATCAAAACCcTTTtCTGTACCTGACATCATCGCAACATGTTTAGCTACATCAAGTGTTAAAGCATAaTCTTCTAGTTGTCTTACAGCTCCGTTATTAACAACcGTACTTGTAAGTACACTTGTAAAATCTCTGTTTtCTTTAAAaTGCTTTAAGTTAATTTCTGCCCAAGCGCTAAAaCGTTTTtGACTTCCAAAGCCTTGTATAACTCTCTTGCACTGATTGCGATTTCTCCATTTTCTTTTtCTTGTATGTTGAACATTTCGCCGATGTTCGATTTTGTTTGTAATGCTTGCATTTGTAGTTCCTCCTTTATTCGAAATCATCGATTGACAAAGTTTCAATCCGTTTTtGGTAACGATATAAATAGAAGTTCTTTAACATGTCATACATTCTGCTAGCTTCATCGTATTCACTCTCTTTtAAATCAGAATTAAGCGTTACACCAAAAGCTGATAATGTAAGTTTtCTAATGTGGTCATGAATCTCACTAGCGTATGCTTTGTAATTTTCATAACATCCAATtCCGTGTTGATATTtCTTTAAAGATAATGGATGTCCTAaGCCGAGATTGTCAGCACCTCTtAAACGTATACTAGGTGT

>NRS19contig00030 [organism=Staphylococcus aureus]

ATCTAGATAAAAaTGAGGCACAAGCTAGCACAAGCTTGCCAaCATCGAATGAATATCAAAACGAAAAGTTAGCTAATGAATTAAAATCGTTATTAGATGAACTAAATGTTAATGAATTAGCTACTGGAAGTTTAAACACTTATTATAAGCGAACTATAAAAATTTCAGGTCTAAAAGCAATGTATGCTCTTAAGTCAAAAGACTTTAAGAAAATGTCAGAAGCAAAATATCAACTTCAAAAGaTTTATAaCGAAATTGACGAAGCACTAAAAaGTAAaTATTAAAAAAaCCACCCTTTtAcGGGTGGTTTtAATTTTCTAGATAATATAAAAGTGTTCATAAaTAAAaCAGTATAGGCAAACAATAAaGTATTGAAAAAaGTAaGTTtAATATGAAAATTGTtAAATGAACGACATCTTTtGTTTTtATAAATATCAAGAAAaTAATCAAACTCAAAATAAaTAACGTAACTGTAGTCATAGgCGTCCATACATAATCAGCATTAGTCATTAAGAaTGGTGCAGCCATtaTGAAAAAaTTtaTAATGCAGaTGAAATaGaCAaTTAGACTATAAATtAGGtAAATAaCAaTaCACaCCCTTcAtAAaTAAATAAatAAaTAATTTAAATCCt

>NRS19contig00033 [organism=Staphylococcus aureus]

CATAgCGaCATTCATATTTtATAAGGAATACTTTTATGAAGAaTAAAAAAaCTGCTACTTGCGCcAACAAGTAaCAGTatCAAgtACTtAAGaaaaatttCAAGTTAAATATAAAACGAAAcAAGGAGGAAGTCAActaTGACTAAAAAttATAAAGACATgACtCAGGacGAAaTAAAAGaCTtattATCTGAAAAagCGGAGAATtGTATGAATTAGCGAAAGAAaTTAAGGGAGAAAGTAAATTTGAtATTTTGCTTTtCTCATCAATAGGAGTTATCGACGGAGATTATTTAGCAGGTTCAAGTTCTGTGATTGGTCATACTTTtGATCTTGCTTacTTATTGGATAGCACTAAGaGTTATAAAGAtATTGTCAATGTTCTCcAAaTGTGTAAATCaCAAAAaattCtCGGTAtaGATGAcgaCAAGGAGgaCTAAAACAATGTATTACAAAACGGGTgACGTATGTCGAAAAATaTTtAAtGTAGATGGCTTtGATTTTCAATTAAGAGTTAAGAAGCGAGCATATAGtgtcgaaatagtcgttttagatcatgaaggaaattcaattgacgggctactagtttctgacgagaacgatctatacacag

>NRS19contig00034 [organism=Staphylococcus aureus]

GTAtGTTtAcGTCcTGTTGAATCTTtgAaTGTTGTGaCTAaGTATTGTGTCATtCcTCATAGcTCCCTTGAaCTtGTTTGAGCTtACTCaTAAAAAACaTTaCtAAAAAATGCtATTAaGATAtGCGtCTTTTGatGTTTAtAaGCAAATGtAGaTATCaTAAAGaTAGTAGCAAGCATTAACATTTCATATATGTTTGtGTGTATAGTCTTTTTACTCTTAAGAAAAATAATTGCTATGCGATAAAAGAGATAAACGCCAAACCCTATTAAAAATATTTCTAACATGTCGCTCACTTCCCCAAAACCTCCTTGACTCGATCTAAGATGTCTTTACACGTAtCCTTTTCCTGCGTCTGCTGTtCCATCTTGTCTTTCGTGGTtCCTTTtCATTTtCTTTTtGTATGCGTCAATGAGTTGGTCGATAGAATATAAGTtGTAAGCTATGTCTATCACTATAaCAaTTGCTTGTTGgTCGGGATAAAaTTCTTtGAATATtATCTGTGGTGTACTAACAACTGCGTCTTGAGCAAATTCTTTATCTTTAAAATTAAACATTTTGTGAAATTCTGTATCTTTAAAACTTGATTCAATCGCTTCTTTTATCT

>NRS19contig00035 [organism=Staphylococcus aureus]

GTGTAATAAATGTAGTTATTAAAATCTAATAAtCCGGATtGTTCTTCTACaTACTTTTTAGAATCATATATGTATGAAGTAAAGTGTTTAGAcAAATGTTTGATATCAGTaTTaCGAAAaTTATaTATTTCTTTTAATTtACTGTCATTTGAGATAACAACGATGCAaGGTTCTTCAAAAAAaGATTGATTTAGATAAAATATCGAAATCTtGTAATCGTCTTTtCTCATGAATGGGAAGGCTTCCGGATTACTACTAAACTGATAAaTGTATCTGTTTtCAACTACATATTTGTAACCTTCTAAAAAATTaCGCAAGTATTCTTTTAAAGTTTtaTtCTCTtCCATCCCTcATCCtCCTCaCGCCaCaCAAGCgCTATTAatCAataTCCAATAATTGTtGTTTTTtCTTATCGAACTCTTCCtGAGAAaTTACTCCGACATCTAATAaTTCTTtATATTttattaaTTcaTCAGCAACAGAAAAaaCTCATTTTTTcAGAaTtGGATGGTTTCATAGAACTTTCTCGAaTAGaGATTtGTtCTTGTATTGTTTCCgCCATTCTAGATACAGtGTTTTTtGATAtGCTTCcTATagCga

>NRS19contig00038 [organism=Staphylococcus aureus]

aaagtaaCtGTATTGATCTGATAAtaTTTTTTCATCTTGCTTtCtAGGTTTCATTTTACTACCTCCTaTAAAATAaCTTTtCCAACTAACCtcACACTTTCGTtAtCATAAAAaTaTAaatCTttATACtttttaTTTAAaGAAaCCAACGTTAATCTATTATCTTCTACATAAACTTTCTTTACGTAAGCATCTCCATTTATAATAAAGACGCCTATTtGTCCATCTTTGATAGTGTGAGATTTTtCAATGAaTATAaTTTGTCCGTTTtAAAtAACGgCTCCATTGAGTCTCCATTtACTTTtAAAGCTATATCATGTGCGGGgACATAACcTCTTACGAaTTCTTTTGAAATAGGCTCGTTATATAATCTTTCGCCAATaCcAGCTGACGCACAACCATATATATCCACTTCGgATTTTTCTTGAaTGTAaGAATTGAAaTCTACcAGATTATCACTGTCaTTaTTTtGTtcTtCtAATTGATtAGtcgCaTATTTTAGTACATtGcTTTGTCTTGgAGGCGTGagTTTACTGtATAtGGAaGTGATGTCGTtATTTTCAaC

>NRS19contig00042 [organism=Staphylococcus aureus]

ctaggTTaaaaGTAggtgataaagTaaaagttaaGAcgattggATAtagaatacacttttaaatttatatcCGGtcttatacgaaGtaaagAAggTaGataaaaaatgattaaGCaaaTaCTAAGATTATTATtCTTACTAGCGATGTATGAGTTAGGTAAGTATGTAACTGAGCAAGTATAtATTATGATGACGgCTAATGATGATGTAGaGgCGCCGAGTGATTACGtCTTTCGAGCGGAGGTAAGTGAGTGATGTGGATTACTATGACTATTGTATTTGCTATATTGCTATTAGTTTGTATCAGTaTTAATAGTGATCGTGCAAGGGAgaTACAAGCGCTCAGATATATGAATGATTATCTACTTGATGAAGTAGTTAAAACTAAAGGaTACAaCGGGTTAAAagAaTACAGGATTGAATTAAaGCGAaTGAATAACGATATtAAAAaGtAaTTTATATTATCGGAGGTATTGCATTGAATGATAAAGATTGAGAAACACGATATCAAAAAGCTTGAAGAATACATTCAGCACA

>NRS19contig00043 [organism=Staphylococcus aureus]

CTCCaCcATTtCCATCACCaTCTGGAAGATTTGAGGGATTCAATGAAATCTTTCCTCCTCCAAAAGGACTGCCAAACTCTGTAAaGTCACCACCTGGAAAaGTCCCATAAAAAATTAATAAAATAAATtGGTCTAAACTCTCATTTAAGTACAAtGTAGAGCCCAcACCATTTGCTGTtCCATCAAaaaTAaCCGAATACCTTTtATtAAaCTTGTCATCTGCGTATAaTTTAGCGTTACTTtCGGCCATATTAGCTTTtGATTGGGCACTTTGAACAGTTTCAAAAGGTGTATTGTAATCATTAAtAGCTAATTCTGACCACTCAGACCATGAACCCGCTTCTTTtCTTTTAACAAACACTTtATTtGTACCGTTCGGTCGATAaGTCATACGCTTGTAATCTGAAGTTACTACTAAATaTTCGACAGTACCGTTAGTACTAACATCTCTTGGATAATTTATAGCTTGCGAAACATAAATAAATTGGGTTGAATCACCTATTCTTTGTTCTGGATTATTAAAATCAAATCCAGT

>NRS19contig00045 [organism=Staphylococcus aureus]

GATAATTAAAaGCTAATTTGCTTAGCAATGTTACGGACATACTAGTGGTTTTGTTTGCGACTTTTTtAaCTtCTTtCcAAGTGTGATTGTCTCGGATATTATCTAAAAATTCATGCCCTGACCAAGTTATATCGTTAATTGTATAACCATAAATATgTCCATCTTCCCAACCGAATTTAACACTAACATACTTTGCTTCTTCCAGTTTTAATAATGCATACATTACAGTTTCAAAATCATATTTTCCAAATACAACATTATCTTTGAAATTGTATTCGGTGAGCGGTTCACCAATCTTTTtATtAGTTtCAATTTCtAACAAAaGaTGTCTAACACAATCATGATCTAATTTCATACTTATCACTACTttAGGTtGATAaCAaCATtATACACGAAAGGAAaGAtAGAAATGCCAcATATTTTAAACGTAACAGTTCCAATACCTGAAACaCACGTGCTTATcACAAAaGATGAaTATgAAGAGTtAATaGCttaCT

>NRS19contig00052 [organism=Staphylococcus aureus]

AGAAGAaTaTAaCgATTTaCcATTCAACCGTTCAAaTAAGCCcGAaTTCATGACTAAGCGAATGAATTTGCCTGAAGTTGACCTTGAAAAaGTAATAGCACCATGGAAAGAAATACTAGCGACTAATAGAGAGATACCAAATTtAGATAATCAAATGtGTATTGGTGGTTTAGaCTTTGCAAACATTCGAGATTTTGCAAGTGTAGGGCTATTATTCCGAAAAAACGATGATtACATTTGGTTAGGACATTCGTTTGtAAGaCAAGGgTTTTtGGATGATGTCAAATTAGAaCCTCCTATTAAAGAATGGGAAAAAATGGGATTATTGACCATtGTCGATGATGATGTCATTGAAaTTGAATATATAGTTGATTGGTTTTtAAAaGCTAGAgAAAAATatGGgcTtGAAAAAGTCATaGCtaGATAa

>NRS19contig00055 [organism=Staphylococcus aureus]

ATAACGACGATTTCTTTTTCTTATATAGTGATTTGAAAGGAAATGAACTTAAAGAAGAATTACTTTATGAAGACAAGGTGTATGAAATTTCTGATTACGAGGTTATTCCTGGTGTATTCTtACAAAAAGAACCGGATAATCCTTATGATGAAAaCGCGATAAAAGTTATGATTTCAAATGAAtACTCTGAATTTCACGTTGGATATGTACCTAGAGAGTATGCTTCAAGATTAGTCAATCATATGGACAACATCGTTTCTTGTAaCGCATATATTAATGGTGGTAAGTATAAAaCTTTAGATTATTTAGAAGAGtAAAATCGTTACTAAaGAATCAGACTATGGATTACGAGTACATTtAGAAaTACAAAGTTTGAGATAGGTAAAgaTTGtATTTTTATAAGTAATTAcATAcTAGGTGT

>NRS19contig00058 [organism=Staphylococcus aureus]

AAATATCTATCTTGCCATTTAACCGTCGTATCAAAGACGTTTTCAGGTTGTATGATTAATtCACTGTACCCAGAATCAACATtGAAATAATTACTTCCAAACGATTTCTCGCTCAACATTGGTTCCTCATTGATGACAACACTTTTtGCTTGCATATCTATTTtCACTAAATCACCTTTTTGtATAATGACATCCCTTGCGCCTTTCGGTTTtGGTAGAATCTCcGTATTGAATGAACCTAaTCCATTCATCTCCATCCACTTATAACCATTATACTTCGCACTATAGATAGCTATGATAGAAGCTGGACGCTGATAAAaCTtaCcGCCATCTATCCACTCTTTCtCATCCATATCAaTAGgTTTACgtCcTATCTGGGTCTTTAATGTGATC

>NRS19contig00061 [organism=Staphylococcus aureus]

TAGGGTTGAACCTTACAGCGAGAAGGGAAAGAGGTGTTGTATCATTTGTTGCATCAATGTTGCATCACCAAAAATGATACAACACCTACGATTACTTTTtACACTCACGTGTTGCATCACTCAAAAAaTGATGCAACATCTGATACAaCACTCTAAAATGTATATTTATtCAATATTTCTTATATTAAATCCTCTAAACTTTCATCTCTTACATACGCTATCTGTACACcAtAATCTTTTCCGAATCGAATTTtCCCAcTTTTaTTACCATCATATACAGACCAATTGTCTAaTtGTCTtAAGATGTTTGAAATCTTTCtAaTTTCCaTaGATCCTctACtAtCTCCCTTAtCCTTACcAAAACaTTCaCAAACaCTTCAAgc

>NRS19contig00068 [organism=Staphylococcus aureus]

CTTGCGAATCCcGCAATTGACCAcGCTGTagTGAAGTATAGAAACGGCATgaGTACAATCGCTAAGACTGTGAAGCAtAaTACTGCTAaTAgaTAGCTTTTATAAatGTtACTCATTTTCTTTTTtCAACgCCTCCATTATtCTCTGgTCTGAtAAGtcGTGATAAGGGAaTTTttCtCTAGCTAATTGGACTGGTATTCTGCCTCGAaTCGCAATGTAaCCTTCgTCTTCAAGCTCTTTATTCAGTTCTCTtATTATTtGTCCTGCTTTGGATTtAGAAACaGATAAAaTtActGCAAGTTCTTtAGCTTGCAAACTaTTTTT

>NRS19contig00074 [organism=Staphylococcus aureus]

TATCGCAACATTCAtATTTtatAAaGAatACTTTTATGAaGAaTAAAAAACTGCTACTTGCGCCAaCAAGTAACAGTGACAAaCGATTAaCAAAaTTAATTCGtgTTCAATATAAaaCGAAAAaaGGAGGAaGTCAAGATGTATTaCGAAATAGGCGAAATCATACGCAAAaaTATTCATGTTAACGGATTCGATTTtAAGCTATTCATTTtAAAaGGtCATATGGGCATATCAATACAA

>NRS21contig00011

ATCTATGTGGCCGAAAAaaCCAAACAAAAAaCGACAAACAAAATACTTTGGAGGAAAaGTGAAAaGCGATGATTGATTTAAAAGTAAAaGTTTTTAAAGGCAAGTTAGCATTGTATGATAGTAAATTAAGTGTTTGGAGGATATTGGTATGAGCAATACTGACAAATACCTTAGAGACATAGCAAGAGAGTTAAAaGGTATACGTAAAGAGTTACAAAaGCGAAACGAAaCAGTTATtATTGATGCAAACTTAGACAGCGTAAGgTCGGCAGTATTAGCCAATAAaGAAAaaCCGAAaTatAACGAaCcActCTTTtAATAGCTAGCACTtAATtGTGTTGGCtATTTTTTAtGTCCAaaaCGTGCTGATGACAtaaaaaGCACGCATggaaaaaCAGTCGACAGACTATAAATGGAGGTATATCTCATGgAAGAAATAAaCTTAAGTTtAATTtGCAATTTttGCAGACCAATCAGATGATCCGGATGAACCAGGTGGAGATGGTAAAAaaGAGATCCTGATAATAAAGAAAaTGACGAAGGTACTGAAATAACTTtCACGCCAGAGCAACAAAaGAAaGTtGATGAAATACTTGAACGTCGTGTAGCCCACGAAAaGAAAaaaGCTGATGAGTATGCAAAAGAAAAAGCAGAAGAAGCCGCTAAAGAAGCTGCTAAATtAGCGAAAATGAACAAGGATCAAAAAGATGAATATGAACGCAAaCAaTTGGAAAAaGAGCTGGAGCAATTACGCT

>NRS26contig00012 [organism=Staphylococcus aureus]

acctggtaaatccggcACTCTtGTTGcATAGtAATTACCAGCAGTTAAATATCCCAAATCGCCTTGCGCATTaTTtAAGTTAACTTGAATTGATTGACCATtCGCCTCTGTCATCTTATGTTGTTGCCAGCTCGTTGTTCCGAATTTATCATCTACATACTGCTTAGCTTGATTTAAAGCGTTGTTAGACGTTTCTTCAACAAATTGCTTAGTTAAGTTTCCATCATTCTTTTTATAAAACGGGTACCATGTGCCGTAGATTTTGTATTTTGTGTACTCATCGTTTGAATCGTCTGGGTACCATGTTGCACGAGCAGTATTATtATCAACAACATAAACAACTAACACACCAGATTTGCTTGATGTATAAGTTGATTCATCGAACGAAGAACCGTCATCAACACCATCTTGTCCAGGCTTCTCTAACGTGCCTATATCCGTCTTtCTGGCGCATCTGTTGCATTAGTAATATGAATAATCATAGATGAGTTAGCGTGTCTTAAAACAGCTTCTATTGACTGTTCAGATGATTCGATCGCTTTACCGTAATCATCAGTAAGTTTAGACTTTTGCCAATTTGTTGTTGAATTACCTTTAAcAAGGTCAGCGCCATTGATTTGTTGTTCAACTTCGTTAACACGTtCAAAAaTCGCTTGCTCTTtATCAACAATTTTCTGGAACTCGCTATTTATATATTGAACGGCTTTGTCTTGTGTTGTTGTAATCATCTGTACCGCTTCATTTTGTTTAATTTCtAATCTTTGAATACCTtGATTAATACGACTATCAATTTCAGTAACCAaCGATTTTGTATCACTTAAACTTTTCTTTAAGTCCTCAACTTCTtCTttAACACTTTCTGTTAAGTCCTGAATTGATTTGATATAAACTAGCTTTGTTTTACCGTCAAAaTTACTAATTAGATCATTCTGGATATTGAAGTTAAATTGACGCTCTACAATTACGTTATTGCTACCGTTTtGAGTAAAATATGCTTGCGCATGTACTCGACCAGTGtATTTTAaGAACTCGTTTGGGATAACGTaTTGCATTCGTCCATTAATTGCATCAACAaTTGTAAGTTCATCaCTAATATAAGCGCCGTGTTCATCGTCGAAGTTATCCGTCTTAAGCACAATACTAGTCATCGCATTATGTTTGCTGATTGATAACGGCTTATTATTCTTAGTTACTGCAAAATTTAAAACACCAGTTCCTCTATCTGATTCATAGAAACTGATGTTTGTGTCAaTAATTGGATTATATTGTGATGTTGTttGTAACTCGATTAAGTTATCGTCTTTCGAAAAaTTATCTACTACCATTATTCAACCTCCTTACCTTCTATTATGCTCCAACCACTATTACCACCAGTACCAAAGTTTCTAACGAAAAACTGGTGAGCAGAAGCAAAGTTATTACGTCTTAGCACTTGTGTTGTGTTACCCGGTGTATTTGATTTTACTTCTAACACCCAGCCTGCAATACCTTTGTAATCTTTAGGGAAGTCAGAAAAACGTTTTGATTCTTCAGTGGTGATATAGAAGTCTAAACCAACAATTTTTAAAtCAGACAATTTCGTGATGCTCTTAGGGATATGTTCCCAATAACCAGCACTTTGtGGGTTAAAATTCCATGAaCCGTTGTTTTTCTTGTTAAAGaTGTCGaTAaCACGTTCAAaTTTGAGCATATTTCTACCTGTGCTGTTTCTaGTtaGTACTTGTCTTAACGCACCATTATAaTGaCCAGGCAGTACATCAAAGAaCCAACCTGCATCTCtAAACGTTTtCGgtAaCGGGAAaTCTAACGCATTTtGTGTGTCTtGCGTATAGaTATaGTAATGACCAACTTCCGTAATATCACTTAGATATGCTGGGTTTtGTATTGGTAaCGGTTTAACACGTCCGCCTGAATCAGTCAtcGATACTTGAGGtGCAaTGTTTTtAaGAaTtGGTtAaCACcTCTTtGGCCGATGgAATAAATTGAGTGATGTCTGTTGTTACCAGGTCCAATAGTTACCCCTATTAAAaGCGCTTTGCGTCCTGTTTCTAGATCGTAATACATATCTAGACCCTCAGCTTCTTGGAAGTCTCCTTTAAAGTTATTATTCACACCGCCAATATCGATACGTCGTTTAAATAaCAATtCTTTtGTTTTtATATCGAAACCTTGTAAGTAGTTAGGGTTGGCTGTATTCGAATCACCTGTATACCAATATAAGATACCTGCATCATAAGTGATACCTTGCATaGGTTGTGTATCTGAAGTGTATTCCATAGGTATATCCATTTGATACAATACTTTGTCTATACCTTTATCAATATCGTCAGCACTTCTAACCTCAACAAAGTTCAACGAATTCTTAAGTTGTCTTTCAGTGGGTTTATATTCACGTCTAAAAATCATTAAATTTTCTACCGGATTATAAATCGCTGACGTATATCTGTCGTTAAATATATTCGGCATGACATCTTGCATTTCATTACCATAAGTTATTTCTCCAGTTCTATATTGGAAACGTACAAACTTGTTGTTTTtGTTACTGTCCAATACAGCTGAATAAaTCCATAATTCTCCATCAATGTATCTATACGCATTGTGTGTACCGTGACCGCCGTTTTTAACAAGCAATCTATCAATAAATTGTCCGTTGGGCTTCAATCTAGATAACATGTAATGATTACCTGGACGAGCTTGCGTCATATAAATAATTTTCGTTCTAGGGTCTACCCAAAATGATTGCATTACTGCatttGTATATGGCGATAAATCaGTGATgAaTTCCGGTTCTTGCTCTTTTGGTTCGAATCGGTATTCTGTCGCTCGATATTCTTTATAGTGTTCATCTACAGCTTTCTCAACCTTTTTAGTGAAAGCATCTAGTGTTGAATAATCATGATACAAACGATCTTGCAATGTCTTATGAtCATAACCaGTATTATCAACaCGCGCGTCTTTTACTTCGTTGATACCGTCGCCGTTATGGCCTAGAaTCATATTGCTAAAACGgCCaTTTAAATACGTTAAATAATCTTCAACACTgTCATTCAAGTATTTAATTTGTTTCGCTGAGTGTGCGTATATTTCTTCTTTTTGATGGTATATAAACATTTTCTCAAGTTTGCTCATACCTTCATCTAACAAGCGATAGTTATACTCATGTTGAGCAACTATTTTCCGACCTGTCATTGAATGTAAACTTGTAATTAATCCGTAAGCCATTGGTTGCCTCCTTTAGTCGTAAAAACTGTAATAATCCTTGATTAACTCGTACATAATAACCTCGTGACCTTTTtCGTTAGGGTGTAAGCCGTCCTCCATGCTCGCTTTCCTAAAAGCTGGATTGTATGGCTTAAAGTAATCTGTGTGATATGCGTCAAACACTGGTACATCTAACTCACTACAAGCTAATATTTGAGCGTTTACATAGTCCTCAAGTGTTAACCCTagTTtGTTTTtgTCcGTgTCTTTACGgCGTATtGTTGTaCCACTCATAGGGCATTGTCTtGTAGCTGTCATCACTAGTATTTTTGAATCTGGATTATTCTTTCTAATAaCTTCAATTGCAGAACAAAaGGCACCGTAAAACGTTTTtGTATCCGTTTTATCAGTGCCTATCGGTACgCCTGCCCAATAACCgTGTAACCAGTCATCATCaGTgCCTTGTAATATGATTAGGTCTCCTCTTATTTGCTCTGCTTGTCTATAAATGCTGTTTTCTACCGCTTCTTTACCTATTGGAACTGTTGCCATtGTtGCGCCACCTCTTGCAAGATTAGTCGTTTTAGCTTtCAATTtCTTGCCTAACATTTCTgTGAAATTaGTTTTtGCgTGCGACCCTcTAGCTACaGAGTCGCCAATcGTTCcAATTGaTTtGAtGTTtCTtAtACTTgATtgACTaGTAAAgTCgTAcATGATCGtaccattagcagttgtaactgttttagtattcatcttatcgactttagcgtttatttttcattctgcttaaccaattcattatttatag

>NRS26contig00013 [organism=Staphylococcus aureus]

GTtGTTTTCAAAAACAATCGTGAAGTAtctGTTTTCTCTgTCGTTAAACTCGACATTTGCAaCTTTTACTGTAAATTCTCCAGCTCCTAAAAAGTCCCCACcTTTCATGAaTGCCTCTTGATtAGTTtCTTGAATGTATtGTGTtCTACCAGTGATTTTCATAATTTTTATACCGTCCTTTTAATTAATTTTtAATTACCATTTCTAATTGCTTGTACAACATCGTTAATACTTGGATTAATGAAACGTTTGTTGTtAATTTtGATGTTGCTTGAGTGTCTTATCTTTGTCTCGAATAAATTTGATGGTTCAGCGTTAAGTACATATTGaTAAGTTTTTtCGCCGTCTTGCTCATGTTCTTCTATTGTCATTCTTGCTAACACGTCAGATTGACTGATGACTGCTTTTTtATTtGGTCTTGTGCCTCTATCGTGATTGTTGGATTGATAGTACTTCCcTCATCATCTTTGtCTTTGTTAATGCCCTcGTGTCCGCTTATagCAAGaTGAAaTTGATAATGTTCTTGTAATTTaGAAaTaTAACGaTAAaTACTTACAATGCGTGTAGCACACTcGCCCCAATCATTAAATgtCGGTTTCTTTGATTTACCGTCCATGATGTCGTCCATAGTGATATCACGTAACTTTTGGATTGTTTCAATCACTACAACATCAATTTGTTTtCCGTTTTCTCTTAGTTGTTCAATAaTTTTAGGCAGCATTTTAATCACTGCACTAAAATGCTTATAATTCTTAATCTGCACAACTGCCCCATCTTCTGTTACCGTTGTTCCGTCCTCATTTATATCTAGTACTAAGGTATTGTTATCTTTTGTTAAAAACGTAGTTTTACCAGTACCGAACTTGCCGTATATCGCAAATTTATAAAACTTGTTTGCATTTTGTttGCTGATGTCTTTtACACCTAGTTGCGTTAAAATATCGACATCTTGATTAGTTTGTTCAGTCATGTTCTACCTCCTCGTACTCAATAGTTTCTGTCACTGTTTTCTTGATTGCTTTGTGATAATCCATATTGATACTCGCTTCTTCCATACCGTTAAACTCCCTAGCTcTATTTCTATTTGTGGAGTAACTAATATCTGAATTGTTATCAGTTGGTTTGTTAGTTATATAAATTGGCATATCCCTATGACGAATGATATAAGTTACAGTCTGCTTCATAGCGACCTCCTACCATTTCATGACTAAGTTAATTAGTCTGTCCTGTTCATCTGTGTTCTCTTCAATCCATTCATCTATTGCTTGGTTAAATAaGTCTGATGCCATATCTAAGTCATTCTCATCTACGACATAAGCATGTTTAATTGGTACGTTGTTCATATCTTTAACTTGTATTGATATGCCCATATGACCTTTTAAAATGAATAGCTTAAAATCGAATCCGTTAACATGAATATTTTTGCGTATGATTTCGCCTATTTCGTAATACATCTTGACTtCCTCCGTTTTTCGTTTTATATtGAACATGAATTTTTtCTtAAGTGTTTTGTTTGATACTGTTACTTgTtGGCGCAaGtAGCTAgttttttattcttcatAAAAGTAactCTTTtAttAGAAtaTGaatGTTGCgaTACTtgCGAATcctGCAaTTGacctAcgCTGtAGtGAaGTataGAAaCGGCAtGAGTACAATCGCTAAGActGTGAAgCATAGtACTGCtActAGGTAGCTTTtAtAAaTGTTACTCATTTtctttttCtCcTCTTTGGTtGTTTCATCGTTTATCAAaCCTTGCATTTCCATtAATTTTtGAGGTATACCAGCTTTTAACTGGATTtCGTATAACATTtGTTGAATGTGTGGTGGCACTTCTACCATTCCTTTCGTGTATAATTTAGTTATCTCCTAGTGAAATGAGGTGATAAGTATGGAaTTTAATGATTTtCAAAaTTtCTTTGgTGAACTTAGTAATCAAGCCGAAAAaGAATTCGGTGGTGACAGTGACTTTTTTAGAGATAGAATAAATAAGTTGAAAGAAGATGCTCCTGAAAACGTATCTTACGAAATtATTtATTCAATAGCTTTATACGAAAGCTTAAAAGCTCAACAAGATATGAAAATTTTGAATACAGTTAAATAtCTTTTAAATCGTGACTAGCAATAtCCAACAaTGATTTGCTCtGAGCATTATTAATTTTTGGATAATCAAAATTtCTAAGTTTAAATCTTGTGTTTTtCTCAATCTTTACAACCTTCCACGTCACAACTGCCATTGTGATGAGGAGGGTTGTTTTGTATAGTGTGTTCATTGATAATTCCTCCTATTAAGATTTTtATTTTtCTCCTAAAAaCTTATTAACAAAGTATTGTTGTCCTTTGCCTGTTACTTTTGGCGTCTTACTAATTGATGTGTGACCGTCCGAATGTGTAATTGATGTTTCTTTAATTTCGAATAACTCACGTTCCATTGAATACTGTGTAGGCATGTTGTAATCCACACCCTTGCGTTTAATAAGGAATCCGTTTtGACGTAACCACTCAAACAATCTGCGTTGCCCGATGTTTACACCGTTTTGTTTAATGATCTTCGCTAACTCTCCAaCTAAAATTGATGTCTTAGTAGTAGCTACTGCGTCCGCAAATACAATCTTTGGTTTGTCGCGTTCAaTCTTTGTTTCTAATTGATTGATTGTGTTGTTAGCAATTTTtAAAGCACGTTGCATAATCATTTCtGGGCTATTCCAAGCTTTtCAACTTGGATGAAATACTCTCTAAAATCAAAACCCTTTtCTGTACctGaCaTCATCGCAACAtGTTTAgCTACATCAAGTGTTAAAGCATAATCTTCTAGTTGTCTTACAGCTCCGTTATTAACAACCGTACTTGTAAGTACACTTGtAAAATCTCTGTTTCTTtAAAATGCTTtAaGTtAATTtCTGCCCAAGCGCTAAAaCGTTTTTtGACTTCCAAAGCCTTGTaTAACTCTCTTGCACTGATTGCGATTTCTCCATTTtCTTTTtCTTGTATGTTGAACATTTCGCCGATGTTCGATTTtGTTTGTAATGCTTGCATTTtATTTCTCCTTTACATTAGCGATATCAACTTGTAGTGCATCGCATATTTtttACTGTGAGgAAaCcGGGGTTTTTAACcTCTGTTTCGATAGATCGAATTGTCGAGTTTTGTAATTCCGTTAGCTTCGCTAGTTGATAGCGTGTTATCCCCTTTTCTTCTCTCAATtCTTTtAAGTTCAGCATCTTACCACTCCTTATTGTCCATAACGATATTTCGTTATATAATTAATCCAACCCCACTACATTGGGAGGTGATTTCCTTGCTTATGCCAGGTTTTAAATCATCCTGTGGTTTTATAGGTTAGTAAGTCTAAATTAGAACATCGTTTGTTGTGTTCCACAGTCAACCAAGAGACGTTAACTAGGGTATGCGTACTAGAAGGTAGTAACTTTTAGGACGCTAGACTTTGACGGAAAACCTAAGCACCATACAGGGCTGGGGACGATACCAGCAAAAaTTGTGCTGTTAGTCGTAGTAATTAGAACCGAACAAAATTTCCGTAACACATACCTTCTACGACAAGGTGTGTGTTTTTTATtGGAAACAAAATGTTTGTAATGCTTGCATAATATTTATGCTCCTTTCATGTATAATGTTGTTATCAAATATTTAAGGTGGTTATTCTTATGGAATTCATACAATCTACTTTGTTTTCAAACGTTGTAGCTTTTCTAGCTTTAGGTCTATCTGCATACTCAATTTTTtAtACTCGTTCTCAAAATAAGTTCAGTTTTGTTATTAGCGATCTTAATTTCTACTATGAAAATAATTTTGTAGAATTAAATTTTGTCGTCGCTAATGACTCGTCTAGAACTCATACTTTAGAAGAATTAATATTTTtAGATAAAAaCAAAAATGTTTtAACACCTATTAACGTAGTATTT

>NRS26contig00016 [organism=Staphylococcus aureus]

ATTACATCATTACATTAGTTCTAATGGTTGTATTCATAGTAATATTTAACAATTTACTCAACAGATATATGgTTTTGTACAAaGAATTAGATTTATTtACATGCaGAATTGGCATGTTATtGGCCTTAATCGTtCtAGTAGAaTTTGCAAAGCAaCAAAATaTGTTGGCTACATTGAGTGTTTtACTAATACTTTtATTCgTAGAAAAACTTaGAatCATTCAAAGgAgTGGCGAGAAGTGAATAAGAaGGAAaCGTATTATGTTATAGAGGTTAATAAAGGAATTTATTTATATAGAAGTCGTGCTGGTGGATATCATTTCACTTATGATTTTATGAATGCGTCGAAATATTCGGATGCTAGCGATGCTGGTGATATTGCCAGAAAATCAGGTGGAAAAGTGTTGTGTTACACAATTACGCATGAGGTAATAGGCTAATGCAATACTTAGTCACAACATTCAAAGATTCAACAGGACGCAAGCATACACACATAACTCGAGCTAAAAGCAATCAAAGGTTTACAGTtGTTGAGGCAGAGAGTAAAGAAGAAGCGAAAGAGAAATATGAGTcACAAAATaCACCTATTGTTTACTACACTAATAATTCTAAAGTGACCTTATTCGAAAGACCTAGTGAAGAAGTATTAGGTtCTTTGTTCGAAAAGAAaTAAAaTCATTAAAGAGGGGAGATAATAATGTTTAATACaCCTAAAATGAAATTACCaGAAAAGCACACcGAGGTATTtAAGACGTATAAAAATGGAACGCCAGAAGAAAAaGcTGAGaTTGAAGGCTGTTTTATTAAAACTGTTAAAGATGAAGATAGTGAATTTTACAGCCCTATGTTAGCCAGTCTAAATGAACAACAGTTAAAGAGTATGTTGAGACAGGtACTTTTTTtGATTGATACAGGAGATGACAATGATGATTAAACAAATATTAAGACTATTATTCTTACTAGCAATGTATGAGCTAGGTAAGTATGTAACTGAGCAAGTATATATTATGATGACGGCTAATGATGATGTAGAAGCGCCGAGTGACTTTGAAAAAaTCAGAGCTGAAGTTTCGTGGTAATAGCTATTATCATTTTTGAATTAATtATATTAATGTGTTTAGCAATAGCAcTGGAGgTgTTGtAAATATGTGGATTGTCATTTCAATTGTTTTATCTATATTTTtATtGATCTTGTTAAGTAGCATTTCTCATAAGaTGAAAACCaTAGAAGCATTGGAGTATATGAATGCTTATCTTTTCAaGCAGTTAgTAAAAATAaTGGTGTTGAAGGTTTAGAAGATTATGAAAATGAAGTTGAaCGAATTAaGAAAAaGATtCAAAaGCTAAAGaGAGGCGTTGGCTtCtCTGCtCTATCTAAAATAATGAAAGGAGCCGAACATGTtAGACAAAGTCACTCAAATAGAAaCAaTTAAATATGATCGTGATGTCTCATATTCTTATGCTGCTAGTCGCCTATCCACaCATTGGACTAATCACAATATGGCTTGGTCTGACTTTATGCAGAAGCTAGcACAAaCAGTTAGAACTAAAGAaGATTTAACTGAGTACAATAAAaTGTCTAAGTCCGAACAAGCAGATATAAAaGATGTTGGCGGATTTGTCGGCGGTTATTTAAAaGAAGGTAAaCGGCGTGCTGGTCAAGTCATGAATCGTTCAATGCTAACACTtGATATCGATTATGCTGCTCAAGATATGACCGACATATTATCTATGTTTTATGATTTTGCATATTGTTTATATTCAACACATAAGCATAGAGAGATAAGTCCAAGACTGCGTTTAGTGATTCCTTTAaacGAATGTAAATGCAGATGAGTAtGAAGCTATTGGGCGTAAaGTCGCaGATATCGTTGGCATGGATTACTTCGATGATACAACTTATCAACCACATAGGTTAATGTATTGGCCTTCAACTAGTAACGATGCGGAATTTTTCTTTACCTATGAAGATTTACCTTTGTTAGACCcAGATAAAATATTAAATGAATATGTTGATTGGACTGACACATTAGAATGGCCAACGTCTtCAaGGgAAGAGAGTAAGACTAAAAGATTAGCAGATAAGCAAGGcGACCCAGAaGAAAaGCCGGGAATTGTTGgtGCATTTTGTAGAGCCTATACGATAGAAGAAGCTATAGAAaCTTTTATTCCTGATTtATACGAAAAaCATTCTACTAACCGTTATACCTATCATGAAGGTTCAACTGCAGGTGGATTGGTGTTATACGAAAaTAACAAGTTTGCCTATTCTCATCATAATACGGATCCCGTAaGCGGTATGCTTGTGAACAGTTTTGATTTAGTACGCATACACTTATATGGTGCTCAAGATGAAGACGCTAAAACAGATACTCCGGTTAATCGACTACCTAGTTATAAAGCAATGCAGCAAAGAGCGCAAAATGATGAAGTTGTTAAAAAGCAATTAATTAACGACAAAATGTCTGATGCAATGCAGGATTTCGATGAAATAGTAAATaGCGATGATGCATGGTCTGAGACGTTaGAAATTACTTCGAAaGGTACTTTCAAAGCTAGTATCCCAAATATAGAAATTATATTGCGTAATGATCCAAATTTAAAaGGAAAAaTAGCCTTTAACGAATTTACGAAACAAATTGAATGTTTAGGGAAAGTGCCATGGAATACTAATTTTAAGACACGTCAATGGCAAGACGGTGATGATAGCAGTTTAAGAAGTTATATCGAAAAGATTTATGACATACACCATTCAGGTAAAACAAAAGATGCCATTATAAGCGTAGCAATGCAAAATGCTTATCATCCAGTAAGGGATTATCTAAATAAAATATCGTGGGATGGACATAAACGTCTTGAAAAGTTATTTATCAAATACTTAGGTGTTGAAGACACTGAAGTGAATAGAACAACTaCCAAAAAGGCATTGACTGCTGGAATCGCTCGAGTAATGGAGCCTGGATGTAAATTTGACTATATGCTTACACTTTATGGTCCTCAAGGTGTAGGTAAATCTGCTTTGCTAAAAAaTTAGGTGGTGCAtGGTTTTCTGACAGTTTAGTTTCTGTTACTGGTAAGGAAGCATATGAGGCATTACAAGGCGTTTGGTTAATGGAAATAGCaGAACTTGCAGCTACAaGAAAAGCTGAAGTTGAAGCCATTAAGCATTTCATATCTAAaCAAGTTGACCGGTTTCGTGTTGCTTATGGACATTATATTGAAGATTTTCCAAGGCAATGTATTTtCATTGGTACAACTaaTAAAGTTGATTtCTTAAGAGATGAAactGGTGGAAGACgTTTTtGGCCAATGACTGTAAATCCAGAGAGAGTTGAAGTGAACTGGTCTAAACTAACCAAAGAAGAGATCGACCAAATCTGGGCAGAAGCTAAATACTATTATGAACAAGGAGAAGAGTTGTTCCTTAACCCTGAACTAGAAGAAGAAATGCGTTCAATACAAAGCAAaCATACTGAGGAATCTCCATATACAGGTATTATTGATGAATATCTTAACACACCAATTCCTAGCAATTGGGATGACCTAACTATTTTTGAACGAAGACGATTTTATCAAGGTGATGTTGATATGTTACCAACAGGAAATGTAGATTACGTTGAAAGAAATAAGGTCTGTGCGCTTGAAGTGTTTGTTGAATGTTTTGGtAAGGATAAGGGaGATAGTAGAGGATCTATggaaattagaaagatttcaaacatcttaagacaattagacaattggtctgtatatgatggtaat

>NRS26contig00031 [organism=Staphylococcus aureus]

gtatacGGAtaCAAAaaTTGaCTGtAactaGTtGaTAGCGcGCctgAatcTATGAaCaCATTAAGAGAATTAGCAGAAGCAATACAAAACAACTCTATTTCAGAAAGTGTATTGCAACAGATTGGCTCAAAAGTTAGTACAGAAGATTTTGAGagATTCAagCAATCATTAAACAGTTTGTATGCAGATAAAAaTCATAGTCATACAATCAAACAGATTGAaGGATTAGAAAaTGCTTTATCAAAAAATCAGACATAAATCACAGTCATGATGAACGTTATaTTTtATCATCAAATGCTTTTACAAAaGAGGAAGCAGATAAACTTTATCAACCTaTCGGTTCTTCGCAGCCGTCACTGAATATTTGGACAGGCAGTGAAACAGAATATAATTATTtGTAtcAAAAAGACCCTAATACACTTTATTTAATtAAGGGGTGATTTTtATGGAAGGTAATTTTAAAaaTGTAAaGAAGCTTATTtACGAAGGCGAAGAATATACAAAaGTATATGCTGGAAATATCCAAATATGGAAAAaGCCTTCATCTTTTGTAATAAAaCCCTTACCTAAAAaTAAATATCCGGATAGCATAGAAGATTCAACAGCAAAATGGACAATAAATGGAGTTGCACCTAATAAAaGTTATCAGGTGACAATAGAAAATGTACGTAGCGGTATAATGAGGATTTCGCAAACTAATTTAGGGTCAAGTGAATTAGGAATATCAGGAGTCAATAGCGGAGTTGCAaGTAAAAaTATCAACTTTAGTAATCCTTCAGGGATGTTGTATGTCACTATAAGTGATGTTTATTCAGGATCTCCGACATTGACCATTGAATAATTTTAAACGACTAATTTTTAGTCGTTTTTTtATTTtGGATAAAaGGAGCAAACAAaTGGATATTAACTGGAAATTGAGATTtAAAAaTAAaGCGGTATTAACGGGATTGATTGGGGCATTATtGCTATTTATCAAGCAAGTTACAGATTTATtCGGATTCGATTTATCAACTCAATTAAATCAaGCCAGCGCGATTAtAGGTGCTATCCTCACGCTACTTACAGGGATTGGCGTTATTACTGACCCAACGTCAAAaGGTGTTGCTGATTCATCAATAGCACAGACATATCAAGCGCCTAGAGATAGTAGCAAAGAAGAAcAaCAAGTCACTTGGAAAaCTTCACAAGATACTAGCTTAACACCGGAATtAAGTACAAAaGCACCGAAAGaGTACGACACATCACAACCATTTACAGACACCTCTAATGAAAtCGGTTTtGACGTGAaCgAGTATCATCACGgAGgTGgCGACAATGCAaGCAAAaTTGACTAAAAaGaGTTtAtAGAGTGGTTGAAAaCTtCtgAGGgAAAaCAaTTTAatATCGACCTTTGGtATGCATTTCAATGCTTTGaTTATGCTAATGCTGGTtGGAAAGCTTTGTTTGGATTACTCCTAAAAGGTGtAGGCGCAAAAGATATtCCGTTCGCTAACAACTtCGACGGATTAGCTACTGTATACCAAAATACACCGGACttcttagCACAACCctGGCGACATGGtGgTaTTCGGTAGcAActACGGTGCtGGATAtGGtCACGTtGCATGGGTAATTGAAGCAACTTTAGATTAcATCATTGTATATGAGCAGAATTGGCTAGGCGGTGGCTGGACTGACGGAATCGAACAACCcGGCTGGGgTTGGgAAAAaGTTACAAGACGACAACATGCTTAcGATTTCCCtATGTGGTTTAtCCGTCCtAActtcAAAAGcGAaACAGCGCCACGATCAataCAATCTCCTACACAAGCACCTAAAAAAGAAACAGCTAAGCCACAACCTAAAGCAGTAGAACTTAAAATCATCAAAGATGTGGTTAAAGGTTATGACCTACCTAAGCGTGGTAGTAACCCTAAAGGTATAGTTATACACAACGACGCAGGgaGCAAAGGGGCGACTGCTGAAGCATATCGTAACGGATTAGTAAATGCACCTTTATCAAGATTAGAAGCGGGCATTGCGCATAGTTACGTATCAGGCAACaCAGTTTGGCAAGCCTTAGATGAATCACAAGTAGGTTGGCATACCGCTAATCAAATAGGTAATAAATATTATTaCGGTATTGAaGTATGTCAATCAATGGGCGCAGATAACGCGACATTCTTAAAAATGAACAGGCAACTTTCCAAGAATGCGCTAGATTGTtGAAAAAaTGGGGATTACCAGCAAACAGAAATACAATCaGATTGCACAATGAATTtACTTCAACATCATGCCCTCATAGAAGTTCGGTTTtACACACTGGTTTTGATCCAGTAACTCGCGGTCTATTGCCAGAAGACAAGCGGTTGCAACTTAAAGACTACTTTATCAAGCAGATTAGGGCGTACATGGATGGTAAAaTACCGGTTGCCACTGTCTCTAATGAGTCGAGCGCTTCAAGTAATACAGTTAAACCAGTTGCAAGTGCATGGAAACGTAATAAATATGGTACTTACTACATGGAAGAAAGCGCTAGATTCACAAACGGCAATCAACCAATCACAGTAaGAAAaGTGGGgCCATTCTTATCTTGTCCAGTGGGTTATCAGTTCCAACCTGGTGGGTATTGTGATTATACAGAAGTGATGTTACAAGATGGTCATGTTTGGGTAGGATATACATGGGAGGGGCAACGTTATTACTTGCCTATTAGAACATGGAATGGTTCTGCCCCACCTAATCAGATATTAGGTGACTTATGGGGAGAAATCAGTTAGAATGACATAGTCATGTCTATTTGAGCAGGTGCGTTACATACCTGCTTTCTATTTACATTTAAAGATAAAATGTGCTATTATTTTaCTAGAaCTTTTTAACATTTCTCTCAAGATTTAAATGTAGATAACAGGCAGGTACTACGGTACTTGCCTATTTTTTATGatATAa

>NRS26contig00032 [organism=Staphylococcus aureus]

AGTTCTTGCTCTTTTTAATTCGCAAGTCATTTTTTCTTTAGTTCTaGTTAAaaTGTTTTGcTTTTCTTGTTCATCGAACgcACTATACTTaTCAaTAAgTtCTTGAgTTTTTtCGAGTtCcTTTttATttCTTTTTtCTATTTCAGCTATAAGGTTATTAGATAAATCCGCTTCAATTTTCAAAAGTTTTTTGCTTTGTCTTCTGTTATTTGACCCGAGTTTAAACGTACTTTTTCCATGATTCTGTTGTTCTCTTCAGAATAGTGTACGTATTTTtCTAAAGCTTTTTCTGTTTCTTTTGAAACACCTTTCCCCAACACTTTTACAGTATCAGATGCTTTTTtAGAAGCTGTGCCCATGgTTTGCATAAATCCTTTAAACTTGTTGACTCCTACTTTCAGAAGGTCATCGTCACTCAAAGATTTAtAACCATCTTTCATATCCTTTGAAAACTTTTCTTTGAAGCTTTTGCCTATACTTCCAAGATAATTTTTAAACtCTCCTAGCTTCCTAACAGCACCGCCAATAATTTTGCCACCAAAAAaCTTTatAGTTTCTCCTAAACCGTTAATACCGTTTCTGAACCATTCCACACGATCATATGCGGTTTTAAAAaCTTTATATGCAATTGTAATAGCAGTTATTATAGCACCTATAGGTCCTGTTAAAAaCTTTAAGGCTACACCAGCAAATCTTGCGCCTCCACTTACTGCAAATAAGGATTTTGCGGCTAATCCTAAACCGTTTTTCAAAaGTTTGAACGGTAAAaTTGCTAGCTTTGCAGAATTTTTCAAAACATTTATAGGTTTTAAATTAAACAACATAGCTCCGGCTAATCCTTtAAAGCCTTTTGACGTTTTTCCTGTTGTAGAaCCAAGAAATAaGGTTTGAaGACCTAAAGaTTTCATTGCTTTTGAATTGGtATTAGACAGTATTGtATTTTCAGCAaTGCGTCTATTTAATGATGCATAGCCTTTAGCcGCGCTTCCAAcTGCACGTATTAATAAGCCACCAGCAAGAACAGCAGGGCCAATAGATGCACCAAAAATCGCTAAACCTACCGAAGCCTTTCTAACCCAACCAGGAAGATGTGTAAATCCATCAACTAATTTTGTTAATCCTTCCGCACCTGCTCTAATCATAGGCGTTAAATCTTTACCAACTTCAATTGCTAACGATTCAAAAGCGCCACCTAATTGTTCCAGAGCACCTTTGAGGTTGTCTTTCATCAAATCAGCTGCTTTTTTACTTTCACCATTAGAGTTCTTCAATGATTTGCTATAGCTATTAATTTTATCTGGACCCGCTTCAATCAAGGCTAAAAATCCACTTGCTGCTTCAGTGCCAACTATTGTAGCCACTGTAGCTAGTTTTTGTtCTCTCGTCATGCCTTTCATATTATCTTGGAACTGTCTAATCAATTCACCCATGCCAACAAATTGACCTTTAGCATCAGACAAATGAATACCTAATTTTTtCATTTCCTTAGCTGTATTTTtACTTGGATTAGCTAGCCTGATAAATGAAGCTCTTAGGGCAGTACCTGCTTGAGAACCCTCTAAACCTGAGTTAGATAAAACTTCAATTGCTGCGGAAGTGTCCTCTATTGAAaCTCCTAATGCTTTTGCAGGAGTACCAGCATACTTCAATGCATCTCCCATGTACTGAATATCTGCAGCACTATCATTTGCTGATCTCGCAaGTAAATCAGCAACATGATTTGCATCAGATGCTTTTAAaCcGAAaGAGTTAAtCGCTGAAGCCATTACAGTTGCAGTTGTAGCCATTTCTGcACCACTTGCTTCTGCTGCACTGATAACACCTGGCATAGCCTCCATTGTTtGTTTGGCATTAAAGCCTAAAGCTGCCAATTCTtCCATACCTTTAGCAACTTCGTTAGCACTTTTACTGGTTTTAGCTCCTAAGTCAACTGCTTGATTAGACaTGCTTTtCAaGtCTTTAcTGCTTGCTtGCGCAATCGCTCCAAcTCGAGACATTTGGCCTtCAAAGTCTGCACTtGTTTttAaTGCtGCACcTAaCCcTAAAGTAATTGGTGTAGATACaCCCATCGTCaTTGTACGTCCCAGGGAAGTCATTTTGTCTCCAATAGAaCTAAATTtCTTTGACATGACATCCGCTTGACTTGCAAGTTTACCGAAATGACTTTGAGCTATCATTTGTtCTTTgTTAAAAGTCTTCATTTCGGATGAAGCTTTATCTATTGAACGCTCCAAATtATTTAAAGCAGCTTTTTCTTtATTAACAGCTGTTTCAGCTTTTGCGAcATTAGCgCTATGATTCTTtAaTAGTATTGTTTAAaTCATTAAATTCTTTTTCTGTTTGCTTTAATTTaGTATTAGTTTTAGCGTAAGAACTTTCAATTTTATCATTTGATTTTGAAAGATTGTCATTTTGCACTTTTAGTTTTTGAACTTGATTGCCTTCTTGTTTATATTGTTCAACAAGTGCTTTATGCTTAGCGGACTGCTTCTGTACTGCGTCACTTGCTCTTTTTAGTtGTGCAGTAGTAGCTTGGTTACTATTCTTAAGCTTTTGTTCTGCATCTCTCAACTGTTTAAGTTTTtGATACGCATCTTGTTTACGTTGATTTGTACGTTTATATTGATTTTCAGCTTTTTTAAGTtCTGTATTCGATGATTTTAAGGCTTCTTTAGATTTATCAAGAGCTtAATTTTTCTTTTTtATtGGCTTCTACTAACTTTAAATATGCTTTCTCAACatCTTTtACACTGgATTtAgCTTTTTGgtAATtaGCGTTAaCTTGTTTAAGCTcATCTTCTACTTGaGAATACATCTTTTttGAaCTTtAAGCCtatCaTTTAaCCCCTtAATTCTCGCCTGATATTTttCcaTtGATTtttCtAGACTtATCAAaTGaCTGACagaTTAgctttcatttcacta

>NRS26contig00038 [organism=Staphylococcus aureus]

ATACCACGTATAGCtCCTAtaCCACTTACTTTACTGTGACCTGTCTGAACCACTTGAAcGCCAAaTAACATGAATGAATTATATTTATCAAAATTAAaCTCAATGCCATATTtGTTAGTTAACTCTATtAGtACGTTTTTttGAaTCGTACCTAaTGTTGCACCAGCAAGTATATATTGAGGTGTCTCAATTCCTTCTTCGTCTGCTATCTTtCGCACACGCATTAACTCACGTAAAAaTAaGTCATTGTTTAATATTGTTTTACCtGTACGCTTTGCTCCGTGATTAATTAACATAAaCCAATCTTGTTTTTGCGTTTGCTTCAATATTTCAATTTGTTTGTCCGTATATAAAGATTTAAGTTTATTCATTGACAATCACTTCCGTTATTGCGTCGTGAAGTTGTTTGATTTTATCTTCTGTTCCACTGTCACCTTTATCTATTTGTTCAATCTTCTTCTCAAGCATCTTAATTTCAGTTTCTATTTTCTTGTTAGCTAAAACTTCGTTACCTAACGTCATTCTATTCATACCATCTAAACTAGCGAGGAATGCATCAGCTGTCGCTTTCTTCACTCCCTCTATTTCAATGTCATTCTTAGCTACATTCTTTAGCCACTCATATTCTTCAAAGGCCTTTTGGCGTGTCCATTTTGATTGTTCAGCTACTTCTTGACGCAATTTTTCGtACCTTCCGGAAACCTTCCGATTTTTAAAAAGTGTACTCGCTTCTTTATCTAGATATTCCCCACTCTTACCTTTAGTCGAATACCCTGCGTCAATATATGCTTTCCGTTGGCTCTTGCCCTCTATGAGTCCTAGCACAAACTTTTCTtGCTTCGGTGTTAATTTAATCAATTGTTTTCACTGTATCACACGCCTTTACGTTAATTACTCTTGTtATTTTTtAAATATAAAAATGCCCCTACATCTCGTGCAGGAGCTACGTTCAATAAATGTGAAAGGAGGAAAATAGTTATGACTCAAATTGCAAGAATTAAACTACCCACCATATAGGCAGGCAGTAAGTGATTAATAGCGTAACATATCAACTTTACATGTTTGTCACTTCTCAATCACATCGATGAGAACATCTAATGTGGCTATTACCCCACGTCTTAAGATAATTCTTACAAATCAATTATATAAAATTAATTCACAGTTTAAAAaTaGTGTCATTTtCGTCATTTCtGTCATTTTTGTCATTTTCGTCACTGTAGTAGATAAATCTTTTCtGCTAACTCATCACGGCGCGCCAAGAaGTTGTTtCtGTTCAATTTAGAGTTAGGCATCTTCTTGATAATTGCATCTCTGTTATAACCTTTCTTCAACAACTCTAAGAAGCAAAAGTCAACGTGTCCCAATCTCTGTTGTGATTGATTTATAAACTCAACCTcTTTTAACATCTGTGCATATCTTTTATTTGCTCTCTCGAGCCTCACAACAACATCTTCTACTTTACTCGAGTTTtCCCCTtGTGGTTTCGGTAACGTCGCTTGTATACCATACTGTGCGATTGAATTGCTATCATATTCCGGTATTACATCAGCTAACACATTACACTTCATTTTATGTGTGCCTATCATATTAACAATTGACTCTTTGCTATACATCTACTCTGACACCTCCGCCCTCATCAAATCACACTGATCGCTCAACTTTGCGAAGTCACTCGGCGCCTCTACATCATCATTAGCCGTCATCATAATATATACTTGCTCAGTTACATACTTACCTAGCTCATACATCACTAGTAAGAATAATAGTCTTAGTATTTGTTTAATCATTGTTTATCTACCTTCTTTACTTCGTATAAGACTGGATATAAATTTAAAAAGTGTATTCTATAACCAATCGTTTTAACTTCTACTTTGTCGCCTACTTTTAACCTAGCTTGTATGTCTGCGCTATCAAATTTCTTTTTGAATAGTAAGTCTGAGTTTTCAATGACTTGCTTGTTGTCTAATaCAATATAGAACTTGTCTTCTTTATCTTGTCTCTTGTTATATTTATCTGTAATTGTTCCTTGGTGCGTTTCTTTGTGTTGGTAACTAGCCACTGTATAGATAGGAAATGTGACAACAAGTAACAATGCGAATATGCCGAATAATGACAGTACTCCAACAATAAAGATATCGAACCAATCCATATTTTtAAGTTTTTtAATCATCATTGTCATCTCCAGTATCAATTAAACTAGGCATCATTCTTAACATAGCCCTTAATTCATGTTCATTCATATTAGCCATCATAGGACTGTAAAATTCACTGTCTTTATCATTAATTTCTTTAATGAAATCATCTTCAATCTTAGCTTTTTCTTCAGGTGTTTTATTTTTATATTTCTTGATTATTTCAGTGTACTTTTTCGGGAATTTCATTTTAGGTATGTTAATCGTCGTTTGCCTCCTTAATAAaTGTAAATGATTCAATCTCATCTCTTTTAACCCATACTTCATTGTTGAACACATCTTTGACCGGAAGAAAATCCTCAAACACTAGGTtCATAACAAGATTAAtATAATCGTCAGAAGCTAAATCTGTTGTTGTGTAATAAACTCTATCTGAAATaGTTTtAATTTTAACCTCCGTCATTTCCCACACTCCCTTATATTTTCAAACAACTGACTCACTTTAATAATTGCATCCCTTTTAACTTGCGCCTCGTACTTCTCTTTCGCTTCTTCTTTACTCTCTGCCTCAACAACTGTAAACGTCTGATTATCTCTAGCCACaGTAAAATGTTCGTGTGGTAGTCCTGTTGAATCTTTGAATGTTGTGACTAAGTATTGTG

>NRS26contig00060 [organism=Staphylococcus aureus]

TTGAATAGCATGATtGGCGGTATTAATAAAATATCTAAAGCCATTACTGATAAAAATCTCATCAAGCCAATACCTACATTGTCTACTGGTACTTTAGCAGGAAAGGGTGTAGCTACCGATAATTCAGGAGCATTAACGCAACcGACATTTGCTGTATTAAATGATAGAGGTTCTGgAAACGCCCCAGGTGGTGgAGTTCAaGAaGTAATTCACAGGGCTGACGGAACATTCCATGCACCCCAaGGACGAGATGTGGTTGTTCCACTaGGAGTTGgAGATAgTGTAATAAATGCCAATGACACTCTGAAGTTACAGCGGATGGGTGTTTtGCCAAAATTCCATGGTGGTACGAAAAAGAAAAAaTGGATGGAACAAGTTACtGAAAATCTTGGTAAAAAaGCAGGGGACTTCGGTTCTAAAGCTAAAAACACAGCTCATAATATCAAAAAAGGTGCAGAAGAAATGGTTGAAGCGGCAGGCGaTAAAATCAAAGATGGTGCATCTTGGTTAGGCGATAAAATCGGCGATGTATGGGATTAtGTACAACATCCAGGGAAACTAGTAAATAAAGTAATGTCAGGTTTAAATATTAATTTTGGAGGCGGAGCTAACGCTACaGTAAAAATtGCTAAAGGCGCGTACTCATTGCTCAAAAaGAAaTTAGTAGACAAAGTAAAATCGTGGTTTGAAGATTTTGGTGGTGGAGGCGATGGAAGCTATCTATTTGACcATCCAATTTGGCAAAGgTTTGGGAGCTACACAGGTGGACTTAACTTTAATGGCGGTCGTCACTATGGTATCGACTTTCAAATGCCTACTGGAACGAACATTTATGCTGTTAAAGGCGGTATAGCTGATAAAGTATGGACTGATTACGGTGGCGGTAATTCTATACAAATTAAGACCGGTGCTAACGAATGGAATTGGTATATGCATTTAtCTAAGCAATTAGCAAGACAAGGCCAACGTATTAAAGCTGGTCAACTAATAGGGAAATCCGGTGCTACAGGTAATTTCGTTAGAGGAGCACACTTACATTTCCAATTGATGAGAGGTTCACATCCAGGTAATGATACAGCAGTAGATCCTATGAAATGGTTGAAGTCACTTAAAGGTGGCGGTGGTAAGGTTGGCGGAAGCGgATACGAGAATGCAAAAAGAGCTATTCTAAGAGCACAATCAATTTTAGGTGGACGATATAGATCTGACTATATCACTACTCAGATGTTAAGAGTAGCCAAGCGTGAAAGTAACTATCAGGCAGATGCTATTAACAACTGGGATTCCAACGCAAGAGCAGGTACACCGTCTAAAGGTATGTTCCAAATGATTGAACCTTCATTTAGAGCTTTTGCTAAGCCCGGACATGGAAATATTTATAATCCTACTGATGAGGCGATTTCAGCAATGAAATACATTGTTGCTAAGTACGGTTGGGGAGGCTTTAAACGCGCAGGAGATTATGCCTATGCGAATGGAGGTCTTATAACTAAACATCAAATCGCTGAaGTGGgAGAaGGAGATAAACCaGAAATGGTTATtCCGTTGACACGACGCAAAAGAGCAATTCAATTAACTGAACAGGTTATGCGCATCATCGGTATGGATGGCAAGCCAAATAaCATcACTGTAAATAATGATACTTCAACAGTTGAAAAaTTGTTGAAaCAAATTGTTATGTTAAGTGATAAAGGAAaTAAaTTAACAGATGCATTGATTCAAACTGTTtCTtCTCAGGATAATAACTTAGGTTCTAATGATGCAATTAGAGGTTTaGAAAAAaTaTTGtCAAAaCAaaGTGGGCATAGAGCAAATGCAAATAATTATATGGGAGGTTTGACTAATTAATGCAATCTTTtGtAAAaaTCATAGATGGTTACAAGGAAGAAGTAATAACAGATTTtAATCAGCTTATATTTttAGATGCAAGGGCTGAAaGTCCAAACACCAATGATAACAGTGTAACTATTAACGGAGTAGATGGTATTTtACCGGGCGCAATTAGTTTtGCGCCTTTttCATTAGTATTAAGGTTTGGCTATGATGGTATAGATGTTATAGATTTAAATTtATTtGAGCATTGGTTtAGAtCTGTGTTTAATCGCAGACATCCTTATTATGTTATTACTTCTCAAATGCCTGGTGTTAAATATGCAGTGAATACAGCTAATGTTACATCTAaTTTAAAAGATGGTtCTTCAACTGAAATTGAAGTAAGTTtAAaTGTTTaTAAAGGGTATTCTGAATCAGTTAATTGGACCGATAGCGAGTTCTtATTCGACTCTAATtGGATGTTTGAAAATGGAATTCCTCTTGATTTtCACaCcTAAATaTACTCATaCATCAAATCAATTTaCtATTTGGAACGgTTCTActGATACGATAAATccacgattcaagcacgatttgaa

>NRS26contig00066 [organism=Staphylococcus aureus]

aaaaTCCTCCAACCTAAAaTCTTGATTCCATCTTGCTTtAATAAAaTCTTTtGTTTtAGCTgTATTGTGTTtAAAaTGCTTGCCCGcTTTTtGTTTAAGTAAtCGATAATTtCTTTaTAGGGTATAGAAGATGCTGTCGGGTTGCCcGACAATATATCTATTCTATTTATATTGTTATTaCTTGTATTATTAATACTTGTATTATTCTCTTtGaCaTTTGcGTCAATaGGGGTaTTGACAGAATTATCAATAGGGGTATTGATTTTTGCGTCAATAGGCATTGACGATTGCGTCAAGGGGTACATCTTCCTTTGTTTAACTTCATTACCTTCTTTGATAATTTCGATTTTTAGATAACCAAATTTGATAAGGTTCGAAATTCTACGAGATATAGTTTCTTTAACGACGTTGTATAAAGTTGCAAAGTAACcATTACTTGCTGTGCAGTATCCATACTTATTACTTAAAGACGTTATTTCTGCAAAAAGTAaTTTTtCACTATCAGTAAGTCGATTATCATATCTGACATTTGCCGTTATTATTGAGTAGTAACTTGGTTGTTCAGTCATTCTCAGCACCTTCTTTCAGTGCTTTTATTTTGTCCGGTACTTCCCAGTTATTTATGAATTCTTTAAGTTCATCTGTCATAGGTACGTCATTAAGGATTACGTCTGAACCATGTAAATAAAAaTTAATTTtATtAAACATGAGAGCAGTCTCATAAATATTTTTtGACCATCCAATATGATATGTCTTtCTTTTATAAGTTATTTGCGCTACATAACCACTTTGAGTTAAATAGACTCCTTTGAACTTACTTTTtCCTCTTCTAcGACGTTTTTGGTCTTTGTAAGTTTTGTATTCATATTCAAATATAGAGTCATTTTGATTTTTATGATTCTTATAACCTTGTCCGTCCCAATATTTATCTACTGCGCTGTTGTATGCTTTAGCTGCCtCCCATTCATCAACAAAACTACCTAAATATTTAGATTTGCTATCAATTTTTATTACAGCAGACCATTTTTTtGTTTTTCGATTTAAATAAACACCTTTATAGATACTCGAAGTATTTCTTGTAGGCCTTGCCCATCGTTGTTGATAACCAATTGAAGTGATGTTGTTTTtGGTAAAATCATTATTTTTtATTTTTtGAAAACCATTTTCTAATACAAATCCACTTAAGCTAACGTTGAGTGGATTTGTGTGAATTCTTCTAACGTTATCTACATAAGATTTTGTCCAAATATATTGATTAACCCTCTCATAATCTTCATCATCAACAAAAaTTTCTTCTCCATCTTGTAAAAATATCGATTTAACCATTATTCTCCTCCTTTCAGCATTTTGTTGAGCCTCTCATCAaCTTTTAGCCATGAGTCATGCAAGTGATATTTATCAtCAAACGACTTAACGCCAATCGCATGTTGCTCGTTGTGATGTTCGCGACATAACGCTAAtACATGTTTGtCGTAGTGATTCATCTtATTTCtGTTCATGCCTCTGCCAACTGCTTCATAATGTGCCAGGTCTGCGTGAGGCTTTCCGCATATTACACAGTTGCGGTTAATTGTagCCCAATATAATAACGCTTTATCTTCGCTTAACAACTTACTCGTTTCtACACTCATAGGTATTTGATGATGAAACaTAAACGCTATAATCAGTTCTATtAACTCTCTCGCAACTTTCATTGAACAGTCACGCAGACTGATTTCTtCATAACcTTTcaTAaTTtCCAaTTCTGTTTGTAaTAATTTTCTAGTTGATtCTACtGGTTcGCCCCAGTGAAGTTCTATATCTCTACACATTGCGAATATTTTTTtGCGTTGTTCTATAGAtAGTTTTTTATTGTCCGGAACcTCTACTTCTGCTTTTAGCGGATATCCGTTTTCTAGTAAATCAATGTGACTTTGTTCAAGTTCAACACCAGTAGCAACGACGGAATAAGTACCGTCATTGTCTTTCTGGTATCTTGTAATGTATTGCATTTAAACCACGTCCTAGAACGGTAAATCATCATCATTGATTTCTATTGGACCATTAGCATTAGCGAATGGGTTTGATTGTTGACTCATTGGCGTCTGTTTCCCATTTGCTTGCTGTTCTTTTtGTTTCATCTCATCAGTTTTAGGTTCTGGTTTATTAACTACTTCATCGTCTTTATTCCAAACTTTTACATATGAGAGTCTTACAAAATACTTGCCTTGTTCCTCGTTAAATTTATTTTtAAGTACAATAGTTCCGATTTTGTTAATTAATTGATCTGTGTCAAAAGTTAAATCTGGTAAGTTCAATTtAATTCCTAaTCTACTAAGTAACTCGATATATTGTTTtCTtGATAATCTtGTTGGAATGGTGGG

>NRS26contig00077 [organism=Staphylococcus aureus]

cttcatccttatccatcataccttctatAAAAAaGtgTTTgACATCTAGCGCGTTCTTTTCGCAACTTTtCAATATCGATGCCGACATCTTCTATTTCTTTAGGTTGGTTTTCGATTTTATATGATGTTAAATCAAATTGTTTTAGATAATTGTAAAATTGTTTTAAAACCTCGCCTTCGTCGATGTTACATGCATTTTtATTTTtAGTATTTTTGCAGTTAGAACAAAAGTATAGTTTAGAATACCAAACTTCTTTATTTTtAGGCGTATGCTTGACTGTGTTTAAAGTCAATTTCTGGTTACAGTTTGGACATAATAGTTTACTTCTGAAAATAGCGTTATGTTTTACGATTGTAGAGTTAGTTTTTtCACTTATCCTTAATTTTATTTCTTCGTATTCTTCTTCACTTATAATAGCTTCGTGGGtGTTTTCGACGAATATGTCACCGAAAACAAGATGACCTCTAGCTACCGGACTCGTTAGAGCATTGCCTATAACTGATCTGTGCCAGTTTTTACCTAAGGGTGCTTTGTATTTAGAGTTGTTCAATTTTATAGTTATTtCTCTTAAACTAGTACCTTTTTtCGCTTCTTCTACTGCAAATCGTAATACTTTTTtATATTCATTaGGCACAAaTTTATCGTTtACTCTGTCgTAATAgAAAGGAGGGACAGTTTTAGCTAACCCTTTTCtAgCTGATgCGCGTCGACCCATTGCaGTaCGCTCTTGAATtGTAGTACGCTCCCAcTCTGCCATAGCACcTACTAATGTtACGAACAAACGTCCCATAGCAGAAGTTGTGTCATATACTtCtGTTGCGCTCCTAAaCAACACGTTTTtATtCtCAAACAATTCTAGTATCTCTAGTAAGTCTTTAACACTTCGaGTTAATCGATCTAGTTTATAGACTAAAACCAAATCAAAATTATCtATTTCATTCAACATTTCTTGTAAAGCGGGTCTGTCTTTTTtAGCTCCGGAGTAtCCAGcGTCAGTATATACTTTATGAATTTTCCAGTCGTTTATGTCGCtgTAAGCTCTTAATTTTCTTTCTtGTTCTtCGATAGAGtGTCCTTTTtCTTTTTGTTCAAGTGTACTCACtCtAGTATAAATTGCTACTTTCATATGCTCCCTCCTCAAAATTGGCAAAAAatAATAAGGGTAGGCGGGcTACCCGTGATTTTAGTACTAGCTACTAAATGTGATATAATAAAATAAAAAGTAGGTGATGAAATGTGTGTAAAATTTACTGACGCAGAAATAGCTTATATAAAAGAATCAGTTGAAAATTATAGTAGTGAATTTGATATTTATGACGATGAACAAGAACTTAAATTAAAATTTATGAACAAATTATGTTAAAAaTCAAATCTGAATACAAGGATACCTATTTATTCCGTCTTATTAATTGATTTGGTaTATTCTCTTAATATTTTTtCGTTTtCATCAACAATGTCTTTtAGtGTGTTTAAAAGAAaGTCACAATCACCTTTGGCTACTGCACCAGCTTGTGAATGGTTGATTATGTTTCTCATACTATACGCAATTTCTACCCGTTTTTTGGTTCTATAATTTACTTTACCTTCTTTAGTTAATTCTCCTAATAATTTTGtGTACATAGTTGAATCGGTGTCTTTATGTTTGATTTTATtAACTTTTTTtAATTTGaTTAAAAaCGTTTCTATAGCAACAGCAAAGGTTGCTGCAGCTGgCAAATACAATTCCCTTTTATAaGCTTGTAATCCTTGTTCTATTTGATAaGAAAAaGTTATATCATCAACAATCTCTTTCATACTATTTAAATCTAAGTGGTTGAACGGTTGtATTTCATCATGTGCTTTGTTTATCAATCTTtCTTTCGACTTCGATATCAATGTATTGTAATGATCGTTAGCTAATCTTTTGCCATAATTAAAaaTAAATCTAAATTGTTTTGTAATATTACGGTCCCGATATATTTTCCGTAGTAAATAGATGTGTAATAAATGTAATTATTAAAATCTAATAATCCGGATTGTTCTTCTACATACTTTTtAGAATCATAtATGTATGAAGTAAAGTGTTTAGACAAATATTTGATATCAATATTACGAAAATTATATATTTCTTTTAATTTACTGTCATTTGAGATAACGACGATGCAAGGTTCTTCAAAAAAGaTTGATTTAGaTAAAATATCGAAATCTTGTAATCGtCTTTTCTCATGAATGGGAAaGCTTCtGGATTGCTACtAAACTGATAATtGTATCtGTTTtCAACTaCAtATTTGTAGCCTTCtAAAAATACGtGGCGT

>NRS26contig00099 [organism=Staphylococcus aureus]

ATCTTTtATTTTGTACATTTATACACCTCTTTATTTATATTTATCTCTTATAAAGTAGATAaCCTTTTAAGCCGATTTaTTTATATAACTTAGCGATtGTACTTGCTTGATGTTGGCACCACTCTATAGCAGTAGCGTATtGGTGGGTAGCTGGATTCTTAGGATTCCATCTAATTCGGTACAATGTGTTTtGACCTTTATTGATGTAATCCTTTCTTACGAAGCTAGCACCGCCCATGATTGCTTTTGCTGGaGATGTCCAACCTTTATTTTtaGCAAACGTCATTGCATAATCAGGGTCGTTGTCGAATGCACCAATACCGAAGTAATTATATGCACCGTATCTACCACTAGCGAAGTTACTTGTTCCGTATCCACTTTCTAAGAAaGCGTGCGCGATCAAATAAaTTTCGTtAATGTTGTTTTtcttACAaGCTTCcGCGAaTGCTTtGCCTTGTCCGTCGAGCGTTCCTTTtCCTTTAAGtATTTTGTTAAGCGCACTAACTGAAATGCCTTGATACTTGCCTAAATTAAGCATTtGATAGCATTGCGTGTtACTTTCCcATATTCGCTTAACATTCATTGCTGAGCTCGTTTGTGCTCGTGTTGCATTAGCCCAGCCCCATGtATGAGATTTTTtCGGGTtACCCCTAGACATTTGTCTATCcAGTGCTTGCTGGAATGTGAATGGACTTGTTTCAGTAACGATGCTTGGTTTTtCGTCTGATGGAGTAGGGCCTCGTGTGGACGCACTGTCAACTGATGTTTTAtCACTAATTCTTATTGTTGTTTTtGtCGTTACTTCTTTtATATTTTtCGTGTCAATATATCTCGTTTAATGTATGTCTCAAGCATTTtCTTTTtAACTTGCTCATACTTtGCGTTATCCGGTATACCTTGCTTAATCAAGTCGTAATTAATTAAATCTTTCATACTACGCCAAATATTAGGGTCTACCTTTAACGTCGTTtCAGATAAGTTTTtATCAATCCCTGACAATAACCAAACACCACGTATTAACGCTTGTATTTGATTCAATAAGAATTGTCGTTTGCTATCTGTTTGACCACcACATACTTCAATAACTAGCCAATTAGGGTGACGCGGGTCATCAAAATTGGTTGGTCTAGCAAGCCATGTAGCCTCTCTATCGACATATAAATGCGGTATTTCATAATCGcTTATAAACTTATTTCTTTGCGTATACAGTTCGTCTACAGAACGCATATGCATTGATTCTTTTATATATAATCCTTGAATATCTGAGCGTTCATCACCCATTACAACTATATGATCAATGAAATGCTCTTCTTTATCTAAAACATTGCTGTAAGCAGTGTATTTTATTGTTTTAACTTCTTTAAATTGCGGTTTCTTCGCTTCACCAGTAATTGTTGAGTCATTGGCTTTTGATGCTGAACTTGTATCAGTACTACTAGGTTTGCTAGTATCTTTTGAGTATGGAGGCCTAACAAAGCCTGTAACACTtACATAAGGGTGTCTTACTAATCTTCCTGGAGAACCTGTCCAACTATTAGAATtAACCCAGTTTtGGTCAACGCTATAAAAaTAACTTTtATTAGATGGtCCTACTACTaTtGCGGTGTGTCCGTCCGAACCTATTCCGTtGCCAGGGTGCCAAACTGCGATGTCTCCAGGTTCCGGTaCAAATCCAGATGAATAACGATAGAATCGGAAACCCTTAGGATATCTGTAATTAGCCATAtCCTTAGCATTGCCCCATGTTACAAAaCCCCAATATCTTTtAAAAaTAAaGTTAGGTGTATCCCAACATTGACTGCCCCGATAATTATCTATATTAATCCTCTTACCAATATTCGACTTTGCCCACTCCACCACTTCACTAGCTGTAGGCTTTCTAGTCTTTGGGTTAGGTAATCCCATGTATGCACCTCATTTCAATCAAAaTAAAAaGCCAGTGCCGAAGCACTGACTCTTAACTGTTATTTACATTTACCAAACCAGAAGCACGCCCAGAAGCTATATCCTAAAATCCCTTTtAAGCATGGTAATCACcTCCTTTAAATACCAAAAaTAGTTCTTAGTAAAGCTATGACAATCGTACTGAAGATAGTCCCTATCAAaCCAAGAATCCACATTTtC

>NRS26contig00100 [organism=Staphylococcus aureus]

ACTATTTTGTTAATGTTAGGGTCTGAGATTAATCTTTTATATATTTGATTTGTAAGTTCAGGTTCAACTGATACCCACATATTTAACCACCTCTATGAAAAaTACTGCTCGAaTGTCTTGCGTCCTGCGTCAATTGCAGGGTTCCAAAaCGGCTGTGGCGCTTGTCCTTTAGTAGTATGCCATTTACCGTTAGCGTCTTTATAACTCCACGGTATCTTTTTAGCGCGACTACCTTTAGTGGCATAAATACCTGTGCCGTACTCAACATAAACACTATATTCtGCACCTACATtGATAaCTCCTGTtAGACCGTTGTtCTCAAAtCgAAAGTCTATACTTTCTTTCAAAAATCCTAAGTCAGCAGGAGCTAATGCTACAGCAGTGTTATATATCTTCATCGTTGTTTtAGCGATACCTTTTtAACCCACTCTTCTATtttCTTATCGAaCTtATCCAATTCAACAACCATGCTATCAGCACCGTACTTAACTTTTGCCATATGGCACCTGCTTAAGTCGTAGTAACTTAATTTCATGTTGTCCGCCCTGATCTACAGAATCACCTTCAaTACTAAAGATtCTACCCTCaTACtCAAATAAATTGTTTTtAGATATTGGCAAGTCATAAGGTACATATAGGTTTCTGTCATATTCTTGTGACATTTGATGAAATTTtAGTTGTTCAGATGTAGTAGGCGTATCCATAAATCCTTTAATTGTTTtATCGCTTACAAAGCGCTCTTGTATAATTGGATACTCTCCtACTTTTTtGATACTTCCAATAGAAaTAGTGTGAGGGAATTCGTCGTATGGGTTAAACACAAACAACACCTCtACCTTATTGGTTTAAACGGATGAAACTTTGCTCGTTTATACCTGTTTAATACTCCACTAATGTAATCAGGGACACCATCGTTATAAGTGTACGACACTGTCCCCATACTTCTTGACTTTAAATTCTTTTtAACTTCAGGTCGTTGATAATACTCTAGGACGTCTGCGACATACTTTTtGATTGAGTAAGGATAAATGACTTGACCATCTTTCATAAAATCATTGTTTGTTATATCCCTAACATCTTCTAGTATTCCGTCAACTTCCATCTTAAATATTTCTtCTtCATCACTTTtAaCTTCCACTCCATTTTTCTTGaGTAAAAGTTTAACATCTTCATAAAGAGTCATTTTTATCACTCGCTCTTATCAGACGTAGTACGGCGTGATTTAACCTCTTTGTAACCGACAAGACTGTAATAAGAGTCAAACGCCTTCTTTGTAACAGTAATAGTCATATTGTCTTTTTTACCTTAATCTCTTCTGCAGGATTAGCCATCATATCTCCTCCTATTCAGTTGGTTTAAgCGTTGCGAACGCTTCTGGTTTAACGTTCATGTATGCAATATGCATCGTCGCACGTAAAGCGAACATATCACGTTCAAATAATGATACTGGTTGGCCAGAAGCATCTGATGCTTGTAACGTCGTTAACGTGGCATCTTCAGAAATTGCATACTCAATACCTTGTAAGATACCGTAACGTGCGTAATCCCAATCACCCATTAGTGCTAACGATTTCTTTTGTCGTATACATCCGCTCCAGTATAAGATAGTGGTAATCCCATAATCTCGTtCCCGTTAGCATCAAATAATGGTCTGTCATTAGCATCTAAAGCATTACGCATTTTACTtCTGAATGAaCGTGTAGTTAaTACTCCGTTTGGATCTAACTCTTCATCTTCAATAGTAGCCATTAATGCCGAAAGGTCTACGTATAAATTATTAGTATCTGTAACAACGTTACCTTTCTCTTCTGCGCCTTCAACAAGCGGTTTACCACTAGTTGAAGTGTTGTAAGGTGATTTAGTACCAAAGATAACAGCTTGGTCAAACGCTTTGTAAAATGCCTCTGCAATTAGAGGTTTAACCTCATTAAAGAAATCTTTTGCAGTCCATTTAAGAAACTCTTTTGATAACGGAaTAATTACACCAATTTTCTTAGCTTCCATTTCTGCTTGTGCATATTCAGGCTTAGAaGTTtGAATACGTTCcGTTtCTGATACCCaGTAGgCGCCTACACCTTTTGCTAAGTAaGTAAATTTttCTTTtGTGCTGTCATTGGCtca

>NRS26contig00138 [organism=Staphylococcus aureus]

gacagaaggtaaagatacgCTtCAAtCAtCGAAgCATCAATCAACaCAAAATAGtAAAACAATCAGAACGCAAAATGATAATCAaGTAAAGCAAGATtCTGAACGACAAGGTTCTAAACAGTCACACCAAAaTAaTGCGACTAaTAATACTgAACgTCAAAATGATCAGGTTCAAAATACCCATCATGCTGAaCGTAATGGATCaCAATCGACAACGTCACAATCGAATGATGTTGaTAAATCACAACCATCCATTCCGGCACAAAAGGTAATACCCAATCATGATAAAGCAGCACCAACTTCAACTACACCCCCGTCTAATGATAAAACTGCACCTAAATCAACAAAAGCACAAGATGCAACCACGGACAAACATCCAAATCAACAAGATACACATCAACCTGCGCATCAAATCATAGATGCAAAGCAAGATGATACTGTTCGCCAAAGTGAACAGAAACCACAAGTTGGCGATTTAAGTAAACATATCGATGGTCAAAATTCCCCAGAGAAACCGACAGATAAAAATACTGATAATAAACAACTAATCAAAGATGCGCTTCAAGCGCCTAAAACACGTTCGACTACAAATGCAGCAGCAGATGCTAAAAAGGTTCGACCACTTAAAGCGAATCAAGTACAACCACTTAACAAATATCCAGTTGTTTTtGTACATGGATTTTTAGGATTAGTAGGCGATAATGCACCTGCTTTATATCCAAATTATTGGGgTGGAAATAAATTTAAAGTTATCGAAGAATTGAGAAAGCAAGGCTATAATGTACATCAAGCAAGTGTAAGTGCATTTGGTAGTAACTATGATCGCGCTGTAGAACTTTATTATTACATTAAAGGTGGTCGcGTAGATTATGGCGCAGCACATGCAGCTAAATACGGACATGAGCGCTATGGTAAGACTTATAAAGGAATCATGCCTAATTGGGAACCTGGTAAAAAGGTACATCTTGTAGGGCATAGTATGGGTGGTCAAACAATTCGTTTAATGGAAGAGTTTTTAAGAAATGGTAACAAAGAAGAAATTGCCTATCATAAAGCGCATGGTGGAGAAATATCACCATTATTCACTGGTGGTCATAACAATATGGTTGCATCAATCACAACATTAGCAACACCACATAATGGTTCACAAGCAGCTGATAAGTTTGGAAATACAGAAGCTGTTAGAAAAATCATGTTCGCTTTAAATCGATTTATGGGTAACAAGTATTCGAATATCGATTTAGGATTAACGCAATGGGGCTTTAAACAaTTACCAAATGAGAGTTACATTGACTATaTAAAACGCGTTAGTAAAAGCAAAATTTGGACATCAGACGACAATGCTGCCTATGATTTAACGTTAGATGGCTCTGCAAAATTGAACAACATGACAAGTATGAATCCTAATATTACGTATACGACTTAtACAGGTGTATCATCTCATACTGGTCCATTAGGTTATGAAAATCCTGATTTAGGTACATTTTtCTTAATGGCTACAACGAGTAGAATTATTGGTCATGATGCAAGAGAAGAATGGCGTAAAAaTGATGGTGTCGTACCAGTGATTTCGTCATTACATCCGTCCAATCAACCATTTGTTAATGTTACGAATGATGAACCTGCCACACGCAGAGGTATCTGGCAAGTTAAACCAaTCATACAAGGATGGGATTAACTTGTGTTAAAAAGCCTTTAATATCAGTtGTTACAAAGGaTTTGTAGCGTCTTTAAAAATAAAAAaGGGCAGAAAAAGGGCAGATACCTTTTAGtACACAAGTTTTtCtAATTTTTGCTCTAACTCTCTGTCCATTTTCtCTGTTACATGTGTATACACCTTTATAGTCGTTTTTtCATCTGTATGTCCTACTCTTTTCATAATTGCTTTtAACGATATATTCATTTCCGCCAATAAaCTTATGTgTGTATGCCTtAGTGTGTGaGTag

>NRS26contig00163 [organism=Staphylococcus aureus]

ACGCCACGTATAGAAGTGTaCTTTAAAGCAGTTCGtAGTGGTTACTACACTATAAATGACATTAGAGAgTGGGAAGATTTACCACCAGTTGAAGgTGGAGATAAGCCGCTAATAAGCGGTGATTTATACCCAATTGACACGCCACTTGAATTAAGAAAaTCTTTGAAAGGTGGTGATAAAAaTGTCAATGAAAGCTAAGTATTTTCAAATGAAAAGAAAATCAAAAaGTAAAGGTGAAATATTTATTTATGGTGATATTGTAAGTGATAAATGGTTTGAAAGTGATGTAACTGCTACAGATTTCAAAAaTAAACTAGATGAACTAGGAGACATCAGTGAAATAGATGTTCATATAAATTCATCTGGAGGCAGTGTATTTGAAGGGCATGCAATATACAATATGCTAAAAaaTGCATCCTGCAAAAATTAATATCTATGTCGATGCCTTAGCGGCATCAATTGCTAGTGTTATCGCTATGAGTGGTGACACTATTTTTATGCACAAAAATAGTTTTTtAAtGATTCATAATTCATGGGTTATGACTGTAGGTAATGCAGAAGAaTTAAGAAAGaCAGCGGATTTACTtGAAAAAACaGATGCTGTTAGtAATTCAGCTTATTTAGATAAAGCAAAAGaTTTAGATCAAGAACACTTAAAACaGATGTTAGATGCAGAaaCTTGGCTTACTGCAGAagAAgCCTTGTCTTTCGGCTTGATAGATGAAATTTTAGGAGCTAATGAAATAGCTGCTAGTATCTCTAAAGAGCAATATAaGCGTTTCGAGAACGTCCCAGAAGATTTAAAGAAaGATGtAGACAAAATCACTAAAATTGATGATGTAGATACATCTGAATTGGTtGAAACACCTAAAGAAAGTATGTCACTAGAAGAAAAAGAAAAAaGAGAAAAAaTtAAACGCGAATGCGAAATTTTAAAAaTGACAATGagTtaTTAGGaGGAAaTGAAATGCCGACATTATATGAATTAAAACAATccTtAGGTATGATTGGACAACAaTTAAAAAaTAAAAAtGATGaaTTGAGTCAGAAAGCAACAGATCCAAATATTGATAtGGAAGACATCAAACAACTAGAAACAGAAAAAGCAGGTTtACAACAAAGATTtAACATTGTTGAAAGACAAGTgCAAGACATTGAAGAgAAaGAAAAaGCGAAAGTTAAAGATAAAGGAGAAGCTTATCAATCTTTAAGTGATAATGAGAAGATGGTTAAAGCTAAGGCAGAGTTTTATCGTCACGCGATTTTACCAAATGAATTTGAAAAaCCTTCAATGGAGGCACAaCGTTTATTACACGCTTTACCAACAGGAAATGATTCAGGTGGAGATAAGCTCTTACCAAAAaCACTTTCTAAAGAAaTTGTTTCAGAACCATTTGCTAAAAACCAaTTACGTGAAAAaGCTCGTcTAaCTAACATTAAAGGTTTAGaGATTCCAAGAGTTTCATACACTTTAGACGATGATGATTTCATTACAGACGTAGAAaCAGCAAAAGAaTTAAAAGCAAAAGGTGATACAGTCAAGTTCACTACTAATAAATTCAAaGTATTTGCTGCAATTTCAGATACTGTAATTCATGGATCAGATGTAGATTTAGTAAACTGGGTTGAAAACGCACTACAATCAGGATTAGCAGCTAAAGAGCGTAAAGATGCCTTAGCAGTAAGTCcTAAATCTGGATTAGAACACATGTCATTTTATAATGGATCTGTTAAAGAAGTTGAGGGAGCAGACATGTATGATGCTATTATTAACGCTTTAGCAGATTTACATGAAGATTACCGTGATAACGCAACAATTTATATGCGATATGCGGATTATGTCAAa

>NRS26contig00165 [organism=Staphylococcus aureus]

CCAGTGACCTTATTACGACATGGAGTTCTACTCAAAaTAGTATTGAATCAATGGCGTCATTTTtGAACTCATTTCCAATGTTTAAAGATGATACGCGTAACACACATCCTAAGTTTGTTGCCAGTGCCACATATAACTTTTCtAGTGGTGAAAGTAAATCGAGAAGTAATATTAATTTAACGCTAAATGCTAAAAAaGAaTGGCGAAATATTTTAATTtCTACTGGTGAATCATCTATCGCAAATATGGCTGATGAAAAAGCGGGTGTATCAGCACGTGTAGTTACACTACAAGATCCACCATATCCAGATAATTTTGATTTTACCACATTAGACAAATCGTTtAGGGAGAACTATGGAACATTAGGGTtGGCATTTATtAAACAaTATGAGTCTAAAAAaGACGTGTATAaGAACGCTTTTGAGAGCTATCAACGGTATTTTAATCAAAAAGGTAGTAATGAAaTCATGCAACGTTTAGGACGTGCCTTTGCGTTACTACAAGTTACCGGTGAGGTTTTGAATGATATtGATGGGTTTGAACATGACCATTTTAAAATtATCGAACAAGCCTATGACAGCATGGTTAAAAACAaTAAGaCGATTGAtAAACCTAAGCAACTGTtAGaGGAACTATtACAATATTTAGATGCGAATAGAAATAATATTGTAGGTGACGGTTATGATTCAGTAAATTACGGAGATGTTAAAGCGGTATATAAACATGATTTTCTTtGTATTAAAAaCGAAACTGTTAAAAATAAATTAGGACACGAAATGCAGACGATTACAGGGCAATGGGACAAAAAaGGTTaTTTAATAAAAGATAAAAAAGAATTCAAAAACAaGTAAAACATAAaTCTCAAAGGCATCTGGGTTATGCGATTAAAAAaGAAATAATTGAAGAACTAGGATTTGATTTCTCGATTTCGCATAATCCATATACAGAAAGTTATTAGTACACACAAGTACACACTCAATTTTAAAATATGTGTACTCGATAAATTCAATAATATCAACAGTTACATGCAGATAGTACACGAAGTACACAATGTACACATATAAATATATAGTCATAGTTAAAATTAATAAATTAATTATCGTAGATTTCTAAATAATATACAACTATCACTAAAaTTTCTGTGTACTTTGTGTACTAATTAtCACGAGGCTATATATATCAATGTTTTAACCAGTACACGAAAGTAAAaaaGGTGTGTATTTAACTGTGTATGGTACACAATCAATAtAAaTATGGAGGTTACACATGGATAAAGAACAACTTAAaaGTATATATACGAATATGTGAAAGAATATAAGGAGATACCGATATATCAGTTAGAAGATTTGTTTAAAGAAaTAAATCACGACTATATAGGGAGAACTAGTATCACACACGATAAGGATGAGAATATTGTGTTTTGGAGTGGGTGgAACAAAaTTACAATGTTTGCGTTGATTGAATTAGTtAAAaGTGAACAACTTGATTTAGTGTATAGAGGTAGTTTTAtAATGCGTTATTTGTTGGATGGTAGAGTTCCTAATTTACCATTAGCTATTTGTTATCCAGAAGATGGACAACAAACTGACGTGCCCTCATGGGTGCCTATGGTATTAAGAATAAATAAAGAGGAGAAAATCAAATGAACATAGAAACTATCGTAAATGAATTTGAGACGCGAGCAGGCACGCTATTAAGGTACTACACGGGATTATTAGAACATAGTAAAGTACAACCATGTTGCTTTAAGTTATATAATGATCCATTTGATATGGTTTATGTGGTGATGAACAACAAGTTATTCGGTCATGTATATATTAAAGATTGTAAGGTGAGG

>NRS26contig00175 [organism=Staphylococcus aureus]

ACCGTTaTAaTAAACTTcTCcTGaGTTAgTGTTgTAAAATGTCATTTgACGCcTCTtATCACcTTCgTTTgtAGgCAATCTATcaGgTaCCGACCATTTTTCAGGGTCgTTATTACTTtCAAGATCAGTACTATAACCGACACTTTCAAAGTATGGTAgTTCGGTtGTTtCAAACGACAAaGAAAaTTCCCCTGAtGTTTGTGTTGTGTCAAAaGAAACTTCACTTACTAGTCCTACAAAAAGTTGTCGTCCATCAACaTAATCAAGCTCAAATGCTTGTTTGTCTTTTGGTATATCTAATATATGCTCATACTTAATTGAATTGTCTGGTGTAGCTAATTCCCTTAAaTAAAAaCGTCCAGCAAATAGTGCTTGGACGTCTGACTTTAAATGTGAAGCATAAGCAATTTTAGGTACTTTATACCTTATCTTAAGCTCTACTTTTTtAAGTTCTTCTTTAGCGTAATTATGAAATCTACCATCAATACCCTCTATATCAGAATAGTTACGATGATATCCTGCGCCTGTAACGTTATATTCAACTACTTCCAAGTGATTATAAGTGAAAGGATTGTCACTGACGCGATACTGCGAACCATTCCTTATTACTTCTATATCGTGCGCTATCAACTAACAAACCTCCCTTATAATAAGTTGAAACTTCCGTCTATAGCGTTCATGTCATCAATGCGTGATTTAATTAAATCAAGGTCGCCCTCATTTCTAATCGTTACATTCACAATAGGTCTATTATTTTCTTTTAAGCTATGTTGAACATCGCTAGTCATGTGTCTGTCTATAGAAGTACTTACAGGATCTACTATACTATCTGTCAAAGTAGAGGATAGCTCTTTATTAAAGGCACTGCCAAAGTCTGTAGCAATTACTTTTGCTTGCGATACCGCTAAACCTTTACCTAAGCTACTACCTCCACCGTGTCCACTTACGAATGAAGTTACAGAGTCCCAAGCTGATGAAATCGCATCGCCTACCGCGCTGACTACTTTGTGCGCAGCATTGGCTACACCCTCAGCTACTTTGCCGATTAATTCCGCTCCGGCATTTAAGAAATCACTGAAGAAaCTTTTAATCTTACCAAGTGCATCACTCATACCGTCACCTACATTTtGAGaCAACTCTTTTAAACCCATCAGCtACTTTACTCGCGAAACTTGtAACTGtATtCCAAATGTTAGAAaCCCATTCAGAACCTTTTGTgATAATAAAGTTTAaTGCTTGTCCcATtttttCAGCTACACTCCAAGCAACACGACTGAACCAACTTGTAACAGTGTTCCAAATACTGCTAACAAAATTAGTGATTGTACTCCATATCTGTGACCAACTTGTACCAAACATTGAAAGCGTTCGATTCATTACGCCAGTTAAAAaGCCGATAATTGACTCCCAAACTGATTGCATGTATTGCCAAATCGTATCAAGTACATTGGTAACCGTAGTTTTAATAGTcTCCCAAGCACCTGAGAAGTCGCCAGTAAGCAACTGAATTAAAGCAGTGAACAATCCTACTATGATTTGGACTGCTACAGATATCACTGTTCCTATAGCTTCGAATGCTATTTTTATCACTGTCCATAATCCTTGAATGACATTCATTACGTTTACTATGACACCTATCACTAAAACGCCTAACACAGTCATAAAAaCTTTACCTAACGCTTCTAATATCGGTTTGATTGGTTCTATTGTGGTCtGTATTTTATCCCATAGTTCACTTAACCAATCAATTACGCCTTGAATCGCTTCAGAAACAGCGCTTTTGATATCTTCCCACGCTTTTGTCATATTCTTT

>NRS26contig00180 [organism=Staphylococcus aureus]

tttcgccattcttcaatggactttacatttgaagggtnctaaatcttgatttcgtgattctttaacatctcttCCAcTCGATTtAAATTtaGACTCAaCACCTTttGAACATACTTGtCAAAGGTTCTTTtAAAGCTTTAaGTTTTGCTCAGTATCTTCATCAGAATCGCCTAAAAATCTATCAACtAaGGATGTTGGTAAATTTAGTTCCTGCGCTTTACCTAGCGCGTTACTtCTTAACTtCTCACGTTTTGCCTCTGCGTCGCGTTTTtCTAACTCTTGTTCAAGAGCACTAATACGTTTTTGTTCTTCTGATTGCTCAGGATTACGCTTCCGTACTTCTTGTTCGATTAGATCCTCAAGATTTTtCTCTTTCCATGATTCTAAtCCTTTCGAATGATAACGATCTAATTCAGGTTGAATGAATCGTTTACCTTCTTCTGTATCTAAAAAGCCTTTAACGTCATCAACAGACACCGTCTTAAGTCCGTTTAGATAATCTTTTACTTCTTTATCGTCTTTGTGTTCTTCAAAAAaGACTTAACTTCTTCGATATTCATATATCAAAACTCCTTTTTGCCCTTCGCGTACCCTAACAGTCCGAAAAGTGCATAATAAAAAGCAGTTTAACGACATGCTAAGGTCGAGTAGCAAAGAGACAaCTAAAAAaGTGTGAAATCATTATTTTTAGCATTTTCTTCGCTAATAGATGTTTTAACCATATCTAAATCAGCTTCATTTTtAACTGTTACGTTTACAACAACTTTTtCGTTTtGTAACTCTATTATCTCTTCGTACAAGGATTTAATGTGTTCTAACTTTTCTATAGCTTCGCCAGTATCAACATTTACTTTtATTTTAAAATCCATATCAATTACCACCTTTTCGCTTATATTTCTCCCACTCACGATAAGTCATGAATGGGATAACTTCATTTTtACCATCGTCTTTACGTGCTCTCATTACAGTTGGCAATTCATTTTCATCAATATAATAAAGTAaTTTGCAACGACAaTTAATATTCtCTTTCGCACtGTTTACACCAaTAAaTAGCTTGGGCGCCTGCCCAACACACCCACTTGATTTAAAATTCTGATCTATTTCCACTGATTCCCCATCTAAATGACGATGAGTATCACGTGTTCGTGTATCTTTAGTAGCATGCCAACGTTtCTTCATCTTCAAACCGTTATCTTTAGCAACCATTGCGCTATCAAGTCCAGCTTGTGACATTGCTCTGCCTGCTTCTGTACGAGCCACACGCAATGATTGAGCTTTAGACATGCCGACATCATCACGTATTGCTTTAGCTATCTTAGAGTAACCCTCTCCACTCATAATACCTTGTGTAATGTGCATACGTAtCTTTTtCAATACTTCATCACGAtGTTTTtGTAGTGTTGGCATTAAACGAATGAACTCAATAGGTTGTTCAATAGCTGATTTGATTACCTCTTTACTCGGAACATCAAACTGCATAGATGTTTGACTCGCCATTTCATATAAATAaaGGCTCATAAGGAATTTTTCTATATAAGCATCTTCTTGTGACTTCtGAATCATCTTAGCTACTTGCCTATAGTCATCAGTCAACATTGTACCTATACGAGTTAACTCCTTATTGAGCCTGTTGTATTTATTGAATTCAGTCCATGTAACATACACATCATCATTTTGATATTTCtCAAACaTATCTGCGATGATTTGTTTtAtCTCTTtAAGtCGATtAGCAAaTgTTGTTCTATtGGTTTTTCTGCTTTAGAGATTAAACCcTcGATATACTCATCAATAtCATtCTGATTGGTTA

>NRS26contig00189 [organism=Staphylococcus aureus]

CTTGATGTcTCtAAtaTTTttaGCaTTTTtCtcTTATTTTTtCATCTcTtCTTtGTCACGCCTtAGTTCTTCGAAATTtCTATCTAACTtGTCATAAATTTTTTCTTGCGTtCTCAGACTGTCTTCTATTCTGTCGAATTTTTCAAACATAGtCTTATCATTTtCTtcTAATCGCGTTAAAcGCCAATCTtGTtCGTGTCGTTTGGTAAATCCAAACATTACACcACCCACTTTATTCAAATTAAAAaGCCATAAGATTATAACCTATGACTCTAGATTTTCTGGATACTTTTCTCCTGTAATAATTGCATATTCCTCTTTATCTATAACTTCCATATCTACATACCACGCTATATCTTCTTTACTATATTCTTTCAATTGATACCATGTTTTAATATCTTCGAATGTTGGTGAAATTAATTTAAGCATTTTCAGTCTCTCCTTtAaCCTCTTCTAATTTTTtATTAAGTGTCACAAGTTGTTTtGCCATTAGTGCATTTtGCTTATTAACTtGCATCGATAACTTtGTACTTTGAACAACTTGTTTCTGCATACTAGCAACCATTTTTCGTAAGATGTCATCAGAAGCGACTGTGTTTtGTTCTTCACTGTCAATCTGTTGATGCAAGTCATCTTTTtCTtCTGAATAATCTTcGTTAAAAACTATTTCCCCATTTGAATATTTAAAGGCTTTAGGTCTAAAAaCTTGAGAGAAATTTtCTGGTAAATTTTCAATATCAATACCTTCTTCAAAGCCACCAATGATAGCGTATGAAATTATCTCATTACGCTTGTTAACTAATATTTGCATTATTTTCTCACTCCTATAATTTtGTTAATTGTCCCTCtATTTGCGTTCGCACCAGAGCCTCTTTGACTtCCTAAGTCGAAATAGACATCGTTTGATATAGTTAAAGATGTACGACTAGATTtAGTtAATCCAAACTCaTAAACACCTCcACCATTTCCATcACCATCTagAAGATTTgAGGGATTCAATGAAATCTTTCCTCCTCCAAAaGGACTGCCAAACTCTGTAAAGTCACCACCTGGAAAAGTCCCATAAAAaaTTAATAAAaTAAATTGGTCTAAACTCTCATTTAAGTACAATGTAGAGCCCACACCATTTGCTGTTCCATCAAAAaTAACCGAATACCTTTTATTAAACTTGTCATCTGCGTATAATTTAGCGTTACTTTCGGCCATATTAGCTTTTGATTGGGCACTTTGAACAGTTTCAAAAGGTGTATTGTAATCATTAATAGCTAATTCTGACCACTCAGACCATGAACCCGCTTCTTTTCTTTTAACAAACaCTTTATTTGTACCGTTCGGTCGATAAGTCATACGCTTGTAATCTGAAGTTACTACTAAaTATTCGACAGTACCGTTAGTACTAACACCTCTTGGATAATTTATAGCTTGCGAAACATAAATAAATTGGGTTGAATCACCTATTCTTTGTTCTGGATTATtAAAATCAAATCCAGTAATCTGCATTATCTTACCATCATCTTTGgTAATCTTAGCTTTTtGCCAATTTGAAGTAGAACCACTTGTGACTAAACCACCACTATTCACTGACTGCTTGAaGGCTTCATGTTTCTCATCCATATATCGCTTTTGCTCATCGAaTGTTCTTGAATATGCTTGCGCTTtATTTtCCAAATCAGATATAtGGCTATTAGCAAGTTGCTTTAATtCATCtaTACTTGAAGATTTTGCTataTTTGAAtaTCTGATAGtACCTTTTtcTTtAGCTt

>NRS26contig00225 [organism=Staphylococcus aureus]

ATTTCtCTTTtCTTTtACGTCTGACTTTCACTAAGtCCTCATATACCATCCATTCTTGACCTGTGTATTTAGGCGCTTTACATATCCACGTTAAATTCACATCTCTATACTGATATCTGAATATCTTCGCTTtGATGTTGGCAACTTCAGTCGCCTTACCTTTAACGTCtATAaCTtCAACCAGTTtCCcTtCCTTCCACAAaGAGAAATCGGCTATATACGTAATCGGTCTTTGTTTCCcGAATTTAGGTTGTAATTCAAATTTCGGTTGTATTTCTATATGATCATAATTAGTGTCACTCATATTACTTTCTAAATATTGGTAATATTCACACTCTACTTTGCTATCAAATACAATTCCTTTGTACTCAACTTTCTTAGCGTTGTATTTACTCATTGTGCCACCTCTAAATATCAAATATCGTTGCTTGTAATCCTAGTTCTTGCTCATATAAAAGCCCGTGAGCGCCTTTGAAGCGTTTCAGGTCACTATCAGTCATAATTTtCTTTTCGTCGCTGAAATGGGCTCCTGTGAGCGAATAAACTTCATTTACGTTGTCTTTATACTTGATGACCTTAATATCTTCCGTGCCATCTTCTCGGTATAAGTAATATTTTTCTTTCGGCATTTTtAACACTCCTTAATATTCGACGATTGCGGGTCTTTCTTCTTTTtCTTTCAACTTATCATCAATAaGTTTTTAAGTTtCTCTTGGTCTCCGTTTGCAAAATCAATCATCTTTTGAGCATATACATCTCTACAATGTAATATTTCTTTTATATTTTGTTTTGTGATTACCACGCATCTCGCTCCCTGAAATCGTCTCCGATTACTCTTACTTTTCTTGCTCTTTTTTCATTCTCGAATTTATACGTTGCCAGTTCATATTTTGATTTAGTTCTTTATCACTAAAGTTAGTTGTAAAGATGTTGTTTTtACCTACTCTGTTATCAACAATGCTGAAAAGTTTATTTATAGTGTGTTCTGTGTTTTCTACACCCATATCATCTAGTACAAGTAAATCAATCTCACTAAGTAATTTGACTAGTTCGTCTGTAGTCTCTACTGCATTTTtGTTGTATGTCGCTTTGATACGATCCATCAACATTGGTATATGCATAAAAGCAaCTGTATGCCCTTTAGCTTTAACTGCTTTTGCGATAGCGTATGCTAGGTGGCTTTTACCAGTTCCATATGAACCTTGAAATATTAATGATTTtGGTTCTTTTGTAGAGAAACCCTGTACATACTCTATTGCTGATTGTTtAGCGTGTACTTGTTTTCATTTTGTGGCTTGTAGTtGTTTACTGTTGCATCTCTTAAAGaCGGATTAACGTTTGATTGATTGAATATGTTGTTTATCTtCCGTTGCTTGTTTCGCTTATATTCCTCATAGATTTCACATTTGCAACCGTCTTTATACTCGTAACCATCCGGGTGTTTTtAGTAGGAGCGAACTTATATAAGTCGTATTCACTTCCACATCTCTCACATTTCAATCCTTTttCGACATGAGTAGGTTGATATTTTtCAAACTTTCGTTTATCTTTTCACTGAATAGTGGTTTCATAATATCCCCCTAATCCCAATAACTTTCGTCGTACTTCATACGTTCCAATTGATCTATGCCAGTTGGTTCTGCTTTTGATTGAGGTATCCCTCAAATTTATTA

>NRS26contig00229 [organism=Staphylococcus aureus]

GAaCAAAGTGATGACCCTAGAGAAaTAgAAGATCGTATCAAGTTAATGATTcGTCTAGCTAACCAATTTTAAGgAGGATTTAATCAATGGCAATATTAGAAGaTATTTTtGAAGAaTTAAAACTATtAAATAAGAATTTACGTGTGTTAAATaCTGAACTATCAACTgTGGATTCATCAATCGTACAaGAGAAaGTTAAaGAaGCACCAATGCCAAAAGAaGAAACaGCTcAACTGGAAACAaTTGAaGAaGTTAAGGAAaCGTCtACTGATTTAaCTAAAGATTATATTTtATCAGTaGGAAAaGAGTtCCTTAAAAAAGCAGATACTtCTGATAAGAAAGAATTTAGAAATAAaCTTAACGAACTTGGTGCGGATAAGCTATCTACTATCAAAGAAGAACATTATGAAAAAaTTGTTGATTTCATGGAAGCGAGAATTAATGCATGAAGCTAGATCACTCAAATAGAGCTCATGCAAAGCTAAGTGCAAGTGGTGCGAAACAATGGCTAAACTGCCCACCGAGTATTAAGGCAAGTGAAGGTATTGCAGATAAAAGTTCAGTTTTtGCTGAAGAAGGTACATTCGCCCATGAATTAAGTGAGTTATATTTCAGTCTTAAATATGAAGGCCTAACACAGTTTGAGTTTAATAAAGCTTTTCAAAATTATAAGCGAAATCAATATTACAGTGAAGAGTTGCGTGAaTATGTTGAAGAGTATGTAGCTAATGTAGAAGAAAAaTATAACGAAGCTTTGAGTAGGGATAATGATGTAaTAGCTTtATTtGAAaCAAAaTTGGATTtAGGTAAATAcGTCCcTGAaTCTTTTGGTACTGGTGATGTCATTATATTTtCAGGTGGTGTACTTGAAATtATTGACCTTAAATACGgTAAaGgCATTGAAGTTTCAGCTATAGATAaTCCTCAACTTAGaTTATATGGCTTGGgCGCATATGAACTGCTtAGTTtAATGTATGACATtCATACAGTTCGCATGACTATCAtACAACCACGAATAGATAAcTTttcTACTGAAGAGTTACCAAtATCAAGATtACTTCAATGGgAACCGATTTtGTTAAaCCATTAGCCAGACTTGCTTATAACGGTGAAGGTGAGTTtAAaGCAGGTAGTCATTGTAGATTCTGTAaGATAAaGCATTCATGTAGAACACGTGCAGAATACATGCAAAATGTGCCtCAAAAGCcACCACATTTGTTAAGTGATGAAGAGATTGCAGAACTTTTATATAAACTGCCTGATATCAAAAAATGGGCTGATGAAGTAAAACATTATGCGTTAGATCAAGCGAAAGAAAATGATAAAAACTATCCTGGGTGGAAGCTTGTAGAAGGTCGTTCGCGAAGAGTGATAACTGATACAAAAGCAACGCTTGAAAAGTTAGTTGAAGCGGGTTATAAACCTGAAGATATTACAGAAACCAAGTTACTTAGTATTACGAATTTAGAAAAaTTAATTGGTAAAAAAGCATTTTCTAAAATTACAGAGGGCTTTATAGAAAAGCCGCAAGGTAAATTAACACTTGCTACCGAGTCGGATAAACGACCAGCTATAAAGCAATCTGCTGAAGATGATTTTGACAAACTATAAAAaTTAAAAAGGACGGTATATAAACATGAAAGCAAAAGTATTAAATAAAA

>NRS26contig00286 [organism=Staphylococcus aureus]

TTCTTCATCTTCACCATTGAtttCTCgaaaTATATCTTGTAAGGCttttGTATAAGttttAGTACTCATCTTGTTCAGAACATCTTCTTCAGTCAAtCCTTCATCTTtAAATAAaTCTaCTAaTAaCTGTCGCTCTTTTTGTCTCATTTTTGTTGCGTTAGGTGCTtCTTTTTtATTCTCTTGATTTACTAATTCTAAATACTCATAGCATTTTTCTGCTTCGCCCATTGTTACATCTTCTTTTGtATaGCTCTCTGTTTTtCCTGTTTTACGATCTTTAATTTCAAATTTAATCATTGTATTAGCTCCTTTtATTCAAATAAAaaGACGCAGATATACTGCGCCTTAAATCCCTATCCGTTTGTTACTGTCACTGAAATTTGTCCTGACTTATCGCTTCCATCAGTAGACATAGCAGTGATTACTGAAGTACCTTCAGCTACACCGTGAATTGCTCCTGTATTTTCATCTACAGTAACAAATTCTGGATGTTCACTTGTATATTTCAATATTTTATtCGTTGCTGTGCTTGGTGCAATGTTTGGCTCAACATTGTCATCGGTATTTACCATAATTGATTTAGTTTCTGGTGTAAATGATACGCCTGAGACTAGAaTtGGATTGGTTTTGAATTGAGgTACATCAACTTTACTAGATTCTTTACCATTTTCTTCCCATGCCACTTGGTAAGTACCTTTTGGATAAGTTGTATCCGCTTCTAAATTAGaTAAAGTTACTGACACTTTGCCTTCACCTTGTTCAGAAGCTACGACGTCGTCTCCTTTATAAACCTTTAAAGTTTTAGTCATAAATTATTCTCCTTTGATTTATTTTGAAAGCCCCTATTCTGCTGAAACTGTTGCAGATTTTGAATTAACTGCTACTTCAACATTTTGGGGATTAGCTGGGTAACGAACCtGCAGAATCCTCTGAATGATCTTCACTGTCCGTGTATCCAACGAATACTTTTtGAAGAATTCTGCTTCTCCTTCTTTACCTTCATGATAACCGTATACAATACCTTGTGACGTTCCATCAaCATCAACTTTTCTATTCATCCAGTCaCCTGTTAATTTTGTAGGTTCTGGGGCTTCTGCTTTTTCACCTCGTgTTTTAAATtCAATTGAATCTAAACTAAAaGTACcTTTAAGtAaGgCTaCATATaCCGGCTGaCCTGTTAAACCATCTTCCGATtcGCCAaTTACTGTtaCATACGGTGCTCTTGTATtCTCTCCTACCCAAGaTGTACCATTTTTATCTTTAGTACGTCCAATAACTGTGTTTAAATCATCACTTGGAATATTGAAAATACTCATGTCAGACTTAACTTCATTAGTACCTtGTTTTTtCATCCATACACGTTTGTTAGATGCAAACATATCTACTAAATCTGGTGCTAAACCTGTGATATTTAGGTCAACTGTACCACCTTTTTCATCTTCCCATGTCATGCGTTTAACTACTTTTGTTGCTTCTGGGTTAAAAACTCCAACGTATAATCTTTTAAAACCTACTTTATAAGAACCTTGTCCTTCTGCCATTGCTTATTTCC

>NRS26contig00337 [organism=Staphylococcus aureus]

AATCACTGCCTTTTCTTGTGGTTCGAGTACGACAGTTTCAGCTGAGAATATGTCATAACCTGCATCCGTCTTATGATTTCGTTCGGgCATTCTAGCATTTTTTGATAATAGTTTtACTTGTAATGTGTTAGTCATTTTCCTATTCCTCCTCATATTTATAGACAACTTGACCTGCCATAATCCCTACTGCTTCATCAAGTTCAATACCTTCTTTAACTGAATGTTGAATAGCATTTGTCATTCCCTCAAGTATTTCATCAAACGCTTGCGCTTTCTTATACACGTCCTCAATCTCTTTTAGCAACCCCTCTGTGTCATTACCGTTATACGCACTAGCACTAATAACGGACTGTTCGATTTTTtCGCGATTATTCATTTGTGTCATCCTCCATAAAAaTTTTATTGTTtAATTCcATTCCgAaTTTAACTCTTTCATCATCGTTaCCGAATTtGTTtATTAAATCTTTTtCAACgCTCTTGCAATACCTATCCCATGCGCTtGCTTTCTtCTCCAGTtCTTtGTTACAATCTCGTAACTTCGCTATAACCCCAATAAGCTCATATCGTTGCTTcTTGTACTCATCACGTTGTTTtCTCATCTTCTTCAACCTAGCGTCCATTACGCTTAGTTGGAACCCtGTTTCATAGTTCATTCTACCAATCTCCCATCTTTCCAAATTAATGTCATAGTTAGGCCGTCGTTCAAGATGTAGAATGCTTTGGTAGGgAAAAaCGTGTTCTCTAAACGTTCGTTGATACTAATACTTGTGTGTAACGCTGACATATAGGCTCCCTCTTGAAGCTCGTACACTTCAAACAaCCTATCAAATACTGTATCTTCTGTGATTTCCTCTTCAACTTCAACTATGAAAGGaGTATCAATTGGAaTAAAaCTTGATATCGAACACGTATTTGTATTtCGTTGAAAaCGAACGAATCCATTACTAAAaCTTTtGCAaGAAAAATTTTtCCTTTTGATAGCTCCGGATTTTCTCGCGCCCACTTAATTAATTCATCTAGTCTCATTTCTTTTTAACTTTGATTTTCATTTTTACATCTCCTTAAAATAAAGTTAGTTGCTTCTGTTCCTCATATTCCAAACCATGTTGCTTTATATATATTTCGAGCTCTTCCGCTGTATCAAATGTCTTTTTCACGCCTTGCCAACCTGGTACGATATGCCCATGAAAGTAATAAGTGCCGTTTACTACATGGATATGCGCCACTCCTTCGTTATCCTGATACAGATATCTCTTAGATCCGAAAAATTGGTTTAAGTATTCTTTACATGCGCTATCGGTTTTAGGCATTTATGCTTCCTGCCATTTCTTAAACATTTGGTtATaaGTAGTATCAAACCAGTACGGATCACGTGAATGTTTtGAGgCACATTAAACAAATGTGGCTTCTTCTTACGTAGTTCAGCCTCTTTACGTCGTTGCCTAGCC

>NRS26contig00374 [organism=Staphylococcus aureus]

cgttagcgcatgagaagtgtttgaCGAGATtAaGTCACGTtGtAgTGGAACTGGTGCAAGAAtATtGGTAGATACCAACCCTGACCAtCCCGAGCATTGGTTGTTGAAAGaTTATATTGAAAATACAGATCCTAAAGCAGGTATACTGaGTCACCAATTTAAGCTCGATGACAATAACTTtCTTAATGATAGATATAAAGAGTCTATTAAGGCTTCAACACCATCAGGTATGTTCTATGAACGTAATATCAACGGTATGTGGGTGTCTGGTGACGGTGTAGTATATGCCGACTTTGATTTGAATGAGAATACGATTAAAGCAGATGAACTGGACGACATACCTATCAAAGAATACTTTGCTGGTGTCGACTGGGGTTACGAGCACTATGGATCTATTGTGTTAATAGGACGAGGTATAGATGGTAACTTTTATTTTATTGAGGAGCACGCACACCAATTTAAGTTTATTGATGATTGGGTGGTTATTGCAAAAGATATTGTAAGTAGATATGGCAATATTAATTTTTACTGCGATACTGCACGACCTGAATACATCACTGAATTTAGAAGACATAGATTACGTGCAATTAACGCTGATAAAAGTAAACTATCGGGTGTAGAGGAAGTTGCTAAGTtGTTCAAACAAAACAAGTTACTTGTTCTTTATGATAATATGGATAGGTTTAAGCAAGAGGTATTTAAATATGTTTGGCACCCTACAAACGGAGAGCCTATAAAAGAATTTGATGACGTGTTGGACTCGTTAAGATATGCCATATACACACATACTAAACCTGAACGATTAAGGAGGGGGAAATGACATTGTATAAGTTAATAGaTGATATTGAAGCACAaGGAATATtGCCTAAGCATATTGAGGCTcTAATaGAGTCACATAAaGACGaTaGaGAGAGAATGGtAATCTCTATAATAGATACAAGACACATATTGACTATGTACCAATATTCAAaCGTCGACCAATTGAaGAAAAaGAAGATTTTGAAaCtGGTGGAAaTGTAAGGCGATTAGACGTGTCTGTtAATAaCAAaCTTAACAACTCTTTtGACAGCGAAATTGTTGAtACACGTGTTGgTTATTtACAtGGTGTTCCTGTTACTTATGATTTAGATGAAAACGCAGAAAAaaCGAAAaGTTGAAAAAGTTTATAACCAACTTTGCCATTAGAAATAGTGTTGATGATGAGGATTCTGAAATAGGTAAAaTGGCAGCAATTTGCGGATATGGTGCTAGGTTAGCATATATTGATACGAATGGTGATATtAGGATTAAGAaTATAGATCCcTATAATGTTATTTTGTtGGCGACAATATTTtAGAACcTACATActCATTGCGCTACTTTTATGAAAAAGATGATGATAATGG

>NRS26contig00487 [organism=Staphylococcus aureus]

ttCGTCTATCTGTCTTTCCCTCTGGATAGCATCTAAAGTTttATCTAATTTAATGTTAACTTGCTCTTGAGTTTTTtGACCTAATTTAATCTCATTGAGAGTGCTAAGCATTGTTTTATCATTCTCTTCTAATCTTCTAATTCGCCATtCATGTTCGTGCCGTTtGgTAAaTCCAAACATTACGCCACCTACTTtGTGTTAAATTAAAAaGCCTCAAGCATTACACCTGTGACTTTTCATCTTTTGCCTCTGGATATTTTtCACCAGTGATCAATGCATATTCTTCTTTGTCGATTACACCCATGTCTACGTACCACTTAATTTGCTCATTTTtATAGcAACCCcACACATAAAAaGTTTTAatGTCTTtAAAAGTtGGaTAAATCATCTTCATCATTTAAAcGTCCCCcTCaGTATTTGTTTtGTTAGTTTTCAGTTCGGTCAACTGTTGTGTTAACATAGCGTTTTGTTGCGTCAATTGCATTGTCAACATGTTCACTTGCGTCATCTgCATTTGCATACTCGCAACCATTCCGCGAAGTTCCTCATCACTTAAATCTGACGCACTTTGTTGGTTTGATGCATTCGGTACGTCTTCTTTTTCGAAATTGCTATTGTATTTAATTTCGCCGTTAGTGAAAACAAACTTTCTAGGTTCGAACTCTTCTTTAAATTTAATAGGCACATTGTTATCATCTACATCTAAACTATTGCGTAAACCGCCAGTATTAACGAATCCGATAACTTCGTTTTTATCGTTTACTGTGATTTTCATTATTTCCACCCCATAATTTTAGTTATAGTAACTTTGTTGGCATTCGCTCCAGAACCTGATGTTTTaCCTAAATCAAAGTACACATCGTTATCTATTCTTAAAGTAGTGCTACTTGTTTTGGATAGTAAGCACTCATAAaTACCGCCACCGTTGCCGTCTGAGTCAACTACATTCGCTTTACTCAATTGAATCGCGTTAGGTAATGCGGTTAGTCCGAATCCCTCAATAACGCCACCTGGATAAGTTCCACTTACCAACAAAaTAGAATAGTTTGTGTACGGTTCGGTTAGATTGATTGTTGTACCTACACCATTTGCGCCACCGTCGAACAATACCGTTGACTTATGTTCATTAGGAACCGTCCACTGTGGCTCAAGTCTGCCGTTTGTGATTGATCGTGTGTAAAtCTTTTTAGAGttataaggtgtgagttaacaacttatttgtatCATCtttaacgaacaca

>NRS26contig00526 [organism=Staphylococcus aureus]

CTGAAAACAAGTATAGAAAAGCAACAGGGCAAGCGCCAATTAAAGAAGTAATGACACCTACGAATATGAACGACACAAATGATTTAGGGTAGGTGTTGACCAATGTTGATAACAAAAAACCAaGCAGAAAAATGGTTTGATAATTCATTAGGGAAGCAGTTCAAtCCTGATTTGTTTTATGGATTTCAGTGTTACGaTTACGCAAATAtGTTTTTTATGATAGCAACaGGCGAAAGGTTACAAGGTTTATACGCTtATAATATTCCATTTGATAATAAAGCAaGGATTGAAAAaTATGGTCAAATAATTAAAaCTATGATAGCTTTTtACCGCAAAaGTTGGACATTGTCGTTTtCCCGTCAAAGTATGGTGGCGGAGCTGGACATGTTGAAATTGTTGAGAGCGCTAATCTAAACACTTTCACATCGTTTGGCCAAAATTGGAATGGTAAAGGTTGGACAAATGGCGTTGCGCAACCTGGTTGGGGTCCCGAAACCGTTACAAGACATGTTCATTATTACGATGACCCAATGtATTTTATTAGATTAAATTtCCCAGATAAAGTAAGTGTtGGaGATAAAGCTAAAaGCGTtATTAaGCAAGCAACtGCCAAAAagCAAGCAGTAATTAAaCCtAAAAAATtATGCTTGTAGCCGGTCATGGTTATAACGATCCTGGAGCAGTCGGAAACGGAaCAAATGAACGCGATTTTATCCGTAAATATATAACACCAAATATCGCTAAGTATTTAAGACATGCAGGTCACGAAGTTGCATTATATGGTGGCTCAAGTCAATCACAAGATATGTATCAAGATACTGCTTACGGTGTTAATGTAGGAAATAATAAAGATTATGGCTTATATTGGGTTAAATCACAGGGGTATGACATTGTTCTAGAGATTCATTTAGACGCAGCAGGAGAAAGTGCAAGTGGTGGgCATGTTATTATTTCAAGTCAATTCAATGCAGATACTATTGATAAAAGTaTACAAGaTGTTaTAAAAAATaaCTTAGGACAAATAAGAGgTGTAACACCTCGTAATGATTTACTGAACGTTAATGtATCAGCAGAAATAAaTATCAATTAtCGTTTATCTGAATtAGGTTTTATTACTAatAAAAAaGATATGGATtGGATTAAGAAGAaTTAtGACTtGTATtCtAAaTtAAtAGCTGGTGCgATTCATGGTAAG

>NRS26contig00595 [organism=Staphylococcus aureus]

CGACCTGCTAAAGCTCCCAAATCCTTTATTAAATACAAATTTTCCATAATGCACCTTCCTTTCTAATAAAATAGCACTGTACCAAGTTTCCCACTATCGTCAACTGTTATTTTCCACAATTTACCGTTTGGGGATTTCTGTACAATGCTATTTTGAATAATTCCTGCTTCGCCTATTTTtAaTTtATCTAATTtATTTTTAtCATCTACCGAAATGATACCGTCTTGAGGCAATCCATCAATAtCACTACTGCCTGCATAAGGTATCCCATTTATAGCTTTCCAGTGTGTAGCTGGAAAGTACTGTTTATCGTTTTCAAGTAGCGCTTTGATTTTAACTTCTtCTGTTGCCATTATATTAATACACTCCCTATATCCATTGTCTCGAAAGGAGAATTCAAAGTACTAGTGTATAAATGATTTATACGATTTGCTTGATAGTTATATCTATTATCTTGTGCAATAACTCGTCTGTTAAGTGCTTGTTGAATTTGTACCATATCTTTtATTTCATTGCTGAAAGACACTTCATCTATTGCGTTtACAAATGGATGTGACCTATCAAGTTTAACAACCTTtAaTTCAGTGTTATATCCcATTAATTCATGAACAaaaaTACGCTATCTCTTGGCTCTattttttCATAACCTATATAATTAACATCTAATTCAGTCTTAGGAGTATCATTtATTTGCTTTTtGCAaaTTCTAACAGCTTATCCTGTGTTTCGATATCTTCATTTGTTTGCGTATTAGCATATCGAATCCCAAACTGCTTTGCACTATCTGCGACGTAGTCGACAATTGCTTTGTATTGATTGCGACCTGAATTATCAGCAATTAAATTTAAGACTGTTGATTTTTCAGTTCCAACATACATACAAGGCTTAGCTTTTTtATTTGAAGATATATCAATTCTATTTTTGGGGTCTTCTCCTAAAAATATCATTTCTAAAACGTGCTTGCCTTTATCAATATTTTTATtAAATCTATtGTTtCAGACTGAACCGACTTAGCAAAaCAaGAAATTtGCTTAATTtGCTTGCCGTCTAAAATCAACTTATATATTCCACCTTGAGAGCCCTTTTTtATtGTAAATCTAACtGTTTCATTACCAtaCTtGCAat

>NRS26contig00630 [organism=Staphylococcus aureus]

GTCTgTAgATATAGTaTTTtGCTTACCATTGACCCAGAGaTTAATAGGTATATTTCTAGgTTTATCTAAATTTTGATTCGCCAaCGTAATTCCACCATACATATATTTTGTATTACAAaGACCTGAGTAACTTATTCCAAAGATATCTACTTTATGATCTTTTAATCTCTTCGCTTCATATTCATTATGAAACTGTGAATATAAAGTGTATTTTCCATCAATATACTCTAATTGATGAGAAGTAGAAAAATTTCCAGAACTAACTCCTTGAAGCTTTTCAGGTTCATAATTAGCATAAAAGTTTCTAAGGTTGATTACCCCTACATCAGCATATGCTAGATTGTTTTTtATTAAAAATGTGAAGAAAAACAAGCTAACAGTGAGTATTCTAAATATTTTATTCATTTTGATGGCTCCTTTTTTTtAtATTACAACGCCATATAGGCATTTTTAATCACAATACAACTTTTCCCATCACTTTAATGTTTTAtCCATAGAATTCTTGTATTTATTGAAAGTAGCATCATCGATATCACCGTTCATTTGTATTAAAaaCTtACCTttAGAAtGGGTATGTGAATAGAAAGCTGCACTTTCTTTACCTAATTCATCGTAATATTTTtAGTTTGTTTTAGATCATCACTATTTTtAAATTTCATTACTCTAGCATTTTTATCATCTTGAACAACAAACaTTTTAGCTTCGTCcGTCTTCATTGGAgCAAGCCCAAaGTCTTCACGTGTCATTTCTTTTtCATTGATAaCaTTTAATTTATTATCTTTAAAACCTTTAACGATATCATTTGTAGTATAGCTCTTGCTTTCTtCTTTCTTATCTCCGTCGTTGCTACCACAAGCGCCTAAAATTAACGTACTTGCTAATGCTAAACCTAATAATTTTCTCATTTTtCATTTCTCCTATTTATATTTCTTTATATTTAAAAaCTCTCAACGGCTCAAATGTAATAGAATACTCGCCATAGTGAGTTCCAATACCATATATCTTTTtATATTGTTCTATTGCTTCTAATATGTATTCTTCGCTTAATTGTAGATACTCAGACAATTCATACAAGTTACGTACGCCATAATTG

>NRS26contig00645 [organism=Staphylococcus aureus]

TATGAACATTCACACTATTTCGTAACTTTGCGAACTTTTtGCGAACATACTACCCTTGCCCCTAGTTTCACAATTACACCTTTTTtCGCCTTTATAACAACCACACTCCTAGATCAATAGGTTGTAAGGTTTGAATGTACCTATAGCACTGAACATTTTAATGGgCTATACTTCTCAACAATTCACATCACTTCATAATTAACGTATTGCAATATGTATTCACcGCCTGCAAAGTATGAGGTCATATTTTTATATCAaTGTACAAAAAACTGTTTTGCCCTTAAAACAACCACATTCCTGATTAATAGGTAGTTTAGTTTGAGCATTTTATAATTAACATAAAAAATAGACAAGTACCGAAGTACCTGCCTTATTTCTAAATCCACaGGAATTCATTAATATAATAaTTATAATTGTAACTTAATTAATGTCTATTAtAGTTATATATAaTAGAAGCAAAACCTAAAaTGaCTATAAAAGCtATCAAAaCATAAaCtGAAAAaTTATCAGTTCTAATATAAAaCTTAAAaGTAaGAACATTACAAAaGCAACTATAAAATAGACAGgAATAAACGACTTTCGCCAAAGTATAGAGTTTTTCATGAATTCACTCTCCTATCAACCGAAACATGACGCTGCGGCACCTGTGGCACCACCAGAAACCCCACCAATAATAGCACCTGCAACAGTTCCTAAACCTGGTATAACTGAAGCTGCACTAGCACCGCCTAGCCCACCAGTGCCAGCGCCTCCAACTGTTCCTAGACCACATTTAACCCAGTTAGTTTTTCTCTCTTGACTAGATTTAATTACTTGAACATCAAATCCATCTTTAACCTTTTTATAAACCAAAaCAACATCGTTACCTTTTTTATCTTTAGCATTAGTTGGCATCACTTTTGACGTTTTTCCATCAGATAaTGTTAACTTACCATTTTTGTCAACaGtCCCTTTAAtACCcTTTtCAAATGAAAGATGCAAGGTACTTTCATTTTTTtGATTCAGTTTCCCGATATtATCaCTTTTTtGTtCAGCATGAGCGTtGTCATCcATtACAAGAGAAGATCcTGCTATT

>NRS26contig00701 [organism=Staphylococcus aureus]

AATTTATTGTCGCCATTACGATAATATAAACAATtCTtCGTTTTAAGCAGTTtCATACGTTCACTCCTATAAAGAGAGCCTACCCAAATTGGATAGGCTATTTTtGATTTAAGCGTTACGGAaCACTTCGTTATACTTACTTtGAATGTTAATAATTtCTATATCGCCATCACTATGTTTGATGACTGGTTGCCCGTTATTTtGTAACCCAAACTGTCTTAAAACaTTATAGTTATACTCTAATTTTTGATATTCTTCATTATTTCGATATGGATAAATTACCTTTTCTACCAATACATCAAAgtAAGGTTTTAACCTtACATTTTCATCTTCAGTAAGACGACTTTCTATCGCTTTTTtATAGATATTAAGTTCATATACATTAGTGGTTTTAGGATTGGCATTATAAACAAGaTTAAATAGTTCTTCTGCATCAATTAAATTTACTTTCGCCTCTATGTCTTGACGTTTCAACATTTCAACTTGTGGATTCTCATATGAAGATTCTTTCTCTTTTTGTTGGATTTCTACTATTTTTtCTTCATGTTCATCTAATAATATTTGTCCTAATTCTTTGAATTTaGATTGTAGGCTCAAAGCCTTATTATCCATTTtATTTTtAATAACATCCGTTTTATAGCCTTGTCTAATTAATGATTTCGTTTCTGTTATTAGATCTTCAAAATCTCCTAACAAATTTCTATAACGTCTATCATTAAAATATACATCCCACGTATCACCCGTGATTGTTGTAGTTGTCATTTAtAaGtaCctCTTTCTTTAGTTTttGTTttACaCTtcAATtCGTTtCAAAGCTTCATAGCGTTTCATACTGCCATCAGCTAATTtCTTAaTACTTCTCATCGCTTGTTGCTTTtCTtGTTCTGTCGTAATGATGTAATAACCACGTTCACTAGGTTTATAACTGCAtCcGATAGGATAaCCaTAATCATATACTAATGAATTGATTACTCTTCGTAACCATCGTTCATTGCTTGAATTATATTCATATCCCAATTGA

>NRS26contig00716 [organism=Staphylococcus aureus]

GGACTTTAAAGTATTCCCAATTATAATTCTTCATGATTTTCTTATTGGATTTcGAATTTGGTTTCATGCATTGatGtcTCAAAGAACATGaTGAACAGTCATCaCATTCATATAGTTTGAAGTCTCGTTTAAAACCATATCTATCATTACGGTATGCATATCTTTTAAAACCTATTCTTTTGTTATTAGGACATATAAATTCATcATTAAGTTCGTCATATTTCCAATTTTGAGTGTTGAAAATGCcACTTTTAAACTTTCTAGTTTTATCTTTAATAAACATGCCATACGTAATAAGTGGCGTTTTATTAAAATCATCTATAATaGCCATATAGTTTTGCTCACTACCATAACCTGCATCAGCTACAATATACTccGGTAAATAACCGAAGGTATTTTGAATCATTGTTAAAAATGGgaTTAAatGTTCTAGTATCTGTTGGGTTTTGAAATAGGTCATAGGATAAAACAAATTGAGAATTTGTCGCTATTTGTAAATTGTATCCTGGCTTAAGTTGGCCATTTTTCATATGGTCTTCCTTCATTCTCATAAAAGTTGCATCATGAtcagaTCAGTTTTAGAAAAaCTaTTTCTATCTTTAAGAATCGATTTTtGTtCTTCATATTTATTTTtcTTtCGGAATAATCATCAAATTtCTTTTtGAACTTCTTAATCTCAGTTcTTTTttACGGGTCTGTTTTCTAATTTGAGCACAATCTTCGTtCTCAATAGAATGATTTAAATCTTCGATTtCTTTATCTAAATGACTACCAATTAAATCTATTTCTTCTATTGTTAAATCGCTATCTCCATCTTCTTTTATCTCTGGTATTATTTTTtCTTCAACTAAGTCACGATATAaTGTTTTtGAATTtTCGTTCAATTTCGATTCGTGATTTTGAATACTTTCTTCCgCACcAaTGTATATCTATTGGCActAGCTTCTACTTTTGTACCATCAATAAAAATTGAATTAtcATCAATAAGATTTTGCTTTAAACATTGACTATa

>NRS26contig00796 [organism=Staphylococcus aureus]

TAAAAACATTtCTTTTAAATCCTTCAATGCAGGGCTCAAGGTCAAAGcTCCTtGTCTtGTTtCTtCcGTTtCAAaCCCGTAaTTTTTAaCTCTtGATTtAGTTtGAATGCGTTCGCTCTATCATAAGTAATTTTTtCTACTACATAATGCTCATTCATCTTAATTATCCAaTTTAAAACATCTTGGTAGTCAATATAAGGCTTATCTTGCACTGTTAATAAGCCATCTTCTTCCCATTCTCTATAGGGtATTTTTtCGTTAGAATATTCAACTTTGTGCTTAGGAATCCATGAATGCGATAAAACTGCAACTTTACCATTATCTAACGCAAAAGTAGCACACGCGGCTGTAAAGTCCTCTGTTTCTGATAAATCATAACCAATCGTGCACGGTCTGCCTTCCAGCTCTTCTAAAGAAACAATTTCATtATTTTttGGAGTGTTGGGTAATCAATAAAACTCATCTCGTCATTATTAGCAAAGATaTTAAACCTTTTGGTTATAAAATCTCCACGTTCAGCTGGTGTTCTCTTAGCTTTTTCCCACTCTTCTTTCATCTCATCTAAATTTATAGAGACACCTAAGTTGGGATTTGCTTTTATCCAGTTCGACGAATCATTAATATCATCGTCATCATCCAAAGATGCTAAATAATAAAAAGTTCTTTCGtCTTCTATGATTTGATCTAAGGTGTCTCTTCCCGCTTCTACCaTaTCAACAaGTGgACCATCtAATtGATaCCCTGCTGTCGTAATgTAGATGAGAAgAGGTTgTAACCTTGCaGcTcTTGaGTTTTTTAAACTGAAaTCAATTTatAGTCTTtAAATtCATGAATTTCATCAAAAAtCCCCATgtgTGtATtCAaTCCATCTAAcTtATCgctatctgatgcttggggcataattttgatatcgttgcgtcataatggtattcatctcttaatgttactgtaaa

>NRS26contig00805 [organism=Staphylococcus aureus]

ATaaattttggtatccacttttttaataCaaaaaCCACCTAGCCGATGGTTATACCGACTAAGTGGCATGTGCAGTGTATTAGgAAtAAAAaGTAAAATTACTCGCcTTTGTTACCTTTtACTTTATCAATAAAaTCAGTTGCTTTTtCTTTtGCATTTTCAACGAATTCTTTCGCTTTACCAGAAGCTTTATCTTCTTTACCTTCGTTTTCTAAATTTTtATTATCAGTAACATTACCTACTGTTTCTTTAACATTACCTTTTGCTTGTTCAAATTTACTTTCGTCTGCCATAATAAATGCCTCCTCGGAATAATTAAATGTTATATATAATACTTACCCACTGAAAAaTTATCTAAACATTTtACTTAAATAATTTTtGATATTGATTTGACGTCATTTtaTAaCTAGCGAAATAGATTCATCATTAACTTGAGGGAGTGGGACTGAAATAaTAAAGAATCACTAATGATTTATGATGTATtAGTCACTAGCCATGTGAAATTAAAAATAaGAATAAatGAGTAGCACGCATGCATaTAGgaTTTTACTTtATCCGTAATAGCATCTCATTCCTAAATATCATATAAATACCtGTTTAAaTTAAAAaGCCCAGCAACATCACGTtACtGAGCCATTAATATGATTTATTTAGCAGGAATAATtAGCCAGATTATCAAGTAAGTTCAAGGGCAATCAAAaCATTGTTATATATGTATTTATAAAATTTTCAAGATAATTTATTATTCATACCCTTGCCCTTTGTTTTAAAATTATGCCCTTTTTTGCCCTTGAAAACAACCACACTCCTAAATTAATAGGTGGTGTGGTTATTTTGGTTGTGTGAGAATAAAAAATAACCGCCTCGAATTCGATACGGTTATCTAGCAAGAACCACGTACTTACAAATACGTTTAGAATCTCTTCGACAATCTTGCTATAGACAGTCT

>NRS26contig00876 [organism=Staphylococcus aureus]

GTGCAAatCCATTCTTCAGGTGGCATCTGTTtaTTAAAatGTTtAGCAaGACAAGTtCTTtCGAAaTTAGCATtGAATGCatACTTTTttACAGCAGGgTCAaatAGAGCAaTTTtAAACGTCTCatAATCAGCGTGGAAAGGCTCATTATCTACTTTAGTCATGTCAATCGCACTAATCGCTCCACCATCTATTGAATAAGCTATAATTAagATTTCGAAATCTtCAGCTTCTGTGTATTTATAGGCACCACATTTCGAAATATCGTTAcTGCTATatGTTTctAataTCTAtATTCATAAATTTCAAaTtCTtGacACCTCAATTTCTTTAAAaTTAAAGTGGGGCTAAAAACCCCaCcTATTGACTtATAaGAAATCCTCATCATCAGTGTCTAATTCATCGAAATCATCTTCTGCTGCACTTGCACCGCCAAGAGGTTCGCCTTTTTCTACAAGTTGAATATtGTTCAATCCAACTGCAATGCCCTTATTACCATTTGTGTTAAAAGGAAaTAGATTAATTGAAGCTCTAATATAATCACCACTTACAACAGTTCCaGAATCCGTTAATCTAATTTtGTTTTGGTCAATAATACCAGGTGCTTGTTTGCTTGATGCGTTAATAAAATAAGCGTCTTGATAATTTACATCATCTTCTCTTTCAGTATCTCCATCACGTAATGGGAGTTTCAGATTTGCAGGAACTTTGCCTCCAAATTTACTAACTTTTCCTTCTTCTTTAGCAGCTTCTATAGCTTGTTCAATGGCTTTTATCGTACTTGTATCTGATTTAGGAATGATTAAACTGATTGAATACTTTGCTTCTTGCCCTTCTTGCATACTGTGAGGTTCAAAAAaTaTGTGCATATGATGCTCTTACTTTtCcTGTAaTCA

>NRS26contig00948 [organism=Staphylococcus aureus]

ATTCGTTTCATTTAATATAAACATTGATATTATCACGTCTATATATATTAAATCAATAAACCTATCTTTTCATCTAATTCGCTATAATCTATTGTACTAATATAGTTTTCATGAAGAAGATTTTTATtATGTTCATTTATTtCTTTTTTtGaGTCATTtAAAATAAAAaTAAaCTCATTTTGCCTGTGTCTTTTtATTTTTTtGCTTGCATAGCATCCGTTATTTTCGACTTAATAATCATAGAATtATTAGGGTTAGAAATAGCATTGATAAATTTTtCTTTTtGATTTTTCTtAGCGCTAaTAAAAAaGTCGAAATTATGAACAACACCACTGCTTCCTATAATAGGTAAATCTCTtCCGTAATAAATGTTATGTTCATCCAATTTGTTTGCAACaTCTTCTGTAAATaTGTTCTGTGACTTAGGATTAGAAAGTAaGtACATATCATTAacAAATATAAGGCaCTGTAATAAaTTATgTTtcGATTtATTAAAaTTTTtAAAgTtaGTTTGAaCAAAAATTtcGTGAGTTttAtCGTtAtaTTTAAtACCGTAaGCTGAAaGGtGCTCTTCAAAAATCTTTTtACGTTTTTTTgATTTATTTAAAGAAATaCCATTaTTTTCTAAATCAAATATaGTaTAGCCGTCATCTGTCAGTGTAATCATGTCTCTGGACTGATCGTATAAAGCGTAAATTATTAAATTATCTAAAGAATTATCTTTAAAAGGAGTATCTATTCTtACTAcATTGTTTGATAAATTAGAAAACTCTAAGgTCTGATtATACCAATCAAGAtATTCTTTTTTtAATTTTTtAGCAtCAAATGTAGtCATATTAT

>NRS26contig00998 [organism=Staphylococcus aureus]

AAGGTTCTGCGCCCATGATTtCCTTGTAATTCTCTTTCATCATTTCTATGCCTGCTTTCGTCATTTCTTTGTCcTCGCTTTTGGCCATATTCGGTGCTTTGTTCAATGTCATCCAGTACGAGCGACTCTCCCTCTTGAAAAAaCCACTATTGTTAAGTTTGTCCAAAGCCCCTTGTAaTAACGGCAAAGTATCCTCGTTTTCAGTGATGAAATCATCAATTGCTTTTTCTAATTGTTCTCGAGTTGGTGGGTTTTTtAAATAAGCAGTAGCACATTCCCAAAaTTGTAAAATCGCTTTGTTTCTAGATTCTAGCAAACCGTTAAaGATAACATTGAATCCTGGCATTGCTCCTTTTCTCCCATCTTCGCTATCTTCTGAGAATTTTTCAGCTTTTCGGTCAAATGCAAATGTTACTTTTGCTTCTACTTCGTAaTCTTTTTCTCCGTCATtAATTTTtAATGTtGTAaTtGGATTAAATTCAGTCAAAATATATACCTCTTTtCAATTTTtttATAAAAaaTAGGgAGCTTACGCCCCcTTGATCTATTAGTTTACATAGAATGGTCTTCCGTGTGTGAATCAGATACAACACTAGCTTTCTTTtGATTCTCGAATGTTCCGACTTTTtCGCCGAATTTTtCGTATTCAACTGTAGGCGCACCTGCAGCTTCAAACCACTCTTTCGGCAAGTTATCTTCAGCACCTTCTGCTGTATTCCATTTAACTTTtAATGATAGTTCGATTTTGtCACTTTCATCATCAAATGACATTTCAAATGATTCTGGAaCAaCATAaCCAAACATTCCGTATACGTGGCGT

>NRS26contig01062 [organism=Staphylococcus aureus]

ATCAAATGTCTTTTtAACGCTTTGCCAaCCTGgCACGATATGCCCATGAAAGTAaTAAGTGCCGTttACTACATGAATATGTGCCACTCGCTCGTTATCCTGATACAGATATCTCTTAGAGCTGAAAAaTTGGTTTAAGTATTCTTTGCGTGCGCTATCGTTCATGGTCATCACTCCTTTTAACAATTAGGTAGACCAAACGaCATGCATTCATCATATAGCTCTTCGTTCCTTATGCTTGTCTTAtAGTTTTCAATCACATTGCTAACTtCTTtATGACTCATTGCTTTAACTTGTTCGTCTGTAtATTTTTCGCAGTCTTCTAATTCCAGTTGCTCCTGTAATGACATCACATATTCAACTTGTTTTTGAGTTGCCATCGTTACCCcTCCCACAAGTCAAAAGCTCTTTGGACGTAAAaCTTCGCCTTTGCTAAATCCTCATGACCATTCTTTAACGGTGCTCTAGACAAGTATTTGATTGCATTACCTATTGCGAATGCTAATTGTGGTGGATACTGTGCCGTAAcTTGTTCGATGAAATCTATAATTTCAATGTCGCCGTATGTGTAATGCGCAGGTtGTTTAACATTGTCTTGTGTTTCATTCATATCTACTTTTCtGTTACTGATTATGCTCATtATGCTTCACTCCATTtCTTGAACATTtGGTTATAAGTGACATCGAACCAGTACGGATCACGTGAATGTTTtGAGGTACATTAAACAAATGTGGCTtCTTtCTtCTTAGCTCAGCTtCTTTCTTtCGCTGTCTTTCC

>NRS26contig01113 [organism=Staphylococcus aureus]

ACGTATTTTGAGCTACAACTGTTCCTTCTtCTTCCGATTCTAAAATATAGTTTTTTtCTAATTCTTCATATACTTTTCTttCTTTTTCTGATAAGACTACTGTTTGTTTAGTATCAACTCTGTCAGGCATATCCAGATAATCTTTCGCTTTCATGCTTAAACATATATCTTCTATTTGTTTATATATCTTTTCTTCAGATCCGTCTCTTAGCTCCCACTTAAAAaTATGTTCGCTAACTTGATGAGTTGGTTTAAAGTACCTTTCTCGATAACGACTGAATGAAGACTCAAGTCTTTCACCTCTGTCTATCAAATAAACTTGAGCCCATAAATTCTGTAAATTATTTGGACTAGGTGTTCCTGTTAATCCTATAAATCTATTAATGagTGGTAATTTCTTTTtAATAGATTTAAACCTTTGACTCTTAGGACTTTTAAATGTAGACAGTTCATCAATCACAACCATGTCAAATGGCCATTCTTTTTtATATTGATCGCATAACCATTTAGTATTTTCTTTATTGGTTACATAGATATCAGCCTCTGTGTTTAATGCATCATTTCTTTCTTTAGGTGTTCCTAAGACTAAAGACACTTTCAGATGATTTAAATGGTTCCACTTATCAACTTCATCAACCcATGTATCTTtAGCAACTTGTttGGgTGCtATGACTAACATTTTTTTtAGtGTCTAaCAaCTGCAATTCaCTAAatGCTGtaagtgttgatactgttttccctagccccatatctaa

>NRS26contig01140 [organism=Staphylococcus aureus]

CTACGtttAtGCACGTGGCACAGgTAAaTGgATAAaTaTGATTAACATtGCTGATTATACTAATtcaaaatataatattgcaccagatgaacttaaaacacttccacctgttttcattgcgcattgtaatggcgattatgatgttcccgttgaggaaaGCGAACATATCATGAATCATGTTCCACATTCAACATTTGAACGCGTGAATAAAAACGAGCATGATTTTGATCGTAGACCTAATGACGAAGCAATCACTATTTATCGTAAAGTTGTTGATTTCTTAAACGCTATAACAATGATGTAATATATCATTATTTTAAAAAaCTCAATTTATCATCATGATAAACAGTTATTCGATCAACCATATTATTCATACAATTTATTAAGCTCATAAACAGATACGCCTGTCCCTTTAGTTTtCGCTAATGAGACAGGCGCTTTATTTCACTTTACTATTTTtAAATTCAACACTGTGATGCTACTtATATCTGATGTGCCGTGATAACAGCAcGATTTACATAGCATATTAAATTCAGAAGATGAATTTAACTTGCTTTCAATTGTGTTCCTTTACTTTCAGTCGCTTCAACACGCAACAAGTCATTTATAATACCTGTATAGAAGTTGGCAAGTTCTGCACCTTTACGTTtGAAATCCAGGAaGTCCACACCGATAAAaTCGACATGATCCCATCCTTGTATGAttGGtaaaccggtcattctctaacgtacacgagcgt

>NRS26contig01156 [organism=Staphylococcus aureus]

TCATTTGCGTCACCTTCATCTATGTCAGGTAATTTGTCATTAAATTCAAGACTTTCTTTTTCCATTTCGTCTAATTCGTAATCAACATCATCAACTAGTTGTGATTGTCCTAACCTTGTTCGTTCTGAAaCTTGTCCCTTcAGGTTAATTAGCACTTGTGATTCTTCTAACTtaTTAACTGGAATGTTACGAGTGAACTTAAATATCAGGTTTAAaTAaCTATCATCATCCAAGTTGTACCCTTTACGCTTTAATGCAGaTAAAATAACTTTGAATTGATACCTCAACATAGCTGTCATCTTACGCTCAAACGTCATACACTTGTTCTCTAAAGCCATAAGTTTAAGTTTCATTCCAATGATAGGTACATTTCCGTTAAACTCGTCAGAATTAAAGTTTACTGACTTTGCAAAACGCATGATATTCTTTTCGATTCGATCTAAATGGTTCTCAATCATTGTGTCATTTACATCTTTTGTTAAGTATTTAACGTCCATatCTTTGTCGAACAACTCAAATGCGCCACTCTTTtGtGTTTCTTGAATCATTtCTTCACTCATACCCATACCGcGTAACaCAAGGTATGCTAAACGTGTCTGACtAATCtCACTTGATGCATCGCTCATTGTTAAATCATATGCGTCAATTAAGTGAATAACCTTTtCAGCATCTCCTATCATCTCTTTGTTGTTAGGTaCACCAAACAATGGATTGTAATCaaaTAAAtgTTCA

>NRS26contig01194 [organism=Staphylococcus aureus]

TTttAATTtGCATAAAaTAGGCAAGTACCGAAGTACCTGCCTAAATAACAACAAGATTAACATGTGAATAATGGAAaTAAaaGTCAGCCcGAAGgCTAACTTaCGAATAGATGAAAaTTTGAACACATTGCTGTGTCTAAAaCGATTATAGCATAGATGACGAATATTTCTAGCTCAAAaTTATTATATTTTAATGATAAATTTttATATATTTGTTAATAATTATTTAATTGATTCACATAAATAATTATTGTAAAATTACTTTGTAATCGATTGCAAATAAGTTATAGGAGAAAATAAAaTGAATAAAAAaCTATTAACAAGAACATTGATAGCAAGTGCTTTAGTTTtAACAACAGTAGGTTCAGGTTTTCATTCTTCTTCAAATTATAATGGTATTAATAACGTTGCAAAGGCTTCTGAAATAaCaGATAGAGACTTATGGAAAAAtGTAAGAGATGCTTTGAAaGAAGCAAATATTATCGATAAAaCAGCAAATGAGACAGTTGGTGTTACGTACAATTTAAaTAATGGTGGCGAAAGTAGTATTACGGgTACAGCTGATTTAGATGAACTTAGTAATTTTAATAACAAGCCGATTAATACCGATAGTGTtAAAAGAATTGATTTGTCAAGAATAAATCCAAATGGAAaTAGGTTTGACGCAAATGATGCATGGAAAAaTtAACTGACAAATTAAtACGTGGCGT

>NRS26contig01196 [organism=Staphylococcus aureus]

TGTAATTACATTACCAGTAACCAATCTGGCTTAAAACCACATTTCCGGTAGCCAATCCGGCTATGCAGAGGACTTACTTGCGTAAAGCAGTAAGAAGCTGACTGCATATTTAAACCACctATACTAGTTACTGGGTGGTtGTTTTTtATTTtAATGATTGACATATTAATAGTGGTTGGATTACTATTTAAATATAAACGACAACGCCCCcACCCcTTTTTAGGCAGACAAGTTCTGACGTGGGGgTATTTTTtGTGTTCGTTTATATGTTCGGTCTACCTCTTTTTGTGTAAGTTGTGTATTATATATGTAATTGTGCTAATTAATCGGAGGATAGATATGGTGAAAATATTAACAGAGATTACGAGTAGAGTCGGTAATGGTGTAACAACACCGTATTATGCAATGATAGACTCTTTGGCTGTAGTaGTTAAATCGATTAACAaTAACGAaGGGTTTTaTGCTTTATtCAATGAAGCAGTAGGTTATTTTATTGCAGAGAGGTTAGACTTTTCACATCCTGATTTTGGATTTGCACAGTATAAATCTGATTTAACGATAAATCGCATACCAAATGATTCGAGTTTCAATGACAAGGAAATATTTACATATACTGTTTTAGAAAaTTCAGTtAtACACAtAGAAGGACCAGGTATGATTAATACAATTGATAATAAAGAtATtATtGAATtGATAaTATTTGATTCATTC

>NRS26contig01214 [organism=Staphylococcus aureus]

GTtgACGAACGAgAaCGTAAATGGTAAATTGTAATTAGTTCtCAAACGAGAACGATAGgaGGTGTAAACGTGGTACtAAaTTtAAAAAGATTGAGAGCGGAAAGAATAGCTtGTGGTaTTACGCAAGATGAAATGGCTCACAAAATGGGGTGGAAAACAAGAACGCCTTAtGCAAAGAGAGAAAATGGAATAGTAGATATTGGAGCGAATGAATttATtAAAaTGGCAAAaaTATtAGGTtATGAAACAAaTAaCCTAGATATTTtttACcAatAAcGTTCCcAgAAAAGAACGTAAAACATCTTAAAAGGAGgTGAATTAAATGTCTAGAaCAAAATTGCATGATGTACCAGCTAAAGAAAATACAaTTACAGAACCAAagCAAGTTGTAGTgAaTCCTTtGTTTGCGAAACCTAATgCACTAGCTagTATTTTtGGAATTTCATAtAGTtCGGTgAATCGcATTTTAAAAGAaTGGGAAAAAGATtCTAAAGGTgTTgaTGATTTATATTACtCgTtATCATCAACAATGATTGTTATAaGTATTCCaCGATTCGAGgAGTACATGAAGGCaCGTCATAAAAAaTGGATGTAGGAGgCAAGGCAATGAAAATGTATTtagCTTATATCTGCTTAGTTTCATTGTTAACAATTTTATTACTAGCAATATCTAACATGTATGTcGCTTTTAG

>NRS26contig01230 [organism=Staphylococcus aureus]

aTTTcACGctCTTTGCTCTCTCGCTCCATGATTTtGGATAACACAATTtCTTTATACTCAGCTAAGCGCATACCATAAGGTGCATGTAAGGCTTCTAACAACGCCCAGCCACCTCGTACTCTTTTtGCAACCATTCCTGGAGTTAAACCgTTCTTTTTtATCAATTCATTTTCATGTTCGGTAAATTTATATGGTTTACCGTTAATCTTTACGATACTCATTTATTCCACCTCTATACATTTACTTTTTtAaTCCAATCCTCTAATTTgTGCGTGTTGTGATTTCTAGTAAATAGTTCACTTACATTAACACCTAGAGCATCTGCCAATTTATCTAATACATTTAAGTTAACCATCTCAGCTTTtCCGTTTTtATATCCACTAATAGTTGATCTTGATACGCCAGTTTCATTGTGCAAATCTTGGACACTTACGTTATCTCTAGCCATGATTACCCTTAAATTAGTTGCGAATACTTCGTTCAACTTCATTTATTCCACCTCTATATATGCATGTCTTATTGTTATGTTGTCATACTTTAGTAATTCGTCCGGATTGTCATCTAAGCGCTTTGCCAGCGTATCTTTTTCTTTATCCACATCATCGTAATGCTGATATTCAACTTCTGTAGGTATTCTTATATCAATCGTTGCgTTTATATATGCTTGTTGTTGCATTAGATCACTTC

>NRS26contig01322 [organism=Staphylococcus aureus]

TTTTCTGATTATTTGAAGATTCTACATCTATTTGAATTAAGTATTCTTCACTGAGATATTTAtCAGACATAAAGTCTGAAGGCAAaTCATAAACaGGTGTAATAACAaCAAaGGGTTTGGAAGTTTCAGCGTTTTCAGTGACTTTGTAATAGTATATTCTaGAATTTaTATGTGTTTTGAGCTCTGCATCAGATAATAAAATTCCTTTTATGGTGTTTAATATATTCATTTATCTGGCCAACtCCTTTTttATAATTTCTCTATACTTACGTTCGCTAGCAGCTAATGTTTTtGCAATAACTCCAAAACCTCTTGGTGTATATTTTTttCCATCTCTTGTATAACCATGTTCATTCAAGTGAATAATGTTTTtGCGATTCATAGGACCTACCCATTCAATTAAAACAGCCCTTTCTtGACTACCAACTTTTGtATAaGGCTTAGATTTAGTCATTTCTTCTATACTGGCACCCGTATCTTTAAAGCTCTCGAACTCTTTCTTtAAAGCCTTTATAAAAAaTTCAGATGCTTCATTTAAAGCTTTATCACTCTTAGCCTGCATTGCTTGTTTACCGTATACCGATTCTAATTTATTCAACACTTCAGGTATCCCTTTAATTTCTACACTCATTTTtCTG

>NRS26contig01329 [organism=Staphylococcus aureus]

ttaaTGCTTTTAGTTCTTTAGATGGGTCGGATTTTGTAGATTTTACGCTTTTAACATAATTTGCAGCATCATGAACTGCTTTGTTATAACGATTACGCCTTGTAAaGTCTCCTAATACTACATCTTGCTTAGTGATATTATTGTACGCATCtcTATGTGTAGTGATTTCGACTATTCTCACTAAGTCGTTATATCCTATGGCAGAATCCACCACTCTAACAACATCACCTATTTTAGGGTtAgCTTCTGGGAAATGTTCACGTAACGCTACAAAGTCTAAGGAAaTAGAAGCAGTGACACTTTTCTTtATCAATAACTCCATTGCTTTTTTtAAACTATCTTCTTTTTtAATACGTCCATCAACAAGCGGTGGCGCTTCTCTTTtACCTATCAATTGTGCTAATGGATGAGTGAATTCAATTTGTAGTCCCGCTTCTGCAAAAGTCTGTTGTCCATCAAAATCACCATAACCTTTAATAAAGGTATAACATTTAGATGCATCTTCTTGTATTTTGACGTTATCAGCATTCACACCAGCTTTAATGTAATAATTGGCAAACTTAGATAATTCATCAtACAAaTGAAaCGTTTTaGTCTTTGCATCGtATTCATATtCGAGATGatAACGCtCAAG

>NRS26contig01434 [organism=Staphylococcus aureus]

gTAGaGtaCGgACAAGATATATTGTTGgTCTTtAGTAAGTGTATCAAATTCATCAGATATCAAGGGCATGTtATCACCTCCTtAGGTTGATAACAACATTATACACGAAAGGAGCATAAACATATGAACACAAGATCAGAAGGATTGCGTATAGGCGTCCCACAAGTTTCTAGCAAAGCTGaTGCTTCTTCATCCTATTTAACGGAAAAGGAACGTAACTTAGGAGCGGAAATATTAGAGCTTATTAAAAAaaGTGATTACAGCTACTTAGAAATAAACAAAGTTTTCtATGCATTAGATAGAGAACTTCAATACAGGGCGAATAATAACAAACTTTAACATTATACACGGAAGGAAAGATAGAAATGCcAAAAaTCATAGTACCACcAACACCAGAAAACACAtaTAGAGGCgAAGAAAAATTTGTGAAAAaGTTaTACGcAACACCTACACAAATCCATCAATTGTTTGGAgTATGTAGAAGTACAGTAtACAaCtGGTTGAAaTaTTACcGcAAaGaTAATTTaGgtGTaGAAAATTTAtacATtgaTTATtCACCAaCAGgcactctgattaatatttc

>NRS26contig01444 [organism=Staphylococcus aureus]

AACATGATCTTGAAAAaTGGCAACAATTTATAGATTACTGTATTCGAGATGTAGAAGTAGAAATGACGATTGCTCATAAAATTAAAGACTTtCCAGTAACTGCAATTGAaCAAACATATTGGGTTTTTGACCAaCATATAAACGACAGAGGTATtAAGCTTTCTAAATCATTGATGTTAGGAGCTAATGTGCTCGATAAGCAGAGTAAAGAaGAATTGCTTAAACAaGCTAAACATATAACAGGTTtAGAAAATCCTAATAGTCCTACACAGTTATTGGCTTGGTTAAAGGATGAACAAGGATTAGATATACCTAATTTACAAAaGAAAACGGTTCAGGATTACTTAAAAGAAGCCACAGGAAAAGCTAAAAAAaTGCTAGAAATTAGATTGCAAATGTCTAAAACCAGTGTGAAAAAATACAACAAAATGCATGACATGATGTGCAGTGATGAACGGGTAAGAGGTCTGTTTCAaTTTtACGGtGCCGGTACTGGAAGATGGgCAGGTAGAGGTGTACAaCTTCAGAATTTAaCAAaGCATTATATTTCAGATACTGAATTAGAAATAGCAAGAGATCTtATtAAAg

>NRS26contig1473 [organism=Staphylococcus aureus]

ttaTGCATTGCTAAacaaTTTtGAtGCCCTTtCCAAATaGaTTttGAATGCGCCcAAGCTTtATCTGAGgCATCTGAAtACTTTTGCTTAGTTTGATTGTAAATACTTCCTGtaGTCGATTTAACAGATTGCCAAGCTTTTCCAAACCATTTACCAGTACTATTAGCTATAGCCTTAGTGTgaTATCCTACAGAACTTTTGGCTGAGCTCCAACCTGAACTTAATTTGCTTGGAATCcTTTGATTCCaCTCCACATTTTTtCATTTCGCCGCCAAAATGATTAGCATTTCTGCCCATTTTACTAAAagCTTCGCCAGTTTTAgTTTTACGCCGTCCCAAGCATTTCCAAACCATTtCTTTATATTTtCTCTGTTTCTACGAGCTGTTTCTTCTTGTtCTTTAGCGTATTtATCgCTTTTtTtCTTTTGGTCTTCTCTgAaGTTaGACCACCAaCTTTtAaGGCCATtCCACCACttttcagtatttttatatacacgtccactggataaatccatttcttatcaatatctttattttgctttttaacaacatctactacagcat

>NRS26contig01511 [organism=Staphylococcus aureus]

ATTTGAGATAACGACGATGCAAGgTTCTTCAAAAAaGATTGATTTAGATAAAATATCGAAATCTTGTAATCGTCTTTTCTCATGAATGGGAAAGCTTCTGGATTGCTACTAAACTGATAATTGTATCTGTTTTCAACTACATATTTGTAGCCTTCTAAAAATTTACGCAaGTATTCTTTTAAAGTTTtATTttCTTCCATCCCTCATCCTCCtCACGCCACATAGGCGCTATTAATCTTCCTTCTTtCTTATTGAAAAAaTAAAAAaGATGATTGGGATGCTTAACATtAATGGAAAAAATATGACTATTGGTAATGACAGTACCGCCATATATAAGAAGAATTTATCAAAaTTATATTTTCTCATTTTCATTTCTCCTTtGTTTATATTTCCTTATATTTAAAAACTCTCAACGGCTCAAATGTGATCGAATACTCGCCATAGTGAGTTCCAATACCATATATCTTTTTATATTGTtCTATTGCTtCTAaTaTGTATTCTTCGCTTAATTGTAGATACTCAGACAATTCATACAAGTTACGTACGCCATAATTA

>NRS26contig01531 [organism=Staphylococcus aureus]

AGACCATTCTCTAAACTtATTTGTCAATtCGACGAgCcaGTCAAAAaTATTAGAACTGTTTTGACTGAACgCAATCATTAAATTACCAATACCTTTAAAAACGTTTCCGAATATTTTACCTATCTTGGGTAGATTTGTCTTAGTGTACTCGATAAACGCTTGTATCGCATTTTGTCCTGCTACACTATTAGCCCAATTTTGGAAATCTATAGACATGTTTtGCAGACCTTGTGACATGAATTTAAAtAACGGCATCAATTGAGTAAAGATATTGACTAATCCATCACCAAATCGTCCTGCAGCGTTCAATAAATCACCAAATATAGCCCCACcGATGGTATTCAACGACTCGAACGCTTTTTTtGCAGTGTTAGACGTTTTGACCCATTCTTCAAACTTACGCACATtCGCTTCAaCTAaCATTGATATCTCAGaCAAgAAAGGTTTCattttagttaacgcgcttgcaatacctcttaatcctgctgacatagcgttaaatattttagcttgattctctttaacaatatcacGCCAtGTATCTT

>NRS26contig01565 [organism=Staphylococcus aureus]

ATATCTAAAGGAGGGATGACAAATGTGGGTTCGTGAAATCACCAAAAaCAATAGTACGGCcTATCGCTATTTAGAGCGCTATACAGACCCTTTAACtGGCAaGTATAAAACAGTATCAGTTACACGTAACAAGAATAATGTACGTAGCCAAAAGGaCGCTCAATTAGAATTAAATAAAATAATTGAGCAACGTTTGAAACATTACAGTACGAAACAACTTGAAAaCTTAACGTTCCATGATGCGTGCGATGAaTGGTTAGAGCATTACAAGACACATTCAGGCTCGAAACCAaCCACTATTAAAGAAAaGAAAAGTAATACTAATACAGTtAAAAATGCTATTGATAGCAAAGTACTCATCAGCAAGATTACGCACACCTACTTACAAAaCATCATTAATGAATGGGCTAAATCACATAGTATTGGCCATGTTCAATCTCTTGTTATTGTTATTCGTTCCGTTTtCAAATATGCGTTTtAAaTATtATGAtCTGCACGatATtAGTGTGTTAGATAAAATaTACGTGG

>NRS26contig01594 [organism=Staphylococcus aureus]

GAAGAGTTGAAAGAAATTATAGCGAAAGAAGTTAGAAaTGCTATAAAaGGCGaGAAaCCAaTCAGCTCAGGTGCAATTTTCaGTAAAGTAAGAATCAATAATGACGaTTTAGAAGAAATCAaTAAAAAaCTCAATTTCGCAAAAGATTTGTCGCTAGGAAGATTGAGGAAGCTCAATCATCCGATTCCGCTAAAAAAGTaTCAGCATGGCTTCGAATCAATTCATCAAAAaGCTTAtGTACAAGATGTtCATGACCATATtAGAAAaTTAaCaTtATCAaTTTTtGgAGTGACACTTAATTCAGACTTGAGTGAAaGTGAATACAACCTAGCAGCAAAaaTTTatAGAGATAtCAAAAaCTaTTaTTTAtATATCTATGAAAAGaGAGTTTCaGAaTTAACtATCgATgATTTCGAATGAAGgAGGAACTACAAATGAAACTACTAAGAAGGCTATTCAaTAAAaaCaCGAAAaCTtAATtGACGTGTGGCATGGAAAtCAATGGttaaaagTG

>NRS26contig01678 [organism=Staphylococcus aureus]

AAGGCTTCATGTTCTtAACAATATCATCTAAATGGTTATCTAAAATTGGTGACACTGCTTTTAAATCATTAAGAAAAGGCTCCCATTTGCCTAAAGTATTATCTAATTCTTCTAATTTAGTTTTAATATAATTACAAGTTACATTAGGAATCAGGGACAAAAATTCTTTCTTTTTtACaTTTAACATTTCAATTGCATGTCTTAAATTCTTACGTATTTTGGGAaTTGTATtAATCAAATATTTTATtACATCGACAATTTTCGATGCATATTCATCATATATACCTTGAACATAGTCTGCTATTTTTTTAATACCATCATCGATATGGTCTTTtAAtATTTtCATTTTTCTTCCTAAATAaTTAGAaGgTaTAAcTAGACCCTGTACCatATTTTCGCCGCTACAATTAATTTGAAAaTTTCCATCTAAAATTGTtGCATCTTGTTGTTTCATAaTacTTCtAATaTctGCAATTT

>NRS26contig01729 [organism=Staphylococcus aureus]

TCtCTATCTGATAAaCCACGCTTtATCAaaTTAaaTCTCCTAACCATACCCCGTCTAAATTATCTGGAaGCACATCAGCCTCTCTTATTTCAATATAATCATGTTGTATTAAAGTTTCTTCATATAATCCCATCTGATACATCCTTTACTTACGTTTGCTTCTTATATAATCTGCATAATCTAAAACTCTTTGCCATTCGTCATCTGTCAaTTCTCCTTCAAGATGAGCTGCACGATGTTGTACTTCATCATCGTTTtCTtCAACCCACCCaTTAAaTACGCAGGATTAACATTTAAtGCAGTAGCTATACTTTCtATAGTAtCGTTTTTtAGATTTTTGATATTTCCGcTTTCaTAACGTTGTACAGTAGCTTCAGTTTTACCAATTTTTCTtCCTAGTtCGGCCAAAGTCATACCTTGTTTTTCTCTTGATtGTTTCATTCTTTTtG

>NRS26contig01798 [organism=Staphylococcus aureus]

TCACTTCCCCAAAACCTCCTTGACTCGATCTAAaATGTCTTTACACTCCGCTACTTCCGAAGCCTTTTgCTCCACGTTCTGAAACACTTTCGAATTCCTCCACTTGCTTTAGTTCAGGTGTCCATATAGGTACAATAACTAACTGTGCTATACGTTCTCCTTTTTCGATACGGTAACTACCTAGCTTATATAAATGACGTTCTTTTTCGAAAATCTTTTCGTTATCAATATTTCTTAAAAAGATAGTTTGCATTTTGTCATCTTCATGGTCATTCTTGATATTAATCCCTAAATTGCCGTGATATCCCGCGTCTATCTTGCCTGTTTCAATCACTAAATGCGTTTTACTACTTACACCACTACGGCTAGTTAACAGCCCGACATAGCCCTCTGGTATGCTTACAGCTACATCTGTTTT

>NRS26contig01801 [organism=Staphylococcus aureus]

TAACCACAATCCTAAATTAATAGGATCTGTGGTTTTGTTGGTTGTAGGGGAATAAATATAACCGTATCGATTAAGATACGGTTGTAGCGAATGTAACATTTCTATGTTGTTAAGATATATGTATCGAGTGATGACAAGGAAGACGTCTCCTGTGGGACCAACAGTCAGATACATGGCCTCTGCCCGGCTATATAGTTCACTCCTACTaTATAAAaGTAAGTATAaCATAAAaaGCACCCcGTAAaCtGTtATACGGGAATGCTAAAGtCATATATACTACGGGGAGTAGTATGAAAACTatGCTCTCTatCGTAAGAAAAAACACCCaGTGACATgCTTGGGtgAACAaGgATAGAtgtAAATaGTTGatgCATGtgtAACACATCATAGCAAAAAACTaGCCCgAaGGCTAGC

>NRS26contig01837 [organism=Staphylococcus aureus]

ATAAGAATAGgTATCAATGACAAACAaGGTCGTATCGATCTAaaGCGCATGGCTTAACACcTAGATTACATTTGTTTATGGAAGATGGCTCTATATTCAAAAaTGAGCCCCtgATTATCGACGATGTTGTAAAAGGGTTCCTTACcTACAAGATACCTAAAaaGGTTATCAAACACGCTGGTTATGTTCGCTGTAAGCTGTTTtAGAGAAaGAAGAAGAAAaaTACATGTCGcAAACTTTtCTTtCAATATCaTTGATAGTGGtATTGAATCTGCtGTAGCAAAaGAAaTCGATGTTAAATtGGTAGATGATGCTATTACGAGAATCTTAAAaGATAACGCGACAGATTTATtGAGCAAAGACTTTAAaGAGAAAaTAGaTAAAgatgttattt

>NRS26contig01888 [organism=Staphylococcus aureus]GTGGCCAACATCGTTTTTGCACTAGCAATTGCACGTTTTAACGGTGAATTATTACCATCTATTTTAaCGTTATGTTCACGCCATTTTTGCGCCATaGCTTTAGCGCGTTGTAAAGCTCTTTGGAATCTTGAAATATCTGCTTTtACATCTGTTTCAATTTCGTTTGGTACAGACGTCTTTGCTAATCGTTGAGCTTTCCTTACGTTGCTTTGGAAATCTCTAATaTTGGCCATAATCTTTGCCATAAAATGAGTATCCAAAGGCTAACCTCCTTtCGATTCAaGGAaTTTtCTtGTACCTTCTTtGAaGAGTTCACGTCTTCTTTTTtCTtCTtCTAATCTAAC

>NRS26contig01910 [organism=Staphylococcus aureus]

TATgcAGatACCTGATACACACTTCCAaCAAaaaCAACCACACTCCTAAaTTAATAGGCGGTGTGGTTtATTTttATACTCGTATCAATTtACAAACGTTTtATTGTGTAACTAAaTCTTTTTTTAATATAGCGTTTATCTCTGCTTCATTGTTGAATtCTCTAGTTAAACTATTCACGTATCTTTGCTCGCTTTCCATATCAtCATCCaTAATAAAtgTGaGaTTTTCTTTATTAAACcATGCAtGaGcTACaTAAgaTCTAAtGTTAAATAgcGCTTGAGGAgAATTATTtATAActtta

>NRS26contig01915 [organism=Staphylococcus aureus]

CccTTTCtAATTGTTATAGTGTTTATTTAATATCGATTGAAAAGTGGCGTTGATTTCTTGTTCTTTCATAGGTGgTTTACATGCGAATTGCCCCCATAATAAAGCAAATGAATAAACAATATAATCATTAACGTGACATCTTAATAaaTGCCCAACTAAGCTAGCTAGTGCATTGTTACGATTGCCTTCGGTTGTTCCAAAGCATAACTCTCGCCAATACTTACTATCTCGTCGCGTGTATCCTATGACACTCGGACTAACATTTGATTGTTCATACTCCTTTAACCaCTGTTC

>NRS26contig01923 [organism=Staphylococcus aureus]

TAAGCTTCTACAATTTCgCGTAAtGgAaCaGCTGAGATAAAGCCGTGTCTACGTGCGTAATTTTCGAACTTGCGATTGTTGAAcTtCGATTGGtCTAAAaTGTtGCCATACGTCAACTTGTGGTGGGCAAGTtCTTCGTATAaCaCTTCGGCTTTACGCcTTTCGGATAAaTTTTATtAaTtAAAaTAaCTCcaTtgTCATAGAGACCCTCAAAaTTACCTAAaGACTtCCCATCATCAATTGGTAtCTCtttgttttcaataaccatcttttcgtat

>NRS26contig01925 [organism=Staphylococcus aureus]

TCCTTGTCCTACATATTTTGAAGCATCCATTTCTTCTGGTACAGATTTTGTTTCTTTGTTGGATTCGTTATTGGTAGTTGAACATCCTGATAGTAGTATCGTTGCCATTAAGATAAATTTTGCTTTTTTAAGCATATCTCATCACCTATTTATGCGCTTTTAAAAGTTTCATTTTGTAAATAATTATACTCTCTTAATTTATTTGTGTCTTTTAGTTTCTCTCTATATAAATCTTCAAATACATCTGCCTGCTTAAATCATACGTGGCgT

>NRS26contig01928 [organism=Staphylococcus aureus]

cgtattgacaaagaaatacatgagtatgaaaagacgcaaggaTAAtGATAaGGGTAaGACTTTTAActATGagAAgaTtAAAAaTTtCAAGTaTTCATTgCTAAACGGCTGGGAaTTAAtGGAAGaTGAGTTAAAAACTGAATTCATAAAGATGGCAaTCAAAAACATTCATTTTGAATATGTAAAAGGAaTTAAAGGGAAGCGCCAGAACTCATTGAAGATTACGGGTATAGAGTTTTATTAATTGGAAGTTCGGAATAAC

>NRS26contig01935 [organism=Staphylococcus aureus]

GATCACTGCCTTTTCTTGTGGCTCAAGTACGACAGTTTCAGCTGAGAATATGTCATAACCTGCATCCGTCTTATGATTTCGTTCTGGCATTCTAGCGTCTTCTGATAATaGCTTTACTtGTAGGATGTTAGTCATTTTCCTtGTCCTCCTCATATTTATAGACAACTTGACCCGTCATAATTCCTATTGCTTCATCAaGATCAATATCTTCTTTGAGTGCATCTTGCATaGCATTAGGTAAACCCTc

>NRS26contig01967 [organism=Staphylococcus aureus]

CATATGTTTGTAatCcATACATAAAaTACTCATCTTCAAAcAATTGACTGGCCATCATATCgCTAATAGAAAGCTGTTTACCaTCATGTAATTCATAACCTACATAATGCCCCTCTATGCTTCTTATAAGCCCCTCAGTGTGCTTAGGTGACGCTAATTCAAATGATT

>NRS27contig00011 [organism=Staphylococcus aureus]

GtCTAAaTGTtCTTTtAAtGTGTCAAAGGTTTCGCCGTTTACATTAACTCGAGCTTGAACAATCTCATTAGCACTGTTATTACGTGGTGCCACAACAAGTGCGTTAATTTGACTTTGTAAAGATTTGTTTACTGCTGCTTGCGATCTACCATTATAATAAATTTGctCAGCGAAGTGTTGAATTGTTTtAGCTTTCTGATGCAACTTAAACTCTGTTGTCAAGCCAAGCGCAAATTGCTCTATTCTTTGTAAGTTTTGTATTTCCTTAGCTCTATAATCTCGACCTGCTAAAGCTCCCAAATCCTTTATTAAATACAAATTTTCCATAATGCACCTtCCTTtCTAATAAAaTAGCACtGTACCAAGTTTCCCACTATCGTCAACTGTTATTTTCCACAATTTACCGTTTGGGgATTTCTGTACAATGCtATTTTGAaTAATTCcTGCTTCGCcTATTTTtAAaTtAtCTAATTtaTTTTTATCATCTACCGAAATGATACCGTCTTGaGGcAaTCCATCAaTATCACTACTGCCTGCATAAGGTATCCCATTtATaGCTTtCCAaTGTGtAgCTGG

>NRS27contig00012 [organism=Staphylococcus aureus]

GTGGGTACTCAAATGACCAATCTAGAAGCAaGGTTTTTAatGAAAaGGGATtGCAAAAAATGaTtCTGAAaTTAAAAAAGTGgTAGGTGATTTTtAAAtGGAATTTAATGAATTTAAAGATCGCGCATATTTTTTCAATATGTAAATAAAGGGCCGTATCCAGATGAAGAGGAAAAAaTGAAATTGTATAGTTGCTTTTGTAAAaTATATAATCCTTCTATGAAAGATAGAGAAaTTTtAAAaGTGACTGAATCAAAGTCAGGACTAACCATAATTATGAGGTCTTCTAAAATTGAATATCTACCACAAACAAATCACTTAGTTAAAATTGACAGAGGCTTATATtCCGATAAATtATTCAaCATTAAAGAAATAaGAATTGAtACACCAGATATTGGCTATAATACAGTGGTTTTATCAGAAAAATGAGTGTAGAAATTAAaGGGatACCTGAAGTGTtgAAGAAaTTaGAatcGgTATaCGGTAAACAAtCAAtgCAagCTAAgA

>NRS27contig00016 [organism=Staphylococcus aureus]

tCtaatgcatagaaaactttgtttatttctaagtagctgtaatcacttttttaataagctctAAtATTTCCGCTCcTAAGTTACGTtCcTTTtCCGTTAAATAGGATGAAGAAGCATCAGCTTTGCTAGAAaCTTGTGGGACGCCTATACGCAATCCTtCTGATCTTGTGTtCATTTGTTTATGcTCCTTTCGTGTATAATGTTGTTATCAACCTAAGGAGgTGATAACATGCCCTTGATATCTGATGAATTTGATACACTTACTAAAGACCAaCAATATATCTTGTCcGTACTCTACAAAGATtATTTAGAATGTGtAAAGTTAGgTTCGGTtAAATTAACCTGCAATAATTTtgAAaGTGCTAAAGATATACATACAAAGTATTTtCAAAACTACATTtCGAAGATGTAAAaTACGATTtAAaTAAaCTTAAA

>NRS27contig00017 [organism=Staphylococcus aureus]

gTtGTTtGAATaGgagAtAAAAGCATGACACCGAACTTACAACTTTATAAtAAAGCGTaTGAAaCGCtACAAGGatATGGATtCCCTGTTaTTTCTCGtAAaGAGATGCAACAAGAGaTTCCGTATCCTTTTTTGTAATAAAAATGCCGGAGTCAAACAGAAGTAAATACACGTTTGATAGTTATTCTGGTGAcAcGAATTTAGTTATtGATATTtGGAGtGTAAGTGATGaTTTAGGACATCATGAcGGACTTGTTAAAaGATGTATTGATGaTTTAAcACCTAGCGTTAAAACAAACGATtATGACTTtGAaGAAgAAGATACTAACATCACACAGTtAgTTGATgaTACtACCAATCAAGAATtGCTACACACAtCAgTAaCGATAtCTTACAAAACATTTTAAAAaaCGgAGGAaTATtGAATGG

>NRS27contig00020 [organism=Staphylococcus aureus]

ATTTCCACCTCTTTtAACACAATTAAGTATTATGATACACAACTTGCGCAAAaGATGTAGACAGAACATAATGGCGAACAAAAaCAACCACCCAGTAACTAGTATGGGTGGcGTAGCGACTaTAACAACTCTATGTTATCAAGATATATGTATcGAGTGATGgCAAGGAAGAaGTCTCCTGcGGGACCAACAGtcAGATATATGGCCTCTGCCGGGCTATATAGTTCACTCCTACTATATAAAAGTAAgtATAACATAAAAaGCACCCCGTAAAcTGTTATACGGGAATGCTAAAGTCATATATACTACGGGGAGTAGTATGAAAACTATGCTCTCTAtCGTAAGAAAAaaCaCCcAGtGACATGCTtGGGtGAACAAGGATAgAtGTAAaTAGTt

>NRS27contig00021 [organism=Staphylococcus aureus]

TTAAGAATGGAGACTATATAAAATGACTGAAAATAATAAATTACAAACTATTGAACAACAATTAGTACAAGAAAaGAACGTATCTGACAACGTATTAAACAAAGTGAGAGTTTTAGAGTCACAAGGCAATTtGGAATTGCCAAATGATTATTCACcAAGTAATGCCATGAAACAaGCATGGTtACAAATCAGCCAAGATAaCAAATTAATGAGTTGTAACGATACAAGCAAAGCAAATGCCTTATtAGACatGGTAACGCAAGGTTTAAATCCAGCTAAAAATCAATGCtACTTTATtCCTtAcGGcAACAAAATGCaGTTaCAaCGTAGCTAtcaCGGTAATGTAATGATGTtAAAACGTGATGCAGGTGCTcAaGATaGTt

>NRS27contig00022 [organism=Staphylococcus aureus]

ACTTTaTCtGcAACaTTAGAAACATTACGGAtAACTTTACTTGAATGATTATCTATACCTTTAACGAAACCTAaCATTGAATACATACcAACATCCATGAATTCACGTGAAGGTGAGtGAATaCctaGCgCtcTTTTGgCTGCATTtAAAGCACCTTTTgCTACACTAGCtGCTTTTTCAGCTAAGTCTCTAGCcATATTACCAaTACCTCTCAtCAAaCcACGGATCATATCAGCACCTGCTGATaCAAAGTCaTCCACAAAGcTTTTAACTTTATTTACtGCATTTGTCAtACCTTGACTAaCTTtGTTTACAACATTAACGAATCCTTGAACAACTcTATTAAcAAAGTTAATTAGCGTACTTGTTATAGTAGATAC

>NRS27contig00031 [organism=Staphylococcus aureus]AATACTTCCTGTTGTCGATTTAACAGaTTGCCAAGCTTTTCCAAACCATTTACCAGTaCTATTaGCTATAGCCTTAGTGTGGTATCCTACAGAACTTTTGGCTGAGCTCCAACCTGAACTtAATTTGCTTGGAAtCCCTTTGATTCCGCTCCACATTTTTTTCATTTCGCCGCCAAAATGaTTAGCATTtCTGCCcATTTTACTAAAGGCTTCGCCaGTTTtACTTTTtACGCCGTCCCAaGCATTTCCAAACcATTTCTTTATATTTtCTCTGTTtCTACGAGCTGTTT

>NRS27contig00049 [organism=Staphylococcus aureus]

TTtCTAGTTTCCTCAGTTTGCTGACcTTGaGTAATGAAAATTaCTtGTtCACGAaGTGCATtCATTCCATCAGgtGCATGCATtACGTtCTTTtAGGTCTTtAaCTGTGAATTGGTTATCGTAAATTTTTACAACCATATCCATCAATCTGTCAGC

>NRS28contig00004 [organism=Staphylococcus aureus]

CcTtGTtGTCAGCTAGaCctAAAaGTTTTtttAaTTtATACaTTtCGatAaCATtAGCGATATcGTGGTAATCATTTtCGTTaTTCAatAAATtAGCAaGACCTaCAATATCCCCAAGCGCaCAATGTGACGATGATGTAGTATCtCCATTGCTAACCCcTACAGTTGAAAAAagTAAAaCGGCAAATTCAGTTTCTTTATTGATTTCATTCACTACTtCAAaCAaTTCTCcATtCTTTtCAGCCAaTAAaTCTCTTAATtCTtCcTGCATCATGTCTTTATAATTTTTAGTCATAGTTGACTTCCTCCGTTTTtCGTTTTATATTGAAAATGAATTAATTTTGTTAATCGTTTGTCACTGTTACTTGTTGGCGCAAGTAGCAGTTTTTtCATTCTTCATAAAaGTATTCCTTATAGAATATGAATGTTGCGATACTTGCGAATCCTGCGATTGACCATGCTGTAGTGAAGTACAGCAATGGCATAAGCACAATTGCTAAGACTGTGAAGCATAGTACTGCTACTAGGTAGCTTTTaTAAATGTTACTCATTTtCTTTTTtcTCcTCTTtGGTtGTTTCATCGTTTATCAAACCTTGCATTTCCATTAATTTTTGAGGTATACCaGCTTTtAACTGGATTTCGTATAaCATTTGTTGAATGTGTGGTGGCACTtCTACCATTCCTTTCGTGTATAATTTAGTTATCTCCTAGTGAAAGGAGGTGATAAGTATGGAATTTAATGATTTTCAAAATTtCTTTGGTGAACTTAGTAATCAAGCCGAAAAaGAATTCGGTGGTGACAGTGACTTTTTTAGAGATAGAATAAATAAGTTGAAAGAAGATGCTCCTGAAAACGTATCTTACGAAATTATTTATTCAATAGCTTTATACGAAAGCTTAAAAGCTCAACAAGATATGAAAATTTTGAAtACAGTTAAATATCTTTtAGATCGTGACTAGCAaTATCCAACAATGATTTGCTCTGAGCATTATTAATTTTtGGATAATCAAAaTTTCTAAGTTTAAaTCTTGTGTTTTtCTCAATCTTTACAACcTTCCACGTCACAACTGCCATTGTGATGAGGAGGgTtGTTTtGTATAGTGTGTTCATTGATAATTCcTCCTATTAAGATTTTtATTTTtCTCcTAAAAACTTATTAACAAAGTATtGTTGTCCTTTGCCTGTTACTTTTGGCGTCTTACTAATTGATGTGTGACCGTCCGAATGTGTGATTGATGTTTCTTTAATTTCGAATAACTCACGTTCCATTGAATACTGTGTAGGCATGTTATAATCCACACCCTTGCGTTTAATAAGGAATCCgTTTtGACGTAACCACTCAAACAATCTGCGTTGCCCGATGTTTAtACcGTTTTGTTtAaTGATCTTTGCTAACTCTCCAAcTAAAATTGATGTCTTAGTAGTAGCTACTGCATCTGCAAATACAATTTTTGGTTTaTCAcGTtCAATCTTTGTTTCTAATTGATTGaTTGTGTtGTTAGcAaTTTTTAAAGCACGTTGCaTAATCATTTCTGGACTGTTCCATGCTTTTTCAaCTTGGaTGAAaTaTTGtCTTGCAcGTTTACCGGGTTCacTACGTTGAATCATTGcGATTTCTTTtGCAGTGTCTAGTGTtAGTGCGTGGTCTAAaTAaTTAATAGCGTTACCTtGAGCTGTTACTCTTTTTtGAGTAaGAGCTGtATAATCAATTTTTtCTTCAAAGCCATAATTAATCATTCTTTCAAACCAATCGTTATATCTTGTCTTAACTTCTAATGCTTGATGAAGTTCTCGACcGCTGATTGCGATTTCTCCATTTTCTTTTtCTTGTATGTTGAACATTTCGCCGATGTTCGATTTTGTTtGTAATGCTTGCATAATGTTTATGCTCCTTTCGTGTATAATTTATTTATCGCTACTGCGATGgTGGGTGgTGATAAGATTGAAAACTAACTATAACTTTAGtATCAATGTTAGAAATGCCGGTAAGTTTGAAGAAACACCATGTGAATTTGTAGATGGTAGCAAAGGTGTTCGATTAGCTTACGAAAATGGTTTGgTCGTAACAaTCCAcGTTAaCGgCAaTAAtATTGATAtACGTTCAAGTCACCTATtAATTTtGGTTGATGAAAaCCCTTtAacTTTTGATGTTGATATGAATACAAAAAaTCcTAAatAaTTTTTTTACCATcAaCAGTtAAAGAcAaTGTattttattTTtGgAGaTGTAAGAGGtCtATtGtcGTTAGTAATTCCTC

>NRS28contig00013 [organism=Staphylococcus aureus]

gATGTgTGTatCAATTCTtGATTAGTAGtATCATCGACTAAcTGtGCGAtgTtaGTATCAtCTtCTTCAAAGTCATAatCGTTTGTTTTAACGCTAGGTGTTAAATCATCGATACACCTTTtAACAAGTCCGTCATGATGTCCTAAATCATCGCTTACACTCCAAATATCAATAACTAAATTCGTATCGCCAGAATAACTATCAAACGTGTACTTACTTCTATTTGACTCCGGCATTTTTATTACAAAAAAaGGATACGGAATCTCTTGTTGCATCTCTTTACGAGAAATAACAGGGAATCCATATCCTTGTAGCGTTTCATACGCTTTATTATAAAGTTGTAAGTTCGGTGTCATGCTTTTATCTCCTATTCAAACAACGCTTTCAATTCTTCTACAGTTGATTTTCTtATTACCTCATATACTGGCCACATAAAAGGTTCTGCCTCCATGTATCGAGTACCAAACTCTAAGAAACCACTATAAGCTGCATGCGATGTGATAGTGTATTGCAAATCGCCAGTTTTTttAtATCTGATATTGCGTGATAAATTACCAGTCCAATAaCCCTtATTCATTACTTCTCTAGCTTTCAATTTAGCTCGTACTACATATTCTTTGgCGTTTtCCTGTAAAaTATCATCTACATCATCATCAaTGTtGGTTTtCATATCGTgAAaTtGGTTtAaCAGTgCGTCTAaTCcATcTATATtCATCAATTGACCtCTTCgaTATAATA

>NRS28contig00017 [organism=Staphylococcus aureus]

AGAAGCAACTAACTTTATTTTagAGGaGatGGAAACAATGAAAATCAAAATTGAAAAaGAAATGAATTTACCTGAACTTATCCAATGGGCTTGGGATAACCCcAAGTTATCAGGTAaTAAAaGATTCTATTCAAATGATGTTGAGCGCAACTGTTTTGtGACTTTTCATGTTGATAGCATCTTATGTAATGTGACTGGATATGTATCAATTAACGATAAATTtACTGTtCAAGAGGAGATaTAACAATGAAAATCAAAGTtAAAAAgAAaTGAGATTAGATGAaTTAATtAAATGGGCGCGaGAAAATCCGGATCTATCACaaGGAAAAaTATTTTTttcAACAGGATTTAGTGATGGATTCGTtCgTTTTCAtCcAAATACAAATAAGTgTTCGAcGTCAAGTTTTATtCcAATTGATATCCCCTTcATAGTTGATATTGAAAAaGAaGTAACGgAaGAGACtAAGTTtGATAGGTTGTtAGAGGTATATGAGATtcAaGAAGGAGTCTATAAATCCGCATTACACAAaGGTATCAGTTtGAACGAaCGTTTtGAAGACGACAATATTTTtCCTACTAAAGCATTCTATATCTTAAACGATGACATGACGATGACATTGATTTGGAAAgAT

>NRS28contig00026 [organism=Staphylococcus aureus]

CTCAAAaGCATTGATAATTTCAGCTTTTGTTTGTTTTtCATCAACTtGTAAGCCAGCAACACTTGCTATTTCGACAAGTTCTTTTTtGGTtAaTTTGTCATTTACAATGTAAATCATTTGTTCGTTGCGTTTATTTTCAACACTAGCtAAaGCTTtGATACGTTCATCTGTAGGATCATAACCTTtGCGAGGGTAGACATGCCCTTTCATATAGACATGTCTGTTATCTTCTAAaTCTGTAAAaTCTACTTTAACAATTCCAATGATTTCGGGCATGTTACCACTCCTAATTATTTATTAAACTTCTCcTGGTACTGAATCTGTTTTTTtGtCAGCAGGCACTAATTTAGCGAATGCTTTatCGTCAGCGATGtGTAACGCTACATGCATAGTTGCACGCAATGCcACcaTGTCTtGttcgaataagtttacaggtgtgccatcttcgtttttaaactgtagat

>NRS28contig00027 [organism=Staphylococcus aureus]

GACGTATTTACCTAAATCTAATTTtGTTTCAAATAAAGCTATTACATCATTATCCCTACTCAAAGCTTCGTTATATTTTCTTCTACATTAGCTACATACTCTTCCACATATTCACGCAATTCTTCGCTGTAATATTGATTTCTCTTATAATTTTGAAAaGCCTTATTAAACTCAAACTGTGTTAGGCCTTCATATTTAAGGCTGAAATATAACTCACTTAACTCATGGGCGAATGTACCTTCTTCGGCAAAAACTGTACTTTTAtCTGCAATACCTTCACTTGCCTTAATACTCGGTGGGCAGTTTAGCCATTGTTTTGCTCCACTTGCACTAaGCTTTGCATGAGCTCTATTTGAGTGATCTAGCTTCATGCATTTATtCtCGCaTTCATAAAATCAaCAATTTTttCATAATGcTCTTCTTtGaTAGTAGATAGcTtAT

>NRS28contig00029 [organism=Staphylococcus aureus]

AAAaTaCACGGTCGATtGaCGCTAACAAAGATTtCAATACTGTTAGATACGTATACAaCAGAAACTTACcTAAATCATTAATCGAAGAATTAAAAGCTTATATTGATTCTGGCGGGAAGATTAGtCAAACAACTTTAATGTCTCTATtCTCGTTCTTCCAAGACCcTGAATTGGAaGTCAAGAAAaTaGAAGAaGATGAGAAAGAATCTATTAAAAAAGCTCAAAAAGGTaTTTATAAAGACCCTAGAGACATCAATGATGACGAACAAGATGATGATACAAAaGaTACTGTTGATAAAAAGGAaTGATTGTAATTGCcTAACAAAAACaCTCAAGAATaTtGGGAAGAACGCGGACgCAAAGCAATCGAGAATGAGTtGAAGCgTGATAAAACTAAaGCTGAAGAAATaGAACGtaTATTGAatATGATGATTAAGCGc

>NRS28contig00037 [organism=Staphylococcus aureus]

ATATTGCAAAAATTGATAGTAGAACAACTAAAGTATGCAAGGGGCTTAATGGAGAAATaTTCAAAGTTAAAgACGCTAAAaTTGGTGTTAATTTCTACCCTATGCATATCAATTGTCGTTCAGATTGTGCATTACTACCTAAATCTATGTGGCCGAAAAAaCCAAaCAAAAAACGACAAACAAAATACTTTGGAGGAAAAGTGAAAAGCGATGATtGATTTAAAAGTAAAAGTTTTTAaaGGCAAGTtAGCATTGTATGATAGTAAATTAAGTGTTTGGAGGaTaTtGGtATGAGCAATACTGACAAATACCTTAGAGACATAGCAAGAGAGTTAAAAGgTA

>NRS28contig00039 [organism=Staphylococcus aureus]

ACACAAAATGCGTTAGATTTCCCGTTACCGAAAGCGTTTAGAGATGCAGGTTGGTTCTTTGATGTACTGCCTGGaCACTATAAATGGTGCTCTAAGACAAGTACTTACCAGAAACaGCACAGGTAgAAATATGCTTAAATtCGAaCGTGTCATTGaCaTTTTCAATAaGAAAAACAACGGaGCATGGAATTTCTGCCcGCAAAACGCCGGTTATTGGGAACATATCCCTAAGAGTATTACAAAATTATCAGATTTAAAAATCGTtGGTTTAGATTTCTATATCACTACTGAaGAaTCAAACCGATTTACTGATTTTCCTAAAGACTTT

>NRS29contig00009 [organism=Staphylococcus aureus]

GCTTGGTTGAaTAAGTCTGATGCCATATCTAAGTCATTCTCATCTACGACATAAGCATGTTtAATTGGTACGTTGTTCaTATCTTtAACTtGTATTGATATGCCCATAtGACCTTTTAAAAtGAATAGCTTAAAAatCGAaTCCGTtAACATGAATATTTTTGCGTATGATTTCGCCTATTTCGtAATaCATCTTGaCTtCcTCcGTTTTtCGTTTtAtaTTGAACATGAaTTTTTtCTtAAGTGTTTtGTTGATaCTGTTACTTGTTgaCGCAAGTAGCAGTTTtttATtCTTCATAAAaGTATTCTTTgtAgTaTATGAATGTTGCGATACTTGCGAATCCCGCAATTGACCAtGCTGTAGTGAAGTAcAGcaatGGCATAaGcaCAaTCGCTAAGACTGTGAAGCAcagTACTGCTattAaGTAGCTTTTATaTGTgTCGCTCATTTAATATCCTCCTAATACCATTTTttAtGCTTTCTGATCAAATACtCTtCCAATTTAGAAATATTAATCAGAGTGCCTGTtGGTgAATAaTCAATGTATAAATTTTCtACACCTAAATTaTCTTTGCGGTAATATTTCAaCCAGTtGTATACTGTACTTCTAcATACTAgAGGCGT

>NRS29contig00011 [organism=Staphylococcus aureus]CCCAAACGATAACTTGAAAGGGGCTGTTAAATATTtAACTATTGCATTTGGtCTATCATTTTCTTGTTTCTTTCAATCATTTTATCAAAATACGTATCGTAtCTTTGCCATTCTTCTTGAGTAATTGGCGTCATATTTAATACACcGCCAAGATCTTTGATTGCATATAATAGTAAAAACATTACATCTTGAACAGTAATCGTTTTGTTAAGTAATGTCTCTAGTGAGGCCATACAAGATGGTTCAATTTCAGGGTATTTAAATTtAGTCACTTCACCTTTTAAAGCATGTTCATAAAATGTTTGCATCATCAATGCACGTtCTGAACcaGAGCCTTCAACACAAAGATAAATTtGTACAGCAATACCGCcTCTAACTCTTCGTTGCGATATGCCTGCAAATTTCTtACCATCGATACTTAAGTCAAaTTTTCCTGGGCAATAaGAATGTtCAATTtCCAtCGTATCAAtATCAAAATTCTCATCTtCGAaCATTttGCTAATTAAGAGATACATCACAGTAAACGCTTCATCAATCGTTGTTTCtGTTTGTCCTTTGAaCATCAGCGATATATTTAATACACC

>NRS29contig00019 [organism=Staphylococcus aureus]

CTATTTTAGGGTTAGCTTCTGGGAAATGTTCACGTAACGCTACAAAGTCTAAGGAAATAGAAGCAGTGACACTTTTCTTTATCACTAGCTCCATTGCTTTTTTTAAACtATCTTCTTTTTTAATACGTCCATCAACAAGCGGTGgCGCTTCtCTTTtACCTATCAATTGTGCTAATGGATGAGTGAATTCAATTTGtAGTCCCGcTTCTGCAAAAGTCTGTTGTCCATCAAAaTCACCATAACCTTTAAtAAAGGTATAACATTtAGATGCATCTTCTtGtATTTTGACGTTATCAGCATTCAcACCAGCTTTAaTGTAATAATTGGCAAACTTAGATAATtCATCATACAAATGAAaCgTTTTAGtCTTTGCATCGTATTCATATTCGAGaTGATAACGCTCAAGTCCTTTTTTAAAGATTTCTAATCGTGTATCTCcTTtGCCTAAtCCcTcGAATTTAGATGCATCtACTTTTG

>NRS104contig00002 [organism=Staphylococcus aureus]

GGTGgCGTCATTGGTTTAGCTGGTGCGTtCTCTGTAGCTGGTGTTGGAGCGGTTGGCTTCGGTGCAATGGCTATTACTGCACTAAAAaTGGTAAAaGATGGCACATTGgCAGTAACAAAAGAGgTTCAAAACTTtAGAGATGCGAGCGATCAGTTAAAAaCTACATGGCAAGGCATtGTAAAagAGAATCAAGCAAGTATCTTTAATGCGATGtCAGCAGGTATCAGAGgCGTtACAAGTGCAATGTCTCAATTAAAACCATTCTtATCCGAaGTATCTATGCTGGTCGAaGCAAACGCACGCGAGTTtGAGAaTtGGGTTAAAcATtCCGAAaCAGCTAAGAAaGCATTTGAAGCATTGAATAGCaTAGGTGgcgcAATCTtCGgAGATTTATtGAAcGCCGCAGGAcGATTTGGCGaCGGATTAGTTAACATTTTCACTCAaTTAATGCCGTTGTTCAAaTTTGTGTCTCAAGGACTACAGAACATGTCTATAGCTTTCCAAAATTGGGCTAATAGTGTAGCTGGTCAGAATGCTATTAAAGCGTTTATTGACTATACTACCACTAACTTACCTAAGATTGGCCAGATATTTGGTAATGTATTCGCTGGTATTGGTAATTtAAtGATTGCTTTTGCTCAAAACAGTTCTAATATTTTtGaTTGGTTAGTTAAATtAACTTCGCAATTCAGAaCATGGTCTGAaCAAGTTGGACAATCTCAAGGATTTAAAGACTTTATAAGTTACGTTCAAGAGAATGCTCCTACtATTATGCAGTTAATCGGTAATATCGTAAAAGCGTTAGTTgCTTTTGGCACTGCAATGGCACCTATAGCTAGTAAGTTGTTAGATTTCATCACTAaTTTAGCTGGTTtCATTGCTAAGTtGTTTGAAACACATCCAGCAGTAGCACAGATtATtGGTGTtATGGgTATTTTAGgCGgTGCATTTTGGGCTTTAaTGgcTCcAGTTGTTGCTATAAGTAGTGTGCTTACAAaTGtGTTtGGTTTG

>NRS104contig00003 [organism=Staphylococcus aureus]GTTTTaGCAACAtGATAATAGGTCATAACGGCGACGGtAtCAACGAGGTAAAGACGCGCGTGTTGATAATACTGGTTATGATCATAAGACATTGCAaGATCGTTTGTATCATGATtATtCAAcACTAGaTGCTTTCACTAAAAaGgTtGAGAAagCTGTAGaTGAACACTaTAAaGAATATCAAGCGACAGAAtACcGATTTGAACCAAAAGAGCAAGAACcGGAATTCATCACAGATTTATCGCCATATACTAACGCAGTAATGCAaTCATTTTGGgTAGACCCTAGAACAAAAaTTaTTTACATGACACAAGCGCGTCCAGGCAATCATTACatGTTAtcTAGATTGAAGCCTAaCGGaCAATTTATTGATAGACTGCTAGTTAAAAaTGGcGgaCaCGGCACACACAACGCCTATaGATATATCGGCAaTGAGTTGtGGATTtATTCAGCAGTGTTAGACgCTAACAACAATAACAAGTTTGTACGCTTTAAATACAGAAGCGGAGAAaTGACGTATGGCAACGAAaTGCAAGaCGTTATGCCAAATGTATTTAACGATAGAtATACGTCAGCAATTTATAATCcTATAGAAAaCTTAATGgTTTTtAGACGTGAATATAAAACTTCTGAACAACAAGCTAAGAACGCATtAAATTTtGTtGAAGTAAGAAGTGCTGACGATATTGATAAAGGTATAGACAAAGTACTGTACCAAATGGATATCCCTATGCAaTACACATCAGGTACGCAACCTATGCAAGGTATTGCTTATGATGCAGGTATCTTATATTGGTACACTGGCGATTCAAATCCAGCTAACCCTAATTACTTACAAGGCTTCGATATCAAAACGAAaGAATTGTTATTtAAACGTCGTATCGATATAGgCGGTGTGAaTAaCAaCTTTAAAGGAGATTTCCAAgAGGCTgAGGGTCTAGATATGtATtaCGaTCTaGAAaCAGgACgtAAAg

>NRS104contig00004 [organism=Staphylococcus aureus]GTATATAACAAATTAAAAaGTAaCCTTATTAtCCGAAAACAaTTGGACGgTaGGGTTTTTGATTGCGTTCAGAAAGACGCtGTTTACCcaTATATCGTTgTGGgTGAAACAAACGTCACTAACAAaGAAACGACCACGAGCATGGTCGAaGATGTCGGCATCACTTtGCATGTTTACAGTCAAGCACGTAATAGAGATGAGGCATCGCAAATAaTTCAaTTTTTAGGCTTCGTTTTAAACAaTGAAATAGAAATTGATTATTATTCATTTATTAAAAGTCGAATTGATACACAAGAAGTGATTACTGACATAGATCAGTACACTAAACACGGTATCATTCGGCTTGTTTTtAAATACAGACATAACACATTACAAAGGAGTGTAACGAATGGCGCAGGATAAATATATTGTCGCtCTCcAAATCGCTGATAAGGATTTAGCTAAGAAGCTAACCATCGAAGAAGCAACGCTTTTAGGTAGTTTAGCAGAGGGTGGGCACACTATCAGTAATGACCTTGCTGAAATCATTCAAGGCGgTAAGAAAGATTATAGCCGTAACTCTGTCGAAGAAGAAATCAAGTTGACGCTTGATGTCGTTCCGGgAGATAAAGGTCAATTAGCATTAAAaGAaTCAGTTAAaCAATTCAAACaGTTACGTGTTtGGATTTGGGAAaCTAAAAAaCGCGATGGCAAaCATCACGgTGTATTCGCATATGTAGTtATCGAaGAGCaCGAATGGTCA

>NRS104contig00005 [organism=Staphylococcus aureus]TTGAAAGTTGGATAAATCATCTTAATTTTCTCCATTTAAACGTCCTCCTCTGTATTTGTTTTACCAGCTTTTAGTTCAGTCAACTGTTGTGTTAACATAGCGTTTTGTTGCTTTAATTCCATCGCCAAAATGTTTACTTGCGTCACCTGCATTTGCATACTTGCAACCATTCCGCGAaGTTCTTCATCACTCAAATCTGATTcACTTtGTtGTTTTgaTGCATTCGgTACGTCTTCTTTTtCGAAATTGCTATTGTATTTAATTTCGCCGTTAGTGAAAACAAaCTTTCtAGGTtCgAACTcTtCTTtAAATTtAATAGGCACATTGTtATCATCTACATCTAAACTATTGCGTAAACCGCCAGTATTAACGAATCCGATAACTTCGTTTTtATCGTTTACTGTGATTTTCATTATTTCCACCCCATAATTTTAGTTATAGTAACTTTGTTGGCATTCGCTCCAGAACCTGATGTTTTACCTAAATCAAAGTACACATCGTTATCTATTCTTAAAGTAGTGCTACTTGTTTTGGATAGTAAGCACTCATAAATACCGCcACCGTTGCCGTCTGAGTCAACTACATTCGCTTtACTCAATTGAATCGCGTTAGGTAATGCGGTTAGTCCGAATCCCTCAATAACGCCACcTGgAtAAGTtCcACTtaCCAaCAAAATAGAaTAgTTTGt

>NRS104contig00006 [organism=Staphylococcus aureus]GAatAatCCAGATtCAAAaaTACtAGTGAtGACAGCTaCAAGACAATGCccTATGAGTGGTACAaCAATACGCcGTAAAGACACGGACAAAAaCAAACTAGGGTTAACACTtGAGGACTATGTAAaCGCTCAAaTATTAGCTtGTAGTGAGTtAGATGTACCAGTGTTTGACGCATATCACACAGATtACTTTAAGCCATACAATCCAGCTTTAGGAAAGCGAGCATGGAGGACGGCTTACACCCTAACGAAAAAGGTCACGAGGTTATTATGTACGAGTTAATCAAGGATTATtACAGTTTTtACGACTAAAGGAGGCAACCAATGGCTTACGGATTAATAACAAGTTTGCATTCTATCACTGGCGAAAAaGtAGTTGCTCAGCACGAGTACAACTATCGATTACTTGATAATGGAATGAGCAAACTTGAGAAAATGTTTATAT

>NRS104contig00007 [organism=Staphylococcus aureus]GAGAAAACGACACGTTAAATCTATTACAGGGTGCATTGGACGTAATGAATAATAGCGgTTTTTtCAAGCAGAAATCACGTcTATTCTGGACGCAGATGAACCAAGCGCCAtCGTTAGCCAAAGAAGACGAGAAAGAGGGCGCGAAAGCTGGTATCGAGATGATGAAGAACAACTACAAAGAAATCATGACCGTaGCACCTTATtAGACTATTCGGAAATAAGGCAGATGACAAGTCGTTACATAGGTTATATGAGTAATGACGAGCTGATGAGCATGCTACCTGCCGAATGGAATGACTGGATTATtGGCGCTAGACAAGCATTGATTGACCAAAGaGACATTGCGTTGTACGGCGcTCAATATAATGCGGTTGCTCAAGCTGGTAAaTCACTAAAaCgTTTgTtaGgCAGAaCGAAAGAGAACATTACaTTATTCGt

>NRS104contig00008 [organism=Staphylococcus aureus]CGTGCTATCTTCGTTCAATTTTTCGTTTAATTTTGTATTAACATGAATCGCATGACCGACGCCTTGCAAACTTTTTAATAATACCGGCATATGCTACTCCTTATCTGTAATATAATTTGTGTCTAAAGACTATCTTTTTCATAAGTCTGTTGGCTTTGAAATGATtCCAACCGGGgTACAaCACCGGTTGTTCTAACGTCTTGTTGtATAGGTCAATATTTAAATTGCCTCTAtaTGTGTgcttgttatcaaaaaTGATTTtAtcGCCTGCTTTtAAaTCGaCAtCTTTAaTtACTgaGATGTTTCcTTtATCcaTATAGAAaGTGAAACCGTCTTtATCatCAGCTTtAAcatcttcggctaattcaatttcaactacatttgaattggttgaactgtgttaatgctacatctccgttgtaataaacatctcc

>NRS104contig00009 [organism=Staphylococcus aureus]

TCCTTTAAATCCCAAAAatAGTTTTtAACAAGGCtATAaCAAATGTACTTAGAATCGTCCcTATTAaTCCTAGAATCCACATCTTGATGTCTCTAATATTTTtAGCATTTTtCTCTTTATTTTTttCATCTTCTTCTTTGTCACGCCTTAGTTCTTCGAAATTtCTATCTAACTTGTCATAAATTTTTtCTTGCGTTCTCAGACTGTCTTCTATTCTGTCGAATTTTtCAAACATAGTCTTATCATTTTCTTCTAATCGCGTTAAACGCCAATCTTGTTCGTGTCGTTTGGTAAATCCAAACATTACACCACCCACTTTTtGTTAAATTAAAAaGCCACAAGCATTACACCTGTGACTTTTCATCTTTtGTTTCTGGATATTTTtCACCaGTGATCAATGCATATTCTTCTTTgTCg

>NRS104contig00012 [organism=Staphylococcus aureus]GTTTGTCGTAGTGATTCATCTtGTTtCTGTTCATGCCTcTACCTACTGCTtCGTAATGTGCTAGGTCAGCGTGAGGCTTtCCGCAAaTTACACAGTTGCGGTTGATTGTAGCCCAATAtAATAacGCTTTATCTTCgCTTAACAACTTaCtcGTTtCTAcaCTCATAGGTATTTGATGATGAAaCATAAACGCTATAATCAGTTCTATTAACTCCCTTGCAACTTtCATtGAACAGTCaCGCAGACTGATTtCTtCATAACCTTTCATAATTTCCAATTCTGTTTGtAATAaTTTTCTagTTGATtCcActGGTTCgCCCCAgtgAAGTTCTA

>NRS104contig00014 [organism=Staphylococcus aureus]

ATCTTTGTCAAGCTCTTCGTCAAAACTAAAAaGTTTGTTTTTtCTTTGCATTAGATTGAATTCATTTGCATAATAACCATTATTACcTTCTATTtGAAAAaTAACACCTACAGCAATATTAGAAATCAATAGAATATCTGGATAGTaGTTAAAaCTTGAATACTTTACTTTATACACTTTTCATCACCTCACTAAATGTTtCGTTATTTCTTCTATTATATCATCAAATTCACTAATATTTCTTTCGATAAACTTTTTCAacAaGTAAATATCTT

>NRS120contig00018 [organism=Staphylococcus aureus]

AAAGTTAAGTTATCTACTTCGCGCTTTCCTTGTCTTAATTGTGAAATTACATATTGCGCTACGCCAGTTTGTTTGTGAATTTGGTAACCTGTTATATCACTTTTGATCAATTCAATTATTTTTtAATTTATAATCACTcATATTATCTACATCCATTCTTTTtATCTAAACAaTAAAAaaTGtGTTTTtCTCCCGATAAATAATAACAATGGTAGGCTTAATAAAAaCAATAATAAATACATTCGTTCTGTCATAATTGAAAACCTCCAAATAaTATTATaTTATATAAGTGTAAGGAGGAGCCATCAGGCTCCAAGcATAATGTTAATCTTTGTTGTTTGGCTTTCGGTCTAGGTAGCCGAGATGCCATTCTCTAAGTtGTTTtAACACTTCTGGAATTATCAGTACTGCCAATACTTGATGTTCTAGAAGTGTTTTTATTATGTCTAGCATGAGGCTTTTCACCTCCTTACGCATAATTtGTAAGTCATCAACTAACCTACAAATATAATTATaCTAAaCAAGTGTTTaTTAAGCGAGTGTTTTTtAATTTGCATAAAAaaaTAGGCAAGTA

>NRS120contig00025 [organism=Staphylococcus aureus]

aaaaaTACagTTACAGAaCCggaGCAAGTTGTAGTAaaTCCgttGTTTGCgAAACCTAATgCACTAGCTagTATTTTtGGAATTTCATatAGTTCGGTGAATCGCATTTTAAaaGAaTGGgAAAAaGATTCTAAAGGTGTTGATGATTTATATTACTCGTTATCATCAACAATGATTGTTATAaGTATTCCaCGATTCGAGGAGTACATGAAGGCACGTCATAAAAAATGGATGTAGGAGGCAAGGCAATGAAAATGTATTTAGCTTATATCTGCTTAGTTTCATTGTTAACAaTTTtATTACTAGCAATATCTAACATGTATGTCGCTTTtAGCGTTTAtGCTTGGCTAATAACTTTaGGATGTAATTTAaCAGgAGgATTAGAAAaTGAATAAtGAACAAAaGAaGTAATAGAACACTTGGTTTatCAACTt

>NRS120contig00029 [organism=Staphylococcus aureus]

CTAACCAAGATGCACCATCTTTGATTTTATCGCCTGCTGCTTCAACCATTTCTTCTGCACCTTTTTtGATATTATGCGCTGTGTTTTTAGCTGTAGCTCCAAATTCTCCTGCTTTTTtACCTATATTACCTTTAAGTTGGTCTAGCCAATCTTTCTTTTtCGTACCTCCATGAAACTTTGGTAAAaCaCCCATACgCTGTAACTTCAGAGTGTCATTAGCATTTATTACGCTATCCCCAAcTCCTAGTGGAACAACCACATCtCGtCCTTGGGGTgCATGGAATGTtCCGTCAGCCCTGTGAaTTACTtCTTGAACTCCACCACCTGGGgCGTTTCCAGAACCTCtAtcATTTAatACaGCAAATGtCGGTtGCGTtAATGCTCCcGAATTAtCGgTAgCtAC

>NRS120contig00042 [organism=Staphylococcus aureus]

TTTGGTTATAAGTGACATCGAACCAGTACGGgATCACGTGAATaGTTTTtGAGGCGTtCCATCATAAAGCcAtGGTCTCAaTCTtCTCTTTCTTTCTTCTTCATATTCCGCTCTCACATTTCGTTGGTATAGGTTCAAAaTCGCTTTTTTTCtgATTTTTtCtCTCTctttttcttcatcttttatttgactcttcatatattcaacttcatc

>NRS123contig00014 [Organism=Staphylococcus aureus]

CTAAtGTTATAATGCCTCTATTTGTATCAATtCCcACTCtAaTTtATATCTCGATATGCATACACACCATCTAAAaCAAAaTCAGTGTTTTtATCTATACTTTtGTTGTACTTAAAaaTATCACCTGTTGTATAGTTAACCAGTtCAAATCCTCCaCTCGCATTTAAATTAATTAATATTTtCAAaTCGTGCTTGAaTCGTGGATTTATCGTaTCAGTAGAACCGTTCcAAATaGTAAATTGATTtGATGTATGAGtatATTTAGGtGtGAAATcAAgAGGAATtCcATTTT

>NRS145contig00083 [organism=Staphylococcus aureus]

GACTAGCAtGTCTGaTTTtGAAATGCTTATGGTTGTATTAACAATCATTGGTTTAGTATTGATTAGTACTCAAGaCCaTAAAAAaTAACCTTCTATTCGCTTTGACCGGCATTTTTGAAGGCTATTTttAAaTAAAATATAAGGTCACCGTCTTTTtAACGGGCTCATTAGGGTAACATGTTtCCGAGTGTtGCCCTTTTTGTGTTTCAAGAGTTAATGATTTTCATCTTTTGCTTCTACTTACTACAAATATATTTTAACACATTTTtCTTATGAATTGTAGTTCTGAACATAATCAGAATTAATAAAaCCAACTTTCCATACAGCAGAAaaTACAATTAAAaGTATAGAATGTAATCACGAATTATaTAAAaGTATTGGAGACCTCTTCAtATATAGATAATTCACTTAGTTATTTTAGAAAGAAGCCCCTAACAACTAAAGTtGAAAAaTAGaGGAaCACAGTTGGATGCTACGCATCAACTGCaTAaGAGCCCCcTAAcAaCTAAaGTTTAAAaGTaGgAgCGCCTGCGCTAAGCGCATGCaTAAGaGCCCCTAaC

>NRS145contig00092 [organism=Staphylococcus aureus]

cctagtattttcttcttggttcttcatattctaaagcttggtggctatcacctatacctttaGTAGTCGGgTCTTGAaTCACACCAGTTAATACTAAAAaTCCTAATATAGCGTTTAAACCGTCTGTTAATTGCTCTgTATAAACTTGGATATCATACCCAATAGCTTTTGCGATGTTTtGAGCAAAaTAAAAaGATAGCTGACAATATCGCTACCCAAAaTGATTTTTGTTtCATTCTAATTTTCCAATTAATCATATTCTTaTCTCcTTTTACCCAAAATAAAAGACGACTAATAAGCCGTCTATTTGATATTTATATTATGGTGTGTTAATTTATATATAGAAAAaGGGCAACATGCGCAAACaTGTTACCCTAATGAGCCCGTTAAAAAGACGGTGGCTATTTTAGATTAAaGATTAAaTtAATAaCCATTTAACCATcGAAaCCAGCCAAAGTTAGCGATGGTTATTTTTTATTGCTTAATTCaaTAAGCTTGATtACTAGACCTatCAatGCAATAAGGAaTAAaCCAAACTGcAACaTGGTAC

>NRS145contig00201 [organism=Staphylococcus aureus]

ttctactgatttggaagttacgtatccgcacgagattatatcaattggcgatacagtcagagtaaaacatagagattttaacccgccattgtatgtagaggcagaagttattgctgaagaatatAACATAATTTCAGAAAATAGCACATATACATTCGGTCAACCTAAAGAGTTCAAAGAATCAGAATtACGAGAAGAGTTtAACAAGCGATTAAACCTAATACACCAAAAATtAAaCGACAATATTAGCAATaTCAATACTATAGTAAAAGATGTTGTAGATGGTGAATTAGAATACTTTGAACGCAAAATTCATAAAAGTGATACACCGCCAGAAAATCCAGTCAATGATACGCTTTGGTATGATACAAGTAACCCTGATGTTGCTGTCTTGCgTagatattggaatggtcgatggattgaagcaacaccaaatgatg

>NRS161contig00011 [Staphylococcus aureus]

AaGTTGCATTGGATGGTCGGGTTTGACTGTCCTAACCGCGATGGCACTGTAGCACATGGTGGATGTACATTTTGTtCTGCTGCAGGTAGCGgAGACTTTGCAGGTAaTCGTGCAGATTCAATCgCAgTACAaTTTAAAGAAaTTAAGGAAAaGAtGCATGaGAAaTGGCACGAAGGAAAATATATtgCTTATTTTCAGgCATTtACAAAtACAcATGcACCAgTTgAAGTaTtAAAAGAAAAAATTCG

>NRS408contig00008 [organism=Staphylococcus aureus]

tCAtGAATGgAGATTTtAAGTTAtGAATGTAGAAATTAACGGAAAgTCATTAGAaTTAAGTTTtGgTTTTAAATTTTTAAGAGAAaTCGATAACcGATTAGGTTtAAAAGTTGAACAaGCTTCTATCGGTCAAGGTGTATCAATGTTGCCTGTAGGTTTAGAAAGTGGAAATCCGGTTGTGATTGGCGAAGTTTtAATCGCaGCTACATCTCACTTAAAAAAACAAGCAATTACTATTAATAACATTGATGAAGCATTAGATGAAATCGCAGAAAATATCGGACTAGAAGAATTCGGTTCGGATATTTTAaCGgAGTTGGGAAAGCGACcTATGACCCGAAACCTAGTCGAAGTAGTGgAAGCGGAAGaGAAACCAGCGGAAGCGTAATAACTTACGACAGAATCGTTATAAcTTGTAtGTCAACACTTGGTATTACAGATTTGAACGTTATTGAGCAAATGACATTAACAGAATATAACTaTCGAATGTATGCGAAAGAGTATGAAaTGCTAACCCAAGAATTCGAACGTTACAAACTTGCGTTTGCTATTCGTGATGCTGCAGCTACTAAAAaTGTTGGGACAGAAAATAAACCTAAAGAGGAATATGTTTTTAACAATGCAAACGACGTATTGCCTTATGAAGAAAATATCCAACGGCTTAACGAAGGTAAaGATATAAGATTTAGCAGCGAACGTGATGAATACGAACCACAAAATAATGAATTCTTTAAAGTTATAGCAGAATTTAATAAGCAATAGAAAGAGAGGTGTTAATGTGACGGAATATAAAATTAAAGCGACTATTGAAGCTAGTGTAGCCAAATTCAAAAGGCAAATTGATAGTGCGGTTAAGTCTGTGCAAAGATTTAAACGAGTAGCAGATCAAACTAAAGATGTtGAATTAAACGCTAACGaTAAAAAaTTACAAAAAaCTATCAAGGTTGCTAAAAaGTCTTTAGATGCCTTTAGCAACAAAAaTGTAAAAGCTAAATTAGATGCTAGTATACAAGACTTACAACAAAAGATATTAGAATCAAATTTtGAACTAGACAAACTTAaCTCCAAAGAAGCTAGCCCTGAGGTTAaaCTACAAAAACAAAAGTTAACTAAAGATATCGCTGAAGCAGAAGTTAAGTTATCCGAACTAGAAAAGAAGCGTATCAGTATTGACGTCAATGCAGATAACAGTAAaTTCAATCGAGTGTTAAAAGTATCTAAAGCTAGTCTTGAAGCATTAAATAGGTCTAAAGCCAAAGCTATTATAGACGTGGACAATGGTGTTGCTAACTCTAAAATAAAACGCACTAAAGAAGAGCTTAAAaGTATTCCAAACAAAaCTAGATCTCGACTAGATGTAGATACAGGGCTTTCTATACCAACTATTTATGCGTTTAAAAAATCATTAGACGCATTGCCGAACAAAAAAaCAACAAAGGTAGATGTCGATACTAATGGTTTAAAGAAAGCTTATGCCTACATAATAAAaGCAAAaCGaCAATTTCcAAAGACAGaT

>NRS408contig00009 [organism=Staphylococcus aureus]

ATAGtCGATAtGaTTtGATGTTAGTCCAtacAAaTAgaCATTAtGGTAagACacTCGTACTTAAtAtGCAAACAAtAAATTCGGTATtATcGGTACtGATGaTTTAAAaGAagAAGGCTataTCGCTCAtATTTtAGGTGTAAATGCTGAaGAaGGCGATGAAATTACTGAGTATTTAAATGAAGTCATTCATTAAGGTTCTCCACCAAATGTGGTGGGTATATAATTTAAAGAACTATTTTAAATTACAACTTTTAGAGTTTTTATTATTAGGCGGCCAGTCCATTATTGGGCTTGGTTGTCTTCTTTTTTtCTCCTTTGTATAAGCTGAAAATCATCATTATACGTGCTTTAAAGTTGTTGAAaTTTCTGTAACCAAAAGAAaTTCGCTTGATTAATTTTATCTTATTATTAATTCCTTCTATAGCACCATTATTAAATGCTGGGTAATAAATTGTATTtCTTAaCATCCTTTGATGTTTtCTATAATATTTAACCACTTtCCATACACCTTTACTCACAGACTTTTTACtAACTGAATTTAAACGATTAATAAATTTAGGCCAATTACATAACCTTAGATCTTTTCTTAATCCTTGGACAAGTTCGTAGGAGCGTCGTAGTATATCGTCTTTTGAAAGCATGAATTCTACAATGTCAGATGAGCGTTTATAAGCCTTAAAAGATTTATTCCATCTGTATTTACTAAATATGGTTTTACTAGTATCCATCAATAGAACTTTCCAGTTATTCTTAAAAaTTGTATAATTAGGtCCTTTTTtATTACGGTATTCATTCATAACTTGtACACGATACTTATTAAGTTCCCTATTTAAATGTTGAaCGATATGGAaTCTGTCAAAAaTAATAGCTGCATTAGGAAATAGGTCGCGAAATAATCGGACATAGGGTTCATACATGTCAATAGTAACCGTCTTAACTTGTTGTCGATTTTTtAAATCGAATCGCTCGAAATAGGCACGCAAGAATCTTGTAGTTCTATTTtCTAAAaTATCTATAACATCATGAGTATCATTATCTATAAAAaTGAAaCTCATTGATCCAGTTACATTTTtAACGCTTTtAAATTCATCCATAGCGATGTGTTCTGGCAAACAaTTAAaCGGTTtAaTTCGTAATGAATTAGCcGCTTTAGTtCTAaTACGACTtACAGTAgATGGAGAGatACAGTTATCTTCTGCAGTATCAATCTCTGTTTtAACTTTAGTAgCTTCTTCTATAACTTTTtGAGTAACACATCTTGAGATAAAACACGACTCATCAACAATTGACGTTTTAGCAGTAAAAGTGCCATTGCATTCTAAGCATTtAAAaCGTTGTTTaGCTAAGTTGaGGTATGTGTTATACCCTTGAGTTTTAAGTAAAGTAATACGAGAAaCacGCTtACCAT

>NRS408contig00012 [organism=Staphylococcus aureus]

AATGGTccTTTTAATAACATGAACGCACCTTTTAAAATTGTTAATCCCGCTCTTAATAAACCGAACTTACTTACTAACGCAATGATTCTACCTATTAATCCGCCACCCATAAAGTTaGATACAGCAAGAATAATCGGTATtAAAaaTCtAAaTGCACCAACTAAAGTTATAATGACACCAACTAATTGTGCTGTAGCTGGATGCGCCTCAAACAAGTTAGCTATCCAACcAGTTATTGCTACTGCAACGCGTAATACTGCACTAGCTATAGGAGCCATCGCTGTTGCGAATGCAACTAATCCTcTTGCAATGTTCCCAATTAATTGCATTATTAGTGGTCCATTAGTTTGTATATAACTGACAAAGTCTTTAAACCcTTGAGATTGTCcTACTTGTTCAGACCATtCCCTAAaCTTAGCTGTCATTTGTTCAAGAGATTGGAAAATgCCAGTTGaTGATCcACTGAATGCATtCATCAAATtGTtAATTCCAACGAAAACATTTTtAAAAaTATTACCAaTGATAGGTAaGTTtGTTTTtGTGTATTCAATAAAACGAGTTATCGAATTTtCTCCAGCTGCACTATTAGCCCAGTTAGAGAAAGATTGACCTAATCTATCCAACCAATCAGCCGACCATTGAAACAGTGGTGCTAATTGTGTGAATACATTGACTAATCCATCaCCGAAACCGCCTGCAGCACTTAATAGCTTGTTAAATACCGAAACACCAGTTGTATTCATCATGTTGAAGAACCTTGATGCTACACCGCTATTTTGAGCCCATTTAAACACACTTTGAGACGCCTCTTCCATTCCTCTTGAAATACCACTAAAAAAAGGTTGTAAGCTCTGCATTGCTGTTTTAACAGTATTTAAACCGTTTGCAAGAGTTGTGAATATAGCGGATTGATTTTGCTTTATAATATCAGTCCATGCTGACTTTACGCCATCTAAAGCTTTTTtGTATTCGTTTGTTGCTGAGCTAGCTTGTAAAGTGCCGTCACTAaGCATCTTTATAGCGCTGATaGCCATTGCGCCAAATGcTACAAAGCCAGCGCcGGCTATTGCTACCGCACcACCTAAAGCAAGTACACCgCCAGTtAACACTTTGATAgCGTTTAatAGCGCAAATacTACaGGtACtACgCTCGCtATTAcAGGTATTAAGATAC

>NRS408contig00013 [organism=Staphylococcus aureus]

gaggtaaactatgattcacgacgactagaacatctacatcgctgTTAaTGcGTAAACcACcATTTACTGCTGAACCAAATAGATATACtCCAACTATTGAACTTCCAAATAAATCTTTTACGATTTTTtAATGTTTGAATCGCTTGATTtGGTATTTTtCCGTTAATCAAATTGCTCATGATTtCACCTCGTTGATTATGTTCATATAAAGTTTATATTGATACTCAATTTACTTACCCTAGATTGGACATATACTTAAATTACTGTTCAATAAAGCTGACCGTTAGCGTTTAAGTACATCCTTTCACAATTTGTCTACAGATTAATAATTATTCTTTATTATACAGATCTCCATATAATTTTTGAATTTGGTTCTGTAATTTTTTATTTTCTTTTTCTAATTCCATTACTCTTCTTTTtAAGGTTTTAATAAGGATTTCCTCCGAACGAGAACTTTTCTTGGGTTTTGAGACTACATTTGCTGTTATTTGACGCTCACGAAGGGATTCGATTCTTTGCCTAATATCGTGTTCCTTATAAAGCCATGATTTAGAAACATTAGCTTCCTTTGCTATTGAATTAAAaTTAATAACTTTACCTTCAATCGAAAaTTTAGAAATCGCTTTGTCTACTTTTtCCCTTGTCTTTTTtGATTTCTGCTTCGCCAAACGTACAATTTCTGTTGTATTtCTAACTTGTTTATCCATTGATAATtACCCCGTCAAACTTCCAATGATTTGTTCTAAACGCTCTTTAACACGGCTATTAGTCTCTACTTGTCTTTGCCATTGTTTATCCTTAGCTATGGCTAATAACTCTTCTGTACGCTCTAACTGTTCTTCGTGCTGTGGTAAGAATTGCTTACTGGTACAGAAGTGAGTGCAATCTAAGCATGCATTCGCATGTGGACAACCACCTGCTACTACTGGCAATCTACAATAACCATTTGGAAGCACTTGTGCATTTATATTTTTCTTGAACCATTGAaGCTCTACATCATCGACTTCATTATCTTCATCTAGATCAAGCACATCTCCATTATTGGTAACCAGTTTTtCCTGAAaTTTAGTAAATTCATTTTTtAGAGTTTCaTCAAAGATATGAGCGTATCTGCTTGTCATTTCTGGGCTTTCAtGCCCCAAAAATTTcTGCacAAtATgc

>NRS408contig00015 [organism=Staphylococcus aureus]CGGACAATATGCCCTACTACCTGTAACAGATAAATTTATGTATGAATGGTAGTAGGGCATATTGTCCGTCTATACCAGAAGGACTACGtCCAAAGTtACTTATATACTCACTCTCTAACGTTTCATTATCATTCTTGATATTAATCCcTAAaTtGCCATGATATCCCGCGTCTATCTTGCCTGTTTCAATCACTAAATGTGTTTtACtACTTACACCACTACGGCTAGTTAAcAGCCCGACATAGCCcTCTGGTATGCTTACAGCTACATCTGTTTTtaTtACTGcTTTTCTTGTGGCTCgaGTACGACagTTTCagCTGAGAATATGTCATAACCTGCATCCGTCTTATGATTTCGTTCGGGCATtCTAGCATTTTCTGATAATAGCCTTACTTGTAATGTGTTAGTCATTTTCCTGCTCCTCCTTGATTAAATGAATTGGTTTAACAACAAAGTCTATAAGACTAATAATAGATCCGTCAGATAACTTGTAATGCGTGTCTCTAATATCGCCAACAAGTTGTACAATTTCTAAAGTTGAaTTTGTTtCtGGaTTAAAAACCTtGTCtCCTACACTAATGCTCATTTTCctGTtCCTCCTCATATTTATAGAcAaCTTGACctcGtCATAATCCCTACTGCTTCATCAAGTtcAaaTATCTTCTTTGAGTGCATCTTgCATaGCaTTAGgTAAaCCcTcAAGtATTTCATCAAaCGCTTGTGCTTTCTtATacACGTCcTCAATCTCTTTTAGTAATCCCtCtGTGTCATTgCCGTTAtACGCACTAGCACTTATAACtgATtGTTCaTTTGTTCgCGgTTATTCATTAGTGTCATCCTCCATAAAAaTTTtATTGTTTAATTTCATTCCAAATTTAACTCTTTCATCATCGTTGCCGAATTCGTTTATTAAATCTTTTtCAACACTCTTGCAATACCTATCCCATGCGCTCGCTTTCTTCTCCAGATCTTTGTTGCGCTCTCTTAACTTAGCTATATCTCCAAtAAGCTCATCACGTTGCTTCTtGTACTCTTCACGAtCTTTTAATGCTTTGTGAaGTTTATCTAaTAACTTGTtAAAGTTAGTACAAAGATTTTTaTATtGTtCAtcTGATAAGGTGAaCGTcAtcTCAT

>NRS408contig00016 [organism=Staphylococcus aureus]

ATTGCAAAaTTGATAGTAGAACAACTAAAGTATGCAAGGGGCTTAATGGAGAAATATTCAAAGTTAAAGACGCTAAAATTGGTGTTAATTTCTACCCTATGCATATCAATTGTCGTTCAGATTGTGCATTACTACCTAAATCTATGTGGCCGAAAAAaCcAAaCAAAAaaCGACAAaCAAAaTaCTTtGGAGGAAAagTGAAAaGCGATGATTGATTTAAAaGtAAAaGTTTTtAAaGGCAAGTTAGCATTGTATGATaGTAAATTAaGTGTTtGGAGGATATTGGTATGAGCAATACTGACAAATACCTTAGAGACATAGCAAGAGAGTTAAAAGGTATACGTAAaGAGTTACAAAAGCGAAACGAAaCAGTTATTATTGATGCAAACTTAGACAGCGTAAGGTCGGCAGTATTAGCCAATAAAGAAAAaCCGAAaTATAACGAACCACTCTTTTAATAGCTAGCACTTAATTGTGTTGGCTATTTTTTATGTCCAAAACGTGCTGATGACATAAAAaGCACGCATGGAAAAaCAGTCGACAGACTATAAATGGAGGTATATCTCATGGAAGAAAATAAaCTTAAGTTtAATTTGCAATTTTTtGCAGACCAATCAGATGATCCGGATGAACCAGGTGGAGATGGTAAAAAAAGAGATCCTGATAATAAAGAAAATGACGAAGGTACTGAAATAACTTTCACGCCAGAGCAACAAAAGAAAGTTGATGAAATACTTGAACGTCGTGTAGCCCACGAAAaGAAAAAaGCTGATGAGTATGcAAGAGAAAAAGCAGAAGAAGCTGCTAAAGAaGCTGCTAAATTAGCGAAAATGAACAAGGATCAAAAAGATGAATATGAACGCGAGCAAATGGAAAAAGAGCTGGAGCAATTACGCTCAGAAAAaCAATTAAATGAAATGCGTTCAGAAGCAaGGAAAaTGTTAAGCGAAGCGGAAGTTGATTCATCAGATGAGGTTGTTAATTTAGTtGTAACAGATACTGCTGAaCAAACTAAATTGAATGTTGAAGCTTTTTCtAATGCAGTAAAAAAaGCGGTTAAtGAAGCGGTTAAGATTAACGCTAGACAATCGCCATTGACTGGTGGAGATtCATTtAATCACTCgaCT

>NRS408contig00018 [organism=Staphylococcus aureus]

CAAAGaTTGTTTTAAAGAcTTTTTTtAaatCTtGATTGTAATTCTTTATCCATAGTTTCTTGGTCAGTAACTTTGCCGTCTTTAAAAGGTTCTGCGTGTATATCACCGTATATTTCAGCTAATGCACTTCTAGCTTCCATTACGAGCCCAGCGTGTTCGAATGTTTCTTCTCCTGAATAATTACCATATCCTCTgaTAAAagTtgCGAAATtACTTGCATCTTCCTCGAGTTTTATAGCGTTGGCGTTGACTTCGTCAGAAATAAAATAAGACGCTTTTTGATTTGCAAAAGGCGTCAATACAAaCTTATATCTGTCTTTCTTTTTGTCATACGTTATTTTATATTCTAAACCGAAATGTTCTAAtCCCTTTttAAACATTTCTAACCTTGTATCGCCTTCACCACCATTTTCAAACTTCGAAGACTTAACCTTaCCTTCGACTTCAAAAaGCATTCCAGTACCTTGAAACACAATGTTAAAATATCTTTCTACTGTAAAAGATCCTGTTACATTAACATAAATCCTATCAATCATTAACTTGTCTATAGGAATCTCTCTAGCAGTACATTCAACCAGTTGTCTGTCGCCTTCTGATTTCCTATCAATGACAGTTATTACATATTCTTTCTTGTCATTTTCACCTTCGACATGACTAACAATCCATCTTTTCCCTATAGCGTTAATAACTTCATAAGTATATTTATTTTCTAGAATATCAAAaGTTAATACACCGTCAGCATTAACTTTTTTTACTAAAGTTGTTTCTACTGGTACAGGTGCGCCATTACCTTTAGGTGGTCTTACAATTATTGTCATTCTGACACCTACTTATAATAAAATTTCAAATCAAACTGAACTTTTtGAACTGTTTGATTAAACTCAAATTTATTAGCTCCGTATTtAAATTTTGGTTGGGCTATGTTCGTTTCAGTGCTTATTTCGACACCGTTTTtATAAACTCGAAAGCTATCATAAACAATTTtGTCTCCaGCTTTtaGTTTAATCCCcTcAATTTtCATTATTtCAGCATGCgTTAAATtCCATACAAACGATtCtGtatcttcgcctaaataattgttatctttt

>NRS408contig00019 [organism=Staphylococcus aureus]

CTAAAAgTATTaTTGCAGaTAaTTACGAAgCaGATAACCCAGAGAATCAACAGTTATTTtAAAGGaGGAAAAGATATGAtGAAAAATAGTTtGCAaGCTAAAGAACTTgCGgtAATTTtatcTgTTtCTAAATCCAAAGCAGGACAAATAaTAAGaGAACTGAATAAaGAGCTTGAAGATGAAGGATACATTGCgATACGAGGCAGAAtACcAGTCCAATTAGCTAGGAAAAAaTtCCCTTATCACGACTTATCAGACCAGAGAATAATGGAGGAGTTGAAAAAAGAAAATGAGtAACATttATAAAAGCTACcTaTtAGCAGTactaTGCTTCACagTCTTagCgaTTGTACtcATGCCGTTTCTATACTTCActACaGCgTGGTCAaTTGCaGgATtcGCAAGTAtcgcAaCATTCATATTTtATAAagAaTACTTTTATGAAGAaTAAAAAAaCtGTTACTCACGGCAAtGAGTAACaGTcTAAACAATTagAAAaTTAaTGcAtATTCAATATAAAACGAAatAAAGGAagTGTCAACAaTGTACTACAAAATTGGCGATGTATGTCAAAAaGtAATtAAtGTAGACGGATtCGATTTtAAATTAgCAGTtAAGAAaCAAGATTACAGCATTCTAGTGAATGTCTTAGATTTAgAAgAtAGATTTATCGACGgTATAAATaTAACAGATgAGAATGATCTATAcACAGCATTAGACATATTAAaTCAATCtaTTTATGAAtGGATTGAaGAGAACACAGACGAAAGAGACAGgCTAATTAACTTAGTCATGagATGGTAGGTATAAGCATGAGAGATACAGAAAGAAATATATTGAATATTTTTAAGACGTTATTCGACGAATATACTTTGTCAAACCAACGAGCATTATTGGAAATTGAACGTAATCATCACGGATACTtATCGATTAATTTCTTGCACTATCACGACAGTTACAAAACgAaCAATAAGCTTGTaCAGATACATGAAATCAATCCGgACAGCCAcGAACGAATAAAAaaTTtAaTTAtCGaGgTGTtAAGAGGTCACcgAAaGATTAAAAaaGgaGCATGAT

>NRS408contig00020 [organism=Staphylococcus aureus]

AACTAAACTTACATTAAAAAATaTtAACAAaTATTTAaCATTTTAACCtAAGAaTtAAAACACTTCTTTCACAATCAaTCTCTCATGCCATATCCACTCATTATGATTGTTCCAATAAATGCGACACCAACCATCTATAATTTCAAACACATATaTTAATGTTCCAGGCGCGTATACAGCCTGTCCAACATCGAATCTATAGTTAGTACGATTATCACCGTATCTAGTGGCTGAAGTAGCACCTAAGCCGTCGATTTTCGCATTAAAATAAGCACCTTTTGACCATTTAAGGTTATAAGGCGCTTTACTTCCAACTGTTATTTtACTTGCAGATTTACCGACTGCTTTTtGAGCAGGTGGTTTAACTTtATTtGTGATCTTATTCATTAAGCCcTCACTTTTATACTTAGGTCTAATAAAGtGAGTACAaCCGTAaTAATTATCCCAACGTAaCTTTGCAGGCGTATTTGCGTTACcGTCATAGTTCTGTTCcAAAATTAAAAaTTGATTTGTATTACCaCCATTAAACACTAAaCcAATATGACCGTATTGTTTATATATTCCTTTGGTAAATACAGCCACATCACCTATTTGTGGAACAAACGATGGTGTGTTTTCATATACTGTTGCCATGTTTTtAAAATCGTTATTGATTGCATCTTTtGCATTTCCCcACATTCTAATTTCTAACAACCAATAAATGTAATCAACTGCTAAATCTGCACATTGGTAACCATACCAACCGTCAAAaTCAATATATCTACCTTGATACCAACGTAACCTTGCTCTTGCTTCACtGTATGTTTtCATTATTTtACCTCCTAGTATTTTCTTCTTGGTTCTTCATATTCTAAAGCTTGGTGGCTATCACCTATACCTTTAGTAGTCGGGTCTTGAATCACACCAGTTAATACTAAAAaTCCTAaTATAGCGTTTAAACCGTCTGTTAATtGCTCTGTAtAAACTtGgaTATCAtACCCAATAGCTTTtgCGATGTTTTgAGCAAatAAAaaGAT

>NRS408contig00021 [organism=Staphylococcus aureus]

ACGGCAACAAAATGCAGTTACAACGTAGCTAtCACGGTAATGTAATGATGTtAAAACGTGATGCaGGtGCTCAAGaTGTTGTTGCTcAAGTGATTTATAAAGGCGATACATTCAAGCAAGAAATGGgAgAAaCAGGACGTAtCAAAGCGATTAAACACGAACAAGACTTCTTTAACATCGACAAAGAAAACATTATCGGTGCGTACTGCACAATCGTATTTAATGATGGACGAGATAACTATATTGAAGTCATGACTATTGAACAAATTAAACAAGCATGGATGCAGTCATCAATGATTAAaGATGAAAAAGCATTACAAAATTCTAAAaCACATAATAATTTCAAAGAAGAAATGGCTAAAAAAaCAGTTATCAATAGAGCTGCTAAACGTTATATCAACACATCAACAGATAGCAATaTTTTCAAATAcGCACAAGAATCCGAACAACGTCAACGCAAAGAAGTGTTGgACGCAGAAGTTGAAGAAAATGCAAATCAAGAACAATTGGACTTTGAACAACCAGTTCTtGAAGAAGCACAATACACAGAATTAGAAAATGATAAGCCTATTGATGTATCTGACTTTGAAGAAATAAAAGAACCTGCAACAGAAAAaGAAAGCGAAGAAGAGCCATTTTAATTGAAACAATAGCAACTGGTTCAAGtGGTAACTGCTACGTCTTAAATGATGGACGTACTACGTTACTgCTTGAGGCAGGAaTAAAATTTGAACGTGTTCAAAAGCAtttcAAATATAAAACAAGACATATAGCAGGGTGTCTTATCACACACGAACATGGTGATCATGCAAAGTACACAAAGCAGTTTGTCGACAATGGTGTAATCAGCTATATGACTGCTGGAACACAACAAGCtATGAATTTTGAAAGTCATCGCTTATGCaCGATTAAGGCAAAGCAAGaGCTgCGAATAGGCACaTGGtCAaTTCtACCGTTTGACATCGAACATGATGCtAAC

>NRS408contig00022 [organism=Staphylococcus aureus]

aTGAaCAAATGCAagAaaaCATAAAAaaCGGCAGTGTaCACAGCgCATTAGCAAACAGAATTATGGAGTCTCATTTTAGCTTAGAACATGCTATCGGAATGTTAAAAGCAAATGATTTAACTAGACTCGAAGAAATACATTTAATTCATTTAAGTAGTCAAAATTCAAATGCAAAATACATTAAAAGTGAAATACAAAAaGTGACGGGCGCGCCCGTTTATGTTGGAGGTTTATAAATGCTAAACaGAACAATATTAGTTGGTCGTTTAACTAGAGACCCAGAATTAAGAACCACTCAAAGTGGTGTAAATGTAGCATCATTCACATTAGCAGTTAACCGCACATTTACGAATGCACAAGGAgAGCGCGAGGCAGACTTTATtAATATCATCGTATTTAAAAAaCAAGCAGAGAACGTTAATAAATACCTATCTAAAGGATCGTTGGCGGGCGTAGATGGTAGGTTaCAAACGCGGAACTATGAAAATAAGGAAGGTCAACGTGTATACGTTACGGAAGTTGTTGCCGATAGTATTCAATTTTTAGAACCGAaGAACTCAAATGACACTCAACAAGATTTATAtcAACAACAAGTACAACAAACACGTGGACAATCGCAATATTCAAATAACAAACCAGTAAAAGATAATCCGTTTGCGAATGCAAATGgtCCGATTGAacTAaatGATGATGATTTACCATTCTgaTTTAACCGGTTTGAAAGTGAGGTGTGTATATGACTGGTTGGATAAAACTtCATAGAAAACTATTAGATTCGCCTATTTTtCAGAACGAAAAGTTATTCAAAGTATTTGCATATtGTcTTATGAAAGCTAGTCATAAGGATCATACACAGCTtGTTGgCaGGCGGGTTGTCGAATtAGAAAAAGGTCAATTTGTGTTCGGgAGAAaGCGAGCAAGCGAAGAGTtACGTCTCAAagAA

>NRS408contig00023 [organism=Staphylococcus aureus]

GtATATGTTGTAATgCATTTCtATAaTGaCGgTATGTATTTtCTTtAACAACAGGTTgTTTATAtGTTTTAATCCAATTTtCGAAGTATTCTTCAAGAGTTATATAGTTATCTATATTAAAACCACTTCTTAACtCATTTAACTTGTCTAGTCCAGCAGAATTAGCTtCACGCTTTGTtcTAAAACCTTtCTTACGGTATCTTTTCCTtCATACTTAAaTtCATaTTGCCATTTTTtACCATCGTAACAACGTGTTTTCATGCGTtCCCTCCTCAAAATTgaCAAAAaaTAATAAGGGTAGGCGGGCTACCCGTGaTTTAtaaaaaaagagagagcgcagatgcaccctctcatgTCGCAAATATTTCAGCGACTTGTCTAATTTGAaGCTTGCCGCAAATATTTCAGCGGCTTGTTTTGTATATATGTAATATACCATCAAAGAGAGTGTAGTTCAAGCGATTTAACTAaGAAATCTAATTTTTATACTATTTTCAATTTTATCTACTGTTTCTTTTGAaTATGAtATTtCTCCGGCAGGGTCATACCTATTAATTTtCGATATTCTATCCTTGCTGATTGTAgTGATATTTAAAaCGTTAGCaTAGGTCTTTTTATACTTGAaTCGCTCATATCTTTTgCgAaCcTTCGAATATTTTTtGAAgTCGTCATTCAGCgATTTGTTTtCATCAAGTAaTTTTTGATCGTaTGGGTTTTCTGCTTTTGACACCTTTTCAAGaTTGTtcATGATTTTTttAGCTAAATCCTTACCCGTTACGTCcATTTTTTCCAATACTAAAGgTAACAAATCTTCTTCGATAtGCACATTGAATTTACTTCTGGAAGATGTAAGTGGAACTACC

>NRS408contig00025 [organism=Staphylococcus aureus]

TCAGATGAAATAGTTACCGCATCACTTAAAATCGCTAAAGACAGAAACAAGGTTACTTGGGgATATGCTAAAAGCATTTTGAATACATGGCTTAATGCAAACTTGAAATCTATTGAACAAGTACGTGCATTTGAAAAGCAACAACTTGAAAGCAAAAAaCAAAATTATAAACCTTtCGTTAAACAATCAAAAGAAAAAaCaCCCAAATGGCTCACAGACAGCACGAGAGAAaCGAAAaCGCCGGAAGTAGATGAAAaCCTTGAGAAAGACAGAGAAGCTTTTATTAAGCGTCTAAATAGCAAATGGGAGTGATTGAAAATGGATGCATTTGATAAATACTATCTATTTGATCATGACGGCAACAAAATGTTTTCAGTTACACCACATTTTAAAGATGGTCGGCATTTAGTTGTTGGAaTAAAAGAAaCAAAATTTAATGGTCGTCGTTGGTATTTAGACGATTATGAATTAAATACACTTATTGATAATGAACAAATGGAGTTAGGACACCAAACAAGCTTATTTGAATATATATGAGGGATTACATGGAGATAGAAATTAAATTTAATGAAGTGTTTAATGCGCCGATGGGGTCGCCTCGTCCACGCTTTCGTAAaACAGGTAGATTTGTTCAAACTTACATGCCAACGTCTTACACAAAGCATAAAGCGTATATACAAGGGCAAATGCCTAAGTTAAATCTAGAGCGCGCACTAAAAATCGAATTAGACTTTTACTTTCCATTGCTTAAATCgTGGTCGAAGAAAAaGAAAAGCGAAATGGTTGGGCAGTATAAAGTGACTAAGCCGGATATCGACAACTTAATTAAAaCGGtATtAGATG

>NRS408contig00027 [organism=Staphylococcus aureus]

acatgaaaatgaattgataaaaaaGaatggtttaactccaggaatggttgcaaaaagaGTACGAGGTGGCTGGGCGTTGTTAGAAGCCTTACATGCACCTTATGGTATGCGCTTAGCTGaGTATAAaGAAaTTGTGTTATCCAAAATCATGgAGCGaGAGAGCAAaGAACGTAAATTGGAAAGACAGCgAAAGAAaGAaGCTGAGCTAAGAaGAAAGAAGCCACATttGTTTAATGTACCTCagaaaCATTCACGTGATCCGTACTGGTTtGATaaTACTTATAACCAAATGTTCAAGAAATGGAGTGAAGCATAATGAGcaTAATCAGTAACAGAAAAGTAGATATGAAcaaataCAAGACAAcGTTAAGCAACCtGCGCATtACACATACGGCGACATTGAAATTatAGATTTtATtGAACaGgTTACGGCgCAGTAtCCACCACAATTAGCATTtGCAATAGGTAATGCAAtcAAATAtCtaTCTAGAGCACCGTTgAAAacGGaCAtGAGGAtAgCAAAGgCGAAGTTTtATGTCcAAaGAGCTTTtGAcTTgTGGGAGcAATgACtATGACAgATAaCGCaCGtAAAGAATACTTAAaCCAATTTTtCGGTtCTAAGAGATATCTGTATCAgAaTAACGagCGAGTGGCACATATCCATGTATtAAaTgaCACTTATTACTTTCACGGGCATATCGTaCCAGGTTGGCAaGgCGtgAAAAaGACATTTGATACaGCGgAAGAGCTtGAAAaATATATAAAGCAACATGGTTTGGAATAcGAGGAACAGAAGCAACTAACTTtATTTtaaGGAGttGGAAATGATG

>NRS408contig00028 [organism=Staphylococcus aureus]

GTCACCAGAaTAACTATCAAAcGTGTATTTACTtCtGTTTGaCTCCGGCATTTTTATTACAAAAAAAaGGATACGGAATCTCTTGTTGCATCTCTTTACGAGAAATAACAGGGAATCCATATCCTTGTAGCATTTCATACGCTTTaTTATAAAGTTGTAaGTTCGGTGTCATGCTTTTATCTCCTATtCAAACAACgCTTTCAATTCTTCTACAGTTGATTTTCTTATTACCTCATATACTGGCCACATAAAAGGTTCTGCCTCCATGTATCGAGTACCAAACTCTAAGAAACCACTATAAGCTGCATGCGATGTGATAGTGTATTGCAAATCGCCAGTTTTTTtATATCTGATATTGCGTGATAAATTACCAGTCCAATAACCCTTATTCATTACTTCTCTAGCTTTCAATTTAGCTCGTACTACATATTCTTTGGCGTTTtCCTGTAAAaTaTcATCAACATCATCATCAATGTTGGTTTtcAtATCGTGAAATTGGTTTAACAGTGCGTCTAAtCcATCTaTATtCATCAATTGACCTCTTCGATATAaTaTGACGTTTCGTGTCTGTATATCCTTGTATCAACTATCTTGTAGCGAATGCCATTAaCCAACACGTGGCTAACAGGGTAAGATATTGATtCTTTtATCCTCAGAACACTTACATCGTTTTTTACATCACCAAATTCAAGTTGCTTTCTTGCTCTAGAAATGGGGTTAATATTGCaTGgtAtcGCAtcAtAAGtGATTagTGTGTTtttCTTTTTtgCtAGTTTTAGGaTtGtAAgTtGCTACTTGTTCTaattgaaaaataactctatcttcatatctca

>NRS408contig00029 [organism=Staphylococcus aureus]

ACaCTCTTTCGAACGTATGCCTaTTACAGAaTTTagCAacAaCGAAAGAaGAAAaGGGGATTATGAGAAaGTAAtCaCTTTAATTgATTTGTATGATAATGCTGAATCAGATACTGCTAACTATATGAGTGATTTAAATGACGCTATGTTACTtATTAAAGGTAATTtAAATTTAGATCCTGTAGAAGTTAGAAAACAAAAGGAAGCTAACGTGTTATTTTTAGAGCCAACCGTTTATGAGAATAGGGATACAGGTATCGAAACAGAAGGTTCAGTTGACGGCGGTTATATTTATAAACAATACGATGTACAAGGTACCGAAGCTTATAAAGACCGTTTGAACAGTGATATACACATGTTTACCAACACGCCTAACATGAAAGATGATAACTTTAGTGGCACTCAATCGGGCGAGGCAATGAAATACAAATTATTCGGATTAGAACAACGTACTAAAaCTAAAGAaGGATTGTTCACTAAAGGGTTAaGACGTCGTGCTAAGTTGTTAGAGACAaTaCTTAAAAaTACaCGGTCGATTGACGCTAaCAAAGATTTCAATACtGTTAGATACGtATACAACAGAAACTTACCTAAATCATTAATCGAAGAaTTAAAAGCTTATATTGATTCTGgCGGGAAGATTAGTCAAACAACTTTAATGTCTCTATTCTCGTTCTTCCAAGACCCTGAATTGGAAGTCAAGAAAATAGAAGAAGATGAGAAAGAATCTaTTAAAAAAGCTCAAAAaGGTATTTATAAAGACCCTAGAGACATCAATGATGACGAaCAAGATGATGATACAAaGATACTGTTgataaaaggaat

>NRS408contig00031 [organism=Staphylococcus aureus]

AGAAaTCATTATCGGCAATTTGGAATGCAACAAAAaGTATTTTtGGTTtCTTATTCAATAGTGTTAAATCTATTTTCACTAATATGAAAAaCTGGTTATCTAGTACGTGgAATAATATCAAAAGCAATACCGTCGGCAAGGCTCATTCGTTATTTACGGGTGTAAGGTCTAAATTCACAAGTTTATGGAATgCgACGAAAGATATATTTATTAAATTAAGAAATTGGATGTCAAACATCTGGAACTCTATTAAAGATAACACTGTAGGTATAGCTGGTCGCTTATGGGaTAGAGTGCGTAACaTCTTTGGAAGCATGCGTgACGGTTTAAAaTcTATcATTGGtAAAATTAAaGATCaTATCGGTGGTATGGTAGACGCTGTTAAAAGAGGTCTTAATAAATTAATTGAaGGTTTAAACTGGGTCGGTGGTAAGTTGGGTATGGaCAAAATACCGAAGTtACACACTGGTACTGAACATACGCATACTAcTACAAGaTTAGTTAAGAACGGTAAGATTGCGCGGGATACGTTCGCTACGGTTGGGgATAAAGGACGTGGAAATGGTCCGAATGGTTTCAGAAATGAAATGATTGAATTCCCTAATGGCAAACGGGTACTTACGCCTAATACAGATACGACAGCGTACTTACCTAAAGGTTCAAAaGtAtATAACGGCGCACAAACTtATTCAAtGTtAAATGGAACGCTtCCAAGATTTAGCATAGGtACTATGTGgAAaGATaTTAAATCCGgTGCatCATCGGcATTtAACtGGaCAAAaGAtCAAaTAGGTAAAGGtACCAAATGGC

>NRS408contig00032 [organism=Staphylococcus aureus]

CCTTTAGCAACTtCGTtAGCaCTTTTACtGgTTTTAGCTCCTAaGTCAaCtGcTTGATTAGACATGCTTTTCAAGTCTTTACTGCTTGCTTGCGCAATCGCTCCAACTCGAGACATTTGGCCTTCAAAGTCTGCACTTGTTTTtAATGCTGCACCTAaCCcTAAAGTAATTGGTgTAGATACGCCCATCGTCaTTGTACGTCCCAGGGAAgTcATTTTGTCTCCAATAGAaCTAAATTTCTTtGAcATGACATCCGCTTGACTTGCAaGTttACCgAAATGACTTtGaGCTaTCaTtGTtCTTTGTTAAAAGTCTTCATTTCGGATGAAGCTTTATCTATTGAACGcTCCAAaTTATTTAAAGCaGCTTTTTCTTTATTAACAGCTGTTTCAGCTTTTGCGACATTAGCGCtATGATtCTTAATAGTATTGTTTAAATCATTAAATTCTTTTTCTGTTTGCTTTAATTTAGTATTAGTTTTAGCGTAAGAACTTTCAATTTTATCATTTGATTTTGAAAGATTGTCATTTTGCACTTTTAGTTTTTGAaCTTGATTGCCTTCTTGTTTaTATTGTTCAaCAaGTGCTTtATGCTtAGCGGACTGCTtCTGTACTGCGTCACTTGCtCTTTTtAGTTGTGCAGTAGTAGCttGGTTaCTATtCTTAAGCTTTTGTTCTGCaTCTCTCAACtGTTTAAGTTTTtGATACGCAtCTTgTTTACGTTGAtttgtacgtttatattgattttcagcttttttaagttctGTATTCGAT

>NRS408contig00033 [organism=Staphylococcus aureus]

CTTTGATTAGAtGGCTCtACTaCAACaTTTAAATcGTATGTGTCGCCAACTTtAAGTGTTTtAATGCTATCTGGTATATTAACCGACTTTACCGCAGTTTCCGATGAAGCCGGTTTTGTTACAAAGTTTCTTCGTTACCCTCTGTCACGTTTCCAGTATATTCTTCGCCTAAAATTTTCTTTAAGAAaGCCTCTTCGCCTTTTTCACCGTCTCCATCATGATTTGTCATGTTAGCTGAATCAAAGATATACTTACGTACTGACTTTTTATTATCAACTAAAGGGAAAaGTGCCTCACCTTCAACCTCTTCACTTGAGAAATCCCAATTTTTCTCAGCCGTTTCTCCATCGATTTTAGGATTTGTAAACATAACTTtAGGTAATAAAACTGTTCTAAATGTACCGTcTTTACGCTCTTGTCTGAACCATACAGCTACGTAATTGTTTtGTTtACCTtGTTtCTCTTcGTAAaCGCCATCTTCATCATAaTCTTCATTAAAAACAATTTTGCGAATCTCTTTAGGgAACGCATGCATTTGTAATGAGATTTTACCTTCTCCGTCTGTATTCCCTGATTCAATTGGACCGCCATCAGCATAAGCTGTTTTtAGTTcTCcACCAGTTtCAAcACCtAATTTTttGTAATCCTCTTgTTTTtGTAATATCACTAtATTttAATTCCGCGCCTTCTTTCGTTAATTTAGCGAAACCTAAACCAGTAATGTTAATATACGCCTTtGGCGCACTTGCATGTTTTACTGCCATt

>NRS408contig00034 [organism=Staphylococcus aureus]AAGACGTTTTCAGGTTGTATGATTAATTCACTGTACCCAGAATCAACATTGAAATAATTACTTCCAAACGaTTTCTCGCTCAACaTTGGTTCCTCATTGATGACAAcACTTTTTGCTTGCATATCTATTTTCACTAAATCACCTTTTTGtaTAATGACATCCCTtGCGCCTTtCGGTTTCGGTAGAATCTCCGTATTGAATGAaCCTAaTCCATTCATCTCCATCCACTTATAACCGTTATACTTCGCACTATAGATAGCTATGATAGAagCTGGAcGCTGATAAAACTTACCGCCaTCTATCcActCTTTCtCATCCATATCAATAGgTTTaCGTCTATCTGGGTCTTTAATGTGATCAAATTtCCAAGTTTtAATAGAAAATTTATTACCTACTCTTCTAAGCCGCATATAAACAaCGATTCTGtCCAAGTTATACATTATCGGTTTATTCTGATAGTCGTATATCTTTTTGGGGTCTCCTTTTTGGTTATACAACGTAACAACAATATGTCCTATTTTtCTATCATGATATTTATTTTCATAACCAATAGAAGCAAGTAACTTACCATCACTATCATAAATATGTTGTGCTGTTCTTCCGGCACCTTTACCTTTTTGTTCAACAATACATTTATAGGTAATTtGAAAATCTGTCATCGCTTTAGGGAGCCcTcGTTTCGTGCCAGcACCAACCcAACCTTTTGCAtCAGGAAAaTT

>NRS408contig00035 [organism=Staphylococcus aureus]

GGTGTTTCGACGAATATGTCACCGAAAaCAAGATGACCTCTAGCTACCGGACTCGTTAGAGCATTGCCTATAACTGATCTGTGCCAGTTTTtACCTAAGGGTGCTTTGTATTTAGAGTTGTTCAATTTTATAGTTATTTCTCTTAAACTAGTACCTTTTTtCGCTTCTTCTACTGCAAATCGTAaTACTTTTTtATATTCATTAGGCACAAATTTATCGTTTACTCTGTCGTAATAGAAAGGAGGGACAGTTTTAGCTAACCcTTTtCTAGCTGATGCGCGTCGACCCATTGCAGTACGCTCTTGAATTGTAGTACGCTCCCACTCTGCCATAGCACCTACTAATGTTACGAACAAACGTCCCATAGCAGAAGTTGTGTCATATACTTCTGTTGCGCTCCTAAACAACACGTTTTTATTCTCAAACAATTCTAGTATCTCTAGTAAGTCTTtAACACTTCGAGTTAATCGATCTAGTTTATAGACtAAAACcAAATCAAAATTATCTATTTCATTCAACATTTCTTGTAAAGCGGGTCtGTCTTTTTTAGCTCCGGAGTATCCAGCGTCAGTATATACTTtAtGAATTTTCCAGTCgTTtATGTcGCTGTAAgCtCTtAATTTtCTTtCTtGTTCTTCGATAGaGTgTCCTTTttCTTTttgTtCAAGTGTACtCActctagtataaattgctactttcatgtgct

>NRS408contig00036 [organism=Staphylococcus aureus]

gaCATAGAATTAACAAAAAaGATGGTACTGTAATCAAaTTAAGTGAATACGGGTTtATCGTTAACGATATAGTAATTGATAGCATGCAAATCAACACAAAGTATCAAGACAAAGAAAAtATGAACGGTCGTATATTAATGGGgAGCAATTATAtCAGTAGAGATATAGTTGTTCCTTGTTTTtGTAAAGTTAAAAaTCGTTCAGACATTGCTTATATGCGAGATATGTTGTATAGGTTAACGACAGACATAGAACCTATGTATTTACGAGAAaTAAGAAGAAAAGAAGAGTTGAATTACAGGTTTACTCAACCAACTTCTGATGATTACGTGAAATTAGATAAAAACAACTTCCCGGATTATGAaTATTCAAGACACGATCAACAAATTTATGTAAATGGTAAACAGTATAAAGTTATTTTtAACGGAGTTATAAaCCcTAAaCAAAAaGATAATAAAGTTTCTTTTGAACTAAAATTCGAAACTACAGAATTACCATACGGCGAAAGTATTGgAACAAGCCTAGAGTTAgAAGAAAACAAAAAGGTTGgATtGTGGTCGTTTGaTTTTAaTATTGaTTGGCATGCAGGCGGAGACAAAaGAAaGTATACATTtGAAAaTTTgAGCAAAGGtACaGTTtACTATCatggtagttgctcctaacgaccaattcaacatgta

>NRS408contig00037 [organism=Staphylococcus aureus]

gTCtAAtCCTAGAGAtCtATtGTGtGActCAAcaTCATtCAAAtCATTGAAtGCCTCAATAGCTTCtAATaCATCTTTgTCATCATCTTGATATTGAATTGGATTACCCAaGAAaTAGCCGTTGATAAAATCGCTAATATAaGATGCGTAATCatGCGCTACACGGTTATCTGCCATGTACTCTtCTTtGCGTCGTGTTAACTCAACTAAGTTCTTAGTTTTACCTTCGTAATAATCACTTAACACTTTCAATCTAGGTCGTTGGTAATCCATGTGATGTTCAATGTATTTaCTTaCTtCATTAAcGTTTTGTAATAAATCGgATtCcGTCCCGTCATATGTGTAAACAaCATTGgCTTCATCaTTAAAtAAGTAATTTaTGTTTCccCGTagATCtGTATctGTTTCAAATTCGTTTacTTTtAACATTTGTTCCCTCCTATAATCCTAGAGATTTtaTTGTGTCAACTTTCGAaCtGACATTTGTGCGTTTTCTAACCGGTCTGTAGAATCGTTCCACTGAATAACGCAACGAATCGATaCAATGATTGTATGtATCTACTGGTTCATTGGTATATtCACCTGTaTCTTTGtCCTTTtGCCAtGTGTAGTTgTCAAACTCTTcAATAGTCTTGAAaCAACGTtCaTCAaCAaTgATttCAAATTGCATt

>NRS408contig00038 [organism=Staphylococcus aureus]

TGGTTCGGTAaaTGTAAaTAAGTTTtAGTCAGTGCTTCGGTACTGACTTTTtATTtATTGTTGTAATTATGGTAATATGCAGAAGTGAGCAAGTTGGATAGAtGGTGGCTATctGagtatAaGgaGgtGgtgCctAtGgtGgcaTtactgAaatcTttagAaaGgagacgCctAatgaTtacAaTtagtaCcatgTtgcagTttGgTttaTtCcTtATtGCaTtGaTAGgTCTAGTAaTCAaGCTtATtGAaTtAagcAatAaaaatAaCcatcgctAacTttggctggTttcgatGgttaaatGgTtaTtaatttaatctttaatctaaaatagccaccgtcttttaacgggctcattagggtaacatgtttgcgcatgttgccctttctatatataaattaacacaccataatataaatatcaaatagacggcttattatttagattaaagattaaattaataaccatttaaccatcgaaaccagccaaagttagcgatggttattttttattgcttaattcaataagcttgattactagacctatcaatgcaataaggaataaaccaaactgcaacatggtactaattgtaatcattaggcgtctcctttctaaagatttcagtaatgccaccataggcaccacctccttatactcaGATAGCC

>NRS408contig00039 [organism=Staphylococcus aureus]

tGCaGTCTTTTGTCTTGCTGTGCCTTCGAaTTTGTTtAGGTGCTTGCGCATAtCTTTAACATATtCATTTGGATgTCGACCTcTAATAACCAcATTAgCAATTATTTCTtCTACTTCTTGTTtCATCGCTTCAGTATTAGTCCATAATCGCTCTGACCAAACGACACCATGAAATTGTGTATCAACGATTGTATCTATAACTTCTTTAGCTACTTGTACACCTTCACCTAAAaTACCCGCTTGATCACTGAACaCACGATAAGCTGTTGATTCGAAATATTCCCTCATCGATAATTCTGTTTGAGCTGTTGCATAAGCAATTAAGAATTCTATTTGAaTCTTTAACATCTGTTCTCTAGATACATACATCTTAGTGTTATACTTCTTTAATTCTTCATTTGCTCTATCGCTAAAGTCCTTGTTTTCGACCAATCTTTTTGCTTCTTCTTGAAACGCTTTTaCATCGAACTCATCAATAATCTTTTgtGCTTCTTGTAATGTAACGCCTGCAAAATCTCCGTACTTAACAATAAACGCATTGATTTCtttttcaatgcgcttaatcatcatattcaatatacgttctatttcttcagctttagttttatcacgcttcaactcattctcgattgctttgcgtccgcgttcttcccaatattcttGA

>NRS408contig00044 [organism=Staphylococcus aureus]

ATTTCCAGCATATACTTTTGtATATTCTTCGCCTTCGTAAATAAGTTTCTTTACaTTTTTAAAaTtaCCTTCCAtAAAAATCACCCCTTAATTAAGTAAAGTGTATTAGGGTCTTTTtGATATATATAGTTATATTCATTTTCTGTTCCTGTCCAAATTTTAACCGtCGGTtGAGATGCGCTTTTTAGTTGATATAAATTATCCGCTTGTTGTTTAGTAAAaGCTTGAGATGACAAAACATACCGCTCGTCATGATTATGATTTTTtGGAGCGTATAAATCATTTAGTGTTtGTTTGAaTtCCTCAAAAtCTTCTGCACTAACTTTTGAGCCAATcTGTTGCAaTACaCTTtCTGAAATAGAGTTGTTTTGTATTGCTTCTGCTAaTTCTcTTAATGTGTTCATAGATtCAGGCGCGCTaTCAacTAGTtCAGCAATTTTTGAATCcGTATACGTTTTAGAGTCgTTGAgAgTTgTATCTTGgATTTTtttAACTtCTTGCAATTTATCTTCTAACCCTTCAACATTtgCGATATTGATTTTATCCAaTAAcTCAGGTTCTGCTTTGATATCTGTATCTTtACCGTCAATTTGCCACATTTtAGTGtCAGGATTGAT

>NRS408contig00045 [organism=Staphylococcus aureus]

ATtcACGCTGTCTAATGCAGATAATGCTTATTCGAGTGACGGTTATATTATGTTTGGGTCTGATGAGAaCTATGATTACGGTGCGGGTaTCAGGTTTTCTAAaGAAaGAAaTAAAGGTCTTGTTCAAATTGTTAAtGGACGATaTGCAAcaGgTGGAgATACAACAATCGAaGCAGGGTATGGCAAATTtAaTaTGCTGAAACGACGTGATGGTAATAGGTATATTCATATACAGAGTACAGACCTACTGTCTgTAGGTTCAGATGATGCAGGAGATAGGATAGCTTCTAACTCAATTTATAGACGTACTTATTCGgCCGCAGCTAATTTGCATATtACTtCTGCTGGCaCAaTTGGGCgTTCGACaTCAGCgCGTAAAtACAAGTTAtCTATCGAAAATCAATATAACGATAGAGATgAACAaCTGgAACATTCAAAAGCtATtCTTAAcTtACCTATtaGAACgTGgTTtGATAAAGCTGAGTCTGAAATTTtAGCTAGAGAGCTGAGAGAAGaTAGAAAaTtATcGgAAgACACCTATAAACTTGAtAGATACGTAGGTTtGATtGCTGAaGAGGTGGAGAATTTAGGATTAAagAGTTTGTC

>NRS408contig00046 [organism=Staphylococcus aureus]

TAgTTGTAGTgATAGCGTTCcAAACCTTAGTTGCCACAGTAACTATAGTGTTCCACAACGTTTGTAAGAACGTCCAAATAGCGTTCCAAATTGTCATTGCGATAGTCATAATTGTGGTAAATACTGTAGTTATTACAGTGACTAACAAATTCCAAATCGTAGTAGCGATTGTAATTATCGTGTTCCAGATTGTACTTAAGAATGTCCAAATAGCTGTCCATATCGTCATAACTATTGTCATTATCGTCGTGAAAACAGTTGTGATGATTGtAACTAAAAGGTTCCATACCGTTGTTGCAATAGCGATAATTCCATTCCATAACCCTTGTAAATAAGCGGCTATTtGATTCCAAACAATCATTaTAAAATtGTATACATTAGTTACTGCTGTAGTGATAGCTTTTAAAATAGCATtCCATACAACCGAAGCTACAGTTTTCAACACATTCCAAACTGTAACCATAAACGTTTTTATCGCATTCCAAGCATTTATAaTAAAGTttCTGAATCCTtCATTTttATTCCACAATAAAaCGAATATAGCTATTAATGCAGCGATTACACCGAtaActattgttAttggaccacctaaaataccaaacaCAGT

>NRS408contig00047 [organism=Staphylococcus aureus]

ATTCCTCCTTTtAAGATGTTTaTGATCCTTTCTGCTATACTCCTGTtATGGAGGTGATAGGATGAAACTTAATCACGATTGCGTTAGACTCTTGCTCTTAGAAATAGAATCTAATAAGAAAaTAGGTGAACCACTTACTCGACATAATTTCAACGATAATATtATTTTtGATAAATATGATTTTGAAaCAGTAATGTACtcaCTTTtAAAaTTAgAagAaGCTAAATTtaTTtGTTgCGaTCTGAAATTCATCGAAGGCAGGGTCGTTtCTTGGaTTATTGATGACATCACTTGGTCTGGCCATGAATTTCTCGATAATATTAGAGACAATAAAACTTGGAACGAaGTTAAAAGaGTCGTTAACAAAACATCCAGTATGTCTCTTAATCTTATGGGGAAATTAGCTTTTCAATATCTTTCTCAAAAATTCAATCTAACTTAAATTCATAACCATCAACcAAGGCATATAAGTTATTATTTACGTATGGTATTTCTTCAATGGTGTTGTtGATGAAATGAGATCGGACcATCAGTTCATAtCCGTCATTAATTTGAATATCTAATGGTCGCCTATTACCTTCTTCGTCatAGtAGTAATAGATG

>NRS408contig00048 [organism=Staphylococcus aureus]

TTATTAACTGcTGTTTCTAAAGGTGCGTTCtCTTCCATCCCTCATCcTCCTCGCGCCACATAGGcgTtATTAATcACAATACAACTTTGCCCATTACTTTAATATTACTAAACGAAGCGACTTTGATATCATCATACTTCGGATTTAGAGATACCAAATTAATATAGTCTTCGCATATATCTACACGCTTGATAAGACTTACTCCATCTAATACAACGAGTGCAATTGTACCATCTTTAATAGAATCTTCTTTCTTAATAAAAGCGTATGTTCCTTGTTTTAACATAGGTTCCATTGAATCACCATTAACTAAAaTACAAAAaTCAGCATTTGATGGCGTTTCGTCTTCTTTAAAAAaTACTTCTtCATGCAATATGTCATCATATAATtCTTCTCcTATGCcAGCACcAGTTGCACCACATGCAATATaCGATACTAGTTTAGACTCTTTATATtCATCTATAGAAGTGACTTTATtCTGTTCatctAATTGctCATTTGCgTAGTtAAGTACGTTTtcTTGGCGGGGAGgTGTGAGTTTGTTGTATATGGAAGTGaTGtCGttatc

>NRS408contig00051 [organism=Staphylococcus aureus]

TTGTGGAAACCTTGGAAATATTTCGCTTTAAATTTAGTGGCATCTCCATTTTTGCCTGGTATTCTACTTTCAACTTCCCAAGCCTCATACAATACGCGATCTACAACTGCATCTTCAATTTCATCTGCAAAATCGTCACCATAAAACATTTTAGCAGTACCAGACATTGTTGACTCAACAGAACCACCAGTGTTATAAGAACCGTCCATTGTATCCTCTGTATCTGTATCAGCTTCATGTGATAAGCCGTATTCAGTTAAAAAAaGCATTTTAGTAGCATCTACTTTTTCGCCAGCTTTTCTAAATAAAATAATACGATCATTACTATTTTtCATATTTGCCATTCAATATTCCTCCGTTTTTTAAAATGTTTTGTAAGATATCGTTACTGATGTGtGTATCAATTCTTGATTGGTAGTATCATCAACTAACTGTGTGATGTTAGTATCATCTTCTTCAAAGTCATAATCGTTTGTTTtAACGCTAGGTGTTAAATCATCAATACATCTTTTAACAAGTCCGTcATGATGTCCTAAATCATCaC

>NRS408contig00053 [organism=Staphylococcus aureus]

ATTTgTCGTTCCTCCTTTAAGTTGTTTTGTtATATAATTtAGTTATCTCCCAGTGGAAGGAGGTGAAaTTTATGGATTTAGAGAAAaTTGCtCACGaTATTACAaTCTCGCtATtACCTAGAGCTCTAGATAGACATAAGATTCaTAACGAaTGGCAAGAAGTCGGTGATGACGTAaTTGCATTCGCTAAaGaTAGCATTGCTCGTGACTATTTCAGCATTTACTCTTCTGTGTTaTTGGGATTACAAGAAGAAGAAAAAaGCAGAAAAGATTTAGGATTGTAAGGCAATAGCGCACTTGATTACTTGCACTAATTAAGTGCGCTTATTtAATTAGATATTTCTTACCTTCTCTATCCGAGACCACTTTATATTTTTTTAATTTGCTTTCTTtCACTTTTAACCATTGATTTCCATGCCACACGTCAATTAAGTTTTCGTGTTTTTTATTGAATAGCCTTCTTAGTAGTTTCATTTGTAGTTCCTCCTTCATTCGAAATCATCGATaGTTAATTCTGAAACTCTCTTTTC

>NRS408contig00054 [organism=Staphylococcus aureus]

cTCGgCAATGAATGTTAAaCGAAtATGGGAAAGTAACACGCAGTGCTACCAAATGCTTAATTTAGGCAAGTATCAAGGCGTTTCAGTTAGTTCGCTTAATAAGATACTTAAAGGTAAGGGgACATTGAATAATCAAGGTAAAGCGTTCGCAGAAGCTTGTAAAAAGCACAACATTAATGAAATTTATTTAATCGCGCATGCTTtCTTAGAAAGTGGATATGGAACAAGTAACTTCGCTAACGgAAAAGATGGAGTATACAACtACTTCGgcATTGGCGCTTACGaCAACAATCcTAACTACGCAATGACGTTTGCTAGGAaTAAAGGTTGGACATCTCCAGCAAAAGCAATCATGGGCGGTGCTAGCTTCGTAAGAAaGGATTACATcAACAAAGGGCAAAACACATTGtACCGAATTAGATGGAAtCCTAAAAATCCAGCTACaCAtcAATAtGCTACTGCTATAGAGTGGTGCCAACATCAAGCAaGTACAATCGCTAAGCTATATaaacaaatcggcttaaaa

>NRS408contig00055 [organism=Staphylococcus aureus]

ATAtGTTtATGCTCCTTTCGTGTATAATGTTGTTATCAACCTAAGGAGGTGATAACATgCCCTTGaTATCTGATGAATTTGATACACTTaCTAAAGaCCAACAATATATCTTGTCcGTACTCTACAAAGATTATTtAGAATGTGTAAAGTtAGGTtCGgTtAAATTAACCTGCAATAATTTTGgAAGtgCTAAAGaTATACaTACAAAGTATTTtCAAAAaCTACATTTCGAAGATGTAAAaTACGATTTAAATAAACTTAAAAaCTCTGGGTTCCTAAACGGCGTGTATGCTAGTAACACTATTTATCATGTAACAATTTCAGACAAGACTGTTGTTTACTTTGAAAATGAGTTTAAAAACAaTTTAAAAaGTAtCATTGATAGCATTTCTAAAATTGCTTCAATAATTCCTGGTCTCTAGTTGGGTTTATAACTTCCCAATCATTTGCCATGAGGTCATCGGCTGAAGGTTGCCAATATCTGATAaGGTTTGTCCCATCGCTATTTGAAATGATGcATTGTAA

>NRS408contig00056 [organism=Staphylococcus aureus]

ACGCATTGTGTGTACCGTGACCGCCATTTttAACAAGCAATCTATCAATAAATTGTCCGTTGGGCTTCAATCTAGATAACATGTAATGATTGCCTGGACGCGCTTGTGTCATATAAATAaTTTTtGTTTtAGGGTCTACCCAAAaTGATTGCATTACTGCGTtAGTGTATGGCGATAAGTCAGTGATAAATTCTGGTTCTTGCTCTTTCGGTTCGAATCGATATTCAGTTGCTCGATATTCTTTGTAGTGTTCATCTACAGCTTTCTCGACTTTTTtAGTGAAAGCATCTAGTGTTGAATAATCATGATACAAACGATCTTGTAAAGTTTTGTGACCATAACCAGTATTATCAACACGTGCATCTGTTACTTCATTAATACCGTCGCCGTTATGACCTAGAATCATATTGCTAAAACGGCCATTTAGATATGTTAAAAAGTCAGAGACACTACTAGTAACTTGTAAGTGTTCATACTTAATTtGCTCTCCGTCATGTGCAAaTCTTCTTTgTTTCtATGgta

>NRS408contig00057 [organism=Staphylococcus aureus]

CTATgACAGCTTTTtACcGCAAAAGTtGGATATTGTCGTTTTCCCGTCAAAGTATGGTGGCGGAGCTGGACACGTTGAAaTTGTtGAGAGCGCAAATTTAAATACTTTCACATCATTTGGTCAAAACTGGAACGgTAAaGGTTGGACTAATGGCGTTGCGCAACCTGGTtGGGgTCCTGAAACTGTGACAaGACATGTTCATTATTATGACAATCCAATGTATTTtATTAGGTTAAACTTCCCTAACAACTTAAGCGTTGGCAATAAAGCTAAAGGTATTATTAAGCAAGCGACTACAAAAAaaGAGGCAGTAaTTAAaCCTAAAAAaaTTaTGCTTGTAGCCGGTCATGGTTATAACGATCCTGGAGCAGTAGGAAACGGAACAAACGAACGCGATTTTATACGTAAATATATAACGCCTAATATCGCTAAGTATTTAAGACATGCaGgaCatGAAGTTgCaTTATaCGGtGGCTCAAGtCAAtcAcAAGATATGTATCAAGATACtgCaT

>NRS408contig00058 [organism=Staphylococcus aureus]

tagcctaaatcgccttgcgcattgtttaagttaacttgtattgattgaCCGTTcGCCtCTGTCaTCTTATGTTGTtGCCAaCTCGTtGTTCCGAATTTATCATCTACATACTGCTTAGCTTGATTTAAAGCATTGTTAGATGTTtCTTTAaCAAATTTCTTCGTTAATTCTTCGTCAACTTTTTtATAGAACTGATACCATGTGCCACCGATTTTATATGTTGTGTACTCATCATTTGAATCGTCTGGATACCATGTTGCACGTGCCGTACTATCATCAACAACATAGACAACTAACAAGCCTGATTTCCCtAAAGTATTCGTAGTTGCTGAAACTTCAGAACCATCATCAACGCCATCTTCTTTAGGCGTCTCTAAAGTGCCTATATCTTTAAACGAGGGCGCATCTGTCGCGCTAGTGATATGAaTAATCCTAGATGTATTAATTGCGCTTAAAACGCTATCTATGGACTGTTCAGACGATTCAATTGCTTTACCATAATCATCAGTA

>NRS408contig00059 [organism=Staphylococcus aureus]

GTCAATTCaTTAACCGTAGCGCGCGGATTAGCAAATGTAAGCGTACCTTGTTCTTCTTTCTCTGGATAAGCACCATCAGTTACCGATACACCTTTTtGTACTTGACGGTTtAAGCCAATCGGTTGgTCTTTACCAGTACCTTTTAAGAACGcAGTTTCAAGCGCCACTGCAAATGCTTCTTCGATTTGAACACGAaCAAATCTTTCAAtCCACGCAGGACCAAAATCATTTAAAtCTTTTGGtAAAACAACAAACGCTGTCAATTtATTTTGAATTGCtGTTTCTTCACTGAACGCAGCATCtAaTtGaCCtttaatttcaccatagattttaccccaaacagccacgccagaaGTTTCGGATTTTAAGAACTTCAAACGCAAaCCAGCaTTTTTAATAcctaagtcagctaataatggatgattcgttgttaaatcttcgaagattctatcaattgtttcttctggtaaaagtttttcttctttatatccaacactcttattg

>NRS408contig00062 [organism=Staphylococcus aureus]

ATGAATTTCAAAATCACTCAGTGGAATCAAATAAGCTTGATGACGACAGTAGCGAAACTGGTAAAACGCCAGAATACTCTTTAGATGAGTACTTAAGATATGGATTTGCAAaTCAAAAAaCTtCGGTCAAAaTGACCTaTAAAATAATTGGAGaTTTTAAGCGAAAAGTACCGATTGACGAATTAGGTAACAAAAACGGCTTAGAATActGTAAaGAaGCGGTAGACCTGTTTGGCTGTATAATTTACCCAAATGATACAGAGATTGGTTTTTATTCtCctGAAAcaTTTTATCAAAGAaGcGAGAAAGTGATTcgatATCAATATAAtACTGaTACTGTATCTGCAACTGTCAGTACATtGGAATtAAGAACaGCTATAAAaGTTTTtGgAAAAAaGTATACAgCTGAGGAAAAGAAAAaTTATAATCCTATTAGAaCAaCTGACATtAAatattcaaatggttttataaaagaaggtacttatCGTAC

>NRS408contig00063 [organism=Staphylococcus aureus]

ATTTACCAGTACTATTAGCTATAGCCTTAGTGTGATATCCTACAGAACTTTTGGCTGAGCtCCAACCTGAACTTAATTTGCTTGGAATTCCTTTGATTCCAcTCCACATTTTTTtCATTTCGCcGCCAAAATGATTAGCATTTCTGCCCATTTtACTAAAAGCTTCGCCAGTTTTAGTTTTTACGCCGTCCCAAGCATTTCCAAACCATTTCTTTAtATTTTCTCTGTTTCTACGAGCTGTTTCTTCTTGTTCTTTAGCGTACTTATCACTTTTCTTCTTTTGGTCTTCTCTGAAGTTAGACCACCAACTTTTAAGGCCATTCCACCACTTTTCAGTATTTTTATATACACGACCACTGGATAAATCCATCTCTTTATCAATATCTTTATTTTGCTTTTTAACAACGTCTACTACAGCATCTTTTTTAGATTTTGCCTTTCTTACTtCATCCTTAtGTCTTTGATCAGCAATaGCTAATAaTTtaTC

>NRS408contig00065 [organism=Staphylococcus aureus]

aaaTATCAACTTTAGTAATCCtaCAGGGAcGTTGTATGTCACTATAAGTGATGTTTATTCAGGATCTCCGACATTGACcATTGAATAATTTTAAaCGACTAATTTTtAGTCGTTTtttATTTtGGATAAAaGGAGtAAACAAATGGATATCGGTACAATCGTAAGAACAATTTtATTAATAGTCGCATGGATCAaTCAGTTTTtAGCAATCAAACATATTTCTCCAaTCCCAGTTGACGAAGTGTTTATAAGCACAGTCGTTACTGGGATTGTTtCAATTTGgACGTGGTGGAAGAATAACAACTTTActCACGCATCTAAGAAAGGGCAaCAAAAAATTtaTgAAGTAAAAGCTGGCATTCAGTCAaCTGGTGGCGCACCTAAAGTGAACGGAGATGATAACAATGCCGTCGGTtAGGaCaTACAgTCAAGCTATtAGTtATCTTAAAAGTTTagAGGGTAaGGcGTGGAaT

>NRS408contig00066 [organism=Staphylococcus aureus]

AaTTAaCAtCTAATTCAGTCTTAGgaGtATCATTtATTTGCTTTTTTgCAAATTCTAACAGCttAtCcTGTGTTTCGATATCTTCaTTTGTTTGCGTATTAGCATATCGAATCCCAAACTGCTTTGCACTaTCTGCGACGTAGtCGACAATTGCTTTGtATTGATTGCGACcTGAATTATCAGCAaTTAAATTTAaGACTGTTGATTTTTCAGTTCcAACAtACATACAAGGCTTAGCTTTTTTATTtGAAGATATATCAATTCTATTTTtGGGgTCTTcTCcTAAAAATATCATTTCTAAAACGTGCTTGCCTTTATCAATATTTTTTATTAAaTcTATTGTTtCagACtGAaCCgacttagcaaaacaagaaatttgcttaatttgcttgccgtctaaaatcaacttatatattccaccttgagagcccttttttattgtaatactactgtttcattaccatacttgcaatc

>NRS408contig00069 [organism=Staphylococcus aureus]

aaatttctttggtttgcaCtcaaagtttGtGCTGATTTAGGTAAACTAGAAACTCTTTCaGCTTCTGCTTTTGCTTGTAATTTAGTTTCTTCAAATAGTtGGTTAATCATGTCACCGTACAaTTCATTTTGTCTTTCTTGCGGTTCACCGTTGTTTACTGCATTAATAAaTTCGTTTTtCGCATTTGCGAATGTTTCCGATAAATTTATAGTCATTTTAtGACCTCCTATTTTTGtATtAAAaaaGGAATCTTGAAAATCCATTTGCTGATAATTTACTATCTGCAACATCGATTTCTGATTCCTTTTCTTTCATATTTATTTTTtCAATTACTTTATTTGCTATTGCGTCAaTATCAATGTTAACcTCTGGCGTTTTACTTACCAAAgCTGTTACACGATTTAaTACATCTTTCgATAaCACTtGtGtATCGCTTGCtACAATTTGCATATTgtCGTTTttCAAAc

>NRS408contig00070 [organism=Staphylococcus aureus]

acagcagggtatcaattagatggtccacttgttgatatggtagaagcGGGAAgAGaCaCCTTAGATCAAATCaTAGAaGaCGAAAGAACTTTTTATtaTTTAGCAtCTTTGGATGATGACGATGATATtAATGATTCGTCGAACTGGATAAAAGCAAATCCCAACTTAGGTGTCTCTATAAATTTAGATGAGATGAAAGAaGAGTGGgAAAAAGCTAAGAGAACACCAGCTGAACGTGGAGATTTtATAACCAAAaGGTTTAATATCTTTGCTAATAATGACGAGATGAGTTTtATtGATTACCCAACACTCCAAAAAAATAATGAAaTTGTTTCTTTAGAAGAGCTGgAAGGCAGACCATGCACGATTGGTTATGATTTAtCAGAAaCaGAGGaCTTtaCAGCcGCGTGtGCTACTTTtgCGTTAGATAATGGTAAAGTTGCAGttttat

>NRS408contig00072 [organism=Staphylococcus aureus]

CACCAGTGATTAACTCACCACGTTTTAAGTTGCTTGATTTAAGGTTAACCACAGGTAGACCATCTAACGTATCACtGTTACGGTCATAAaTACGTtCTTTCGTTtCAGGATCTACAATTTTACGTAACAaGCTTCTgTTTtGTGTTTTTGaGaTAAAcGCATTtGCTTCTAATtCGTcATCTTCAaGTAATGCCtcTAAATCAATAATGTTATCTTGTGTGAAGTCACCTTTAATAACCTTATTAGTTTTTtCAATTGATTGTGCAATTGATTTACcGAATGGATTGTTACCTTGATTCAAAATACCCGCTTCATCAAaCTTTTTATAGAATGCTTCaGCAATCATAGGCTtCATTTCTTCAAAGAaTTGtGAaTAAGTGTAATTcAAAAACTCTTTtGTtACAGGTAAgATAACCCCTAATTTAAAcGCTcTcATAGTA

>NRS408contig00073 [organism=Staphylococcus aureus]

GAAAATAAaCGCAACgAACAAATGATTtACATTgTAAATGACAAATTAACCAAAAAAGAACTTGTCGAAATAGCAAGTGTTGCTGGCTTACAAGTTGATGAAAAaCAAACAAAAGCTGAAATTATCAACACTTTTGAGTCGCTAGAGTAGGTGGTTATATGACTACGCTAGCTGATGTAAAAAAaCGTATTGGCCTTAAAGATGAAAAGCAAGATGAACAATTAGAGGAAATTATAAAAAGTTGTGAAAGCCAGTTGTTATCAATGTTACCTATTGAAGTTGAACAAATACCGGAAAGGTTTAGTTACATGATTAAaGAAGTTGCAGTTAAACGCTACAACAGGATTGGTGCTGAAGGTATGACATCAGAAGCGgTTGACGgACGTAGCAaTGCGtATGAATtGAACGATTTcAAGGAgTaTGAAgCTaTTATTGAtaa

>NRS408contig00074 [organism=Staphylococcus aureus]

ctcctctttggttgtttcatcgtttatcaaaccttgcatttccattaatttttgaggtataccagcttttAACTGGATTTCGTATAACATTtGTTGAATGTGTGGTGGCACTTCTACCATTCCTTTCGTGTATAATTTAGTTATCTCCTAGTGAAAGGAGGTGATAAGTATGGAATTTAATGATTTTCAAAATTTCTTTGGTGAACTTAGTAATCAAGCCGAAAAaGAATTCGGTGGTGACAGTGACTTTTTTAGAGATAGAATAAATAAGTTGAAAGAAGATGCTCCTGAAAACGTATCTTACGAAATTATTtATTCAATAGCTTTATACGAAAGCTTAAAAGCTCAACAAGATATGAAAATTTTGAATACAGTTAAATATCTTTtAAATCGTGACTAGCAATATCCAACAATGATTTGCTCtGAGCATTATTAA

>NRS408contig00075 [organism=Staphylococcus aureus]

TtCTTTTTCGAAATTATTGTTGTATTTAATTTCtCCGTTtGTGAATACAAACTTTCtAGGTtCGAACTCTTCTTTGAATTTGATAGGCACATTGTTATCATCTACATCTAAACTATTGCGTAAACCGCCAGTATTAACGtaTCCGATAACTTCGTTTTtATCGTTtACTGTGATTTtCATTAcTtCcACCCcTCAATACGTTtAaTAGTAATTTtGTTTGCATTTGCACCAGAACCCGCACTTTTACCGATGTCATATAGGATATCAACGTCGATTCTGAATGTAGTATTGCTAGTTTTAGAAACaGAACATtCATATAaGCCACcaCCgTTGCCATCACTATcAAcTAGATttGTTTTaGatATTACtAtGGAaTTTGGCATaGAtGTTAAACTgACTTCTGCAAAAGTGCCTCCAGgatAAGTACCTG

>NRS408contig00076 [organism=Staphylococcus aureus]

CATTGGTGcTtCTTTAACTTtCTCTTgTAcGATTGATGAATCCACaGTTGATAGTTCAGTATTTAaCACACGTAAATTCTTATTTAAtAGTTTtAATTcTTcAaaaaTATCTTCTAATATTGCCATtGATTAAATCctCCTTAAAATTGGTTAGCTAGACGAATCATTAACTTGATACGATCTTCTATTTCTCTAGGGTCATCACTTTGTTCATTCAATCTTGCTAACAATTCAAATTGCTCTTCTAAAATTTCTTTTTtACGTTCGACGACAGTTAAATGTAATTGTGCTTCGATAACACGCCATTTTCCCCAACTTTCCATTTCAACCTTTCCTTTTTtCTTAAGTCTCGAAAGTGTGGATTTTGCATGTGTTTTCGATACTCCAAAAACTTcAACTACATCAtCAGGATtGAAATTGTCATATGTTGC

>NRS408contig00077 [organism=Staphylococcus aureus]CAAAGgCGaCAAACTAGCtCAAttGGTTAttGtGCCtATATGGaCACCTGAACTAAAGCAaGTGGaGgAaTtcGAGAGTGTTtCAGAACGTGGAGAAAAGGCTTCGGAAGTAGCGGaGTGTAAAGACATATTAGATCGAGTCAAGGAGGTTTTGGGGAAGTGAATTACATCATTACATTAGTTCTAATGGTTGTATTCATAGTAATATTTAACAATTTACTCAACAGATATATGGTTTTGTACAAAGAATTAGATTTATTTACATGCAGAATTGGCATGTTATTGGCCTTAaTCGTtCTAGTAGAATTTGCAAAGCAACAAAATaTGTTGGCTACATTGAGTGTTTTACTAATACTTTTATTCGTaGAAAAaCTTAGAATCATTCAAaGGAGTGgCGAgAAGTGAATAAGAaGgAaaCgTATTAT

>NRS408contig00084 [organism=Staphylococcus aureus]

TTAACaCGATAGAtGTTTTGACATGTTCAGAaCTTATAGaTAAcGGTCTGTTATTCTTAgTTACTaCAAAATTTAAAaCACCAGtCCcTCTATCTGATTCATAGAAACTGATGTTTGTGTCAATAAttGGATTATATTGTGATGTTGTTTGTAACTCGATTAAGTTATCgTCTTtcGAAAAaTTATCTACTACCATTAcTTAACCTCcTCGCCTTTtATAATGCTCCAACCGCTATTGCCACCAGTTCCAAAGTTTCTAaCTAAAAaTTGATGTGCAGATgCAAAGTTATTACGTCTTAATACTTGTGTtGTGTtGCCTGGTGTATTTGATTTCaCCTCTAACACCcAACCTGCAATACCTTTAAaGTCTTTAGGAAAaTCAGTAAATCGTTTTGATTCTTCAGTAG

>NRS408contig00089 [organism=Staphylococcus aureus]

gaaagcttcatctgtatttttatctataagaaatatattagctatgataaaccTTTGAAATGGTAaTGTtGGAAAATACCATTTTtCAaTAAATTTGATACAAtCCTCGaTTTTCTGTTCATCAAAATAtACATCATCTCGTGAATATATATGTTTTTGTAGATAATTAAAGAGATCAATTCTTTCTTTATTTAAAaTTaTCTTTCcTTGTTTCCACAAATTTATATATTCATCAACGTATTTATTACTAATCATAGGTAATCATCAGATGGCGTTTCTGTGTCTTCTTTCTCTTCGGGCAATAAaTCCGATAaTTGTTtGATTATTTTTtGATATGCAGCATCTCtAGCATTAAATAGTTTGGCTaCTGGtCTTTCccTTTcA

>NRS408contig00090 [organism=Staphylococcus aureus]

AAaTATCATAACCTTTCAGGACAAATTGAAGCTTATGATAAATCACTTAAAGAAATAAGATACACTCGAGATCTTTTCAACAAACATCTAAGCATGAaTAACGAaGACGCATTTGCTGGTTTGGAAaTGGTaGAAGATGAAATtACTAAAAaGCTACGAAGTGCtaTCAAAGAGTtCCAAAAaGTAGTGAAAGCGTTAGAcAaGCTTAACGGTGTTGAAAGCGATAACAAAGTTACTGATTTAACAGAGTGgCGGAAAGTGAATCAGTAaCATTCACTTCTTACTGGTAGCTCTTTAAAaTTAAaTGTTTGTAATGCTTGCATTTGAGTATCCTCCTTTTtCCTCAacaCCCACATTCAgCAGaCGGTTAtCGcAaTGActATCg

>NRS408contig00091 [organism=Staphylococcus aureus]

atacatttttctgtacaatatttgttaaaaattattgataatcgtcattgtaCGTAGTATTATGTTCTTAGGAGGTGTTCAGAAATATGAACAGTTTTAAGGATAGATTAAAGCAAATTATGTCTGAACGGAAGATATCTCAATCAGAGCTATCAAGAAGGACTGGTATTGGTAGAAACTCAATTAGCGATTATTTAAACGGAAAaTATGAAGCGAAACAAGACAAAGTCTTTGAACTAGCAAAGGCTTTAAACGTTAACgAAGCGTGGCTTATGGGGTTTGATATTTCTAAGAATAGAAAAATTGAAAATAACGACATCAcTtCCATATACAGTAAACtCACGCCTCCAAGACAAAGCAATGTActAAAaTATGCGaCTAATC

>NRS408contig00093 [organism=Staphylococcus aureus]

ctATAactcttCTGtttttAACCATATaCAGTGCCCAGTCTaCcACTTcACTAGCTGTAGGTTTtCGAGTCTTTGGATTAGGTAATCCCATGTATGCACCTCATTTCAATCAAAATAAAAaGCCAGTGCCTTAGCACTGACTCCTATACATTACTTACATTTACCAAACCAGAAGCATGCCCAGAAACTATATCCGAAGAATCCTTTAAGCATGGTGATCACCTCCTTTAAATACCGAAAATGGTtCTTATtAAGGCTATGACAATCGTACTAAAGATAGTCCCTACcAAACCGaGAATCCACATTTTCATATCACGTATATTTTTGTcGTTTtCTTTCTTATTTTTTtCgTtCTATCTgTCTTTCccTcTGgaTaG

>NRS408contig00094 [organism=Staphylococcus aureus]AGAATCATTGAAGAGGGTGAACTtAAAACCTATAATTCAGCTGGTAGCGATTTCGATTTACTAGAGGTTGAGCgACAAGATTtCAAAGTATCTGATTTACcGTCAAACGATGAATTGtATATTAAACaTACACTtGTAGACCTTAAACAACAAATTAAATTGGATTTATATTTAATGAATGAATATTAAtCTTTTTTCTtAGCTTTTtCTGATAAAGTGCTTTTtAATTTttGgCTGgCGCCTGACTTTTCAAAACTTTtGTTtAATGGGTTACTACGAGTAGTTTCTTGTTTTTtGTTTttATCTACcATAAaaTTCTCACcACCaTTCAACGtCTACaCtAGtAGGCGTTTTttATTTAGTAAAAtCatAAT

>NRS408contig00099 [organism=Staphylococcus aureus]

GAAATTGAACACTTACACATCAATACTGGTGGTAAAGAGCTTACTCAAGAGCAAATAGAAGAGGCTAAaGCTTTTATAGACAGTCAAGAATTTAAAGATATGaTTCGAGAaGCTAAAGAATCAcATCAAAGAGTTATGGAGTCTAAAATCACTGATAGAACTAAATTGTGATTAACAGCGCCTgTgTGGCgCTTTAATATAAAaGACGTCTATTTCAGCAGTGTTTGAAAGGAAGTTTaTAATGAAAaTAaCTAATTGCAAAatAAAAAaaGAAaCTATAGTaTATGAAGTTTTAACTAgTGGTAATcaaCCATTCaCTTATGAGTTACCTAAAgATTTATCGTCACATAAtGCGCgTAAATAC

>NRS408contig00100 [organism=Staphylococcus aureus]

AAGcATCTaGtCAGTTaGaTGCTTATTTTgAAGAAACTAAACGTTATGTGatgTCGAGACgATATCAAGGCATACCCAAAAaTATATATTATAAAAaTCAGCGCATCGAATAGGTGTGCTTTTtAATTTTtAAGGAGGAAATAAGCAATGGCAGAAGGACAAGGTTCTTATAAAGTAGGTTTTAAAaGATTATACGTTGGAGTTTTTAACCCAGAAGCAaCAAAAGTAGTTAAACGCATGACATGGGAAGATGAAAAAGGTGGTACAGTTGACCTAAATATCACAGGTTTAGCACCAGATTTAGTAGATATGTTTGCATCTAACAAACGTGTATGGATGAAAaaCAAGGTACTAATGAAGTT

>NRS408contig00102 [organism=Staphylococcus aureus]

TaGaTGAAAGTGATGAGGATACGCTTGAGTTATtAAGGTTTAGATATTGGGATTGTCCTATTGGTTGTTATGAATGGGAAGATATAGCACATTACTTTGGTACAAGTAAGACAAGTATATTACGTAGAAGGAATGCACTGATCGATAAGTTAGCAAAGTATATTGGTTATGTGTAGCGGACTTTtACCCtATGTAAGTCCGCATTAAAACAGTTTATTATGTTAGTATCAGATTAATATTTAAGGTTATTAAATGCTAATACGAcGCATGAACAAGAGGCGCATTACTAtGTGATGTGTCTTTTTATTtATGAGGTATGAACATGTtCAAaCTAATTGTAAATACATTACTacaCA

>NRS408contig00104 [organism=Staphylococcus aureus]

CACCTTGTATACAAAaTTTATATTTGTGTTCCGATGTTGCATCAATTCTTGCCAAACTTTTAAAAATAGCTGTTAGAGGGTTACCCCTATACCCCTTTACTCCCTAACACTACTTTttAAaCTTtaTAGTGAATTTGATGCAACATTGGAAaCAAACAGGGTTGAACCCTACAGCGAGAAGGGGAagAGGTGTTGTATCATTtGTTGCATCAATGTTGCATCACCAAAAATGAtaCAACACCTAAGaTTaCTTTTTACACcACCGTGTTGCATCaCTCAAAAAATGaTGCAACATTTgATACAACATCTAAAAATGTATATTTATtCAATATTtCTTAtATTataTCcTC

>NRS408contig00106 [organism=Staphylococcus aureus]

AATTAAgTGTCACTCCAAAaTTGATAATGTTAATTTtCTAATATGGTCATGAACATCTTGTACATAAGCTTTTtGATGAATTGATTCGAAGCCATGCTGATACTTTTTtAGCGGAATCGGATGATTGAGCTTCCTCAATCTTCCTAGCGACAAATCTTTTGCGAAATTGAGTTTTTtATTGATTTCTTCTAAATCGTCATtATTGATTCTTACTTtACtGAAAATTGCACCTGAgCTGATTGGTTtCTCGCCTTTTATAGcaTTTCTAaCTTCTTTCGCTATAaTTtCTTtCAACTcTTCTTTGGTTAaTGtGATTTGTTCCATaGTTtCCTCCTGTtACGACA

>NRS408contig00109 [organism=Staphylococcus aureus]

GTCTCCGCAaTTTTCTTAGCGTTTTCTTCAGATTTAAAATCTTGAGCATGGTTAACCATTTCAGAAGTTGTAAAACTTCCTGTGAAATCTTGATATACTACACGTTCTGTACCTtCTTtGTCGATTTGTACTAAAATAAACCTTTCTGTATTGTCGATAATTTCTTTtGCCATAaTTAAATGACCTCCTTAAATTTTTGtATAAAAaTAGTGCTAAGGATtACTCTtCcTCAGCACATTGTTGATTTTCTTTATTTTCTTGTATATACGCTTTtAaCATCGCGTTTtCTtGTGTtAACCTCATAATTTCCTGTGATAAATAaTgAATT

>NRS408contig00112 [organism=Staphylococcus aureus]

GATATTATTAAaTCCTTGAAGCACCTAATCGAGACCCAAGGGACGAGTTTGATACACCAATCCTTAAATCCGATGTGCTATCAATTGAAGATTTAAAaGAAGGAATGAAATTGAGTGGCACAGTTAGAAATGTAGTAGATTTTGGAGCATTTGTAGATATAGGTGTTAAACAATTAGATATTAACCAAGAaTATTTAACGCAGGTTGATGTTATAGCtCAAAAGTGTAATGCAGAaTTAAAATATCATCAATCTCTACTTCCTCAATAtCAGaCACcTAaCGATGAATCAGCTAAAAAATATTtGTGGCGtGTcTTAGT

>NRS408contig00113 [organism=Staphylococcus aureus]

gtttaaacgtcgtatcgatataggcggtagtgaataataactttagAGGaGACTtCCAAGAaGCTgAGGGtCtCGACATGTATtACGATCTAGAAACAGGACGCAAAGCGCTTTTAATTGGGGTAACTATTGGACCAGGTAACAACAGACATCACTCAATTTATTCTATCGGTCAAAGAGGTGTAAACCAATTCTTAAAAAACATCGCACCTCAAGTATCAATGACTGATTCAGGTGGACGTGTTAAACCGTTACCAGTGCAAAACCCAGCATATTTAAGTGaTGTTACTGAGGTtGgTAACtaTTACttAtACT

>NRS408contig00116 [organism=Staphylococcus aureus]

TtCCGCATCGTTACTAGTTGAAGGCCAATACATTAACCTATGTGGTTGATAAGTTGTaTCATCGAAGTAATCCATGCCAaCGATATCTGCGACTTTACGCCCAATAGCTTCATACTCATCTGCATTTACATTTCGTTTTAAAGGAATCACTAAACGCAGTCTTGGACTTATCTCTCTATGCTTATGTGTTGAATATAAACAaTATGCAAAATCATAAAACATAGATAaTATGTCGgCCATATCTTGAGCAGCATAATCGaTATCAAGTGTTAGCATTGAACGATTCATGaCTTGACCAGCACGCcGTTtACcTt

>NRS408contig00118 [organism=Staphylococcus aureus]

AaTAAaTTAAAaTTaGTAGGTGCTGATGTATGGACTTCTATTGAAAGTGCGTTTGCACCAGTAATGGAAGAATTAATCAAAAAGCTATCTATAGCGGTTGATTGGTTTTCCAATTTAAGTGAtGGTTCTAAAAGATCAATTGTTATTTtCGGTGGTATTGCTGCTGCAATTGGTCCTGtAGTTTTTGGATTAGGCGCATTTATAAGTACAATTGGCAATGCAGTAACTGTATTAGCCCCACTATTAGCTGGTATTGCAAAGGCTGATGGATTAaTTAGTTTTTtATCGACTAAAGtacctata

>NRS408contig00119 [organism=Staphylococcus aureus]

GTTCATTTAGACTGGCTAACATAGGGCTGTAAAATTCACTATCTTCATCTTTAACAGTTTTAATAAAACAGCCTTCAATCTCAGCTTTTTCTTCTGGCGTTCCATTTTTATACGTCTTAAATACCTCGGTGTGCTTTTCTGGTAATTTCATTTTAGGTGTATTAAACATTATTATCTCCCCTCTTTAATGATTTtATTtCTTTTCGAACAAAGACCTAATACTTCTTCACTAGGtCTTTCGAATAAGGTCACTTTAGAATTATTAGTGTAGTAaaCAaTAGGTGTATTTTGTGACTCATA

>NRS408contig00120 [organism=Staphylococcus aureus]

ATTTCAaCAACATCtGgATTtAaTAAGAAAAGCTTTGaTGGTTGATGaTAGATGTCTCGTtCAATTAGCACATATGCATTACCTTTTTCATTTCTGATtGTTTCAATTTGATTAATAAAAaTCAAAACTGcTCAGaGAATTATTCGGTGACACTgTAaGTAAATCAGaTACTTCTGtATTAACTACTTTATAATCTTCATACATTtCAaGGGCAAACTAGCCATCGAATTaGATAACTTTGTAATAGCTGAAAATATcGTTTcATTAGTTTCAAGCGtATTATTAATTACaCCCC

>NRS408contig00121 [organism=Staphylococcus aureus]

ttccattgcatgttgtcacctcccgcttaacaaaacctactataCACGATaCGTGTACTTGAGTCAACATAAAAGTTTGCTTTTCGTGTATTTTTTTGTTGAATaCCAAAAATAaTTGGGTTATACTATAGGTAAaTTTAAGGAGGTAaGAAAaTGgATAaaaaaGAATTAGCGAAATTTATAGGCAATAAAATCAGATACTATAGAACCAAATTGAACTTAACTCAAGATCAACTTGGaGAAAAACTaCAACACTAAAAAaGCTaCTATTTCAAATTATgaGACaGGGTaC

>NRS408contig00123 [organism=Staphylococcus aureus]

GTACGAGAAACAAGTTAAAAGGGATGCAGTTATTAAAGTGGGTCAGTTGTTTGAAAATATAAGGGAGTGTGGGAAATGATTAAAAAACTTAAAAaTATGGATGGGTTCGACATCTTTATTGTTGGAATACTGTCATTATTCGGTATAACCGCATTGCTACTtGTTGTCGCATTGCCTATCTATACAGTGGCTAGTTACCAAAaCAAaGAaGTaCATCAaGGGaCAATTACAGAtAAATatAAcaaaagacaagataaagaggacaaattctatattgtattagatgataaac

>NRS408contig00126 [organism=Staphylococcus aureus]

CCTATAAATaCaCAGAAGCTGAAGATTTCGAAATCTTAATTATAGCTtaTTCAATAGAtGgTGGAGCGATTAGTGCGATTGACATgACTAAAGTAGATAAtGAGCcTTTCCACGCTGATTATGAGACGTTTAAAATTGCTCTATTTGACCCTGCTGTAAAAAAgtAtGCATtcAATGCtAATTtCGAAAGAaCTTGTCTTGCtAAACATTTTAATAAACAGATGCCACCTGAAGAATGGATTTGCACAATGGTtAATTCAATGCGTATTGGCTTACCTGCTtCgC

>NRS408contig00127 [organism=Staphylococcus aureus]

TTCCACAATAAATTTGAATTAAAAgCAGAAGCTAACGGTATTGATGAATATGAATATGAATATGGAGTGAATGGTCGTTTCCAACGTGGATTTGCAACACTACCTGAGGCTGTAACAAAGAAACTTAAGGCGACTGGATACAGATTCCATGACACTACAAAAGCAGATGCGTTAACTGGCGAAGATTTAACAGCAATTCCACAACCTAAGGTAGATTCATCAACGGTTACACCAGGAGAGGTATAAAAaTAGGgCGTTAAGCCCtaTTtATTTTGTTtAAATTta

>NRS408contig00128 [organism=Staphylococcus aureus]

ACTATAACAAGTACGCTAATTaactttgttaatagagTtattcaaGGATtcGTTAATGTTGTAAACAAAGTTAGTCAAGGTATGACAAATGCAGTAAATAAAATAAAAAGCTTTATAGGAGATTTTGTGTCTGCAGgTGCTGATATGATCCGTGGTTTAATTAGAGGTATTGGACAAATGGCTGGCCAATTAGTAGATGCGGcTAAAAATGTTGCTAAGAAAGCTTTAGATGCAGCTAAAAGTGCTTTGGGtATTCACTCACCTTCACGTGAATTCAT

>NRS408contig00129 [organism=Staphylococcus aureus]

AAAGTTTTTtAACTACGAGGAACAAGTTAAATTACAATTCAAATCTAAAGATtGGTACTGGAACGCTtATTTTGAAGGACCAATAAAGCTGCACAAaGAATTTACAATACCTGTTAAGTTCACTATCAAAGTAGTACTAACAGATCCTTACAAATATTCAGTAACAGGAAATAAAAATACTGCGATTTCAGACCAAGTTTCAGTTGTAAAtAGTGGGACTGCTGACACTCCTTTAATtGTtGAAGCCCGAGCAATTAAATCATCTAGTTACTTttATG

>NRS408contig00133 [organism=Staphylococcus aureus]

GACaatgcatcaataaagacttaaaaCTTTTAGATTaTCGAGTTCAACAAATTTTAGAAGGTGTTCTATCAGAAAGTACCACATACGGTGATGCAAGAAATAAATTAGAAACATTGAAATTATTGCTGAATCTCATTTTAAAACcGAACATGCTTCAGTTATTTACAAaTTAGCATTGAAAAAGTTAGACAAAAAAaTCAACGCCACTCCAATTAAAGAGTGACGAAAAaGGAGGATTTCAAATGTTTAAGATTTTtAAAt

>NRS408contig00135 [organism=Staphylococcus aureus]

AGGAAATGAATCTAGTGTTCCACCATAGTCAGCATTAACCTGATACGCTTCTTCTCCTGTTTCTAAaTCGAAaGCCGTTAAATAGTTTCTATTATTTGGATTACtGTCTCCTGTATACCAaTAcAaGTaTTTTTCATCAAAAGTCACACCCTGcATTGGTTGGGTTTcgtttgttagtctcatagggatactgattttatgcaaaactttatcaatatttttatcaacatcgtctaaacttcttatctctatataat

>NRS408contig00138 [organism=Staphylococcus aureus]

TGaCAGCTAATGAtGaTGTAgAGGCGCcGAGTGATTACGTcTTTCGAgCGGAGGTAAGTGAGTGATgTGgATTACTATGACTATTGTATTTGCTATATTGCTATtAGTTTGTATCAGTATTAATAGTGATCATGCAAGAGAGATACAAGCACTCAGATATATGAATGATTATCTACTTGATGAAGTAGTTAAAACTAAAGGATACAACGGGTTAGAAGAATACAGGATTGAaTTG

>NRS408contig00157 [organism=Staphylococcus aureus]

TtCTCTTTCGCTTCTTCTTTACTCTCTGCCTCAACAACTGTAAACCTTTGATTaCtcttAGCTttAGTTATGTGTGTATGcTtgCGTCCTGTTGAAtCTTtGAaTgttgtGA
